# EAES/SAGES evidence-based recommendations and expert consensus on optimization of perioperative care in older adults

**DOI:** 10.1007/s00464-024-10977-7

**Published:** 2024-06-28

**Authors:** Deborah S. Keller, Nathan Curtis, Holly Ann Burt, Carlo Alberto Ammirati, Amelia T. Collings, Hiram C. Polk, Francesco Maria Carrano, Stavros A. Antoniou, Nader Hanna, Laure-Meline Piotet, Sarah Hill, Anne C. M. Cuijpers, Patricia Tejedor, Marco Milone, Eleni Andriopoulou, Christos Kontovounisios, Ira L. Leeds, Ziad T. Awad, Meghan Wandtke Barber, Mazen Al-Mansour, George Nassif, Malcolm A. West, Aurora D. Pryor, Franco Carli, Nicholas Demartines, Nicole D. Bouvy, Roberto Passera, Alberto Arezzo, Nader Francis

**Affiliations:** 1 Department of Digestive Surgery, University of Strasbourg, Strasbourg, FR USA; 2 Surgical Unit, Dorset County Hospital, Dorchester, Dorset UK; 3 SAGES, Los Angeles, CA USA; 4 Department of Surgical Sciences, University of Torino, Turin, Italy; 5 Department of Surgery, University of Louisville School of Medicine, Louisville, KY USA; 6 Department of General and Minimally Invasive Surgery, Busto Arsizio Circolo Hospital, ASST-Valle Olona, Varese, Italy; 7 Department of General Surgery, Papageorgiou General Hospital, Thessaloniki, Greece; 8 Department of Surgery, Queen’s University, Kingston, ON Canada; 9 Department of Surgery, University Hospital CHUV, Lausanne, Switzerland; 10 Department of Surgery, The University of Toledo College of Medicine and Life Sciences, Toledo, OH USA; 11 Department of Surgery, Maastricht University Medical Centre, Maastricht, The Netherlands; 12 Department of Colorectal Surgery, University Hospital Gregorio Marañón, Madrid, Spain; 13 Department of Clinical and Surgical Gastrointestinal Diseases, University of Naples “Federico II”, Via Pansini 5, Naples, Italy; 14 Department of Surgery, Hellenic Red Cross Korgialeneio Benakeio NHS, Athens, Greece; 15 Department of Surgery and Cancer, Imperial College London, Chelsea and Westminster Campus and the Royal Marsden Hospital, London, UK; 16 Department of Surgery, Yale School of Medicine, New Haven, CT USA; 17 Department of Surgery, University of Florida, Jacksonville, FL USA; 18 Department of Surgery, College of Medicine, University of Florida, Gainesville, FL USA; 19 Department of Colorectal Surgery, AdventHealth, Orlando, FL USA; 20 Cancer Sciences Unit, Faculty of Medicine, University of Southampton, Southampton, UK; 21 Long Island Jewish Medical Center and System Chief for Bariatric Surgery, Donald and Barbara Zucker School of Medicine at Hofstra/Northwell Health, Great Neck, NY USA; 22 Department of Anesthesia, Faculty of Medicine and Health Sciences, McGill University, Montreal, Canada; 23 Innovative Surgical Techniques, Endoscopic and Endocrine Surgery, Department of Surgery, Maastricht University Medical Center, Amsterdam, Netherlands; 24 Division of Nuclear Medicine, University of Torino, Turin, Italy; 25 Department of Surgery, Yeovil District Hospital, Higher Kingston, Yeovil, UK; 26 Complex Cancer and Exenterative Service, University Hospitals Southampton, Southampton, UK; 27 NIHR Southampton Biomedical Research Centre, Perioperative and Critical Care Theme, University of Southampton, Southampton, UK; 28 The Griffin Institute, Northwick Park and St Mark’s Hospital, Y Block, Watford Rd, Harrow, HA1 3UJ UK

**Keywords:** Frail, Elderly, Older adults/aged, Laparoscopy, Robotic surgical procedures, Perioperative care, Prehabilitation, Enhanced recovery after surgery (ERAS), Frailty, Abdominal Surgery

## Abstract

**Background:**

As the population ages, more older adults are presenting for surgery. Age-related declines in physiological reserve and functional capacity can result in frailty and poor outcomes after surgery. Hence, optimizing perioperative care in older patients is imperative. Enhanced Recovery After Surgery (ERAS) pathways and Minimally Invasive Surgery (MIS) may influence surgical outcomes, but current use and impact on older adults patients is unknown. The aim of this study was to provide evidence-based recommendations on perioperative care of older adults undergoing major abdominal surgery.

**Methods:**

Expert consensus determined working definitions for key terms and metrics related to perioperative care. A systematic literature review and meta-analysis was performed using the PubMed, Embase, Cochrane Library, and Clinicaltrials.gov databases for 24 pre-defined key questions in the topic areas of prehabilitation, MIS, and ERAS in major abdominal surgery (colorectal, upper gastrointestinal (UGI), Hernia, and hepatopancreatic biliary (HPB)) to generate evidence-based recommendations following the GRADE methodology.

**Result:**

Older adults were defined as 65 years and older. Over 20,000 articles were initially retrieved from search parameters. Evidence synthesis was performed across the three topic areas from 172 studies, with meta-analyses conducted for MIS and ERAS topics. The use of MIS and ERAS was recommended for older adult patients particularly when undergoing colorectal surgery. Expert opinion recommended prehabilitation, cessation of smoking and alcohol, and correction of anemia in all colorectal, UGI, Hernia, and HPB procedures in older adults. All recommendations were conditional, with low to very low certainty of evidence, with the exception of ERAS program in colorectal surgery.

**Conclusions:**

MIS and ERAS are recommended in older adults undergoing major abdominal surgery, with evidence supporting use in colorectal surgery. Though expert opinion supported prehabilitation, there is insufficient evidence supporting use. This work has identified evidence gaps for further studies to optimize older adults undergoing major abdominal surgery**.**

**Supplementary Information:**

The online version contains supplementary material available at 10.1007/s00464-024-10977-7.

The global population is aging. The increases in the proportion of older adults and in life expectancy have led to a proportional increase in older patients presenting for surgery [1]. There is inherent decline in physiological reserve and functional capacity with age that increase the likelihood of older adults being frail; in turn, frailty is associated with poor outcomes, longer hospital lengths of stays (LOS), loss of independence, and a high risk of morbidity and mortality after surgery [2]. Frailty is associated with advanced age, but chronological age does not accurately predict surgical outcomes [3]. The complex relationship between biology, environment, and social support paired with variable adoption of Enhanced Recovery After Surgery (ERAS) protocols and Minimally Invasive Surgery (MIS) may more accurately influence surgical outcomes in the older adult. Thus, optimizing perioperative care in older adults undergoing major abdominal surgery is imperative.

Perioperative optimization initially centered on ERAS, multimodal pathways introduced in the 1990’s to hasten time to recovery after surgery [4]. Since their introduction, ERAS protocols have been adopted by most surgical service lines, with universal reductions in LOS, morbidity, and post-discharge nursing needs by 30–50%. Adding MIS to an ERAS protocol further optimizes clinical outcomes and the recovery benefit [5]. More recently, attention has focused on prehabilitation, where interventions work to improve functional capability prior to a surgical procedure [6]. There is data to support prehabilitation improving functional capacity both preoperatively and postoperatively [7]. The principles of ERAS and prehabilitation are particularly well-suited for older adult, who may run a higher risk of complications [8]. Despite growing awareness of ERAS and prehabilitation, there is variation in their use and no clear guidance on the optimal preoperative and postoperative optimization pathways [9]. There is also wide variations in the incidence of co-morbidities in this population, heterogeneity in how elderly are defined, contention on the specific elements and application of ERAS and prehabilitation programs, and the metrics for measuring success. Therefore, a consensus on recommendations and high-level evidence to guide the usage of ERAS, prehabilitation, and perioperative optimization in older adults undergoing major abdominal surgery is warranted.

The goal of this work was to provide evidence-based recommendations to optimize perioperative care in elderly patients undergoing major abdominal surgery.

## Methods

### Participants

A steering group comprised of 24 stakeholders with experience in evidence synthesis, guideline development methodology and perioperative optimization from the Society of American Gastrointestinal and Endoscopic Surgery (SAGES) and European Association of Endoscopic Surgery (EAES), a methodologist with expertise in guideline development (S.A.), a medical librarian (H.A.B.), and a SAGES Guidelines Committee Fellow (A.R.) was convened in September 2020. As the proposed target audience of this study is patients and their surgeons, this study followed a patient-centered perspective, and included a patient representative (D.K.) in all phases to guide patient preferences. All participants were well versed in ERAS and prehabilitation principles, with experience in the development and application appropriate to their respective role.

### Time frame

The first online conference on October 24th, 2021, focused on team introductions, review of the process review, and expectations. An in-person consensus meeting was held in February 2022 at the EAES Winter Meeting, where articles and results of the systematic review explicitly created for the guideline and pertinent to a Key Question were provided to panel members for review. A consensus conference was held at the SAGES 2022 annual meeting, presenting an interim analysis of the systematic review. After presenting the data and audience discussion, participants voted on the proposed recommendations. The final voting meeting was held in June 2022. The panel subsequently convened for several virtual panel meetings between 2022 and 2023, where the literature search was updated, the GRADE evidence profile and summary of findings tables were reviewed, and the Evidence-to-Decision (EtD) tables and specific recommendations were generated. This was followed by critical review and revision by the authors and SAGES executive board review and approval in the latter part of 2023 and 2024.

### Selection of key questions and outcomes of interest

Perioperative optimization of older adults undergoing major abdominal surgery was divided into four main topic areas: (i) definitions of key terms, application, and outcomes, (ii) preoperative optimization, (iii) MIS, and (iv) use of postoperative patient optimization and ERAS programs. For topic area i, an appraisal of terminology was performed, reviewing the literature and proposing sets of definitions. This was expanded to include all relevant nomenclature related to the topics, such as the definition, assessment and implications of frailty. Topic areas ii, iii, and iv were subdivided into **PICO** (Patient, Intervention, Comparator, Outcomes) pre-defined Key Questions (KQs). Each KQ was formulated, revised, and unanimously approved by the steering group. Each KQ stem was separately assessed in colorectal, upper gastrointestinal, hepatobiliary pancreatic and hernia surgery. Then, the panel determined which outcomes should be considered based on their clinical and lived experience, considering both the patient and surgeon perspective in decision making. The primary outcomes adopted were LOS, readmissions and early complications.

### Data analysis

A systematic review and meta-analysis of articles collected from PubMed, Embase, Cochrane Library, and Clinicaltrials.gov databases was conducted and reported using PRISMA-S standards and written according to the SAGES Guidelines Committee’s standard operating procedure (Supplement 1) [10, 11]. For topic area i, the panel collectively selected the terminology that required refinement, and voted on working definitions of selected terminology, recommendations for application of each, and the outcomes for reporting results in studies focused on perioperative care of older adults. A modified Delphi method was used for consensus on agreement, with an agreement of 70% or more considered to reach consensus. For topic areas ii, iii, and iv, literature searches were conducted for each KQ individually, then consolidated under their overarching topic area. Identified studies were then reviewed by two independent reviewers to determine which areas were mapped into each KQ. The Covidence® platform (Veritas Health Ltd, Australia) [12] was used for title and abstract screening, followed by full-text review and data extraction for relevant papers. Two independent researchers assessed the eligibility of all studies, with a project lead (DK) resolving cases of disagreement. From the data extraction and Forest Plot results (generated using Cochrane RevMan [21]), evidence-based recommendations for each KQ were generated. Recommendations were graded using the Grading of Recommendations Assessment, Development and Evaluation (GRADE) approach [13–15] and the GRADE guideline development tool [16]. Where applicable, the Appraisal of Guidelines Research & Evaluation tool (AGREE II) [17] and Essential Reporting Items for Practice Guidelines in Healthcare (RIGHT) checklist was used [18]. Cochrane Risk of Bias and Newcastle–Ottawa Scale tools were used to assess study quality. [19, 20]. For results with insufficient evidence, expert opinion from a consensus-based approach from the steering group was recorded.

### Inclusion and exclusion criteria

Clinical studies in older adults (over 65 years) undergoing major abdominal surgery, defined as upper or lower gastrointestinal, hepatobiliary, or hernia, and describing preoperative optimization, use of minimally invasive approaches, postoperative optimization, and ERAS were included. The study designs considered were: randomized controlled trials; comparative non-randomized studies; prospective and retrospective case series (of more than 10 patients; and systematic review and meta-analysis. Articles were eligible for inclusion if published between January 1, 2000, to February 27, 2023. Articles were excluded if they were studies on children (under 18 years of age), narrative reviews, commentary or editorial formats, animal studies, or if published in non-English language.

### Evidence appraisal

Methods outlined in the Grading of Recommendations Assessment, Development and Evaluation (GRADE) approach handbook [22] were used to appraise the available evidence. GRADEPro evidence tables were created from the groups’ data extraction and analysis. The highest level of data available was used for tables; less rigorous data addressing the same outcomes was not considered nor used in decision making. The group judged the certainty of evidence using the risk of bias across available studies, and the certainty of evidence was evaluated at the individual study level. If concerns arose in any of the risk of bias assessments certainty was downgraded [19]. Data were imported for each KQ into Evidence to Decision (EtD) tables (Supplement 4), which served as the framework through which recommendations were developed.

### Development of recommendations

Outcomes from the Evidence to Decision (EtD) Tables were reviewed by the group as desirable or undesirable effects for the interventions [16]. The group then discussed the magnitude of desirable and undesirable effects, listed as anticipated absolute effects for the magnitude and 95% confidence interval (CI), the certainty of evidence, variation in values that may be assigned to outcomes, and the balance of these effects. Absolute percentage differences were calculated by the GRADEPro software based on the available data. The group voted on the overall certainty of evidence based on the risk of bias, inconsistency, indirectness, and imprecision of individual outcomes. After choosing whether the balance of these considerations favored the intervention, comparator, or combination, the panel discussed the acceptability and feasibility of this judgment.

For each decision, both the available evidence as well as pertinent additional considerations taken either from panel experience or interpretation of evidence were discussed. Based on the balance of effects and the acceptability and feasibility of a favored option, the panel voted on the final recommendation for that KQ. While serial voting was used to come to a consensus on individual components of the EtD, a supermajority (defined as ≥ 80% agreement), was used as the cut-off for consensus agreement. GRADE guidelines were followed to determine strong versus conditional recommendations. A strong recommendation occurred when the true effect was close to the estimated effect. If there was some uncertainty in the evidence, the strength of the final recommendation was downgraded as conditional. If there was no identified evidence for a KQ, an expert recommendation was stated based on the consensus of the experts within the steering group. Subgroups were addressed in the discussion for the justification for each recommendation and are specified for each KQ.

### Guideline document review

All recommendations drafted were distributed to the full steering group for review and revision of the content, to ensure completeness, and accuracy. For external review, the manuscript was sent to the SAGES Guidelines Quality Assurance Task Force to minimize the introduction of bias in the recommendations. The final document was submitted to the SAGES Board for approval and published online (https://www.sages.org) for public comment an additional quality assurance.

## Results

Evidence synthesis was conducted across the three subjects of prehabilitation, MIS, and ERAS, resulting in 20,106 total papers reviewed, with 10,901 unique items screened across the 24 KQs.

### Terminology, recommendations for application, and outcomes

From the evidence synthesis, the group agreed on terminology that required standardization across the population level and perioperative periods (ERAS), then collectively voted on definitions and recommendations for their application in studies on older adults undergoing major abdominal surgery. All working definitions are seen in Table 1. In brief, any person aged 65 years or older meets the definition of “older adult”. “Older adult” in used in place of elderly per age inclusive language guidelines from the American Medical Association and Gerontological Society on Aging. Frailty was agreed as a state of increased vulnerability to stress from aging-associated decline in reserve and function across multiple physiologic systems. The panel agreed that any study on perioperative care of older adults should include one of the many available indices to measure frailty and assign a score to each patient, but could not reach consensus on any individual instrument. The panel recommended that frailty and comorbidity be separated and reported individually. The panel agreed that a risk score should be assessed for comorbidity burden and the name and score should be detailed, but there was no consensus on specific assessment tools.

**Table 1:** Proposed definitions for perioperative care of elderly surgical patients undergoing major abdominal surgery

Term	Definitions
Elderly	Any person 65 years or older. “Elderly” and “Older Adult” can be used interchangeably
Frailty	State of increased vulnerability to stress from aging-associated decline in reserve and function across multiple physiologic systems
Prehabilitation	Process of improving functional capability of a patient prior to a surgical procedure. Prehabilitation programs use a combination of physical and cognitive exercise, nutritional supplementation, smoking cessation, and stress reduction to improve preoperative functional status and postoperative outcomes. The elements are timing before surgery are not universal
Anemia	A hemoglobin level less than 13 g/dL in men and less than 12 g/dL in women
Alcohol cessation	Stopping consumption of alcohol-containing beverages for at least 30 days prior to surgery, with a lack of alcohol-related withdrawal symptoms, including nausea; vomiting; fast heart rate; agitation; headache; sweating; and delirium tremens present at the time of surgery
Tobacco cessation	The process of discontinuing tobacco smoking and any nicotine-containing replacements, including patches, chewing gums, lozenges, nasal spray, and inhalers for at least 30 days prior to surgery
Minimally Invasive Surgery (MIS)	Any laparoscopic, robotic, endoluminal, or assisted procedure
Conversion to open	Change from a MIS to open surgical approach outside of specimen extraction to complete a procedure
Enhanced Recovery After Surgery (ERAS)	Multimodal perioperative care pathways designed to achieve early recovery after major surgery by using evidence-based interventions. ERAS protocols encompass multiple components across preoperative, intraoperative and postoperative periods, a distinction from prehabilitation programs

Alcohol and smoking cessation were agreed upon as no consumption or any alcohol-containing beverage or nicotine-containing product for 4 weeks prior to surgery. Anemia in adults was agreed as a hemoglobin level less than 13 g/dL in men or less than 12 g/dL in women. The panel agreed that anemia should be included in any study reporting perioperative care in older adult population and corrected before any operation, but not on the timing, level to correct to, or the need to reassess. MIS was agreed to cover any laparoscopic, robotic, or assisted procedure; there was no agreement on including endoluminal procedures. The panel agreed that use of MIS versus open should be reported, and the type of MIS (laparoscopic or robotic or assisted) should be stated. Conversion to open was agreed as the inability to complete the dissection through an MIS approach, usually requiring an incision larger than that required to remove the specimen. If a conversion from MIS to open occurred, it should be clearly reported. Prehabilitation was agreed upon as the process that occurs between the time of diagnosis and the beginning of treatment, and includes physical, nutritional and psychological assessments to prepare a patient for the stress of surgery by improving their preoperative functional status. The panel agreed that a prehabilitation program should be implemented in elderly patients undergoing surgery but were not able to agree upon the timing or elements for a prehabilitation program from the literature. Prehabilitation was distinguished from ERAS—multimodal perioperative care pathways designed to achieve early recovery after major surgery by using evidence-based interventions—as ERAS protocols encompass components across the preoperative, intraoperative and postoperative periods and prehabilitation programs occur before surgery. There was a strong recommendation to adopt ERAS protocols in all elderly patients undergoing elective major abdominal surgery. The group recommended the specific elements used and compliance with ERAS protocol elements should be described. There was consensus that the outcomes that should be reported in perioperative care of older adults undergoing major abdominal surgery included: delirium; postoperative complications; LOS; and readmission. None of the screened articles investigated the effect of any of the above interventions on postoperative quality of life (QoL) in older adult, and given the heterogeneity of different QoL tools, the expert panel could not make a recommendation for including QoL. The quality of evidence and strength of the recommendations are seen in Table 2.

**Table 2:** Recommendations for reporting on perioperative care of elderly surgical patients undergoing major abdominal surgery

Statement	Quality of evidence	Strength of recommendation
Studies should include a frailty index score as part of the baseline demographic data	Low	A
Researchers should separately report a frailty index and comorbidity burden	Low	B
The elements within the prehabilitation program being tested should be clearly stated	Low	A
Researchers should state the length of time that the prehabilitation program runs for	Low	A
The intervention for each prehabilitation element should be described in detail e.g., the exercise program or the combinations of programs being used	Low	A
The time frame a prehabilitation program is put into place before surgery and the length of time that the prehabilitation program runs for should be stated	Low	B
The time between the end of the prehabilitation program and the date of surgery should be stated	Low	B
The compliance rate of participants on the various aspects of the prehabilitation program should be stated	Low	A
Reporting on anemia should include a clear definition of the parameter and level (units) that define anemia	High	A
The intervention used to correct anemia, and the time between correction and surgery should be stated	High	A
If MIS is used, the type should be stated (laparoscopic, robotic, endoluminal, or assisted, with single or multiport distinction)	High	A
If conversion to another platform occurs, then this should be clearly stated	Low	B
The specific elements in an ERAS protocol should be reported, and if the whole protocol was used or select elements only	High	A
Compliance with the ERAS pathway or select elements should be reported	Low	B
Any study on alcohol cessation should include the type of alcohol consumed, frequency of use, time between stopping use and surgery, and the presence or absence of any alcohol-related withdrawal symptoms at the time of surgery	Low	A
Any study on tobacco cessation should include the type of nicotine used, frequency of use, and the time between stopping use and surgery	Low	A
Postoperative outcomes of delirium, in addition to complications, length of stay, and readmission should be reported	Low	B

## Key questions

With the terminology agreed upon, each KQ stem was for colorectal, upper gastrointestinal, hepatobiliary pancreatic and hernia surgery separately. Search strings and full PRIMSA flow diagrams for each KQ are seen in Supplement 2. Meta-analysis was only conducted in the topic areas of MIS and ERAS as there were not enough quality data in the prehabilitation section.

## Prehabilitation (topic area ii, KQ1–KQ16)

Prehabilitation and preoperative sections (topic area ii, KQ 1–16) were addressed according to item-specific KQ. Literature search, review and synthesis was performed, resulting in 169 studies assessed at the full-text level. 144 were excluded and 9 items were duplicates leaving 16 unique studies across all KQs among which 5 RCT and 11 observational studies were found. Most studies were considered at low to moderate risk of bias by the reviewers, the main characteristics are summarized in Supplement 3 and underpinned the following weak recommendations.

### KQ1: Should a prehabilitation (vs. no prehabilitation) program be used in elderly patients undergoing colorectal surgery?

#### Recommendation

The panel recommends that a prehabilitation program be used prior to colorectal surgery in elderly patients (Expert opinion based on low certainty of evidence).

#### Summary of evidence

70 studies were assessed at the full-text level and 16 were identified for KQ1. Of the 16, five were RCTs, eight retrospective papers and three prospective case–control studies. Ten studies involved patients undergoing colorectal surgery, while data about colorectal procedures were extracted from six other studies. Fifteen studies analyzed the 30-day complications, thirteen the LOS and six the 30-day readmission rates [23–39].

#### Benefits of intervention

The analysis of 30-day complications showed better results in the prehabilitation group, including the 30-day postoperative LOS. However, the differences between the two groups were not statistically significant. Thirty-day readmission rates were analyzed by only six papers, showing no statistically significant differences between the two groups. [25, 28, 33, 35–37].

#### Harms and burdens

None of the analyzed outcomes showed undesirable effects for the intervention, which the panel considered critical.

#### Certainty in the evidence of effects

Low certainty of evidence was defined for all outcomes. Although the group considered the majority of included studies with low risk of bias and 5 RCTs have been included in the analysis, the heterogeneity among the included studies was very high. Similarly, the definition of elderly was heterogeneous, as well as definitions of prehabilitation programs. Finally, postoperative outcomes derived from colorectal procedures were merged with other types of surgical procedures in some included studies, thus the real results of the comparison between prehabilitation and no-prehabilitation program on postoperative outcomes are difficult to obtain.

#### Conclusions and research recommendations

Although there are limitations in the volume and strength of the current literature, the panel members agreed from the data and clinical experience that the application of a prehabilitation program can positively impact postoperative outcomes in elderly patients undergoing colorectal procedures, especially in terms of 30-day postoperative complications, LOS, and 30-day readmission rates.

### KQ2: Should a prehabilitation (vs. no prehabilitation) program be used in elderly patients undergoing Upper GI surgery?

#### Recommendation

The panel recommends a prehabilitation program be used prior to Upper GI surgery in elderly patients (Expert opinion based on low certainty of evidence).

#### Summary of evidence

24 studies were assessed at the full-text level with three studies addressing this question. In two studies, upper GI surgery patients were included, but no data could be extracted on postoperative outcomes related specifically to the prehabilitation in older adults undergoing upper GI surgery [24, 34]. One prospective study specifically analyzed the adoption of a prehabilitation program in older adults undergoing upper GI surgery and was included in the final analysis [39].

#### Benefits of intervention

In the included study, the analysis of 30-day complications showed similar results between the two groups, while no data were reported in terms of LOS and 30-day readmissions.

#### Harms and burdens

The 30-day postoperative complications did not show undesirable effects for the intervention, which the panel considered critical.

#### Certainty in the evidence of effects

Low certainty of evidence was defined for the 30-day postoperative complications. The included study had a high risk of bias due to unpaired comparison between the groups.

#### Conclusions and research recommendations

Based on the data and clinical experience, the panel members recommend that a prehabilitation program be used before surgery to positively impact postoperative outcomes after upper GI surgery in older adults. Future research is needed to develop stronger evidence in this topic area.

### KQ3: Should a prehabilitation (vs. no prehabilitation) program be used in elderly patients undergoing hepatobiliary pancreatic (HPB) surgery?

#### Recommendation

The panel cannot recommend that a prehabilitation program be used in elderly patients prior to HPB surgery (Expert opinion based on very low certainty of evidence).

#### Summary of evidence

28 studies were assessed at the full-text level. Three studies included patients who underwent HPB surgery [24, 33, 34]. Data on 30-day mortality, LOS and 30-day readmissions were retrieved, but results related specifically to the HPB surgery could not be extracted.

#### Benefits of intervention

There was no data on postoperative complications directly related to the HPB surgery that could be extracted from the included studies,

#### Certainty in the evidence of effects

Low certainty of evidence was defined for the analyzed outcomes. Despite the presence of an RCT, it was not possible to extract evidenced-based data related to HPB surgery.

#### Conclusions and research recommendations

There was no evidence to support a recommendation for prehabilitation, but from the clinical experience of the panel members, it was suggested that a prehabilitation program could positively impact postoperative complications in elderly patients undergoing HPB surgery. Further research is needed to develop evidence in this topic area.

### KQ4: Should a prehabilitation (vs. no prehabilitation) program be used in elderly patients undergoing Hernia surgery?

#### Recommendation

The panel cannot recommend that a prehabilitation program be used in elderly patients prior to Hernia surgery (Expert opinion based on very low certainty of evidence).

#### Summary of evidence

16 studies were assessed at the full-text level. One study made it to extraction in this KQ [33], but no study met the inclusion criteria to be included in the final analysis.

#### Conclusions and research recommendations

There is no evidence in the literature to support a prehabilitation program in older adults undergoing hernia surgery, but from clinical experience the panel members suggested adoption of a prehabilitation program in older adults undergoing hernia surgery could lead to improved postoperative outcomes. Further research is needed to develop evidence in this topic area.

### KQ5-8: Should perioperative optimization (vs. no optimization) of anemia be used in elderly patients undergoing major abdominal surgery (including Colorectal, Upper GI, Hernia, and HPB)?

#### Recommendation

The panel cannot recommend that anemia be corrected prior to Colorectal, Upper GI, Hernia, or HPB surgery in elderly patients (Expert opinion based on low certainty of evidence).

#### Summary of evidence

Only one prospective study was included in the analysis, involving 248 abdominal surgery patients. The patients were not matched, and the relevant outcomes were 30-day complications and LOS [29].

#### Benefits of intervention

The comparison between the two groups showed no significant difference in terms of 30-day postoperative complications or LOS.

#### Certainty in the evidence of effects

Low certainty of evidence based on a prospective study. The panel considered the sample small in size and the results preliminary.

#### Conclusions and research recommendations

There is no evidence in the literature reviewed to support correction of anemia in older adults undergoing colorectal, upper GI, hernia, or HPB surgery. Based on the panels experience, members suggest correcting anemia prior to major abdominal surgery in elderly patients. Further research is needed to develop evidence in this topic area.

### KQ9-12 Should smoking cessation (vs. no smoking cessation) be applied in older adult patients undergoing Colorectal, Upper GI, Hernia, or HPB surgery?

#### Recommendation:

The panel cannot recommend perioperative smoking cessation prior to colorectal, Upper GI, Hernia, or HPB surgery in elderly patients (Expert opinion based on very low certainty of evidence).

#### Summary of evidence

Six studies were identified from the screening as eligible to be included in the analysis, but after the full-text review, no study met the inclusion criteria.

#### Conclusions and research recommendations

The effect of smoking cessation on outcomes after surgery is well accepted in general, but no study in the current perioperative literature focused on this aspect in older adults. While there is no evidence to support smoking cessation in older adults prior to colorectal, upper GI, hernia, or HPB surgery in elderly patients, the clinical experience of the panel members suggests preoperative smoking cessation 4 weeks before major abdominal surgery in older adults, and future research to develop evidence on this topic area.

### KQ13-16: Should alcohol cessation (vs. no alcohol cessation) be applied in older adult patients undergoing Colorectal, Upper GI, Hernia, or HPB surgery?

#### Recommendation

The panel cannot recommend alcohol cessation in elderly patients undergoing Colorectal, Upper GI, Hernia, or HPB surgery (Expert opinion based on very low certainty of evidence).

#### Summary of evidence

No studies were identified regarding alcohol cessation or the comparison between alcohol cessation versus no alcohol cessation/ active alcohol consumption in elderly patients undergoing colorectal, upper GI, hernia, or HPB surgery.

#### Conclusions and research recommendations

It is generally accepted that alcohol cessation is associated with better outcomes after surgery than no alcohol cessation. However, there was no evidence found in the literature to support alcohol cessation in older adults prior to colorectal, upper GI, hernia, or HPB surgery over no alcohol cessation. It was noted that various definitions exist for alcohol excess, which risks heterogeneity. Based on the panel experience, members suggest stopping perioperative alcohol intake in elderly patients 4 weeks before undergoing major abdominal surgery, and future research to develop evidence on this topic area.

## Role of MIS in older adults (topic area iii, KQ17-KQ20)

### KQ17: Should MIS (vs open) Colorectal surgery be used in older adults?

#### Recommendation

The panel recommends MIS over open surgery in elderly patients undergoing Colorectal surgery (Conditional recommendation based on moderate certainty of evidence).

#### Summary of evidence

349 studies were assessed at the full-text level. 197 were extracted, and 82 included in the analysis. Among the 82 studies included in the systematic review and meta-analysis, 81, 72 and 7 observational studies were selected for the outcomes of 30-day complications, LOS and 30-day readmission, respectively [40–121].

#### Benefits of intervention

The outcomes for MIS compared to open colorectal surgery were:

1. 30-day complications: (81 observational studies of 131,241 participants) absolute difference 101 fewer patients per 1000 (95% CI 121 fewer to 78 fewer) [40–65, 67–121]
2. Length of stay: (72 observational studies of 87,465 participants) mean difference 2.48 days fewer (95% CI 2.9 fewer to 2.05 fewer) [40–48, 51, 53–62, 64, 65, 67–78, 80–82, 84–95, 97–104, 106–118, 120, 121]
3. 30-day readmission: (7 observational studies of 16,075 participants) absolute difference 15 fewer patients per 1000 (95% CI 46 fewer to 36 more) [54, 66, 72, 82, 110, 111, 117]

The evidence is primarily based on short-term observational studies. However, the panel recognized the impact of the large number of studies and patients included (Supplement 5).

#### Harms and burdens

None of the outcomes showed undesirable effects for the intervention.

#### Certainty in the evidence of effects

The certainty of evidence was evaluated as moderate based on the outcomes of 30-day complications, LOS and 30-day readmission. These outcomes were limited by containing unmatched data and burdened by high heterogeneity (Supplement 4).

#### Decision criteria and additional considerations

While MIS is widely accepted as the gold standard for colorectal surgery, concerns remain about its use in older adult population, where cardiac and pulmonary comorbidities could affect ventilation, insufflation, and hemodynamic safety. The expert panel came to its opinion considering colorectal disease as a whole, without making distinctions on malignant or benign conditions or the location of the disease process in the lower GI tract.

#### Conclusions and research recommendations

From the data and clinical experience, the panel recommends MIS over open colorectal surgery in older adults. The choice for either approach should always be made on a patient-by-patient basis, considering specific comorbidities, expertise, and equipment availability.

### KQ18: Should MIS (vs open) Upper GI surgery be used in older adults?

#### Recommendation

The panel recommends that elderly adults undergoing Upper GI surgery may benefit from MIS over open surgery (Conditional recommendation based on very low certainty of evidence).

#### Summary of evidence

251 studies were assessed at the full-text level. 99 were extracted. The analysis considered 18 observational studies for the outcome of 30-day complications, 14 observational studies for the outcome LOS and 2 observational studies for the outcome of 30-day readmission [122–139]. No RCT evidence met the inclusion criteria.

#### Benefits of intervention

Three outcomes were considered with desirable anticipated effects for MIS as compared to the open technique, including 30-day complications, LOS, and 30-day readmission. Overall, the panel evaluated these outcomes as critical.

1. 30-day complications: (18 observational studies with 10,431 participants) absolute difference 71 fewer patients per 1000 (95% CI 98 fewer to 40 fewer) [122–139]
2. Length of stay: (14 observational studies with 4134 participants) mean difference 2.84 days fewer (95% CI 4.24 fewer to 1.45 fewer) [122, 123, 125–127, 129–132, 134–136, 138, 139]
3. 30-day readmission: (2 observational studies with 328 participants) absolute difference 47 fewer patients per 1000 (95% CI 66 fewer to 8 more) [123, 126]

Short-term observational studies were taken into consideration for the present evidence.

#### Harms and burdens

None of the outcomes showed undesirable effects for the intervention.

#### Certainty in the evidence of effects

The certainty of evidence was considered very low based on three selected outcomes (30-day complications, LOS and 30-day readmission). These outcomes were limited by unmatched data; interval estimates cross statistical and clinical significance (Supplement 4).

#### Decision criteria and additional considerations

The analysis focused on overall upper GI diseases, without any discrimination in terms of etiology (i.e., oncologic or functional) or localization along the upper gut, as most of the studies which met inclusion criteria focused on gastric rather than esophageal conditions. Moreover, the panel decided not to include bariatric-related gastric resection in this guideline, as it was already expanded on a previous review from the SAGES Guideline Committee [140].

#### Conclusions and research recommendations

From the literature and clinical experience, the panel recommends that MIS over open surgery be used in elderly patients undergoing upper GI surgery. Any further decision on which technique to be used should be made on a patient-centered basis, always considering specific comorbidities and conditions.

### KQ19: Should MIS (vs open) HPB surgery be used in older adults?

#### Recommendation:

The panel recommends that elderly adults undergoing HPB surgery may benefit from MIS over open surgery (Conditional recommendation based on low certainty of evidence).

#### Summary of evidence

223 studies were assessed at the full-text level, including 8 that were added during the extraction process. 86 studies were extracted. Only observational evidence was found to meet the inclusion criteria for selected outcomes in 37 studies. The analysis considered 36 studies for the outcome of 30-day complications, 25 articles for the outcome LOS and 5 observational studies for the outcome of 30-day readmission [52, 124, 141–175]. No RCT evidence was included.

#### Benefits of intervention

Outcomes considered in this analysis showed desirable anticipated effects for MIS as compared to the open technique, listed as follows. Overall, the panel evaluated these outcomes as critical.

1. 30-day complications: (36 observational studies with 6222 participants) absolute difference 122 fewer patients per 1000 (95% CI 152 fewer to 88 fewer) [52, 124, 141–145, 147–175]
2. Length of stay: (25 observational studies with 5862 participants) mean difference 3.85 days lower (95% CI 5.12 lower to 2.59 lower) [141–153, 156–158, 161–163, 166–168, 173–175]
3. 30-day readmission: (5 observational studies with 2180 participants) absolute difference 21 fewer patients per 1000 (95% CI 50 fewer to 21 more) [146, 161, 167, 168, 174]

Short-term observational studies were taken into consideration for the present evidence.

#### Harms and burdens

None of the outcomes showed undesirable effects for the intervention.

#### Certainty in the evidence of effects

Low certainty of evidence was considered for 30-day complications and LOS. Very low certainty of evidence was defined for 30-day readmission. The outcome of 30-day complications was marked by moderate heterogeneity. However, intervals are estimated beyond decision thresholds. 30-day readmissions interval estimates cross decision thresholds (Supplement 4).

#### Decision criteria and additional considerations

The analysis developed the Key Question of treating all HPB pathologies without distinction on the etiology or localization of disease, due to the minimal number of studies on this topic related to HPB disease. As most of the articles meeting inclusion criteria dealt with benign cholecystitis conditions, the panel unanimously voted to submit a single, comprehensive recommendation on the subject.

#### Conclusions and research recommendations

From the evidence and clinical experience, the panel recommends MIS over open surgery for elderly patients undergoing HPB surgery. The recommendation was defined as conditional due to *low* and *very low* certainty of evidence as well as for the intrinsic variety of the topics expanded on the present KQ. Further research is needed to develop evidence in this topic area beyond cholecystectomy for benign disease.

### KQ20: Should MIS (vs open) Hernia surgery be used in older adults?

#### Recommendation

The panel recommends that elderly adults undergoing Hernia surgery may benefit from MIS over open surgery (Conditional recommendation based on very low certainty of evidence).

#### Summary of evidence

67 studies were assessed at the full-text level and 18 were extracted. The analysis considered 5 observational studies for the outcome of 30-day complications and 5 articles for the outcome LOS [176–180]. In this, four articles are unique and the Neupane study is listed twice under complications, once for inguinal and once for ventral hernia surgery. No study met the inclusion criteria for the outcome of 30-day readmission. No RCT evidence was included.

#### Benefits of intervention

The analysis showed desirable anticipated effects for MIS in hernia surgery as compared to the open technique. Overall, the panel evaluated these outcomes as critical.

1. 30-day complications: (5 observational studies with 29,285 participants, with the Neupane article is included once for inguinal and once for ventral surgery) absolute difference 30 fewer patients per 1000 (95% CI 58 fewer to 20 more) [176–179]
2. Length of stay: (5 observational studies with 29,040 participants) mean difference 5.08 days fewer (95% CI 10.6 fewer to 0.44 more) [176–180]

Short-term observational studies were taken into consideration for the present evidence.

#### Harms and burdens

There were no outcomes with undesirable effects for the intervention. However, none of the included studies reported 30-day readmission rates, which was a critical outcome.

#### Certainty in the evidence of effects

Certainty of evidence was considered very low both for 30-day complications and LOS outcomes. The first was affected not only by a high risk of bias in 2 out of 3 studies but also from considerable heterogeneity and intervals estimates; the latter had a high or unclear risk of bias in 4 out of 5 articles, characterized by tremendous heterogeneity and intervals estimates. (Supplement 4).

#### Decision criteria and additional considerations

In the present analysis, overall hernia surgery was taken into consideration irrespective of the localization of the defect or the difference between primary or recurrent abdominal wall repair. None of the included studies considered the 30-day readmission rate, significantly restraining the strength of the recommendation.

#### Conclusions and research recommendations

Based on the evidence and clinical experience, the panel recommends MIS over open surgery for elderly adults undergoing hernia surgery. The recommendation was defined as conditional due to *low* and *very low* certainty of evidence. The decision on whether the MIS technique should be preferred over the traditional approach should always consider specific patients’ comorbidities and conditions. Further research is needed to develop evidence in this topic area.

## Role of ERAS in older adults (topic area iv, KQ21-KQ24)

### KQ21: Should ERAS (vs conventional care) be used in elderly patients undergoing Colorectal surgery?

#### Recommendation

The panel recommends that elderly adults undergoing Colorectal surgery benefit from ERAS programs over conventional care (Strong recommendation based on moderate certainty of evidence).

#### Summary of evidence

67 studies were assessed at the full-text level, 48 were extracted, and 9 were included in the analysis—one RCT [181] and 8 observational studies; no observational studies were found to meet the inclusion criteria. Literature research found no article considering the outcome of 30-day complications or LOS.

#### Benefits of intervention

The analysis showed both strong benefits and small undesirable effects for ERAS programs in colorectal surgery. Considering the panel’s wide experience with patients undergoing colorectal surgery, it was emphasized that the negative outcomes were largely limited in frequency.

1. 30-day complications: (1 RCT study with 150 participants) absolute difference 308 fewer patients per 1000 (95% CI 445 fewer to 144 fewer) [181]
2. Length of stay: (1 RCT study with 150 participants) mean difference 2.25 days fewer (95% CI 3.52 fewer to 0.97 fewer) [181]
3. 30-day readmission: (1 RCT study with 150 participants) absolute difference 13 fewer patients per 1000 (95% CI 42 fewer to 163 more) [181]

#### Harms and burdens

The outcome considered showed some undesirable effects for the intervention, which the panel considered small in size.

1. 30-day readmission: (1 RCT study with 150 participants) absolute difference of 13 more patients per 1000 (95% CI 42 fewer to 163 more) [181]

Considering the results from the analysis, the panel pointed out the very small sample size and even smaller event rate in conjunction with a confidence interval that crosses multiple standards of clinical meaningfulness. However, the estimated effects of the evidence reviewed ranged from moderate benefits to minimal harms.

#### Certainty in the evidence of effects

Moderate certainty of evidence was defined for the outcome of 30-day complications. In this case, the number of events was below the threshold of 300. Hence the certainty was downgraded for imprecision. The panel assessed the certainty of evidence of the outcome LOS as moderate due to the high risk of bias of two trials and to the lack of reporting of LOS to follow-up, missingness, and planned statistical analysis. The readmission rate was considered with low certainty of evidence because of the aforementioned very small sample size and event rate. The panel evaluated all three selected outcomes as critical (Supplement 4).

#### Decision criteria and additional considerations

The item expanded on in this KQ was considered a priority by the panel, with a large desirable effect despite the analysis results. Undesirable effects were evaluated as small in size, even though the patients living far away from their hospital could be more affected by ERAS programs than those living closer. Nonetheless, any positive impact and undesirable anticipated effects should be considered for patients as part of shared decision making. In addition, readmission practices vary greatly from one country to another, possibly confounding the results. The panel determined that there was *probably no important uncertainty or variability* in how much people acknowledged the main outcome. The patient representative thought patients valued the complication outcomes highly.

#### Conclusions and research recommendations

Based on the evidence and clinical experience, use of an ERAS protocol was strongly recommended for older adults undergoing colorectal surgery. The panel considered the intervention was *probably* feasible to implement in 83% of cases. Issues restraining universal implementation of ERAS were lack of home support and transportation for patients living far away from the operative institution. The panel suggests more assistance at the institutional level including social support could address these limitations. Moreover, the panel agreed that more standardized data collection are needed to generate stronger evidence, such as multi-institutional studies and collaborative groups and registries for large, international studies.

### KQ22: Should ERAS (vs conventional care) be used in elderly patients undergoing Upper GI surgery?

#### Recommendation

The panel recommends that the older adult population undergoing Upper GI surgery may benefit from either ERAS programs over conventional care (Conditional recommendation based on low certainty of evidence).

#### Summary of evidence

12 studies were assessed at the full-text level. Five were extracted. Only two RCTs were analyzed [182, 183], and no comparative observational studies were included. The outcome of 30-day complications, LOS and 30-day readmission were the only ones addressed in the present analysis.

#### Benefits of intervention

30-day complications and LOS outcomes showed desirable anticipated effects for ERAS over traditional care in patients undergoing upper GI surgery. Overall, the panel considered both outcomes as critical and small in size.

1. 30-day complications: (2 RCT studies with 299 participants) absolute difference 2 more patients per 1000 (95% CI 368 fewer to 342 fewer) [182, 183]
2. Length of stay: (2 RCT studies with 299 participants) mean difference 0.83 days lower (95% CI 1.65 lower to 0.01 lower) [182, 183]

Complications were assumed to be trivial by the Expert Panel, but may change if additional data were available.

#### Harms and burdens

The outcome considered showed undesirable effects for the intervention, which the panel considered critical and small in size.

1. 30-day readmission: (2 RCT studies with 299 participants) absolute difference 69 more patients per 1000 (95% CI 6 more to 226 more) [182, 183]

Expert Panel highlights a small number of events and a confidence interval that crosses minimally important differences.

#### Certainty in the evidence of effects

Very low certainty of evidence was defined for the outcome of 30-day complications. Although one trial was methodologically acceptable, the other did not explain the randomization process properly. The intervention-control groups were not fully compared enough to derive the quality of the randomization process indirectly. The two studies included in the analysis of the outcome of 30-day complications had opposite findings, with one demonstrating fewer complications with ERAS and the other less with conventional care. This may be explained by the lack of definition of complications and unknown comparability between cohorts in the high risk of bias trial.

In addition to the small sample size and relatively small event size, there was a wide confidence interval with the estimated effects ranging from large benefit to large harm with ERAS. Concerning the LOS outcome, the small sample size increases the fragility of this outcome. Low certainty of evidence was considered for the outcomes of LOS and 30-day readmission. Selected articles reported the same issues on randomization mentioned above. In addition, a small sample size increased the fragility of the outcome of LOS. This, together with the small number of events and a confidence interval that crosses minimally important differences, burdened the outcome of 30-day readmission (Supplement 4).

#### Decision criteria and additional considerations

The panel saw the issue developed in the present KQ as a priority. A possibly important uncertainty or variability in how people value the main outcome was identified, and thus, possibly due to the long distance traveled by patients. In these cases, readmission and LOS may be more important than complications as they are very impactful to patients living far away from the hospital or operative institution.

#### Conclusions and research recommendations

The panel states that balance between desirable and undesirable effects does not favor the intervention or the comparison. The recommendation is based on a low certainty of evidence. Nonetheless, it was common among the panel members that the intervention was probably feasible to implement. This takes into account global considerations such as local culture, MIS technology, lack of follow-up due to long distances travelled by patients, and lack of social support. The advice is for a closer follow-up after discharge and implementation of institutional support. Concerning possible research priorities, more studies on ERAS implementation in resource-limited environments are needed, as well as multi-institutional RCT specifically looking at ERAS in upper GI surgery in elderly patients and prospective observational studies due to feasibility issues of conducting RCTs.

### KQ23: Should ERAS (vs conventional care) be used in elderly patients undergoing HPB surgery?

#### Recommendation:

The panel recommends that the older adult population undergoing HPB surgery may benefit from an ERAS program over conventional care (Conditional recommendation based on a very low certainty of evidence).

#### Summary of evidence

17 studies were assessed at the full-text level. Five were extracted. Only two observational comparative studies addressing main outcomes were used for the final recommendation [184, 185]. No RCTs were included.

#### Benefits of intervention

All three main outcomes expressed desirable anticipated effects for ERAS over traditional care in patients undergoing upper HPB surgery. Overall, the panel considered all outcomes as critical and small in size.

1. 30-day complications: (2 observational studies with 265 participants) absolute difference 85 fewer patients per 1000 (95% CI 335 fewer to 343 more) [184, 185]
2. Length of stay: (2 observational studies with 265 participants) mean difference 2.03 days lower (95% CI 5.01 lower to 0.95 higher) [184, 185]
3. 30-day readmission: (2 observational studies with 265 participants) absolute difference 29 fewer patients per 1000 (95% CI 68 fewer to 77 more) [184, 185]

#### Harms and burdens

There were no undesirable effects with ERAS for any critical outcomes.

#### Certainty in the evidence of effects

Very low certainty of evidence was assessed for all main outcomes. Both studies were judged to be of high risk of bias based on the Newcastle–Ottawa Scale, due to unclear descriptions of how patients were selected for either intervention and a lack of reporting on follow-up. Additionally, the two included studies had opposite findings, introducing considerable heterogeneity into the analysis (I2 84%). Lastly, small sample sizes and large confidence intervals increase the fragility, and thus imprecision of this outcome; indeed, the estimated effects range from large benefits to large harms (Supplement 4).

#### Decision criteria and additional considerations

The item addressed in the present KQ was considered a priority by the panel, with small desirable anticipated effects in favour of the intervention. The panel determined that there was possibly important uncertainty or variability in how much people acknowledged the main outcome.

#### Conclusions and research recommendations

The intervention probably favored the balance between desirable and undesirable effects. The recommendation was defined as conditional due to a very low certainty of evidence.

### KQ24: Should ERAS (vs conventional care) be used in elderly patients undergoing hernia surgery?

#### Recommendation

The panel cannot recommend an ERAS programs over conventional care for elderly patients undergoing hernia surgery (Conditional recommendation based on the absence of evidence on this topic).

#### Summary of evidence

Five studies were assessed at full-text screening, none met the inclusion criteria. No observational studies or RCTs were found to meet the inclusion criteria on this topic.

#### Benefits of intervention

There were no desirable effects with ERAS for any critical outcomes due to a lack of evidence on the subject.

#### Harms and burdens

There were no undesirable effects with ERAS for any critical outcomes due to a lack of evidence on the subject.

#### Certainty in the evidence of effects

Literature research found no evidence on this topic (Supplement 4).

#### Decision criteria and additional considerations

Considering the complete absence of evidence on the matter, which was considered a priority by the Expert Panel, it feels like stating there was possibly important uncertainty or variability in how much people acknowledge the main outcome. Nonetheless, a common awareness emerged among the panel members regarding the possible gain from pushing for more ERAS surgical operations, which are already provided with effective, enhanced perioperative programs. Indeed, Nissen techniques and paraesophageal hernia (PEH) already have a low LOS. On the other hand, esophagectomy has limited and poor-quality data in elderly patients to draw any conclusion.

#### Conclusions and research recommendations

There is no evidence in the literature to support using an ERAS protocol over conventional care for elderly patients undergoing hernia surgery, but from clinical experience the panel members suggested that use of ERAS protocols in older adults undergoing hernia surgery could lead to improved postoperative outcomes. Further research is needed to develop evidence in this topic area.

## Discussion

This collaborative SAGES-EAES Consensus addressed perioperative optimization in older adult patients. The main goal was to provide evidence-based recommendations for perioperative prehabilitation in the older adult population in order to standardize preoperative workup, implement patient’s physiological, functional and psychological status, and surgical treatment. Additionally, the vast expertise in perioperative management and MIS of the Expert Panel further enriched and drove the consensus discussion, especially in the topics with low level of evidence. What the panel found during this process was a lack of standard definitions for prehabilitation, ERAS, and their components, as well as a lack of strong evidence outside of clinical experience to support their use in the perioperative care of older adults undergoing major abdominal surgery.

In the current work, almost all of the given recommendations were conditional, based on low to very low certainty of evidence, with the exception of KQ21 dealing with ERAS programs in colorectal surgery. In many key questions, the panel could not provide an evidence-based recommendation due to lack of evidence, small sample size, or high risk of bias reported. In these cases, the panel made suggestions based on their clinical experience to provide guidance on the practical application of MIS, prehabilitation, and ERAS topics for practicing surgeons. With this approach, the panel reached consensus on providing conditional recommendations supporting the adoption of preoperative optimization among older adults across all specialities and the project achieved its objectives to inform the global perioperative community on perioperative care of older adults.

With the lack of standardized terms and evidence exposed, the project added additional value in providing universal definitions and recommendations for reporting on perioperative care components. These definitions can be applied in future studies to ensure investigators use common terminology and outcomes. Despite the wide acceptance of these concepts, gaps in the literature across surgical specialties in the risk stratification of comorbidity and frailty, the application of MIS, and the details for application of prehabilitation were also highlighted. This investigation can help guide future work for evidence-based clinical application of these perioperative care concepts in older adults.

### Implementation

Given the diversity of the experts and stakeholders involved in this project representing European and North American practice, we believe that it is feasible to successfully implement these definitions, recommendations, and suggestions into clinical practice and that stakeholders will accept the recommendations. The primary considerations regarding the implementation of this Evidence-Based Recommendations and Expert Consensus include costs and support for developing prehabilitation and ERAS programs. Furthermore, some of the recommended techniques, such as MIS, require specialized knowledge and skills acquisition. Lastly, in order to achieve the full benefit of these recommendations, standardizing aspects of perioperative management in these specific groups of patients is required.

### Limitations of this consensus

One of the main limitations of this Evidence-Based Recommendations and Expert Consensus is the low certainty of evidence for most of the key questions, except in appraisal the role of MIS in colorectal surgery. This project also did not address insurance coverage and cost analysis to society as it took a patient-centered perspective. In the development of the recommendations, we were not able to consider certain aspects of diversity, equity, and inclusion due to unavailability in the literature that was reviewed thus may limit their generalizability. Finally, these recommendations represent the current practice and the adoption and implementation of the recommendations will be regularly updated in the future with the needs for future studies to develop evidence proposed.

## Conclusions

With the increasing life expectancy, perioperative optimization is essential as a risk mitigation toward achieving enhanced postoperative value among older adult patients. This collaborative Evidence-Based Recommendations and Expert Consensus provides a valuable tool to the perioperative community in standardizing definitions for perioperative care components and guiding the decision process concerning use of prehabilitation, ERAS, and MIS in elderly patients undergoing major abdominal surgery. These recommendations and suggestions can reduce inappropriate age-related inequity in access to surgical intervention and perioperative optimization.

## Disclaimer

The Evidence-Based Recommendations and Expert Consensus are intended to provide the best available approach to medical conditions as established by a systematic review of available data and expert opinion. The suggested approach may not necessarily be the only acceptable given the complexity of the healthcare environment and the condition specifically treated. This Evidence-Based Recommendations and Expert Consensus is intended to be flexible, as the surgeon must always choose the best suited-to-patient approach and other clinical variables at the moment of decision. This Consensus applies to all appropriately credentialed physicians, regardless of specialty, and addresses the clinical situation in question. This work was developed under the auspices of SAGES and EAES, the Consensus and Guidelines committee, and approved by the Board of Governors. The recommendations of each Consensus undergo multidisciplinary review and are considered valid at the time of production based on the data available.

### Supplementary Information

Below is the link to the electronic supplementary material.

Supplement 1 PICO Questions and Literature Search Strategies
Supplementary file1 (DOCX 122 KB)

Supplement 2 PRISMA Flow Diagrams
Supplementary file2 (DOCX 614 KB)

Supplement 3 Quality Assessment for KQ1-KQ16
Supplementary file3 (DOCX 21 KB)

Supplement 4 Evidence to Decision (EtD) tables for KQ17-KQ24
Supplementary file4 (DOCX 103 KB)

Supplement 5 Forest plots for KQ17-KQ24
Supplementary file5 (PDF 349 KB)

Supplement 6 Quality assessment for KQ17-KQ24
Supplementary file6 (DOCX 39 KB)
