# Supplementary material for: EAES/SAGES evidence-based recommendations and expert consensus on optimization of perioperative care in older adults
Source: Surg Endosc. 2024 Jun 28;38(8):4104–26. doi: 10.1007/s00464-024-10977-7 (PMC11289045; doi:10.1007/s00464-024-10977-7)
Supplement: Supplementary file 1 — Supplement 1 PICO Questions and Literature Search Strategies Supplementary file1 (DOCX 122 KB) [file 464_2024_10977_MOESM1_ESM.docx]

**EAES/SAGES Evidence-Based Guidelines on Optimization of Perioperative Care in Older Adults**

# PICO QUESTIONS

## Perioperative optimization (prehabilitation))

P= Elderly patients

I= intervention is preoperative optimization

- 1. Nutritional
  2. Exercise
  3. Neurocognitive dysfunction, including counselling
  4. Optimization of preoperative anaemia
  5. Alcohol and smoking cessation

C=standard care

O= specific outcomes using either – it depends on what has been reported – length of stay, readmissions, complications (Clavien–Dindo classification or using comprehensive complication index), mortality

Q1: Should a prehabilitation (vs. no prehabilitation) programme be used in elderly patients undergoing colorectal surgery?

Q2: Should a prehabilitation (vs. no prehabilitation) programme be used in elderly patients undergoing Upper Gastrointestinal (UGI) surgery?

Q3: Should a prehabilitation (vs. no prehabilitation) programme be used in elderly patients undergoing hepatobiliary pancreatic (HPB) surgery?

Q4: Should a prehabilitation (vs. no prehabilitation) programme be used in elderly patients undergoing hernia surgery?

Q5: Should perioperative optimization (vs. no optimization) of anaemia be used in elderly patients undergoing colorectal surgery?

Q6: Should perioperative optimization (vs. no optimization) of anaemia be used in elderly patients undergoing upper gastrointestinal surgery?

Q7: Should perioperative optimization (vs. no optimization) of anaemia be used in elderly patients undergoing hepatobiliary pancreatic surgery?

Q8: Should perioperative optimization (vs. no optimization) of anaemia be used in elderly patients undergoing hernia surgery?

Q9: Should smoking cessation (vs. no smoking cessation) be applied in elderly patients undergoing colorectal surgery?

Q10: Should smoking cessation (vs. no smoking cessation) be applied in elderly patients undergoing UGI  surgery?

Q11: Should smoking cessation (vs. no smoking cessation) be applied in elderly patients undergoing HPB surgery?

Q12: Should smoking cessation (vs. no smoking cessation) be applied in elderly patients undergoing hernia surgery?

Q13: Should excess alcohol cessation (vs. no alcohol cessation) be applied in elderly patients undergoing colorectal surgery?

Q14: Should excess alcohol cessation (vs. no alcohol cessation) be applied in elderly patients undergoing UGI surgery?

Q15: Should excess alcohol cessation (vs. no alcohol cessation) be applied in elderly patients undergoing HPB surgery?

Q16: Should excess alcohol cessation (vs. no alcohol cessation) be applied in elderly patients undergoing hernia surgery?

## Role of Minimally Invasive Surgery (MIS)

P= Elderly patients

I= MIS

1. Upper gastrointestinal
2. Colorectal
3. Hepato-pancreato-biliary
4. Henia

C=open

O= Optimized outcome by length of stay, readmissions, complications (Clavien–Dindo classification or using comprehensive complication index), mortality

Q17: should laparoscopic and/ or robotic (vs open) colorectal surgery be used in the elderly?

Q18: should laparoscopic and/ or robotic (vs open) Upper GI surgery be used in the elderly?

Q19: should laparoscopic and/ or robotic (vs open) HBP surgery be used in the elderly?

Q20: should laparoscopic and/ or robotic (vs open) hernia surgery be used in the elderly?

## Postoperative optimization and enhanced recovery after surgery (ERAS)

P= Elderly patients

I= ERAS in the postoperative phase

1. Early Nutrition
2. Early ambulation
3. Multimodal Pain management
4. Goal-directed Fluid management
5. Early removal of Tubes and drains

C= conventional recovery

O= outcome

Q21: should ERAS (vs conventional care) be used in elderly patients undergoing colorectal surgery?

Q22: should ERAS (vs conventional care) be used in elderly patients undergoing upper GI surgery?

Q23: should ERAS (vs conventional care) be used in elderly patients undergoing HPB surgery?

Q24: should ERAS (vs conventional care) be used in elderly patients undergoing hernia surgery?

**Literature Search Strategies**

| **Summary: Run Dates: 22 March 2021 to 1 April 2021; and 27 February 2023**  **Librarian: Holly Ann Burt** | | | | |
| --- | --- | --- | --- | --- |
| **OVERALL**  **SEARCH RESULTS** | Total items identified by database searches | | | 19,737 |
|  | Total trials identified in searches | | | 537 |
|  | Total items located by handsearching trials, databases, and references | | | 368 |
|  | Total duplicates identified (across KQs, by HAB, in Endnote, in Covidence) | | | 8,667 |
|  | Total unique items screened | | | 10,901 |
| **Databases searched (Coverage)** | | | | |
| **Medicine**  Embase/Elsevier (1947-2021)  [PubMed](https://www.ncbi.nlm.nih.gov/pubmed/10653237)/NLM (1809/1966-2021) | | **Systematic Reviews**  Cochrane Library*/ Cochrane (2009-2021) | **Clinical Trials**  Clinicaltrials.gov/NLM (2000-2021) | |
| Databases hand-searched for articles published by the trials retrieved by Clinicaltrials.gov and Cochrane: EU Clinical Trials Register/EuraPharma, Clinicaltrials.gov, PubMed, and Google Scholar | | | | |
| *For Cochrane searches: Replace [MH xxxxx] with the appropriate Cochrane MeSH search term | | | | |

## Topic 2

| **TOPIC 2: Perioperative optimization (prehabilitation)**  (KQ1-KQ12 combined in this first screening) | | |
| --- | --- | --- |
| **Topic 2**  **Search**  **Results** | Total items identified by database searches | 4,017 |
|  | Additional articles located by handsearching | 65 |
|  | Total trials identified | 192 |
|  | Total duplicates | 1,265 |
|  | Total items screened | 2,634 |
|  | Records excluded | 2,542 |
|  | Unique items to be split between KQ1-KQ16 | 92 |

### KQ1

| **KQ1: Should prehabilitation (vs. no prehabilitation) programme be used in elderly patients undergoing colorectal surgery?** | | |
| --- | --- | --- |
| **Database** | **Final search strategies** | **Results** |
| PubMed  KQ1  (22 Mar) | (("Aged, 80 and over"[mh] OR "Frail Elderly"[mh] OR elderly[tiab] OR "older adult" [tw] OR septuagenar*[tw] OR octogenar*[tw] OR nonagenar*[tw] OR centenarian*[tw] OR supercentenar*[tw]) AND ("Colorectal Surgery"[mh] OR ((abdomen[tiab] OR "anal canal"[tiab] OR "anal canal"[mh] OR anus[tiab] OR bowel[tiab] OR colon[tiab] OR colon [mh] OR rectum[tiab] OR rectum[mh] OR "Colon, Sigmoid"[mh] OR Sigmoid[tiab]) AND ("surgical procedures, operative"[mh] OR "general surgery"[mh] OR surgery[sh] OR resection[tiab] OR surg*[tiab])) OR ((rectal [tiab] OR Colorectal[tiab] OR abdominal[tiab]) AND Surger*[tiab])) AND ("Preoperative Exercise"[mh] OR ((preop*[tw] OR "pre-op"[tw] OR Prehabilit*[tw] OR Pre-habilit*[tw]) AND (diet[mh] OR diet[tiab] OR "Nutrition Therapy"[mh] OR nutrition[tiab] OR (healthy[tiab] OR eating[tiab]) OR Exercise[mh] OR Exercis*[tiab] OR walk*[tiab] OR swim*[tiab] OR flexibil*[tiab] OR precondition*[tiab] OR physiotherapy [tiab] OR fitness [tiab] OR "physical activity"[tiab] OR cycl*[tiab] OR bicycl*[tiab] OR train*[tiab] OR Cognition[mh] OR "Cognitive Behavioral Therapy"[mh] OR Cognitive[tiab] OR Counseling[mh] OR Counsel*[tiab] OR Counsel*[ot])) OR "Preoperative Care"[mh]) AND ("Clinical Study"[pt] OR "Comparative Study"[pt] OR "Epidemiologic studies" [mh] OR "Evaluation Study"[pt] OR "Meta-Analysis"[pt] OR "Multicenter Study"[pt] OR "Systematic Review"[pt] OR "Validation Study" [pt] OR randomized[tiab] OR analysis[tiab] OR study[tiab] OR studies [tiab] OR "Practice Guideline"[pt] OR "practice guidelines as topic"[mh] OR guideline[title] OR guidelines[title])) NOT ("Case Reports"[pt] OR "case report*"[tiab] OR "case stud*"[tiab] OR ("animals"[MH:noexp] NOT "humans"[MH]) OR rat[tiab] OR rats[tiab] OR mouse[tiab] OR mice[tiab] OR dog[tiab] OR dogs[tiab] OR porcine[tiab] OR infant[mh] OR (child[mh] NOT adult[mh]) OR children[title] OR child[title] OR infant[title] OR infants[title] OR "Endometrial Neoplasms"[mh] OR "Ovarian Neoplasms"[mh] OR "Urinary Bladder Neoplasms"[mh] OR "Urogenital Neoplasms"[mh] OR "Whipple disease"[mh] OR "Whipple's disease"[tw] OR "Whipple disease"[tw] OR "Bladder cancer*"[tw] OR "Endometrial cancer*"[tw] OR "Ovarian cancer*"[tw] OR "Renal cancer*"[tw] OR Gynecologic[tiab] OR Urologic[tiab] OR Urothelial[tiab] OR Urethra[tiab] OR ("1905/01/01"[pdat]:"1999/12/31"[pdat])) | 1,129 |
| Embase  KQ1  (22 Mar) | (('very elderly' OR 'frail elderly' OR elderly:kw,ti,ab OR 'septuagenarian*':kw,ti,ab OR 'octogenarian*':kw,ti,ab OR 'nonagenarian*':kw,ti,ab OR 'centenarian*':kw,ti,ab OR 'supercentenarian*':kw,ti,ab) AND ('colorectal surgery'/exp OR 'abdominal surgery'/exp OR 'abdominal surgery' OR 'rectum surgery'/exp OR 'rectum surgery' OR (('abdomen'/exp OR abdomen OR 'anal canal'/exp OR 'anal canal' OR 'anus'/exp OR anus OR 'colon'/exp OR colon OR 'rectum'/exp OR rectum OR 'intestine'/exp OR intestine:ti,ab OR 'sigmoid'/exp OR sigmoid) AND (resection:kw,ti,ab OR 'surgery'/exp OR surgery:kw,ti,ab OR surgical:kw,ti,ab)) OR 'colorectal surgery') AND ('preoperative care'/exp OR 'preoperative exercise'/exp OR 'preoperative exercise' OR (('preop*':ti,ab OR 'pre op':ti,ab OR 'Prehabilit*':kw,ti,ab OR 'Pre-habilit*':kw,ti,ab ) AND ('diet'/exp OR diet:ti,ab OR 'diet therapy'/exp OR 'diet therapy' OR 'nutrition'/exp OR nutrition:ti,ab OR 'healthy eating'/exp OR 'healthy eating' OR 'exercise'/exp OR exercis*:ti,ab OR walk*:ti,ab OR swim*:ti,ab OR flexibil*:ti,ab OR precondition*:ti,ab OR 'physiotherapy'/exp OR physiotherapy OR cycl*:ti,ab OR bicycl*:ti,ab OR train*:ti,ab OR 'physical activity'/exp OR 'physical activity' OR fitness OR 'cognition'/exp OR cognition:ti,ab OR 'cognitive behavioral therapy'/exp OR 'cognitive behavioral therapy' OR Cognitive:ti,ab OR Counseling:ti,ab OR Counsel*:ti,ab)) OR 'preoperative care') AND ('practice guideline'/exp OR 'practice guideline' OR [cochrane review]/lim OR [systematic review]/lim OR [meta analysis]/lim OR [clinical study]/lim OR 'clinical study'/exp) AND [2000-2021]/py) NOT ( 'case study'/exp OR 'case report' OR 'case study' OR [conference abstract]/lim OR [animals]/lim OR 'aged plant'/exp OR 'aged plant' OR 'child*':ti OR 'infant*':ti OR 'female genital tract tumor'/exp OR 'urogenital tract tumor'/exp OR (Whipple NEAR/3 disease) OR 'bladder cancer*':kw,ti,ab OR 'Endometrial cancer*':kw,ti,ab OR 'Ovarian cancer*':kw,ti,ab OR Gynecologic:kw,ti,ab OR Urologic:kw,ti,ab OR Urothelial:kw,ti,ab OR Urethra:kw,ti,ab) | 541 |
| Cochrane  KQ1  (24 Mar) | ([MH Aged, 80 and over] OR [MH Frail Elderly] OR elderly:kw,ti,ab OR (septuagenarian*):kw,ti,ab OR (octogenarian*'):kw,ti,ab OR (nonagenarian*):kw,ti,ab OR (centenarian*):kw,ti,ab OR (supercentenarian*):kw,ti,ab) AND ([MH Colorectal Surgery] OR (abdominal surgery):kw,ti,ab OR (rectal surgery):kw,ti,ab OR (abdomen:ti,ab OR (anal canal):ti,ab OR (anus):ti,ab OR (colon):ti,ab OR (rectum):ti,ab OR (bowel):ti,ab OR (Sigmoid):ti,ab OR [MH anal canal] OR [MH colon] OR [MH rectum] ) AND ([MH surgical procedures, operative] OR resection:kw,ti,ab OR surgery:kw,ti,ab OR surgical:kw,ti,ab)) OR (colorectal surgery):kw,ti,ab) AND ([MH Preoperative Exercise] OR (((preop*):ti,ab OR (pre-op):ti,ab OR (Prehabilit*):kw,ti,ab OR (Pre-habilit*):kw,ti,ab) AND ([MH diet] OR (diet):ti,ab OR [MH nutrition therapy] OR (nutrition):ti,ab OR (healthy eating):ti,ab OR [MH exercise] OR (exercis*):ti,ab OR (walk*):ti,ab OR (swim*):ti,ab OR (flexibil*):ti,ab OR (precondition*):ti,ab OR (physiotherapy):ti,ab OR (cycl*):ti,ab OR (bicycl*):ti,ab OR (train*):ti,ab OR (physical activity):ti,ab OR (fitness):ti,ab OR [MH cognition} OR (cognition):ti,ab OR [MH Cognitive behavioral therapy] OR (Cognitive):ti,ab OR [MH counseling] OR (Counsel*):ti,ab)) OR [MH Preoperative Care]) NOT ([MH Endometrial Neoplasms] OR [MH Ovarian Neoplasms] OR [MH Urinary Bladder Neoplasms] OR [MH Urogenital Neoplasms"[mh] OR "Whipple disease] OR Gynecologic:kw,ti,ab OR Urologic:kw,ti,ab OR Urothelial:kw,ti,ab OR Urethra:kw,ti,ab); 1/1/2000-12/31/2021 | 244 |
| ClinTrials  (31 Mar) | ("Colorectal Surgery" OR "Abdominal Surgery" OR "Rectal Surgery") AND (prehabilitation OR preoperative) \| Older Adult | 43 |
| **KQ1**  **SEARCH RESULTS** | Total items identified by database searches | 1,957 |
|  | Total trials identified in searches | 70 |
|  | Total KQ1 duplicates removed during Topic 2 screening | 1,825 |
|  | Total KQ1 records identified during Topic 2 screening | 62 |
|  | Hand-searched items located after Topic 2 screening | 8 |
|  | **Total items screened** | **70** |

### KQ2

| **KQ2: Should prehabilitation (vs. no prehabilitation) programme be used in elderly patients undergoing Upper Gastrointestinal (UGI) surgery?** | | | |
| --- | --- | --- | --- |
| **Search Concepts** | | **Elderly + UGI + Prehibilitation + Limits** | |
| **Database** | **Final search strategies** | | **Results** |
| PubMed  KQ2  (1 April) | (("Aged, 80 and over"[mh] OR "Frail Elderly"[mh] OR elderly[tiab] OR "older adult" [tw] OR septuagenar*[tw] OR octogenar*[tw] OR nonagenar*[tw] OR centenarian*[tw] OR supercentenar*[tw]) AND ((("Upper Gastrointestinal Tract" [mh] OR esophagus[mh] OR esophagus[tiab] OR stomach[mh] OR stomach[tiab] OR duodenum[mh] OR duodenum[tiab] OR Spleen[mh] OR Spleen[tiab]) AND ("surgical procedures, operative"[mh] OR "general surgery"[mh] OR surgery[sh] OR resection[tiab] OR surg*[tiab])) OR "Bariatric Surgery"[mh] OR Fundoplication[mh] OR Fundoplication[tiab] OR Gastrectomy[mh] OR Gastrect*[tiab] OR Oesophagect*[tiab] OR Splenectomy[mh] OR Splenect*[tiab] OR Esophagectomy[mh] OR Esophagect*[tiab] OR ((UGI[tiab] OR "Upper GI*" OR "gastro-esophageal"[tiab] OR "Upper Gastrointest*"[tiab]) AND Surger*[tiab])) AND ("Preoperative Exercise"[mh] OR ((preop*[tw] OR "pre-op"[tw] OR Prehabilit*[tw] OR Pre-habilit*[tw]) AND (diet[mh] OR diet[tiab] OR "Nutrition Therapy"[mh] OR nutrition[tiab] OR (healthy[tiab] OR eating[tiab]) OR Exercise[mh] OR Exercis*[tiab] OR walk*[tiab] OR swim*[tiab] OR flexibil*[tiab] OR precondition*[tiab] OR physiotherapy [tiab] OR fitness [tiab] OR "physical activity"[tiab] OR cycl*[tiab] OR bicycl*[tiab] OR train*[tiab] OR Cognition[mh] OR "Cognitive Behavioral Therapy"[mh] OR Cognitive[tiab] OR Counseling[mh] OR Counsel*[tiab] OR Counsel*[ot])) OR "Preoperative Care"[mh]) AND ("Clinical Study"[pt] OR "Comparative Study"[pt] OR "Epidemiologic studies" [mh] OR "Evaluation Study"[pt] OR "Meta-Analysis"[pt] OR "Multicenter Study"[pt] OR "Systematic Review"[pt] OR "Validation Study" [pt] OR randomized[tiab] OR analysis[tiab] OR study[tiab] OR studies [tiab] OR "Practice Guideline"[pt] OR "practice guidelines as topic"[mh] OR guideline[title] OR guidelines[title])) NOT ("Case Reports"[pt] OR "case report*"[tiab] OR "case stud*"[tiab] OR ("animals"[MH:noexp] NOT "humans"[MH]) OR rat[tiab] OR rats[tiab] OR mouse[tiab] OR mice[tiab] OR dog[tiab] OR dogs[tiab] OR porcine[tiab] OR infant[mh] OR (child[mh] NOT adult[mh]) OR children[title] OR child[title] OR infant[title] OR infants[title] OR "Endometrial Neoplasms"[mh] NOT "Esophageal Neoplasms"[mh] OR "Ovarian Neoplasms"[mh] OR "Urinary Bladder Neoplasms"[mh] OR "Urogenital Neoplasms"[mh] OR "Whipple disease"[mh] OR "Whipple's disease"[tw] OR "Whipple disease"[tw] OR "Bladder cancer*"[tw] OR "Endometrial cancer*"[tw] OR "Ovarian cancer*"[tw] OR "Renal cancer*"[tw] OR Gynecologic[tiab] OR Urologic[tiab] OR Urothelial[tiab] OR Urethra[tiab] OR ("1905/01/01"[pdat]:"1999/12/31"[pdat])) | | 306 |
| Embase  KQ2  (22 Mar) | (('very elderly' OR 'frail elderly' OR elderly:kw,ti,ab OR 'septuagenarian*':kw,ti,ab OR 'octogenarian*':kw,ti,ab OR 'nonagenarian*':kw,ti,ab OR 'centenarian*':kw,ti,ab OR 'supercentenarian*':kw,ti,ab) AND ('Splenectomy'/exp OR (('upper gastrointestinal tract'/exp OR 'upper intestine*':kw,ti,ab OR 'esophagus'/exp OR esophagus:kw,ti,ab OR 'spleen'/exp OR spleen:kw,ti,ab OR 'stomach'/exp OR stomach:kw,ti,ab OR 'duodenum'/exp OR duodenum:kw,ti,ab OR 'upper gi*':kw,ti,ab OR ugi:ti,ab) AND (resection:kw,ti,ab OR 'surgery'/exp OR surgery:kw,ti,ab OR surgical:kw,ti,ab)) OR 'stomach fundoplication'/exp OR fundoplication:kw,ti,ab OR 'gastrectomy'/exp OR gastrectomy:kw,ti,ab OR 'gastrect*':kw,ti,ab OR oesophagectomy OR 'esophagus resection'/exp OR 'esophagus resection':kw,ti,ab OR esophagectomy:kw,ti,ab OR 'Splenect*':kw,ti,ab) AND ('preoperative care'/exp OR 'preoperative exercise'/exp OR 'preoperative exercise' OR (('preop*':ti,ab OR 'pre op':ti,ab OR 'Prehabilit*':kw,ti,ab OR 'Pre-habilit*':kw,ti,ab ) AND ('diet'/exp OR diet:ti,ab OR 'diet therapy'/exp OR 'diet therapy' OR 'nutrition'/exp OR nutrition:ti,ab OR 'healthy eating'/exp OR 'healthy eating' OR 'exercise'/exp OR exercis*:ti,ab OR walk*:ti,ab OR swim*:ti,ab OR flexibil*:ti,ab OR precondition*:ti,ab OR 'physiotherapy'/exp OR physiotherapy OR cycl*:ti,ab OR bicycl*:ti,ab OR train*:ti,ab OR 'physical activity'/exp OR 'physical activity' OR fitness OR 'cognition'/exp OR cognition:ti,ab OR 'cognitive behavioral therapy'/exp OR 'cognitive behavioral therapy' OR Cognitive:ti,ab OR Counseling:ti,ab OR Counsel*:ti,ab)) OR 'preoperative care') AND ('practice guideline'/exp OR 'practice guideline' OR [cochrane review]/lim OR [systematic review]/lim OR [meta analysis]/lim OR [clinical study]/lim OR 'clinical study'/exp) AND [2000-2021]/py) NOT ( 'case study'/exp OR 'case report' OR 'case study' OR [conference abstract]/lim OR [animals]/lim OR 'aged plant'/exp OR 'aged plant' OR 'child*':ti OR 'infant*':ti OR 'female genital tract tumor'/exp OR 'urogenital tract tumor'/exp OR 'esophagus tumor'/exp OR (Whipple NEAR/3 disease) OR 'bladder cancer*':kw,ti,ab OR 'Endometrial cancer*':kw,ti,ab OR 'Ovarian cancer*':kw,ti,ab OR Gynecologic:kw,ti,ab OR Urologic:kw,ti,ab OR Urothelial:kw,ti,ab OR Urethra:kw,ti,ab) | | 114 |
| Cochrane  KQ2  (24 Mar) | ([MH Aged, 80 and over] OR [MH Frail Elderly] OR elderly:kw,ti,ab OR (septuagenarian*):kw,ti,ab OR (octogenarian*'):kw,ti,ab OR (nonagenarian*):kw,ti,ab OR (centenarian*):kw,ti,ab OR (supercentenarian*):kw,ti,ab) AND ([MH Splenectomy] OR (([MH upper gastrointestinal tract] OR (upper intestine*):kw,ti,ab OR OR [MH esophagus] OR [MH stomach] OR [MH duodenum] OR [MH spleen] OR esophagus:ti,ab OR OR stomach:ti,ab OR duodenum:ti,ab OR spleen:ti,ab OR 'upper gi*':kw,ti,ab OR ugi:kw,ti,ab OR (gastro-esophageal):ti,ab) AND (resection:kw,ti,ab OR [MH surgical procedures, operative] OR surgery:kw,ti,ab OR surgical:kw,ti,ab)) OR [MH Fundoplication] OR fundoplication:kw,ti,ab OR [MH Gastrectomy] OR gastrectomy:kw,ti,ab OR (gastrect*):kw,ti,ab OR oesophagectomy OR (Splenect*):kw,ti,ab) AND ([MH Preoperative Exercise] OR (((preop*):ti,ab OR (pre-op):ti,ab OR (Prehabilit*):kw,ti,ab OR (Pre-habilit*):kw,ti,ab) AND ([MH diet] OR (diet):ti,ab OR [MH nutrition therapy] OR (nutrition):ti,ab OR (healthy eating):ti,ab OR [MH exercise] OR (exercis*):ti,ab OR (walk*):ti,ab OR (swim*):ti,ab OR (flexibil*):ti,ab OR (precondition*):ti,ab OR (physiotherapy):ti,ab OR (cycl*):ti,ab OR (bicycl*):ti,ab OR (train*):ti,ab OR (physical activity):ti,ab OR (fitness):ti,ab OR [MH cognition} OR (cognition):ti,ab OR [MH Cognitive behavioral therapy] OR (Cognitive):ti,ab OR [MH counseling] OR (Counsel*):ti,ab)) OR [MH Preoperative Care]) NOT ([MH Endometrial Neoplasms] OR [MH Ovarian Neoplasms] OR [MH Urinary Bladder Neoplasms] OR [MH Urogenital Neoplasms"[mh] OR "Whipple disease] OR Gynecologic:kw,ti,ab OR Urologic:kw,ti,ab OR Urothelial:kw,ti,ab OR Urethra:kw,ti,ab); 1/1/2000-12/31/2021 | | 37 |
| ClinTrials KQ2  (31 Mar) | ("Bariatric Surgery" OR Fundoplication OR Splenectomy OR pancreatectomy OR Gastrectomy OR (("Upper Gastrointestinal" OR "upper gI") AND surgery)) AND (prehabilitation OR preoperative) \| Older Adult | | 41 |
| **KQ2**  **SEARCH RESULTS** | Total items identified by database searches | | 498 |
|  | Total trials identified in searches | | 46 |
|  | Total KQ2 duplicates removed during Topic 2 screening | | 430 |
|  | Total KQ2 records identified during Topic 2 screening | | 22 |
|  | Hand-searched items located after Topic 2 screening | | 2 |
|  | **Total items screened** | | **24** |

### KQ3

| **KQ3: should prehabilitation (vs. no prehabilitation) programme be used in elderly patients undergoing hepatobiliary pancreatic (HPB) surgery?** | | | |
| --- | --- | --- | --- |
| **Search Concepts** | | **Elderly + HPB + Prehibilitation + Limits** | |
| **Database** | **Final search strategies** | | **Results** |
| PubMed  KQ3  (22 Mar) | (("Aged, 80 and over"[mh] OR "Frail Elderly"[mh] OR elderly[tiab] OR "older adult" [tw] OR septuagenar*[tw] OR octogenar*[tw] OR nonagenar*[tw] OR centenarian*[tw] OR supercentenar*[tw]) AND "Hepato-pancreato-biliary"[tiab] OR HPB[tiab] OR hepatopancreaticobiliary[tiab] OR Whipple[tiab] OR Whipple [ot] OR ((liver[mh] OR liver[tiab] OR pancreas [mh] OR pancreas[tiab] OR gallbladder [mh] OR gallbladder [tiab] OR "bile ducts"[mh] OR "bile Duct*"[tiab] OR jejunum[mh] OR jejunum[tiab]) AND ("surgical procedures, operative"[mh] OR "general surgery"[mh] OR surgery[sh] OR surg*[tiab] OR resection[tiab])) OR Hepatectomy[mh] OR Hepatect*[tiab] OR pancreatectomy[mh] OR pancreatect*[tiab] OR Hepatojejunost*[tiab] OR ((Renal[tiab] OR biliary[tiab]) AND surger*[tiab])) AND "Preoperative Exercise"[mh] OR ((preop*[tw] OR "pre-op"[tw] OR Prehabilit*[tw] OR Pre-habilit*[tw]) AND (diet[mh] OR diet[tiab] OR "Nutrition Therapy"[mh] OR nutrition[tiab] OR (healthy[tiab] OR eating[tiab]) OR Exercise[mh] OR Exercis*[tiab] OR walk*[tiab] OR swim*[tiab] OR flexibil*[tiab] OR precondition*[tiab] OR physiotherapy [tiab] OR fitness [tiab] OR "physical activity"[tiab] OR cycl*[tiab] OR bicycl*[tiab] OR train*[tiab] OR Cognition[mh] OR "Cognitive Behavioral Therapy"[mh] OR Cognitive[tiab] OR Counseling[mh] OR Counsel*[tiab] OR Counsel*[ot])) OR "Preoperative Care"[mh]) AND ("Clinical Study"[pt] OR "Comparative Study"[pt] OR "Epidemiologic studies" [mh] OR "Evaluation Study"[pt] OR "Meta-Analysis"[pt] OR "Multicenter Study"[pt] OR "Systematic Review"[pt] OR "Validation Study" [pt] OR randomized[tiab] OR analysis[tiab] OR study[tiab] OR studies [tiab] OR "Practice Guideline"[pt] OR "practice guidelines as topic"[mh] OR guideline[title] OR guidelines[title])) NOT ("Case Reports"[pt] OR "case report*"[tiab] OR "case stud*"[tiab] OR ("animals"[MH:noexp] NOT "humans"[MH]) OR rat[tiab] OR rats[tiab] OR mouse[tiab] OR mice[tiab] OR dog[tiab] OR dogs[tiab] OR porcine[tiab] OR infant[mh] OR (child[mh] NOT adult[mh]) OR children[title] OR child[title] OR infant[title] OR infants[title] OR "Endometrial Neoplasms"[mh] OR "Ovarian Neoplasms"[mh] OR "Urinary Bladder Neoplasms"[mh] OR "Urogenital Neoplasms"[mh] OR "Whipple disease"[mh] OR "Whipple's disease"[tw] OR "Whipple disease"[tw] OR "Bladder cancer*"[tw] OR "Endometrial cancer*"[tw] OR "Ovarian cancer*"[tw] OR "Renal cancer*"[tw] OR Gynecologic[tiab] OR Urologic[tiab] OR Urothelial[tiab] OR Urethra[tiab] OR ("1905/01/01"[pdat]:"1999/12/31"[pdat])) | | 723 |
| Embase  KQ3  (22 Mar) | (('very elderly' OR 'frail elderly' OR elderly:kw,ti,ab OR 'septuagenarian*':kw,ti,ab OR 'octogenarian*':kw,ti,ab OR 'nonagenarian*':kw,ti,ab OR 'centenarian*':kw,ti,ab OR 'supercentenarian*':kw,ti,ab) AND ('biliary surgery' OR (('liver'/exp OR liver:kw,ti,ab OR 'pancreas'/exp OR pancreas:kw,ti,ab OR 'gallbladder'/exp OR gallbladder:kw,ti,ab OR 'bile duct'/exp OR 'bile duct' OR 'bile ducts' OR 'jejunum'/exp OR jejunum:kw,ti,ab) AND (resection:kw,ti,ab OR 'surgery'/exp OR surgery:kw,ti,ab OR surgical:kw,ti,ab)) OR 'liver surgery'/exp OR 'liver surgery' OR 'liver resection'/exp OR 'liver resection' OR 'pancreatectomy'/exp OR pancreatectomy OR 'hepatect*':kw,ti,ab OR 'nephrect*':kw,ti,ab OR 'hepatojejunostomy'/exp OR hepatojejunostomy:kw,ti,ab OR 'hepatopancreaticobiliary surgery' OR 'hepato pancreato biliary' OR hepatopancreaticobiliary OR HPB:kw,ti,ab OR 'biliary tract surgery'/exp OR 'biliary tract surgery' OR 'Renal surgery') AND ('preoperative care'/exp OR 'preoperative exercise'/exp OR 'preoperative exercise' OR (('preop*':ti,ab OR 'pre op':ti,ab OR 'Prehabilit*':kw,ti,ab OR 'Pre-habilit*':kw,ti,ab ) AND ('diet'/exp OR diet:ti,ab OR 'diet therapy'/exp OR 'diet therapy' OR 'nutrition'/exp OR nutrition:ti,ab OR 'healthy eating'/exp OR 'healthy eating' OR 'exercise'/exp OR exercis*:ti,ab OR walk*:ti,ab OR swim*:ti,ab OR flexibil*:ti,ab OR precondition*:ti,ab OR 'physiotherapy'/exp OR physiotherapy OR cycl*:ti,ab OR bicycl*:ti,ab OR train*:ti,ab OR 'physical activity'/exp OR 'physical activity' OR fitness OR 'cognition'/exp OR cognition:ti,ab OR 'cognitive behavioral therapy'/exp OR 'cognitive behavioral therapy' OR Cognitive:ti,ab OR Counseling:ti,ab OR Counsel*:ti,ab)) OR 'preoperative care') AND ('practice guideline'/exp OR 'practice guideline' OR [cochrane review]/lim OR [systematic review]/lim OR [meta analysis]/lim OR [clinical study]/lim OR 'clinical study'/exp) AND [2000-2021]/py) NOT ( 'case study'/exp OR 'case report' OR 'case study' OR [conference abstract]/lim OR [animals]/lim OR 'aged plant'/exp OR 'aged plant' OR 'child*':ti OR 'infant*':ti OR 'female genital tract tumor'/exp OR 'urogenital tract tumor'/exp OR (Whipple NEAR/3 disease) OR 'bladder cancer*':kw,ti,ab OR 'Endometrial cancer*':kw,ti,ab OR 'Ovarian cancer*':kw,ti,ab OR Gynecologic:kw,ti,ab OR Urologic:kw,ti,ab OR Urothelial:kw,ti,ab OR Urethra:kw,ti,ab) | | 172 |
| Cochrane  KQ3  (24 Mar) | ([MH Aged, 80 and over] OR [MH Frail Elderly] OR elderly:kw,ti,ab OR (septuagenarian*):kw,ti,ab OR (octogenarian*'):kw,ti,ab OR (nonagenarian*):kw,ti,ab OR (centenarian*):kw,ti,ab OR (supercentenarian*):kw,ti,ab) AND ([MH Hepatectomy] OR [MH pancreatectomy] OR (pancreatect*):ti,ab OR (Hepatojejunost*):ti,ab OR (Renal surger*):ti,ab OR (biliary surger*):ti,ab OR (Hepato-pancreato-biliary):ti,ab OR (HPB):ti,ab OR hepatopancreaticobiliary:ti,ab OR (([MH liver] OR (liver):ti,ab OR [MH pancreas] OR (pancreas):ti,ab OR [MH gallbladder] OR (gallbladder):ti,ab OR [MH bile ducts] OR (bile Duct*):ti,ab OR [MH kidney] OR (kidney*):ti,ab OR [MH jejunum] OR (jejunum):ti,ab) AND ([MH surgical procedures, operative] OR (resection):ti,ab,kw OR (surger*):ti,ab,kw)) OR (Hepatect*):ti,ab) AND ([MH Preoperative Exercise] OR (((preop*):ti,ab OR (pre-op):ti,ab OR (Prehabilit*):kw,ti,ab OR (Pre-habilit*):kw,ti,ab) AND ([MH diet] OR (diet):ti,ab OR [MH nutrition therapy] OR (nutrition):ti,ab OR (healthy eating):ti,ab OR [MH exercise] OR (exercis*):ti,ab OR (walk*):ti,ab OR (swim*):ti,ab OR (flexibil*):ti,ab OR (precondition*):ti,ab OR (physiotherapy):ti,ab OR (cycl*):ti,ab OR (bicycl*):ti,ab OR (train*):ti,ab OR (physical activity):ti,ab OR (fitness):ti,ab OR [MH cognition} OR (cognition):ti,ab OR [MH Cognitive behavioral therapy] OR (Cognitive):ti,ab OR [MH counseling] OR (Counsel*):ti,ab)) OR [MH Preoperative Care]) NOT ([MH Endometrial Neoplasms] OR [MH Ovarian Neoplasms] OR [MH Urinary Bladder Neoplasms] OR [MH Urogenital Neoplasms"[mh] OR "Whipple disease] OR Gynecologic:kw,ti,ab OR Urologic:kw,ti,ab OR Urothelial:kw,ti,ab OR Urethra:kw,ti,ab); 1/1/2000-12/31/2021 | | 71 |
| ClinTrials  (31 Mar) | ("Hepato-pancreato-biliary" OR hepatopancreaticobiliary OR Hepatectomy OR pancreatectomy OR Whipple) AND (prehabilitation OR preoperative) \| Older Adult | | 17 |
| **KQ3**  **SEARCH RESULTS** | Total items identified by database searches | | 983 |
|  | Total trials identified in searches | | 36 |
|  | Total KQ3 duplicates removed during Topic 2 screening | | 922 |
|  | Total KQ3 records identified during Topic 2 screening | | 25 |
|  | Hand-searched items located after Topic 2 screening | | 3 |
|  | **Total items screened** | | **28** |

### KQ4

| **Q4: Should prehabilitation (vs. no prehabilitation) programme be used in elderly patients undergoing hernia surgery?** | | | |
| --- | --- | --- | --- |
| **Search Concepts** | | **Elderly + Hernia + Prehibilitation + Limits** | |
| **Database** | **Final search strategies** | | **Results** |
| PubMed  KQ4  (22 Mar) | (("Aged, 80 and over"[mh] OR "Frail Elderly"[mh] OR elderly[tiab] OR "older adult" [tw] OR septuagenar*[tw] OR octogenar*[tw] OR nonagenar*[tw] OR centenarian*[tw] OR supercentenar*[tw]) AND (((Hernia[tiab] OR Hernia[mh]) AND ("surgical procedures, operative"[mh] OR "general surgery"[mh] OR surgery[sh] OR surg*[tiab])) OR Herniorrhaphy[mh] OR Herniorrhap*[tiab]) AND ("Preoperative Exercise"[mh] OR ((preop*[tw] OR "pre-op"[tw] OR Prehabilit*[tw] OR Pre-habilit*[tw]) AND (diet[mh] OR diet[tiab] OR "Nutrition Therapy"[mh] OR nutrition[tiab] OR (healthy[tiab] OR eating[tiab]) OR Exercise[mh] OR Exercis*[tiab] OR walk*[tiab] OR swim*[tiab] OR flexibil*[tiab] OR precondition*[tiab] OR physiotherapy [tiab] OR fitness [tiab] OR "physical activity"[tiab] OR cycl*[tiab] OR bicycl*[tiab] OR train*[tiab] OR Cognition[mh] OR "Cognitive Behavioral Therapy"[mh] OR Cognitive[tiab] OR Counseling[mh] OR Counsel*[tiab] OR Counsel*[ot])) OR "Preoperative Care"[mh]) AND ("Clinical Study"[pt] OR "Comparative Study"[pt] OR "Epidemiologic studies" [mh] OR "Evaluation Study"[pt] OR "Meta-Analysis"[pt] OR "Multicenter Study"[pt] OR "Systematic Review"[pt] OR "Validation Study" [pt] OR randomized[tiab] OR analysis[tiab] OR study[tiab] OR studies [tiab] OR "Practice Guideline"[pt] OR "practice guidelines as topic"[mh] OR guideline[title] OR guidelines[title])) NOT ("Case Reports"[pt] OR "case report*"[tiab] OR "case stud*"[tiab] OR ("animals"[MH:noexp] NOT "humans"[MH]) OR rat[tiab] OR rats[tiab] OR mouse[tiab] OR mice[tiab] OR dog[tiab] OR dogs[tiab] OR porcine[tiab] OR infant[mh] OR (child[mh] NOT adult[mh]) OR children[title] OR child[title] OR infant[title] OR infants[title] OR "Endometrial Neoplasms"[mh] OR "Ovarian Neoplasms"[mh] OR "Urinary Bladder Neoplasms"[mh] OR "Urogenital Neoplasms"[mh] OR "Whipple disease"[mh] OR "Whipple's disease"[tw] OR "Whipple disease"[tw] OR "Bladder cancer*"[tw] OR "Endometrial cancer*"[tw] OR "Ovarian cancer*"[tw] OR "Renal cancer*"[tw] OR Gynecologic[tiab] OR Urologic[tiab] OR Urothelial[tiab] OR Urethra[tiab] OR ("1905/01/01"[pdat]:"1999/12/31"[pdat])) | | 100 |
| Embase  KQ4  (22 Mar) | (('very elderly' OR 'frail elderly' OR elderly:kw,ti,ab OR 'septuagenarian*':kw,ti,ab OR 'octogenarian*':kw,ti,ab OR 'nonagenarian*':kw,ti,ab OR 'centenarian*':kw,ti,ab OR 'supercentenarian*':kw,ti,ab) AND ('hernia surgery'/exp OR 'herniorrhaphy'/exp OR 'herniorrhap*':kw,ti,ab OR (('hernia'/exp OR 'hernia':kw,ti,ab) AND ('surgery'/exp OR surgery:kw,ti,ab OR surgical:kw,ti,ab)) OR 'hernia surgery') AND ('preoperative care'/exp OR 'preoperative exercise'/exp OR 'preoperative exercise' OR (('preop*':ti,ab OR 'pre op':ti,ab OR 'Prehabilit*':kw,ti,ab OR 'Pre-habilit*':kw,ti,ab ) AND ('diet'/exp OR diet:ti,ab OR 'diet therapy'/exp OR 'diet therapy' OR 'nutrition'/exp OR nutrition:ti,ab OR 'healthy eating'/exp OR 'healthy eating' OR 'exercise'/exp OR exercis*:ti,ab OR walk*:ti,ab OR swim*:ti,ab OR flexibil*:ti,ab OR precondition*:ti,ab OR 'physiotherapy'/exp OR physiotherapy OR cycl*:ti,ab OR bicycl*:ti,ab OR train*:ti,ab OR 'physical activity'/exp OR 'physical activity' OR fitness OR 'cognition'/exp OR cognition:ti,ab OR 'cognitive behavioral therapy'/exp OR 'cognitive behavioral therapy' OR Cognitive:ti,ab OR Counseling:ti,ab OR Counsel*:ti,ab)) OR 'preoperative care') AND ('practice guideline'/exp OR 'practice guideline' OR [cochrane review]/lim OR [systematic review]/lim OR [meta analysis]/lim OR [clinical study]/lim OR 'clinical study'/exp) AND [2000-2021]/py) NOT ( 'case study'/exp OR 'case report' OR 'case study' OR [conference abstract]/lim OR [animals]/lim OR 'aged plant'/exp OR 'aged plant' OR 'child*':ti OR 'infant*':ti OR 'female genital tract tumor'/exp OR 'urogenital tract tumor'/exp OR (Whipple NEAR/3 disease) OR 'bladder cancer*':kw,ti,ab OR 'Endometrial cancer*':kw,ti,ab OR 'Ovarian cancer*':kw,ti,ab OR Gynecologic:kw,ti,ab OR Urologic:kw,ti,ab OR Urothelial:kw,ti,ab OR Urethra:kw,ti,ab) | | 45 |
| Cochrane  KQ4  (24 Mar) | ([MH Aged, 80 and over] OR [MH Frail Elderly] OR elderly:kw,ti,ab OR (septuagenarian*):kw,ti,ab OR (octogenarian*'):kw,ti,ab OR (nonagenarian*):kw,ti,ab OR (centenarian*):kw,ti,ab OR (supercentenarian*):kw,ti,ab) AND ([MH Herniorrhaphy] OR (((Hernia):ti,ab,kw OR [MH Hernia]) AND ([MH surgical procedures, operative] OR surgery:kw,ti,ab OR surgical:kw,ti,ab)) OR (Herniorrhap*):ti,ab,kw) AND ([MH Preoperative Exercise] OR (((preop*):ti,ab OR (pre-op):ti,ab OR (Prehabilit*):kw,ti,ab OR (Pre-habilit*):kw,ti,ab) AND ([MH diet] OR (diet):ti,ab OR [MH nutrition therapy] OR (nutrition):ti,ab OR (healthy eating):ti,ab OR [MH exercise] OR (exercis*):ti,ab OR (walk*):ti,ab OR (swim*):ti,ab OR (flexibil*):ti,ab OR (precondition*):ti,ab OR (physiotherapy):ti,ab OR (cycl*):ti,ab OR (bicycl*):ti,ab OR (train*):ti,ab OR (physical activity):ti,ab OR (fitness):ti,ab OR [MH cognition} OR (cognition):ti,ab OR [MH Cognitive behavioral therapy] OR (Cognitive):ti,ab OR [MH counseling] OR (Counsel*):ti,ab)) OR [MH Preoperative Care]) NOT ([MH Endometrial Neoplasms] OR [MH Ovarian Neoplasms] OR [MH Urinary Bladder Neoplasms] OR [MH Urogenital Neoplasms"[mh] OR "Whipple disease] OR Gynecologic:kw,ti,ab OR Urologic:kw,ti,ab OR Urothelial:kw,ti,ab OR Urethra:kw,ti,ab) ; 1/1/2000-12/31/2021 | | 8 |
| ClinTrials  (31 Mar) | ("Herniorrhaphy" OR (Hernia AND Surgery)) AND (prehabilitation OR preoperative) \| Older Adult | | 15 |
| **KQ4**  **SEARCH RESULTS** | Total items identified by database searches | | 168 |
|  | Total trials identified in searches | | 17 |
|  | Total KQ4 duplicates removed during Topic 2 screening | | 137 |
|  | Total KQ4 records identified during Topic 2 screening | | 14 |
|  | Hand-searched items located after Topic 2 screening | | 1 |
|  | **Total items screened** | | **15** |

### KQ5

| **Q5: Should perioperative optimisation (vs. no optimisation) of anaemia be used elderly patients undergoing colorectal surgery?** | | | |
| --- | --- | --- | --- |
| **Search Concepts** | | **Elderly + Colorectal + Anemia + Limits** | |
| **Database** | **Final search strategies** | | **Results** |
| PubMed  KQ5  (22 Mar) | (("Aged, 80 and over"[mh] OR "Frail Elderly"[mh] OR elderly[tiab] OR "older adult" [tw] OR septuagenar*[tw] OR octogenar*[tw] OR nonagenar*[tw] OR centenarian*[tw] OR supercentenar*[tw]) AND ("Colorectal Surgery"[mh] OR ((abdomen[tiab] OR "anal canal"[tiab] OR "anal canal"[mh] OR anus[tiab] OR bowel[tiab] OR colon[tiab] OR colon [mh] OR rectum[tiab] OR rectum[mh] OR "Colon, Sigmoid"[mh] OR Sigmoid[tiab]) AND ("surgical procedures, operative"[mh] OR "general surgery"[mh] OR surgery[sh] OR resection[tiab] OR surg*[tiab])) OR ((rectal [tiab] OR Colorectal[tiab] OR abdominal[tiab]) AND Surger*[tiab])) AND ((Anemia[mh] OR Anemia[tiab] OR Anaemia[tiab]) AND ("Preoperative Care"[mh] OR preop*[tw] OR "pre-op"[tw] OR Prehabilit*[tw] OR Pre-habilit*[tw])) AND ("Clinical Study"[pt] OR "Comparative Study"[pt] OR "Epidemiologic studies" [mh] OR "Evaluation Study"[pt] OR "Meta-Analysis"[pt] OR "Multicenter Study"[pt] OR "Systematic Review"[pt] OR "Validation Study" [pt] OR randomized[tiab] OR analysis[tiab] OR study[tiab] OR studies [tiab] OR "Practice Guideline"[pt] OR "practice guidelines as topic"[mh] OR guideline[title] OR guidelines[title])) NOT (("animals"[MH:noexp] NOT "humans"[MH]) OR rat[tiab] OR rats[tiab] OR mouse[tiab] OR mice[tiab] OR dog[tiab] OR dogs[tiab] OR porcine[tiab] OR infant[mh] OR (child[mh] NOT adult[mh]) OR children[title] OR child[title] OR infant[title] OR infants[title] OR "Endometrial Neoplasms"[mh] OR "Ovarian Neoplasms"[mh] OR "Urinary Bladder Neoplasms"[mh] OR "Urogenital Neoplasms"[mh] OR "Whipple disease"[mh] OR "Whipple's disease"[tw] OR "Whipple disease"[tw] OR "Bladder cancer*"[tw] OR "Endometrial cancer*"[tw] OR "Ovarian cancer*"[tw] OR "Renal cancer*"[tw] OR Gynecologic[tiab] OR Urologic[tiab] OR Urothelial[tiab] OR Urethra[tiab] OR ("1905/01/01"[pdat]:"1999/12/31"[pdat])) | | 72 |
| Embase  KQ5  (22 Mar) | (('very elderly' OR 'frail elderly' OR elderly:kw,ti,ab OR 'septuagenarian*':kw,ti,ab OR 'octogenarian*':kw,ti,ab OR 'nonagenarian*':kw,ti,ab OR 'centenarian*':kw,ti,ab OR 'supercentenarian*':kw,ti,ab) AND ('colorectal surgery'/exp OR 'abdominal surgery'/exp OR 'abdominal surgery' OR 'rectum surgery'/exp OR 'rectum surgery' OR (('abdomen'/exp OR abdomen OR 'anal canal'/exp OR 'anal canal' OR 'anus'/exp OR anus OR 'colon'/exp OR colon OR 'rectum'/exp OR rectum OR 'intestine'/exp OR intestine:ti,ab OR 'sigmoid'/exp OR sigmoid) AND (resection:kw,ti,ab OR 'surgery'/exp OR surgery:kw,ti,ab OR surgical:kw,ti,ab)) OR 'colorectal surgery') AND (('preop*':ti,ab OR 'pre op':ti,ab OR 'Prehabilit*':kw,ti,ab OR 'Pre-habilit*':kw,ti,ab OR 'preoperative care'/exp ) AND ('anemia'/exp OR anemia:ti,ab OR Anaemia:ti,ab)) AND ('practice guideline'/exp OR 'practice guideline' OR [cochrane review]/lim OR [systematic review]/lim OR [meta analysis]/lim OR [clinical study]/lim OR 'clinical study'/exp OR 'case report' OR 'case study') AND [2000-2021]/py) NOT ([conference abstract]/lim OR [animals]/lim OR 'aged plant'/exp OR 'aged plant' OR 'child*':ti OR 'infant*':ti OR 'female genital tract tumor'/exp OR 'urogenital tract tumor'/exp OR (Whipple NEAR/3 disease) OR 'bladder cancer*':kw,ti,ab OR 'Endometrial cancer*':kw,ti,ab OR 'Ovarian cancer*':kw,ti,ab OR Gynecologic:kw,ti,ab OR Urologic:kw,ti,ab OR Urothelial:kw,ti,ab OR Urethra:kw,ti,ab) | | 85 |
| Cochrane  KQ5  (24 Mar) | ([MH Aged, 80 and over] OR [MH Frail Elderly] OR elderly:kw,ti,ab OR (septuagenarian*):kw,ti,ab OR (octogenarian*'):kw,ti,ab OR (nonagenarian*):kw,ti,ab OR (centenarian*):kw,ti,ab OR (supercentenarian*):kw,ti,ab) AND ([MH Colorectal Surgery] OR (abdominal surgery):kw,ti,ab OR (rectal surgery):kw,ti,ab OR (abdomen:ti,ab OR (anal canal):ti,ab OR (anus):ti,ab OR (colon):ti,ab OR (rectum):ti,ab OR (bowel):ti,ab OR (Sigmoid):ti,ab OR [MH anal canal] OR [MH colon] OR [MH rectum] ) AND ([MH surgical procedures, operative] OR resection:kw,ti,ab OR surgery:kw,ti,ab OR surgical:kw,ti,ab)) OR (colorectal surgery):kw,ti,ab) AND ([MH Anemia] OR anemia:ti,ab OR Anaemia:ti,ab) AND ((preop*):ti,ab OR (pre op):ti,ab OR (Prehabilit*):kw,ti,ab OR (Pre-habilit*):kw,ti,ab OR [MH Preoperative care]) NOT ([MH Endometrial Neoplasms] OR [MH Ovarian Neoplasms] OR [MH Urinary Bladder Neoplasms] OR [MH Urogenital Neoplasms"[mh] OR "Whipple disease] OR Gynecologic:kw,ti,ab OR Urologic:kw,ti,ab OR Urothelial:kw,ti,ab OR Urethra:kw,ti,ab) ; 1/1/2000-12/31/2021 | | 12 |
| ClinTrials  (31 Mar) | ("Colorectal Surgery" OR "Abdominal Surgery" OR "Rectal Surgery") AND (prehabilitation OR preoperative) AND (Anemia OR anaemia) \| Older Adult | | 0 |
| **KQ5**  **SEARCH RESULTS** | Total items identified by database searches | | 169 |
|  | Total trials identified in searches | | 9 |
|  | Total KQ5 duplicates removed during Topic 2 screening | | 146 |
|  | Total KQ5 records identified during Topic 2 screening | | 14 |
|  | Hand-searched items located after Topic 2 screening | | 0 |
|  | **Total items screened** | | **14** |

### KQ6

| **Q6: Should perioperative optimisation (vs. no optimisation) of anaemia be used elderly patients undergoing UGI surgery?** | | | |
| --- | --- | --- | --- |
| **Search Concepts** | | **Elderly + UGI + Anemia + Limits** | |
| **Database** | **Final search strategies** | | **Results** |
| PubMed  KQ6  (1 April) | (("Aged, 80 and over"[mh] OR "Frail Elderly"[mh] OR elderly[tiab] OR "older adult" [tw] OR septuagenar*[tw] OR octogenar*[tw] OR nonagenar*[tw] OR centenarian*[tw] OR supercentenar*[tw]) AND ((("Upper Gastrointestinal Tract" [mh] OR esophagus[mh] OR esophagus[tiab] OR stomach[mh] OR stomach[tiab] OR duodenum[mh] OR duodenum[tiab] OR Spleen[mh] OR Spleen[tiab]) AND ("surgical procedures, operative"[mh] OR "general surgery"[mh] OR surgery[sh] OR resection[tiab] OR surg*[tiab])) OR "Bariatric Surgery"[mh] OR Esophagectomy[mh] OR Esophagect*[tiab] OR Fundoplication[mh] OR Fundoplication[tiab] OR Gastrectomy[mh] OR Gastrect*[tiab] OR Oesophagect*[tiab] OR Splenectomy[mh] OR Splenect*[tiab] OR ((UGI[tiab] OR "Upper GI*" OR "gastro-esophageal"[tiab] OR "Upper Gastrointest*"[tiab]) AND Surger*[tiab])) AND ((Anemia[mh] OR Anemia[tiab] OR Anaemia[tiab]) AND ("Preoperative Care"[mh] OR preop*[tw] OR "pre-op"[tw] OR Prehabilit*[tw] OR Pre-habilit*[tw])) AND ("Clinical Study"[pt] OR "Comparative Study"[pt] OR "Epidemiologic studies" [mh] OR "Evaluation Study"[pt] OR "Meta-Analysis"[pt] OR "Multicenter Study"[pt] OR "Systematic Review"[pt] OR "Validation Study" [pt] OR randomized[tiab] OR analysis[tiab] OR study[tiab] OR studies [tiab] OR "Practice Guideline"[pt] OR "practice guidelines as topic"[mh] OR guideline[title] OR guidelines[title])) NOT (("animals"[MH:noexp] NOT "humans"[MH]) OR rat[tiab] OR rats[tiab] OR mouse[tiab] OR mice[tiab] OR dog[tiab] OR dogs[tiab] OR porcine[tiab] OR infant[mh] OR (child[mh] NOT adult[mh]) OR children[title] OR child[title] OR infant[title] OR infants[title] OR "Endometrial Neoplasms"[mh] OR "Esophageal Neoplasms"[mh] OR "Ovarian Neoplasms"[mh] OR "Urinary Bladder Neoplasms"[mh] OR "Urogenital Neoplasms"[mh] OR "Whipple disease"[mh] OR "Whipple's disease"[tw] OR "Whipple disease"[tw] OR "Bladder cancer*"[tw] OR "Endometrial cancer*"[tw] OR "Ovarian cancer*"[tw] OR "Renal cancer*"[tw] OR Gynecologic[tiab] OR Urologic[tiab] OR Urothelial[tiab] OR Urethra[tiab] OR ("1905/01/01"[pdat]:"1999/12/31"[pdat])) | | 19 |
| Embase  KQ6  (22 Mar) | (('very elderly' OR 'frail elderly' OR elderly:kw,ti,ab OR 'septuagenarian*':kw,ti,ab OR 'octogenarian*':kw,ti,ab OR 'nonagenarian*':kw,ti,ab OR 'centenarian*':kw,ti,ab OR 'supercentenarian*':kw,ti,ab) AND ('Splenectomy'/exp OR (('upper gastrointestinal tract'/exp OR 'upper intestine*':kw,ti,ab OR 'esophagus'/exp OR esophagus:kw,ti,ab OR 'spleen'/exp OR spleen:kw,ti,ab OR 'stomach'/exp OR stomach:kw,ti,ab OR 'duodenum'/exp OR duodenum:kw,ti,ab OR 'upper gi*':kw,ti,ab OR ugi:ti,ab) AND (resection:kw,ti,ab OR 'surgery'/exp OR surgery:kw,ti,ab OR surgical:kw,ti,ab)) OR 'stomach fundoplication'/exp OR fundoplication:kw,ti,ab OR 'gastrectomy'/exp OR gastrectomy:kw,ti,ab OR 'gastrect*':kw,ti,ab OR oesophagectomy OR 'esophagus resection'/exp OR 'esophagus resection':kw,ti,ab OR esophagectomy:kw,ti,ab OR 'Splenect*':kw,ti,ab) AND (('preop*':ti,ab OR 'pre op':ti,ab OR 'Prehabilit*':kw,ti,ab OR 'Pre-habilit*':kw,ti,ab OR 'preoperative care'/exp ) AND ('anemia'/exp OR anemia:ti,ab OR Anaemia:ti,ab)) AND ('practice guideline'/exp OR 'practice guideline' OR [cochrane review]/lim OR [systematic review]/lim OR [meta analysis]/lim OR [clinical study]/lim OR 'clinical study'/exp OR 'case report' OR 'case study') AND [2000-2021]/py) NOT ([conference abstract]/lim OR [animals]/lim OR 'aged plant'/exp OR 'aged plant' OR 'child*':ti OR 'infant*':ti OR 'female genital tract tumor'/exp OR 'urogenital tract tumor'/exp OR 'esophagus tumor'/exp OR (Whipple NEAR/3 disease) OR 'bladder cancer*':kw,ti,ab OR 'Endometrial cancer*':kw,ti,ab OR 'Ovarian cancer*':kw,ti,ab OR Gynecologic:kw,ti,ab OR Urologic:kw,ti,ab OR Urothelial:kw,ti,ab OR Urethra:kw,ti,ab) | | 29 |
| Cochrane  KQ6  (24 Mar) | ([MH Aged, 80 and over] OR [MH Frail Elderly] OR elderly:kw,ti,ab OR (septuagenarian*):kw,ti,ab OR (octogenarian*'):kw,ti,ab OR (nonagenarian*):kw,ti,ab OR (centenarian*):kw,ti,ab OR (supercentenarian*):kw,ti,ab) AND ([MH Splenectomy] OR (([MH upper gastrointestinal tract] OR (upper intestine*):kw,ti,ab OR OR [MH esophagus] OR [MH stomach] OR [MH duodenum] OR [MH spleen] OR esophagus:ti,ab OR OR stomach:ti,ab OR duodenum:ti,ab OR spleen:ti,ab OR 'upper gi*':kw,ti,ab OR ugi:kw,ti,ab OR (gastro-esophageal):ti,ab) AND (resection:kw,ti,ab OR [MH surgical procedures, operative] OR surgery:kw,ti,ab OR surgical:kw,ti,ab)) OR [MH Fundoplication] OR fundoplication:kw,ti,ab OR [MH Gastrectomy] OR gastrectomy:kw,ti,ab OR (gastrect*):kw,ti,ab OR oesophagectomy OR (Splenect*):kw,ti,ab) AND ([MH Anemia] OR anemia:ti,ab OR Anaemia:ti,ab) AND ((preop*):ti,ab OR (pre op):ti,ab OR (Prehabilit*):kw,ti,ab OR (Pre-habilit*):kw,ti,ab OR [MH Preoperative care]) NOT ([MH Endometrial Neoplasms] OR [MH Ovarian Neoplasms] OR [MH Urinary Bladder Neoplasms] OR [MH Urogenital Neoplasms"[mh] OR "Whipple disease] OR Gynecologic:kw,ti,ab OR Urologic:kw,ti,ab OR Urothelial:kw,ti,ab OR Urethra:kw,ti,ab) ; 1/1/2000-12/31/2021 | | 2 |
| ClinTrials KQ6  (31Mar) | ("Bariatric Surgery" OR Fundoplication OR Splenectomy OR pancreatectomy OR Gastrectomy OR (("Upper Gastrointestinal" OR "upper gI") AND surgery)) AND (prehabilitation OR preoperative) AND (Anemia OR anaemia)\| Older Adult | | 0 |
| **KQ6**  **SEARCH RESULTS** | Total items identified by database searches | | 50 |
|  | Total trials identified in searches | | 1 |
|  | Total KQ6 duplicates removed during Topic 2 screening | | 44 |
|  | Total KQ6 records identified during Topic 2 screening | | 5 |
|  | Hand-searched items located after Topic 2 screening | | 0 |
|  | **Total items screened** | | **5** |

### KQ7

| **Q7: Should perioperative optimisation (vs. no optimisation) of anaemia be used elderly patients undergoing HPB surgery?** | | | |
| --- | --- | --- | --- |
| **Search Concepts** | | **Elderly + HPB + Anemia + Limits** | |
| **Database** | **Final search strategies** | | **Results** |
| PubMed  KQ7  (22 Mar) | (("Aged, 80 and over"[mh] OR "Frail Elderly"[mh] OR elderly[tiab] OR "older adult" [tw] OR septuagenar*[tw] OR octogenar*[tw] OR nonagenar*[tw] OR centenarian*[tw] OR supercentenar*[tw]) AND ("Hepato-pancreato-biliary"[tiab] OR HPB[tiab] OR hepatopancreaticobiliary[tiab] OR Whipple[tiab] OR Whipple [ot] OR ((liver[mh] OR liver[tiab] OR pancreas [mh] OR pancreas[tiab] OR gallbladder [mh] OR gallbladder [tiab] OR "bile ducts"[mh] OR "bile Duct*"[tiab] OR jejunum[mh] OR jejunum[tiab]) AND ("surgical procedures, operative"[mh] OR "general surgery"[mh] OR surgery[sh] OR surg*[tiab] OR resection[tiab])) OR Hepatectomy[mh] OR Hepatect*[tiab] OR pancreatectomy[mh] OR pancreatect*[tiab] OR Hepatojejunost*[tiab] OR ((Renal[tiab] OR biliary[tiab]) AND surger*[tiab])) AND ((Anemia[mh] OR Anemia[tiab] OR Anaemia[tiab]) AND ("Preoperative Care"[mh] OR preop*[tw] OR "pre-op"[tw] OR Prehabilit*[tw] OR Pre-habilit*[tw])) AND ("Clinical Study"[pt] OR "Comparative Study"[pt] OR "Epidemiologic studies" [mh] OR "Evaluation Study"[pt] OR "Meta-Analysis"[pt] OR "Multicenter Study"[pt] OR "Systematic Review"[pt] OR "Validation Study" [pt] OR randomized[tiab] OR analysis[tiab] OR study[tiab] OR studies [tiab] OR "Practice Guideline"[pt] OR "practice guidelines as topic"[mh] OR guideline[title] OR guidelines[title])) NOT (("animals"[MH:noexp] NOT "humans"[MH]) OR rat[tiab] OR rats[tiab] OR mouse[tiab] OR mice[tiab] OR dog[tiab] OR dogs[tiab] OR porcine[tiab] OR infant[mh] OR (child[mh] NOT adult[mh]) OR children[title] OR child[title] OR infant[title] OR infants[title] OR "Endometrial Neoplasms"[mh] OR "Ovarian Neoplasms"[mh] OR "Urinary Bladder Neoplasms"[mh] OR "Urogenital Neoplasms"[mh] OR "Whipple disease"[mh] OR "Whipple's disease"[tw] OR "Whipple disease"[tw] OR "Bladder cancer*"[tw] OR "Endometrial cancer*"[tw] OR "Ovarian cancer*"[tw] OR "Renal cancer*"[tw] OR Gynecologic[tiab] OR Urologic[tiab] OR Urothelial[tiab] OR Urethra[tiab] OR ("1905/01/01"[pdat]:"1999/12/31"[pdat])) | | 51 |
| Embase  KQ7  (22 Mar) | (('very elderly' OR 'frail elderly' OR elderly:kw,ti,ab OR 'septuagenarian*':kw,ti,ab OR 'octogenarian*':kw,ti,ab OR 'nonagenarian*':kw,ti,ab OR 'centenarian*':kw,ti,ab OR 'supercentenarian*':kw,ti,ab) AND ('biliary surgery' OR (('liver'/exp OR liver:kw,ti,ab OR 'pancreas'/exp OR pancreas:kw,ti,ab OR 'gallbladder'/exp OR gallbladder:kw,ti,ab OR 'bile duct'/exp OR 'bile duct' OR 'bile ducts' OR 'jejunum'/exp OR jejunum:kw,ti,ab) AND (resection:kw,ti,ab OR 'surgery'/exp OR surgery:kw,ti,ab OR surgical:kw,ti,ab)) OR 'liver surgery'/exp OR 'liver surgery' OR 'liver resection'/exp OR 'liver resection' OR 'pancreatectomy'/exp OR pancreatectomy OR 'hepatect*':kw,ti,ab OR 'nephrect*':kw,ti,ab OR 'hepatojejunostomy'/exp OR hepatojejunostomy:kw,ti,ab OR 'hepatopancreaticobiliary surgery' OR 'hepato pancreato biliary' OR hepatopancreaticobiliary OR HPB:kw,ti,ab OR 'biliary tract surgery'/exp OR 'biliary tract surgery' OR 'Renal surgery') AND (('preop*':ti,ab OR 'pre op':ti,ab OR 'Prehabilit*':kw,ti,ab OR 'Pre-habilit*':kw,ti,ab OR 'preoperative care'/exp ) AND ('anemia'/exp OR anemia:ti,ab OR Anaemia:ti,ab)) AND ('practice guideline'/exp OR 'practice guideline' OR [cochrane review]/lim OR [systematic review]/lim OR [meta analysis]/lim OR [clinical study]/lim OR 'clinical study'/exp OR 'case report' OR 'case study') AND [2000-2021]/py) NOT ([conference abstract]/lim OR [animals]/lim OR 'aged plant'/exp OR 'aged plant' OR 'child*':ti OR 'infant*':ti OR 'female genital tract tumor'/exp OR 'urogenital tract tumor'/exp OR (Whipple NEAR/3 disease) OR 'bladder cancer*':kw,ti,ab OR 'Endometrial cancer*':kw,ti,ab OR 'Ovarian cancer*':kw,ti,ab OR Gynecologic:kw,ti,ab OR Urologic:kw,ti,ab OR Urothelial:kw,ti,ab OR Urethra:kw,ti,ab) | | 22 |
| Cochrane  KQ7  (24 Mar) | ([MH Aged, 80 and over] OR [MH Frail Elderly] OR elderly:kw,ti,ab OR (septuagenarian*):kw,ti,ab OR (octogenarian*'):kw,ti,ab OR (nonagenarian*):kw,ti,ab OR (centenarian*):kw,ti,ab OR (supercentenarian*):kw,ti,ab) AND ([MH Hepatectomy] OR [MH pancreatectomy] OR (pancreatect*):ti,ab OR (Hepatojejunost*):ti,ab OR (Renal surger*):ti,ab OR (biliary surger*):ti,ab OR (Hepato-pancreato-biliary):ti,ab OR (HPB):ti,ab OR hepatopancreaticobiliary:ti,ab OR (([MH liver] OR (liver):ti,ab OR [MH pancreas] OR (pancreas):ti,ab OR [MH gallbladder] OR (gallbladder):ti,ab OR [MH bile ducts] OR (bile Duct*):ti,ab OR [MH kidney] OR (kidney*):ti,ab OR [MH jejunum] OR (jejunum):ti,ab) AND ([MH surgical procedures, operative] OR (resection):ti,ab,kw OR (surger*):ti,ab,kw)) OR (Hepatect*):ti,ab) AND ([MH Anemia] OR anemia:ti,ab OR Anaemia:ti,ab) AND ((preop*):ti,ab OR (pre op):ti,ab OR (Prehabilit*):kw,ti,ab OR (Pre-habilit*):kw,ti,ab OR [MH Preoperative care]) NOT ([MH Endometrial Neoplasms] OR [MH Ovarian Neoplasms] OR [MH Urinary Bladder Neoplasms] OR [MH Urogenital Neoplasms"[mh] OR "Whipple disease] OR Gynecologic:kw,ti,ab OR Urologic:kw,ti,ab OR Urothelial:kw,ti,ab OR Urethra:kw,ti,ab) ; 1/1/2000-12/31/2021 | | 5 |
| ClinTrials  (31 Mar) | ("Hepato-pancreato-biliary" OR hepatopancreaticobiliary OR Hepatectomy OR pancreatectomy) AND (prehabilitation OR preoperative) AND (Anemia OR anaemia)\| Older Adult | | 0 |
| **KQ7**  **SEARCH RESULTS** | Total items identified by database searches | | 78 |
|  | Total trials identified in searches | | 5 |
|  | Total KQ7 duplicates removed during Topic 2 screening | | 69 |
|  | Total KQ7 records identified during Topic 2 screening | | 4 |
|  | Hand-searched items located after Topic 2 screening | | 0 |
|  | **Total items screened** | | **4** |

### KQ8

| **Q8: Should perioperative optimisation (vs. no optimisation) of anaemia be used elderly patients undergoing hernia surgery?** | | | |
| --- | --- | --- | --- |
| **Search Concepts** | | **Elderly + Hernia + Anemia + Limits** | |
| **Database** | **Final search strategies** | | **Results** |
| PubMed  KQ8  (22 Mar) | (("Aged, 80 and over"[mh] OR "Frail Elderly"[mh] OR elderly[tiab] OR "older adult" [tw] OR septuagenar*[tw] OR octogenar*[tw] OR nonagenar*[tw] OR centenarian*[tw] OR supercentenar*[tw]) AND (((Hernia[tiab] OR Hernia[mh]) AND ("surgical procedures, operative"[mh] OR "general surgery"[mh] OR surgery[sh] OR surg*[tiab])) OR Herniorrhaphy[mh] OR Herniorrhap*[tiab]) AND ((Anemia[mh] OR Anemia[tiab] OR Anaemia[tiab]) AND ("Preoperative Care"[mh] OR preop*[tw] OR "pre-op"[tw] OR Prehabilit*[tw] OR Pre-habilit*[tw])) AND ("Clinical Study"[pt] OR "Comparative Study"[pt] OR "Epidemiologic studies" [mh] OR "Evaluation Study"[pt] OR "Meta-Analysis"[pt] OR "Multicenter Study"[pt] OR "Systematic Review"[pt] OR "Validation Study" [pt] OR randomized[tiab] OR analysis[tiab] OR study[tiab] OR studies [tiab] OR "Practice Guideline"[pt] OR "practice guidelines as topic"[mh] OR guideline[title] OR guidelines[title])) NOT (("animals"[MH:noexp] NOT "humans"[MH]) OR rat[tiab] OR rats[tiab] OR mouse[tiab] OR mice[tiab] OR dog[tiab] OR dogs[tiab] OR porcine[tiab] OR infant[mh] OR (child[mh] NOT adult[mh]) OR children[title] OR child[title] OR infant[title] OR infants[title] OR "Endometrial Neoplasms"[mh] OR "Ovarian Neoplasms"[mh] OR "Urinary Bladder Neoplasms"[mh] OR "Urogenital Neoplasms"[mh] OR "Whipple disease"[mh] OR "Whipple's disease"[tw] OR "Whipple disease"[tw] OR "Bladder cancer*"[tw] OR "Endometrial cancer*"[tw] OR "Ovarian cancer*"[tw] OR "Renal cancer*"[tw] OR Gynecologic[tiab] OR Urologic[tiab] OR Urothelial[tiab] OR Urethra[tiab] OR ("1905/01/01"[pdat]:"1999/12/31"[pdat])) | | 8 |
| Embase  KQ8  (22 Mar) | (('very elderly' OR 'frail elderly' OR elderly:kw,ti,ab OR 'septuagenarian*':kw,ti,ab OR 'octogenarian*':kw,ti,ab OR 'nonagenarian*':kw,ti,ab OR 'centenarian*':kw,ti,ab OR 'supercentenarian*':kw,ti,ab) AND ('hernia surgery'/exp OR 'herniorrhaphy'/exp OR 'herniorrhap*':kw,ti,ab OR (('hernia'/exp OR 'hernia':kw,ti,ab) AND ('surgery'/exp OR surgery:kw,ti,ab OR surgical:kw,ti,ab)) OR 'hernia surgery') AND (('preop*':ti,ab OR 'pre op':ti,ab OR 'Prehabilit*':kw,ti,ab OR 'Pre-habilit*':kw,ti,ab OR 'preoperative care'/exp ) AND ('anemia'/exp OR anemia:ti,ab OR Anaemia:ti,ab)) AND ('practice guideline'/exp OR 'practice guideline' OR [cochrane review]/lim OR [systematic review]/lim OR [meta analysis]/lim OR [clinical study]/lim OR 'clinical study'/exp OR 'case report' OR 'case study') AND [2000-2021]/py) NOT ([conference abstract]/lim OR [animals]/lim OR 'aged plant'/exp OR 'aged plant' OR 'child*':ti OR 'infant*':ti OR 'female genital tract tumor'/exp OR 'urogenital tract tumor'/exp OR (Whipple NEAR/3 disease) OR 'bladder cancer*':kw,ti,ab OR 'Endometrial cancer*':kw,ti,ab OR 'Ovarian cancer*':kw,ti,ab OR Gynecologic:kw,ti,ab OR Urologic:kw,ti,ab OR Urothelial:kw,ti,ab OR Urethra:kw,ti,ab) | | 7 |
| Cochrane  KQ8  (24 Mar) | ([MH Aged, 80 and over] OR [MH Frail Elderly] OR elderly:kw,ti,ab OR (septuagenarian*):kw,ti,ab OR (octogenarian*'):kw,ti,ab OR (nonagenarian*):kw,ti,ab OR (centenarian*):kw,ti,ab OR (supercentenarian*):kw,ti,ab) AND ([MH Herniorrhaphy] OR (((Hernia):ti,ab,kw OR [MH Hernia]) AND ([MH surgical procedures, operative] OR surgery:kw,ti,ab OR surgical:kw,ti,ab)) OR (Herniorrhap*):ti,ab,kw) AND ([MH Anemia] OR anemia:ti,ab OR Anaemia:ti,ab) AND ((preop*):ti,ab OR (pre op):ti,ab OR (Prehabilit*):kw,ti,ab OR (Pre-habilit*):kw,ti,ab OR [MH Preoperative care]) NOT ([MH Endometrial Neoplasms] OR [MH Ovarian Neoplasms] OR [MH Urinary Bladder Neoplasms] OR [MH Urogenital Neoplasms"[mh] OR "Whipple disease] OR Gynecologic:kw,ti,ab OR Urologic:kw,ti,ab OR Urothelial:kw,ti,ab OR Urethra:kw,ti,ab) ; 1/1/2000-12/31/2021 | | 0 |
| ClinTrials  (31 Mar) | ("Herniorrhaphy" OR (Hernia AND Surgery)) AND (prehabilitation OR preoperative) AND (Anemia OR anaemia)\| Older Adult | | 0 |
| **KQ8**  **SEARCH RESULTS** | Total items identified by database searches | | 15 |
|  | Total trials identified in searches | | 0 |
|  | Total KQ8 duplicates removed during Topic 2 screening | | 12 |
|  | Total KQ8 records identified during Topic 2 screening | | 3 |
|  | Hand-searched items located after Topic 2 screening | | 0 |
|  | **Total items screened** | | **3** |

### KQ9

| **Q9: Should smoking cessation (vs. no smoking cessation) be applied in elderly patients undergoing colorectal surgery?** | | | |
| --- | --- | --- | --- |
| **Search Concepts** | | **Elderly + Colorectal + Smoking Cessation + Limits** | |
| **Database** | **Final search strategies** | | **Results** |
| PubMed  KQ9  (22 Mar) | (("Aged, 80 and over"[mh] OR "Frail Elderly"[mh] OR elderly[tiab] OR "older adult" [tw] OR septuagenar*[tw] OR octogenar*[tw] OR nonagenar*[tw] OR centenarian*[tw] OR supercentenar*[tw]) AND ("Colorectal Surgery"[mh] OR ((abdomen[tiab] OR "anal canal"[tiab] OR "anal canal"[mh] OR anus[tiab] OR bowel[tiab] OR colon[tiab] OR colon [mh] OR rectum[tiab] OR rectum[mh] OR "Colon, Sigmoid"[mh] OR Sigmoid[tiab]) AND ("surgical procedures, operative"[mh] OR "general surgery"[mh] OR surgery[sh] OR resection[tiab] OR surg*[tiab])) OR ((rectal [tiab] OR Colorectal[tiab] OR abdominal[tiab]) AND Surger*[tiab])) AND (("Smoking Cessation"[mh] OR "Tobacco Use Cessation Devices"[mh] OR "Tobacco Use Cessation"[mh] OR "Smoking Reduction"[mh] OR ((Ex-Smokers[mh] OR Smok*[tiab] OR Smoking [ot]) AND (Cease[tiab] OR Cessation[tiab] OR stop*[tiab] OR "giving up"[tiab] OR quit*[tiab] OR reduction[tiab] OR reduce[tiab] OR abstinen*[tiab] OR decreas*[tiab]))) AND (preop*[tw] OR "pre-op"[tw] OR Preoperative Care[mh] OR Prehabilit*[tw] OR Pre-habilit*[tw])) AND ("Clinical Study"[pt] OR "Comparative Study"[pt] OR "Epidemiologic studies" [mh] OR "Evaluation Study"[pt] OR "Meta-Analysis"[pt] OR "Multicenter Study"[pt] OR "Systematic Review"[pt] OR "Validation Study" [pt] OR randomized[tiab] OR analysis[tiab] OR study[tiab] OR studies [tiab] OR "Practice Guideline"[pt] OR "practice guidelines as topic"[mh] OR guideline[title] OR guidelines[title])) NOT (("animals"[MH:noexp] NOT "humans"[MH]) OR rat[tiab] OR rats[tiab] OR mouse[tiab] OR mice[tiab] OR dog[tiab] OR dogs[tiab] OR porcine[tiab] OR infant[mh] OR (child[mh] NOT adult[mh]) OR children[title] OR child[title] OR infant[title] OR infants[title] OR "Endometrial Neoplasms"[mh] OR "Ovarian Neoplasms"[mh] OR "Urinary Bladder Neoplasms"[mh] OR "Urogenital Neoplasms"[mh] OR "Whipple disease"[mh] OR "Whipple's disease"[tw] OR "Whipple disease"[tw] OR "Bladder cancer*"[tw] OR "Endometrial cancer*"[tw] OR "Ovarian cancer*"[tw] OR "Renal cancer*"[tw] OR Gynecologic[tiab] OR Urologic[tiab] OR Urothelial[tiab] OR Urethra[tiab] OR ("1905/01/01"[pdat]:"1999/12/31"[pdat])) | | 22 |
| Embase  KQ9  (22 Mar) | (('very elderly' OR 'frail elderly' OR elderly:kw,ti,ab OR 'septuagenarian*':kw,ti,ab OR 'octogenarian*':kw,ti,ab OR 'nonagenarian*':kw,ti,ab OR 'centenarian*':kw,ti,ab OR 'supercentenarian*':kw,ti,ab) AND ('colorectal surgery'/exp OR 'abdominal surgery'/exp OR 'abdominal surgery' OR 'rectum surgery'/exp OR 'rectum surgery' OR (('abdomen'/exp OR abdomen OR 'anal canal'/exp OR 'anal canal' OR 'anus'/exp OR anus OR 'colon'/exp OR colon OR 'rectum'/exp OR rectum OR 'intestine'/exp OR intestine:ti,ab OR 'sigmoid'/exp OR sigmoid) AND (resection:kw,ti,ab OR 'surgery'/exp OR surgery:kw,ti,ab OR surgical:kw,ti,ab)) OR 'colorectal surgery') AND (('preop*':ti,ab OR 'pre op':ti,ab OR 'prehabilit*':kw,ti,ab OR 'pre-habilit*':kw,ti,ab OR 'preoperative care'/exp) AND ('smoking cessation'/exp OR 'nicotine gum'/exp OR 'nicotine gum' OR (('ex smoker*' OR smok*:ti,ab) AND (cease:ti,ab OR cessation:ti,ab OR stop*:ti,ab OR 'giving up':ti,ab OR quit*:ti,ab OR reduction:ti,ab OR reduce:ti,ab OR abstinen*:ti,ab OR decreas*:ti,ab)) OR 'smoking cessation')) AND ('practice guideline'/exp OR 'practice guideline' OR [cochrane review]/lim OR [systematic review]/lim OR [meta analysis]/lim OR [clinical study]/lim OR 'clinical study'/exp OR 'case report' OR 'case study') AND [2000-2021]/py) NOT ([conference abstract]/lim OR [animals]/lim OR 'aged plant'/exp OR 'aged plant' OR 'child*':ti OR 'infant*':ti OR 'female genital tract tumor'/exp OR 'urogenital tract tumor'/exp OR (Whipple NEAR/3 disease) OR 'bladder cancer*':kw,ti,ab OR 'Endometrial cancer*':kw,ti,ab OR 'Ovarian cancer*':kw,ti,ab OR Gynecologic:kw,ti,ab OR Urologic:kw,ti,ab OR Urothelial:kw,ti,ab OR Urethra:kw,ti,ab) | | 9 |
| Cochrane  KQ9  (24 Mar) | ([MH Aged, 80 and over] OR [MH Frail Elderly] OR elderly:kw,ti,ab OR (septuagenarian*):kw,ti,ab OR (octogenarian*'):kw,ti,ab OR (nonagenarian*):kw,ti,ab OR (centenarian*):kw,ti,ab OR (supercentenarian*):kw,ti,ab) AND ([MH Colorectal Surgery] OR (abdominal surgery):kw,ti,ab OR (rectal surgery):kw,ti,ab OR (abdomen:ti,ab OR (anal canal):ti,ab OR (anus):ti,ab OR (colon):ti,ab OR (rectum):ti,ab OR (bowel):ti,ab OR (Sigmoid):ti,ab OR [MH anal canal] OR [MH colon] OR [MH rectum] ) AND ([MH surgical procedures, operative] OR resection:kw,ti,ab OR surgery:kw,ti,ab OR surgical:kw,ti,ab)) OR (colorectal surgery):kw,ti,ab) AND ([MH Smoking Cessation] OR [MH Tobacco Use Cessation Device] OR [MH Tobacco Use Cessation] OR [MH Smoking Reduction] OR (((ex smoker*):ti,ab OR (smok*):ti,ab) AND ((Cease):ti,ab OR (Cessation):ti,ab OR (stop*):ti,ab OR (giving up):ti,ab OR (quit*):ti,ab OR (reduc*):ti,ab OR (abstinen*):ti,ab OR (decreas*):ti,ab))) AND ((preop*):ti,ab OR (pre op):ti,ab OR (Prehabilit*):kw,ti,ab OR (Pre-habilit*):kw,ti,ab OR [MH Preoperative care]) NOT ([MH Endometrial Neoplasms] OR [MH Ovarian Neoplasms] OR [MH Urinary Bladder Neoplasms] OR [MH Urogenital Neoplasms"[mh] OR "Whipple disease] OR Gynecologic:kw,ti,ab OR Urologic:kw,ti,ab OR Urothelial:kw,ti,ab OR Urethra:kw,ti,ab) ; 1/1/2000-12/31/2021 | | 2 |
| ClinTrials  (31 Mar) | ("Colorectal Surgery" OR "Abdominal Surgery" OR "Rectal Surgery") AND ("Smoking Cessation") \| Older Adult | | 1 |
| **KQ9**  **SEARCH RESULTS** | Total items identified by database searches | | 34 |
|  | Total trials identified in searches | | 1 |
|  | Total KQ9 duplicates removed during Topic 2 screening | | 31 |
|  | Total KQ9 records identified during Topic 2 screening | | 2 |
|  | Hand-searched items located after Topic 2 screening | | 0 |
|  | **Total items screened** | | **2** |

### KQ10

| **Q10: Should smoking cessation (vs. no smoking cessation) be applied in elderly patients undergoing UGI surgery?** | | | |
| --- | --- | --- | --- |
| **Search Concepts** | | **Elderly + UGI + Smoking Cessation + Limits** | |
| **Database** | **Final search strategies** | | **Results** |
| PubMed  KQ10  (1 Apr) | (("Aged, 80 and over"[mh] OR "Frail Elderly"[mh] OR elderly[tiab] OR "older adult" [tw] OR septuagenar*[tw] OR octogenar*[tw] OR nonagenar*[tw] OR centenarian*[tw] OR supercentenar*[tw]) AND ((("Upper Gastrointestinal Tract" [mh] OR esophagus[mh] OR esophagus[tiab] OR stomach[mh] OR stomach[tiab] OR duodenum[mh] OR duodenum[tiab] OR Spleen[mh] OR Spleen[tiab]) AND ("surgical procedures, operative"[mh] OR "general surgery"[mh] OR surgery[sh] OR resection[tiab] OR surg*[tiab])) OR "Bariatric Surgery"[mh] OR Fundoplication[mh] OR Fundoplication[tiab] OR Gastrectomy[mh] OR Gastrect*[tiab] OR Oesophagect*[tiab] OR Esophagectomy[mh] OR Esophagect*[tiab] OR Splenectomy[mh] OR Splenect*[tiab] OR ((UGI[tiab] OR "Upper GI*" OR "gastro-esophageal"[tiab] OR "Upper Gastrointest*"[tiab]) AND Surger*[tiab])) AND (("Smoking Cessation"[mh] OR "Tobacco Use Cessation Devices"[mh] OR "Tobacco Use Cessation"[mh] OR "Smoking Reduction"[mh] OR ((Ex-Smokers[mh] OR Smok*[tiab] OR Smoking [ot]) AND (Cease[tiab] OR Cessation[tiab] OR stop*[tiab] OR "giving up"[tiab] OR quit*[tiab] OR reduction[tiab] OR reduce[tiab] OR abstinen*[tiab] OR decreas*[tiab]))) AND (preop*[tw] OR "pre-op"[tw] OR Preoperative Care[mh] OR Prehabilit*[tw] OR Pre-habilit*[tw])) AND ("Clinical Study"[pt] OR "Comparative Study"[pt] OR "Epidemiologic studies" [mh] OR "Evaluation Study"[pt] OR "Meta-Analysis"[pt] OR "Multicenter Study"[pt] OR "Systematic Review"[pt] OR "Validation Study" [pt] OR randomized[tiab] OR analysis[tiab] OR study[tiab] OR studies [tiab] OR "Practice Guideline"[pt] OR "practice guidelines as topic"[mh] OR guideline[title] OR guidelines[title])) NOT (("animals"[MH:noexp] NOT "humans"[MH]) OR rat[tiab] OR rats[tiab] OR mouse[tiab] OR mice[tiab] OR dog[tiab] OR dogs[tiab] OR porcine[tiab] OR infant[mh] OR (child[mh] NOT adult[mh]) OR children[title] OR child[title] OR infant[title] OR infants[title] OR "Endometrial Neoplasms"[mh] OR "Esophageal Neoplasms"[mh] OR "Ovarian Neoplasms"[mh] OR "Urinary Bladder Neoplasms"[mh] OR "Urogenital Neoplasms"[mh] OR "Whipple disease"[mh] OR "Whipple's disease"[tw] OR "Whipple disease"[tw] OR "Bladder cancer*"[tw] OR "Endometrial cancer*"[tw] OR "Ovarian cancer*"[tw] OR "Renal cancer*"[tw] OR Gynecologic[tiab] OR Urologic[tiab] OR Urothelial[tiab] OR Urethra[tiab] OR ("1905/01/01"[pdat]:"1999/12/31"[pdat])) | | 6 |
| Embase  KQ10  (22 Mar) | (('very elderly' OR 'frail elderly' OR elderly:kw,ti,ab OR 'septuagenarian*':kw,ti,ab OR 'octogenarian*':kw,ti,ab OR 'nonagenarian*':kw,ti,ab OR 'centenarian*':kw,ti,ab OR 'supercentenarian*':kw,ti,ab) AND ('Splenectomy'/exp OR (('upper gastrointestinal tract'/exp OR 'upper intestine*':kw,ti,ab OR 'esophagus'/exp OR esophagus:kw,ti,ab OR 'spleen'/exp OR spleen:kw,ti,ab OR 'stomach'/exp OR stomach:kw,ti,ab OR 'duodenum'/exp OR duodenum:kw,ti,ab OR 'upper gi*':kw,ti,ab OR ugi:ti,ab) AND (resection:kw,ti,ab OR 'surgery'/exp OR surgery:kw,ti,ab OR surgical:kw,ti,ab)) OR 'stomach fundoplication'/exp OR fundoplication:kw,ti,ab OR 'gastrectomy'/exp OR gastrectomy:kw,ti,ab OR 'gastrect*':kw,ti,ab OR oesophagectomy OR 'esophagus resection'/exp OR 'esophagus resection':kw,ti,ab OR esophagectomy:kw,ti,ab OR 'Splenect*':kw,ti,ab) AND (('preop*':ti,ab OR 'pre op':ti,ab OR 'prehabilit*':kw,ti,ab OR 'pre-habilit*':kw,ti,ab OR 'preoperative care'/exp) AND ('smoking cessation'/exp OR 'nicotine gum'/exp OR 'nicotine gum' OR (('ex smoker*' OR smok*:ti,ab) AND (cease:ti,ab OR cessation:ti,ab OR stop*:ti,ab OR 'giving up':ti,ab OR quit*:ti,ab OR reduction:ti,ab OR reduce:ti,ab OR abstinen*:ti,ab OR decreas*:ti,ab)) OR 'smoking cessation')) AND ('practice guideline'/exp OR 'practice guideline' OR [cochrane review]/lim OR [systematic review]/lim OR [meta analysis]/lim OR [clinical study]/lim OR 'clinical study'/exp OR 'case report' OR 'case study') AND [2000-2021]/py) NOT ([conference abstract]/lim OR [animals]/lim OR 'aged plant'/exp OR 'aged plant' OR 'child*':ti OR 'infant*':ti OR 'female genital tract tumor'/exp OR 'urogenital tract tumor'/exp OR 'esophagus tumor'/exp OR (Whipple NEAR/3 disease) OR 'bladder cancer*':kw,ti,ab OR 'Endometrial cancer*':kw,ti,ab OR 'Ovarian cancer*':kw,ti,ab OR Gynecologic:kw,ti,ab OR Urologic:kw,ti,ab OR Urothelial:kw,ti,ab OR Urethra:kw,ti,ab) | | 2 |
| Cochrane  KQ10  (24 Mar) | ([MH Aged, 80 and over] OR [MH Frail Elderly] OR elderly:kw,ti,ab OR (septuagenarian*):kw,ti,ab OR (octogenarian*'):kw,ti,ab OR (nonagenarian*):kw,ti,ab OR (centenarian*):kw,ti,ab OR (supercentenarian*):kw,ti,ab) AND ([MH Splenectomy] OR (([MH upper gastrointestinal tract] OR (upper intestine*):kw,ti,ab OR OR [MH esophagus] OR [MH stomach] OR [MH duodenum] OR [MH spleen] OR esophagus:ti,ab OR OR stomach:ti,ab OR duodenum:ti,ab OR spleen:ti,ab OR 'upper gi*':kw,ti,ab OR ugi:kw,ti,ab OR (gastro-esophageal):ti,ab) AND (resection:kw,ti,ab OR [MH surgical procedures, operative] OR surgery:kw,ti,ab OR surgical:kw,ti,ab)) OR [MH Fundoplication] OR fundoplication:kw,ti,ab OR [MH Gastrectomy] OR gastrectomy:kw,ti,ab OR (gastrect*):kw,ti,ab OR oesophagectomy OR (Splenect*):kw,ti,ab) AND ([MH Smoking Cessation] OR [MH Tobacco Use Cessation Device] OR [MH Tobacco Use Cessation] OR [MH Smoking Reduction] OR (((ex smoker*):ti,ab OR (smok*):ti,ab) AND ((Cease):ti,ab OR (Cessation):ti,ab OR (stop*):ti,ab OR (giving up):ti,ab OR (quit*):ti,ab OR (reduc*):ti,ab OR (abstinen*):ti,ab OR (decreas*):ti,ab))) AND ((preop*):ti,ab OR (pre op):ti,ab OR (Prehabilit*):kw,ti,ab OR (Pre-habilit*):kw,ti,ab OR [MH Preoperative care]) NOT ([MH Endometrial Neoplasms] OR [MH Ovarian Neoplasms] OR [MH Urinary Bladder Neoplasms] OR [MH Urogenital Neoplasms"[mh] OR "Whipple disease] OR Gynecologic:kw,ti,ab OR Urologic:kw,ti,ab OR Urothelial:kw,ti,ab OR Urethra:kw,ti,ab) ; 1/1/2000-12/31/2021 | | 0 |
| ClinTrials  (31 Mar) | ("Bariatric Surgery" OR Fundoplication OR Splenectomy OR pancreatectomy OR Gastrectomy OR (("Upper Gastrointestinal" OR "upper gI") AND surgery)) AND ("Smoking Cessation") \| Older Adult | | 2 |
| **KQ10**  **SEARCH RESULTS** | Total items identified by database searches | | 10 |
|  | Total trials identified in searches | | 2 |
|  | Total KQ10 duplicates removed during Topic 2 screening | | 6 |
|  | Total KQ10 records identified during Topic 2 screening | | 2 |
|  | Hand-searched items located after Topic 2 screening | | 0 |
|  | **Total items screened** | | **2** |

### KQ11

| **Q11: Should smoking cessation (vs. no smoking cessation) be applied in elderly patients undergoing HPB surgery?** | | | |
| --- | --- | --- | --- |
| **Search Concepts** | | **Elderly + HPB + Smoking Cessation + Limits** | |
| **Database** | **Final search strategies** | | **Results** |
| PubMed  KQ11  (22 Mar) | (("Aged, 80 and over"[mh] OR "Frail Elderly"[mh] OR elderly[tiab] OR "older adult" [tw] OR septuagenar*[tw] OR octogenar*[tw] OR nonagenar*[tw] OR centenarian*[tw] OR supercentenar*[tw]) AND ("Hepato-pancreato-biliary"[tiab] OR HPB[tiab] OR hepatopancreaticobiliary[tiab] OR Whipple[tiab] OR Whipple [ot] OR ((liver[mh] OR liver[tiab] OR pancreas [mh] OR pancreas[tiab] OR gallbladder [mh] OR gallbladder [tiab] OR "bile ducts"[mh] OR "bile Duct*"[tiab] OR jejunum[mh] OR jejunum[tiab]) AND ("surgical procedures, operative"[mh] OR "general surgery"[mh] OR surgery[sh] OR surg*[tiab] OR resection[tiab])) OR Hepatectomy[mh] OR Hepatect*[tiab] OR pancreatectomy[mh] OR pancreatect*[tiab] OR Hepatojejunost*[tiab] OR ((Renal[tiab] OR biliary[tiab]) AND surger*[tiab])) AND (("Smoking Cessation"[mh] OR "Tobacco Use Cessation Devices"[mh] OR "Tobacco Use Cessation"[mh] OR "Smoking Reduction"[mh] OR ((Ex-Smokers[mh] OR Smok*[tiab] OR Smoking [ot]) AND (Cease[tiab] OR Cessation[tiab] OR stop*[tiab] OR "giving up"[tiab] OR quit*[tiab] OR reduction[tiab] OR reduce[tiab] OR abstinen*[tiab] OR decreas*[tiab]))) AND (preop*[tw] OR "pre-op"[tw] OR Preoperative Care[mh] OR Prehabilit*[tw] OR Pre-habilit*[tw])) AND ("Clinical Study"[pt] OR "Comparative Study"[pt] OR "Epidemiologic studies" [mh] OR "Evaluation Study"[pt] OR "Meta-Analysis"[pt] OR "Multicenter Study"[pt] OR "Systematic Review"[pt] OR "Validation Study" [pt] OR randomized[tiab] OR analysis[tiab] OR study[tiab] OR studies [tiab] OR "Practice Guideline"[pt] OR "practice guidelines as topic"[mh] OR guideline[title] OR guidelines[title])) NOT (("animals"[MH:noexp] NOT "humans"[MH]) OR rat[tiab] OR rats[tiab] OR mouse[tiab] OR mice[tiab] OR dog[tiab] OR dogs[tiab] OR porcine[tiab] OR infant[mh] OR (child[mh] NOT adult[mh]) OR children[title] OR child[title] OR infant[title] OR infants[title] OR "Endometrial Neoplasms"[mh] OR "Ovarian Neoplasms"[mh] OR "Urinary Bladder Neoplasms"[mh] OR "Urogenital Neoplasms"[mh] OR "Whipple disease"[mh] OR "Whipple's disease"[tw] OR "Whipple disease"[tw] OR "Bladder cancer*"[tw] OR "Endometrial cancer*"[tw] OR "Ovarian cancer*"[tw] OR "Renal cancer*"[tw] OR Gynecologic[tiab] OR Urologic[tiab] OR Urothelial[tiab] OR Urethra[tiab] OR ("1905/01/01"[pdat]:"1999/12/31"[pdat])) | | 13 |
| Embase  KQ11  (22 Mar) | (('very elderly' OR 'frail elderly' OR elderly:kw,ti,ab OR 'septuagenarian*':kw,ti,ab OR 'octogenarian*':kw,ti,ab OR 'nonagenarian*':kw,ti,ab OR 'centenarian*':kw,ti,ab OR 'supercentenarian*':kw,ti,ab) AND ('biliary surgery' OR (('liver'/exp OR liver:kw,ti,ab OR 'pancreas'/exp OR pancreas:kw,ti,ab OR 'gallbladder'/exp OR gallbladder:kw,ti,ab OR 'bile duct'/exp OR 'bile duct' OR 'bile ducts' OR 'jejunum'/exp OR jejunum:kw,ti,ab) AND (resection:kw,ti,ab OR 'surgery'/exp OR surgery:kw,ti,ab OR surgical:kw,ti,ab)) OR 'liver surgery'/exp OR 'liver surgery' OR 'liver resection'/exp OR 'liver resection' OR 'pancreatectomy'/exp OR pancreatectomy OR 'hepatect*':kw,ti,ab OR 'nephrect*':kw,ti,ab OR 'hepatojejunostomy'/exp OR hepatojejunostomy:kw,ti,ab OR 'hepatopancreaticobiliary surgery' OR 'hepato pancreato biliary' OR hepatopancreaticobiliary OR HPB:kw,ti,ab OR 'biliary tract surgery'/exp OR 'biliary tract surgery' OR 'Renal surgery') AND (('preop*':ti,ab OR 'pre op':ti,ab OR 'prehabilit*':kw,ti,ab OR 'pre-habilit*':kw,ti,ab OR 'preoperative care'/exp) AND ('smoking cessation'/exp OR 'nicotine gum'/exp OR 'nicotine gum' OR (('ex smoker*' OR smok*:ti,ab) AND (cease:ti,ab OR cessation:ti,ab OR stop*:ti,ab OR 'giving up':ti,ab OR quit*:ti,ab OR reduction:ti,ab OR reduce:ti,ab OR abstinen*:ti,ab OR decreas*:ti,ab)) OR 'smoking cessation')) AND ('practice guideline'/exp OR 'practice guideline' OR [cochrane review]/lim OR [systematic review]/lim OR [meta analysis]/lim OR [clinical study]/lim OR 'clinical study'/exp OR 'case report' OR 'case study') AND [2000-2021]/py) NOT ([conference abstract]/lim OR [animals]/lim OR 'aged plant'/exp OR 'aged plant' OR 'child*':ti OR 'infant*':ti OR 'female genital tract tumor'/exp OR 'urogenital tract tumor'/exp OR (Whipple NEAR/3 disease) OR 'bladder cancer*':kw,ti,ab OR 'Endometrial cancer*':kw,ti,ab OR 'Ovarian cancer*':kw,ti,ab OR Gynecologic:kw,ti,ab OR Urologic:kw,ti,ab OR Urothelial:kw,ti,ab OR Urethra:kw,ti,ab) | | 2 |
| Cochrane  KQ11  (24 Mar) | ([MH Aged, 80 and over] OR [MH Frail Elderly] OR elderly:kw,ti,ab OR (septuagenarian*):kw,ti,ab OR (octogenarian*'):kw,ti,ab OR (nonagenarian*):kw,ti,ab OR (centenarian*):kw,ti,ab OR (supercentenarian*):kw,ti,ab) AND ([MH Hepatectomy] OR [MH pancreatectomy] OR (pancreatect*):ti,ab OR (Hepatojejunost*):ti,ab OR (Renal surger*):ti,ab OR (biliary surger*):ti,ab OR (Hepato-pancreato-biliary):ti,ab OR (HPB):ti,ab OR hepatopancreaticobiliary:ti,ab OR (([MH liver] OR (liver):ti,ab OR [MH pancreas] OR (pancreas):ti,ab OR [MH gallbladder] OR (gallbladder):ti,ab OR [MH bile ducts] OR (bile Duct*):ti,ab OR [MH kidney] OR (kidney*):ti,ab OR [MH jejunum] OR (jejunum):ti,ab) AND ([MH surgical procedures, operative] OR (resection):ti,ab,kw OR (surger*):ti,ab,kw)) OR (Hepatect*):ti,ab) AND ([MH Smoking Cessation] OR [MH Tobacco Use Cessation Device] OR [MH Tobacco Use Cessation] OR [MH Smoking Reduction] OR (((ex smoker*):ti,ab OR (smok*):ti,ab) AND ((Cease):ti,ab OR (Cessation):ti,ab OR (stop*):ti,ab OR (giving up):ti,ab OR (quit*):ti,ab OR (reduc*):ti,ab OR (abstinen*):ti,ab OR (decreas*):ti,ab))) AND ((preop*):ti,ab OR (pre op):ti,ab OR (Prehabilit*):kw,ti,ab OR (Pre-habilit*):kw,ti,ab OR [MH Preoperative care]) NOT ([MH Endometrial Neoplasms] OR [MH Ovarian Neoplasms] OR [MH Urinary Bladder Neoplasms] OR [MH Urogenital Neoplasms"[mh] OR "Whipple disease] OR Gynecologic:kw,ti,ab OR Urologic:kw,ti,ab OR Urothelial:kw,ti,ab OR Urethra:kw,ti,ab) ; 1/1/2000-12/31/2021 | | 2 |
| ClinTrials  (31 Mar) | ("Hepato-pancreato-biliary" OR hepatopancreaticobiliary OR Hepatectomy OR pancreatectomy) AND ("Smoking Cessation") \| Older Adult | | 1 |
| **KQ11**  **SEARCH RESULTS** | Total items identified by database searches | | 18 |
|  | Total trials identified in searches | | 2 |
|  | Total KQ11 duplicates removed during Topic 2 screening | | 15 |
|  | Total KQ11 records identified during Topic 2 screening | | 1 |
|  | Hand-searched items located after Topic 2 screening | | 0 |
|  | **Total items screened** | | **1** |

### KQ12

| **Q12: Should smoking cessation (vs. no smoking cessation) be applied in elderly patients undergoing hernia surgery?** | | | |
| --- | --- | --- | --- |
| **Search Concepts** | | **Elderly + Hernia + Smoking Cessation + Limits** | |
| **Database** | **Final search strategies** | | **Results** |
| PubMed  KQ12  (22 Mar) | (("Aged, 80 and over"[mh] OR "Frail Elderly"[mh] OR elderly[tiab] OR "older adult" [tw] OR septuagenar*[tw] OR octogenar*[tw] OR nonagenar*[tw] OR centenarian*[tw] OR supercentenar*[tw]) AND (((Hernia[tiab] OR Hernia[mh]) AND ("surgical procedures, operative"[mh] OR "general surgery"[mh] OR surgery[sh] OR surg*[tiab])) OR Herniorrhaphy[mh] OR Herniorrhap*[tiab]) AND (("Smoking Cessation"[mh] OR "Tobacco Use Cessation Devices"[mh] OR "Tobacco Use Cessation"[mh] OR "Smoking Reduction"[mh] OR ((Ex-Smokers[mh] OR Smok*[tiab] OR Smoking [ot]) AND (Cease[tiab] OR Cessation[tiab] OR stop*[tiab] OR "giving up"[tiab] OR quit*[tiab] OR reduction[tiab] OR reduce[tiab] OR abstinen*[tiab] OR decreas*[tiab]))) AND (preop*[tw] OR "pre-op"[tw] OR Preoperative Care[mh] OR Prehabilit*[tw] OR Pre-habilit*[tw])) AND ("Clinical Study"[pt] OR "Comparative Study"[pt] OR "Epidemiologic studies" [mh] OR "Evaluation Study"[pt] OR "Meta-Analysis"[pt] OR "Multicenter Study"[pt] OR "Systematic Review"[pt] OR "Validation Study" [pt] OR randomized[tiab] OR analysis[tiab] OR study[tiab] OR studies [tiab] OR "Practice Guideline"[pt] OR "practice guidelines as topic"[mh] OR guideline[title] OR guidelines[title])) NOT (("animals"[MH:noexp] NOT "humans"[MH]) OR rat[tiab] OR rats[tiab] OR mouse[tiab] OR mice[tiab] OR dog[tiab] OR dogs[tiab] OR porcine[tiab] OR infant[mh] OR (child[mh] NOT adult[mh]) OR children[title] OR child[title] OR infant[title] OR infants[title] OR "Endometrial Neoplasms"[mh] OR "Ovarian Neoplasms"[mh] OR "Urinary Bladder Neoplasms"[mh] OR "Urogenital Neoplasms"[mh] OR "Whipple disease"[mh] OR "Whipple's disease"[tw] OR "Whipple disease"[tw] OR "Bladder cancer*"[tw] OR "Endometrial cancer*"[tw] OR "Ovarian cancer*"[tw] OR "Renal cancer*"[tw] OR Gynecologic[tiab] OR Urologic[tiab] OR Urothelial[tiab] OR Urethra[tiab] OR ("1905/01/01"[pdat]:"1999/12/31"[pdat])) | | 5 |
| Embase  KQ12  (22 Mar) | (('very elderly' OR 'frail elderly' OR elderly:kw,ti,ab OR 'septuagenarian*':kw,ti,ab OR 'octogenarian*':kw,ti,ab OR 'nonagenarian*':kw,ti,ab OR 'centenarian*':kw,ti,ab OR 'supercentenarian*':kw,ti,ab) AND ('hernia surgery'/exp OR 'herniorrhaphy'/exp OR 'herniorrhap*':kw,ti,ab OR (('hernia'/exp OR 'hernia':kw,ti,ab) AND ('surgery'/exp OR surgery:kw,ti,ab OR surgical:kw,ti,ab)) OR 'hernia surgery') AND (('preop*':ti,ab OR 'pre op':ti,ab OR 'prehabilit*':kw,ti,ab OR 'pre-habilit*':kw,ti,ab OR 'preoperative care'/exp) AND ('smoking cessation'/exp OR 'nicotine gum'/exp OR 'nicotine gum' OR (('ex smoker*' OR smok*:ti,ab) AND (cease:ti,ab OR cessation:ti,ab OR stop*:ti,ab OR 'giving up':ti,ab OR quit*:ti,ab OR reduction:ti,ab OR reduce:ti,ab OR abstinen*:ti,ab OR decreas*:ti,ab)) OR 'smoking cessation')) AND ('practice guideline'/exp OR 'practice guideline' OR [cochrane review]/lim OR [systematic review]/lim OR [meta analysis]/lim OR [clinical study]/lim OR 'clinical study'/exp OR 'case report' OR 'case study') AND [2000-2021]/py) NOT ([conference abstract]/lim OR [animals]/lim OR 'aged plant'/exp OR 'aged plant' OR 'child*':ti OR 'infant*':ti OR 'female genital tract tumor'/exp OR 'urogenital tract tumor'/exp OR (Whipple NEAR/3 disease) OR 'bladder cancer*':kw,ti,ab OR 'Endometrial cancer*':kw,ti,ab OR 'Ovarian cancer*':kw,ti,ab OR Gynecologic:kw,ti,ab OR Urologic:kw,ti,ab OR Urothelial:kw,ti,ab OR Urethra:kw,ti,ab) | | 1 |
| Cochrane  KQ12  (24 Mar) | ([MH Aged, 80 and over] OR [MH Frail Elderly] OR elderly:kw,ti,ab OR (septuagenarian*):kw,ti,ab OR (octogenarian*'):kw,ti,ab OR (nonagenarian*):kw,ti,ab OR (centenarian*):kw,ti,ab OR (supercentenarian*):kw,ti,ab) AND ([MH Herniorrhaphy] OR (((Hernia):ti,ab,kw OR [MH Hernia]) AND ([MH surgical procedures, operative] OR surgery:kw,ti,ab OR surgical:kw,ti,ab)) OR (Herniorrhap*):ti,ab,kw) AND ([MH Smoking Cessation] OR [MH Tobacco Use Cessation Device] OR [MH Tobacco Use Cessation] OR [MH Smoking Reduction] OR (((ex smoker*):ti,ab OR (smok*):ti,ab) AND ((Cease):ti,ab OR (Cessation):ti,ab OR (stop*):ti,ab OR (giving up):ti,ab OR (quit*):ti,ab OR (reduc*):ti,ab OR (abstinen*):ti,ab OR (decreas*):ti,ab))) AND ((preop*):ti,ab OR (pre op):ti,ab OR (Prehabilit*):kw,ti,ab OR (Pre-habilit*):kw,ti,ab OR [MH Preoperative care]) NOT ([MH Endometrial Neoplasms] OR [MH Ovarian Neoplasms] OR [MH Urinary Bladder Neoplasms] OR [MH Urogenital Neoplasms"[mh] OR "Whipple disease] OR Gynecologic:kw,ti,ab OR Urologic:kw,ti,ab OR Urothelial:kw,ti,ab OR Urethra:kw,ti,ab) ; 1/1/2000-12/31/2021 | | 0 |
| ClinTrials  (31 Mar) | ("Herniorrhaphy" OR (Hernia AND Surgery)) AND ("Smoking Cessation")\| Older Adult | | 1 |
| **KQ12**  **SEARCH RESULTS** | Total items identified by database searches | | 7 |
|  | Total trials identified in searches | | 1 |
|  | Total KQ12 duplicates removed during Topic 2 screening | | 5 |
|  | Total KQ12 records identified during Topic 2 screening | | 1 |
|  | Hand-searched items located after Topic 2 screening | | 0 |
|  | **Total items screened** | | **1** |

### KQ13

| **Q13: Should excess alcohol cessation (vs. no alcohol cessation) be applied in elderly patients undergoing colorectal surgery?** | | | |
| --- | --- | --- | --- |
| **Search Concepts** | | **Elderly + Colorectal + Alcohol Cessation + Limits** | |
| **Database** | **Final search strategies** | | **Results** |
| PubMed  KQ13  (22 Mar) | (("Aged, 80 and over"[mh] OR "Frail Elderly"[mh] OR elderly[tiab] OR "older adult" [tw] OR septuagenar*[tw] OR octogenar*[tw] OR nonagenar*[tw] OR centenarian*[tw] OR supercentenar*[tw]) AND ("Colorectal Surgery"[mh] OR ((abdomen[tiab] OR "anal canal"[tiab] OR "anal canal"[mh] OR anus[tiab] OR bowel[tiab] OR colon[tiab] OR colon [mh] OR rectum[tiab] OR rectum[mh] OR "Colon, Sigmoid"[mh] OR Sigmoid[tiab]) AND ("surgical procedures, operative"[mh] OR "general surgery"[mh] OR surgery[sh] OR resection[tiab] OR surg*[tiab])) OR ((rectal [tiab] OR Colorectal[tiab] OR abdominal[tiab]) AND Surger*[tiab])) AND (("Alcohol Abstinence"[mh] OR (Alcohol[tiab] AND abstinence[tiab]) OR (("Alcohol Drinking"[mh] OR (Alcohol [tiab] AND drinking[tiab]) OR "Alcohol use"[tiab] OR "Alcohol consumption"[tiab] OR "Alcohol dependence"[tiab]) AND (Cease[tiab] OR Cessation[tiab] OR stop*[tiab] OR "giving up"[tiab] OR quit*[tiab] OR reduction[tiab] OR reduce[tiab] OR abstinen*[tiab] OR decreas*[tiab]))) AND (preop*[tw] OR "pre-op"[tw] OR Preoperative Care[mh] OR Prehabilit*[tw] OR Pre-habilit*[tw])) AND ("Clinical Study"[pt] OR "Comparative Study"[pt] OR "Epidemiologic studies" [mh] OR "Evaluation Study"[pt] OR "Meta-Analysis"[pt] OR "Multicenter Study"[pt] OR "Systematic Review"[pt] OR "Validation Study" [pt] OR randomized[tiab] OR analysis[tiab] OR study[tiab] OR studies [tiab] OR "Practice Guideline"[pt] OR "practice guidelines as topic"[mh] OR guideline[title] OR guidelines[title])) NOT (("animals"[MH:noexp] NOT "humans"[MH]) OR rat[tiab] OR rats[tiab] OR mouse[tiab] OR mice[tiab] OR dog[tiab] OR dogs[tiab] OR porcine[tiab] OR infant[mh] OR (child[mh] NOT adult[mh]) OR children[title] OR child[title] OR infant[title] OR infants[title] OR "Endometrial Neoplasms"[mh] OR "Ovarian Neoplasms"[mh] OR "Urinary Bladder Neoplasms"[mh] OR "Urogenital Neoplasms"[mh] OR "Whipple disease"[mh] OR "Whipple's disease"[tw] OR "Whipple disease"[tw] OR "Bladder cancer*"[tw] OR "Endometrial cancer*"[tw] OR "Ovarian cancer*"[tw] OR "Renal cancer*"[tw] OR Gynecologic[tiab] OR Urologic[tiab] OR Urothelial[tiab] OR Urethra[tiab] OR ("1905/01/01"[pdat]:"1999/12/31"[pdat])) | | 3 |
| Embase  KQ13  (22 Mar) | (('very elderly' OR 'frail elderly' OR elderly:kw,ti,ab OR 'septuagenarian*':kw,ti,ab OR 'octogenarian*':kw,ti,ab OR 'nonagenarian*':kw,ti,ab OR 'centenarian*':kw,ti,ab OR 'supercentenarian*':kw,ti,ab) AND ('colorectal surgery'/exp OR 'abdominal surgery'/exp OR 'abdominal surgery' OR 'rectum surgery'/exp OR 'rectum surgery' OR (('abdomen'/exp OR abdomen OR 'anal canal'/exp OR 'anal canal' OR 'anus'/exp OR anus OR 'colon'/exp OR colon OR 'rectum'/exp OR rectum OR 'intestine'/exp OR intestine:ti,ab OR 'sigmoid'/exp OR sigmoid) AND (resection:kw,ti,ab OR 'surgery'/exp OR surgery:kw,ti,ab OR surgical:kw,ti,ab)) OR 'colorectal surgery') AND (('preop*':ti,ab OR 'pre op':ti,ab OR 'prehabilit*':kw,ti,ab OR 'pre-habilit*':kw,ti,ab OR 'preoperative care'/exp) AND ('alcohol abstinence'/exp OR 'alcohol abstinence' OR (Alcohol:ti,ab AND abstinence:ti,ab) OR (('Alcohol use':ti,ab OR (Alcohol:ti,ab AND drinking:ti,ab) OR 'alcohol consumption'/exp OR 'alcohol consumption' OR 'Alcohol dependence':ti,ab OR 'alcoholism'/exp OR alcoholism) AND (Cease:ti,ab OR Cessation:ti,ab OR stop*:ti,ab OR 'giving up':ti,ab OR quit*:ti,ab OR reduction:ti,ab OR reduce:ti,ab OR abstinen*:ti,ab OR decreas*:ti,ab)) OR 'alcohol abstinence')) AND ('practice guideline'/exp OR 'practice guideline' OR [cochrane review]/lim OR [systematic review]/lim OR [meta analysis]/lim OR [clinical study]/lim OR 'clinical study'/exp OR 'case report' OR 'case study') AND [2000-2021]/py) NOT ([conference abstract]/lim OR [animals]/lim OR 'aged plant'/exp OR 'aged plant' OR 'child*':ti OR 'infant*':ti OR 'female genital tract tumor'/exp OR 'urogenital tract tumor'/exp OR (Whipple NEAR/3 disease) OR 'bladder cancer*':kw,ti,ab OR 'Endometrial cancer*':kw,ti,ab OR 'Ovarian cancer*':kw,ti,ab OR Gynecologic:kw,ti,ab OR Urologic:kw,ti,ab OR Urothelial:kw,ti,ab OR Urethra:kw,ti,ab) | | 3 |
| Cochrane  KQ13  (24 Mar) | ([MH Aged, 80 and over] OR [MH Frail Elderly] OR elderly:kw,ti,ab OR (septuagenarian*):kw,ti,ab OR (octogenarian*'):kw,ti,ab OR (nonagenarian*):kw,ti,ab OR (centenarian*):kw,ti,ab OR (supercentenarian*):kw,ti,ab) AND ([MH Colorectal Surgery] OR (abdominal surgery):kw,ti,ab OR (rectal surgery):kw,ti,ab OR (abdomen:ti,ab OR (anal canal):ti,ab OR (anus):ti,ab OR (colon):ti,ab OR (rectum):ti,ab OR (bowel):ti,ab OR (Sigmoid):ti,ab OR [MH anal canal] OR [MH colon] OR [MH rectum] ) AND ([MH surgical procedures, operative] OR resection:kw,ti,ab OR surgery:kw,ti,ab OR surgical:kw,ti,ab)) OR (colorectal surgery):kw,ti,ab) AND ([MH Alcohol Abstinence] OR ((Alcohol):ti,ab AND (abstinence):ti,ab) OR (((Alcohol):ti,ab AND drinking:ti,ab) OR (Alcohol use):ti,ab OR (alcohol consumption):ti,ab OR (Alcohol dependence):ti,ab OR (alcoholism):kw,ti,ab)) AND ((Cease):ti,ab OR (Cessation):ti,ab OR (stop*):ti,ab OR (giving up):ti,ab OR (quit*):ti,ab OR (reduc*):ti,ab OR (abstinen*):ti,ab OR (decreas*):ti,ab))) AND ((preop*):ti,ab OR (pre op):ti,ab OR (Prehabilit*):kw,ti,ab OR (Pre-habilit*):kw,ti,ab OR [MH Preoperative care]) NOT ([MH Endometrial Neoplasms] OR [MH Ovarian Neoplasms] OR [MH Urinary Bladder Neoplasms] OR [MH Urogenital Neoplasms"[mh] OR "Whipple disease] OR Gynecologic:kw,ti,ab OR Urologic:kw,ti,ab OR Urothelial:kw,ti,ab OR Urethra:kw,ti,ab) ; 1/1/2000-12/31/2021 | | 1 |
| ClinTrials  (31 Mar) | ("Colorectal Surgery" OR "Abdominal Surgery" OR "Rectal Surgery") AND ("Alcohol Cessation" OR "Alcohol Abstinence") \| Older Adult | | 1 |
| **KQ13**  **SEARCH RESULTS** | Total items identified by database searches | | 8 |
|  | Total trials identified in searches | | 2 |
|  | Total KQ13 duplicates removed during Topic 2 screening | | 6 |
|  | Total KQ13 records identified during Topic 2 screening | | 0 |
|  | Hand-searched items located after Topic 2 screening | | 0 |
|  | **Total items screened** | | **0** |

### KQ14

| **Q14: Should excess alcohol cessation (vs. no alcohol cessation) be applied in elderly patients undergoing UGI surgery?** | | | |
| --- | --- | --- | --- |
| **Search Concepts** | | **Elderly + UGI + Alcohol Cessation + Limits** | |
| **Database** | **Final search strategies** | | **Results** |
| PubMed  KQ14  (1 Apr) | (("Aged, 80 and over"[mh] OR "Frail Elderly"[mh] OR elderly[tiab] OR "older adult" [tw] OR septuagenar*[tw] OR octogenar*[tw] OR nonagenar*[tw] OR centenarian*[tw] OR supercentenar*[tw]) AND ((("Upper Gastrointestinal Tract" [mh] OR esophagus[mh] OR esophagus[tiab] OR stomach[mh] OR stomach[tiab] OR duodenum[mh] OR duodenum[tiab] OR Spleen[mh] OR Spleen[tiab]) AND ("surgical procedures, operative"[mh] OR "general surgery"[mh] OR surgery[sh] OR resection[tiab] OR surg*[tiab])) OR "Bariatric Surgery"[mh] OR Fundoplication[mh] OR Fundoplication[tiab] OR Gastrectomy[mh] OR Gastrect*[tiab] OR Oesophagect*[tiab] OR Esophagectomy[mh] OR Esophagect*[tiab] OR Splenectomy[mh] OR Splenect*[tiab] OR ((UGI[tiab] OR "Upper GI*" OR "gastro-esophageal"[tiab] OR "Upper Gastrointest*"[tiab]) AND Surger*[tiab])) AND (("Alcohol Abstinence"[mh] OR (Alcohol[tiab] AND abstinence[tiab]) OR (("Alcohol Drinking"[mh] OR (Alcohol [tiab] AND drinking[tiab]) OR "Alcohol use"[tiab] OR "Alcohol consumption"[tiab] OR "Alcohol dependence"[tiab]) AND (Cease[tiab] OR Cessation[tiab] OR stop*[tiab] OR "giving up"[tiab] OR quit*[tiab] OR reduction[tiab] OR reduce[tiab] OR abstinen*[tiab] OR decreas*[tiab]))) AND (preop*[tw] OR "pre-op"[tw] OR Preoperative Care[mh] OR Prehabilit*[tw] OR Pre-habilit*[tw])) AND ("Clinical Study"[pt] OR "Comparative Study"[pt] OR "Epidemiologic studies" [mh] OR "Evaluation Study"[pt] OR "Meta-Analysis"[pt] OR "Multicenter Study"[pt] OR "Systematic Review"[pt] OR "Validation Study" [pt] OR randomized[tiab] OR analysis[tiab] OR study[tiab] OR studies [tiab] OR "Practice Guideline"[pt] OR "practice guidelines as topic"[mh] OR guideline[title] OR guidelines[title])) NOT (("animals"[MH:noexp] NOT "humans"[MH]) OR rat[tiab] OR rats[tiab] OR mouse[tiab] OR mice[tiab] OR dog[tiab] OR dogs[tiab] OR porcine[tiab] OR infant[mh] OR (child[mh] NOT adult[mh]) OR children[title] OR child[title] OR infant[title] OR infants[title] OR "Endometrial Neoplasms"[mh] OR "Esophageal Neoplasms"[mh] OR "Ovarian Neoplasms"[mh] OR "Urinary Bladder Neoplasms"[mh] OR "Urogenital Neoplasms"[mh] OR "Whipple disease"[mh] OR "Whipple's disease"[tw] OR "Whipple disease"[tw] OR "Bladder cancer*"[tw] OR "Endometrial cancer*"[tw] OR "Ovarian cancer*"[tw] OR "Renal cancer*"[tw] OR Gynecologic[tiab] OR Urologic[tiab] OR Urothelial[tiab] OR Urethra[tiab] OR ("1905/01/01"[pdat]:"1999/12/31"[pdat])) | | 3 |
| Embase  KQ14  (22 Mar) | (('very elderly' OR 'frail elderly' OR elderly:kw,ti,ab OR 'septuagenarian*':kw,ti,ab OR 'octogenarian*':kw,ti,ab OR 'nonagenarian*':kw,ti,ab OR 'centenarian*':kw,ti,ab OR 'supercentenarian*':kw,ti,ab) AND ('Splenectomy'/exp OR (('upper gastrointestinal tract'/exp OR 'upper intestine*':kw,ti,ab OR 'esophagus'/exp OR esophagus:kw,ti,ab OR 'spleen'/exp OR spleen:kw,ti,ab OR 'stomach'/exp OR stomach:kw,ti,ab OR 'duodenum'/exp OR duodenum:kw,ti,ab OR 'upper gi*':kw,ti,ab OR ugi:ti,ab) AND (resection:kw,ti,ab OR 'surgery'/exp OR surgery:kw,ti,ab OR surgical:kw,ti,ab)) OR 'stomach fundoplication'/exp OR fundoplication:kw,ti,ab OR 'gastrectomy'/exp OR gastrectomy:kw,ti,ab OR 'gastrect*':kw,ti,ab OR oesophagectomy OR 'esophagus resection'/exp OR 'esophagus resection':kw,ti,ab OR esophagectomy:kw,ti,ab OR 'Splenect*':kw,ti,ab) AND (('preop*':ti,ab OR 'pre op':ti,ab OR 'prehabilit*':kw,ti,ab OR 'pre-habilit*':kw,ti,ab OR 'preoperative care'/exp) AND ('alcohol abstinence'/exp OR 'alcohol abstinence' OR (Alcohol:ti,ab AND abstinence:ti,ab) OR (('Alcohol use':ti,ab OR (Alcohol:ti,ab AND drinking:ti,ab) OR 'alcohol consumption'/exp OR 'alcohol consumption' OR 'Alcohol dependence':ti,ab OR 'alcoholism'/exp OR alcoholism) AND (Cease:ti,ab OR Cessation:ti,ab OR stop*:ti,ab OR 'giving up':ti,ab OR quit*:ti,ab OR reduction:ti,ab OR reduce:ti,ab OR abstinen*:ti,ab OR decreas*:ti,ab)) OR 'alcohol abstinence')) AND ('practice guideline'/exp OR 'practice guideline' OR [cochrane review]/lim OR [systematic review]/lim OR [meta analysis]/lim OR [clinical study]/lim OR 'clinical study'/exp OR 'case report' OR 'case study') AND [2000-2021]/py) NOT ([conference abstract]/lim OR [animals]/lim OR 'aged plant'/exp OR 'aged plant' OR 'child*':ti OR 'infant*':ti OR 'female genital tract tumor'/exp OR 'urogenital tract tumor'/exp OR 'esophagus tumor'/exp OR (Whipple NEAR/3 disease) OR 'bladder cancer*':kw,ti,ab OR 'Endometrial cancer*':kw,ti,ab OR 'Ovarian cancer*':kw,ti,ab OR Gynecologic:kw,ti,ab OR Urologic:kw,ti,ab OR Urothelial:kw,ti,ab OR Urethra:kw,ti,ab) | | 12 |
| Cochrane  KQ14  (24 Mar) | ([MH Aged, 80 and over] OR [MH Frail Elderly] OR elderly:kw,ti,ab OR (septuagenarian*):kw,ti,ab OR (octogenarian*'):kw,ti,ab OR (nonagenarian*):kw,ti,ab OR (centenarian*):kw,ti,ab OR (supercentenarian*):kw,ti,ab) AND ([MH Splenectomy] OR (([MH upper gastrointestinal tract] OR (upper intestine*):kw,ti,ab OR OR [MH esophagus] OR [MH stomach] OR [MH duodenum] OR [MH spleen] OR esophagus:ti,ab OR OR stomach:ti,ab OR duodenum:ti,ab OR spleen:ti,ab OR 'upper gi*':kw,ti,ab OR ugi:kw,ti,ab OR (gastro-esophageal):ti,ab) AND (resection:kw,ti,ab OR [MH surgical procedures, operative] OR surgery:kw,ti,ab OR surgical:kw,ti,ab)) OR [MH Fundoplication] OR fundoplication:kw,ti,ab OR [MH Gastrectomy] OR gastrectomy:kw,ti,ab OR (gastrect*):kw,ti,ab OR oesophagectomy OR (Splenect*):kw,ti,ab) AND ([MH Alcohol Abstinence] OR ((Alcohol):ti,ab AND (abstinence):ti,ab) OR (((Alcohol):ti,ab AND drinking:ti,ab) OR (Alcohol use):ti,ab OR (alcohol consumption):ti,ab OR (Alcohol dependence):ti,ab OR (alcoholism):kw,ti,ab)) AND ((Cease):ti,ab OR (Cessation):ti,ab OR (stop*):ti,ab OR (giving up):ti,ab OR (quit*):ti,ab OR (reduc*):ti,ab OR (abstinen*):ti,ab OR (decreas*):ti,ab))) AND ((preop*):ti,ab OR (pre op):ti,ab OR (Prehabilit*):kw,ti,ab OR (Pre-habilit*):kw,ti,ab OR [MH Preoperative care]) NOT ([MH Endometrial Neoplasms] OR [MH Ovarian Neoplasms] OR [MH Urinary Bladder Neoplasms] OR [MH Urogenital Neoplasms"[mh] OR "Whipple disease] OR Gynecologic:kw,ti,ab OR Urologic:kw,ti,ab OR Urothelial:kw,ti,ab OR Urethra:kw,ti,ab) ; 1/1/2000-12/31/2021 | | 0 |
| ClinTrials KQ14  (31 Mar) | ("Bariatric Surgery" OR Fundoplication OR Splenectomy OR pancreatectomy OR Gastrectomy OR (("Upper Gastrointestinal" OR "upper gI") AND surgery)) AND ("Alcohol Cessation" OR "Alcohol Abstinence") \| Older Adult | | 0 |
| **KQ14**  **SEARCH RESULTS** | Total items identified by database searches | | 8 |
|  | Total trials identified in searches | | 2 |
|  | Total KQ14 duplicates removed during Topic 2 screening | | 6 |
|  | Total KQ14 records identified during Topic 2 screening | | 0 |
|  | Hand-searched items located after Topic 2 screening | | 0 |
|  | **Total items screened** | | **0** |

### KQ15

| **Q15: Should excess alcohol cessation (vs. no alcohol cessation) be applied in elderly patients undergoing HPB surgery?** | | | |
| --- | --- | --- | --- |
| **Search Concepts** | | **Elderly + HPB + Alcohol Cessation + Limits** | |
| **Database** | **Final search strategies** | | **Results** |
| PubMed  KQ15  (22 Mar) | (("Aged, 80 and over"[mh] OR "Frail Elderly"[mh] OR elderly[tiab] OR "older adult" [tw] OR septuagenar*[tw] OR octogenar*[tw] OR nonagenar*[tw] OR centenarian*[tw] OR supercentenar*[tw]) AND ("Hepato-pancreato-biliary"[tiab] OR HPB[tiab] OR hepatopancreaticobiliary[tiab] OR Whipple[tiab] OR Whipple [ot] OR ((liver[mh] OR liver[tiab] OR pancreas [mh] OR pancreas[tiab] OR gallbladder [mh] OR gallbladder [tiab] OR "bile ducts"[mh] OR "bile Duct*"[tiab] OR jejunum[mh] OR jejunum[tiab]) AND ("surgical procedures, operative"[mh] OR "general surgery"[mh] OR surgery[sh] OR surg*[tiab] OR resection[tiab])) OR Hepatectomy[mh] OR Hepatect*[tiab] OR pancreatectomy[mh] OR pancreatect*[tiab] OR Hepatojejunost*[tiab] OR ((Renal[tiab] OR biliary[tiab]) AND surger*[tiab])) AND (("Alcohol Abstinence"[mh] OR (Alcohol[tiab] AND abstinence[tiab]) OR (("Alcohol Drinking"[mh] OR (Alcohol [tiab] AND drinking[tiab]) OR "Alcohol use"[tiab] OR "Alcohol consumption"[tiab] OR "Alcohol dependence"[tiab]) AND (Cease[tiab] OR Cessation[tiab] OR stop*[tiab] OR "giving up"[tiab] OR quit*[tiab] OR reduction[tiab] OR reduce[tiab] OR abstinen*[tiab] OR decreas*[tiab]))) AND (preop*[tw] OR "pre-op"[tw] OR Preoperative Care[mh] OR Prehabilit*[tw] OR Pre-habilit*[tw])) AND ("Clinical Study"[pt] OR "Comparative Study"[pt] OR "Epidemiologic studies" [mh] OR "Evaluation Study"[pt] OR "Meta-Analysis"[pt] OR "Multicenter Study"[pt] OR "Systematic Review"[pt] OR "Validation Study" [pt] OR randomized[tiab] OR analysis[tiab] OR study[tiab] OR studies [tiab] OR "Practice Guideline"[pt] OR "practice guidelines as topic"[mh] OR guideline[title] OR guidelines[title])) NOT (("animals"[MH:noexp] NOT "humans"[MH]) OR rat[tiab] OR rats[tiab] OR mouse[tiab] OR mice[tiab] OR dog[tiab] OR dogs[tiab] OR porcine[tiab] OR infant[mh] OR (child[mh] NOT adult[mh]) OR children[title] OR child[title] OR infant[title] OR infants[title] OR "Endometrial Neoplasms"[mh] OR "Ovarian Neoplasms"[mh] OR "Urinary Bladder Neoplasms"[mh] OR "Urogenital Neoplasms"[mh] OR "Whipple disease"[mh] OR "Whipple's disease"[tw] OR "Whipple disease"[tw] OR "Bladder cancer*"[tw] OR "Endometrial cancer*"[tw] OR "Ovarian cancer*"[tw] OR "Renal cancer*"[tw] OR Gynecologic[tiab] OR Urologic[tiab] OR Urothelial[tiab] OR Urethra[tiab] OR ("1905/01/01"[pdat]:"1999/12/31"[pdat])) | | 5 |
| Embase  KQ15  (22 Mar) | (('very elderly' OR 'frail elderly' OR elderly:kw,ti,ab OR 'septuagenarian*':kw,ti,ab OR 'octogenarian*':kw,ti,ab OR 'nonagenarian*':kw,ti,ab OR 'centenarian*':kw,ti,ab OR 'supercentenarian*':kw,ti,ab) AND ('biliary surgery' OR (('liver'/exp OR liver:kw,ti,ab OR 'pancreas'/exp OR pancreas:kw,ti,ab OR 'gallbladder'/exp OR gallbladder:kw,ti,ab OR 'bile duct'/exp OR 'bile duct' OR 'bile ducts' OR 'jejunum'/exp OR jejunum:kw,ti,ab) AND (resection:kw,ti,ab OR 'surgery'/exp OR surgery:kw,ti,ab OR surgical:kw,ti,ab)) OR 'liver surgery'/exp OR 'liver surgery' OR 'liver resection'/exp OR 'liver resection' OR 'pancreatectomy'/exp OR pancreatectomy OR 'hepatect*':kw,ti,ab OR 'nephrect*':kw,ti,ab OR 'hepatojejunostomy'/exp OR hepatojejunostomy:kw,ti,ab OR 'hepatopancreaticobiliary surgery' OR 'hepato pancreato biliary' OR hepatopancreaticobiliary OR HPB:kw,ti,ab OR 'biliary tract surgery'/exp OR 'biliary tract surgery' OR 'Renal surgery') AND (('preop*':ti,ab OR 'pre op':ti,ab OR 'prehabilit*':kw,ti,ab OR 'pre-habilit*':kw,ti,ab OR 'preoperative care'/exp) AND ('alcohol abstinence'/exp OR 'alcohol abstinence' OR (Alcohol:ti,ab AND abstinence:ti,ab) OR (('Alcohol use':ti,ab OR (Alcohol:ti,ab AND drinking:ti,ab) OR 'alcohol consumption'/exp OR 'alcohol consumption' OR 'Alcohol dependence':ti,ab OR 'alcoholism'/exp OR alcoholism) AND (Cease:ti,ab OR Cessation:ti,ab OR stop*:ti,ab OR 'giving up':ti,ab OR quit*:ti,ab OR reduction:ti,ab OR reduce:ti,ab OR abstinen*:ti,ab OR decreas*:ti,ab)) OR 'alcohol abstinence')) AND ('practice guideline'/exp OR 'practice guideline' OR [cochrane review]/lim OR [systematic review]/lim OR [meta analysis]/lim OR [clinical study]/lim OR 'clinical study'/exp OR 'case report' OR 'case study') AND [2000-2021]/py) NOT ([conference abstract]/lim OR [animals]/lim OR 'aged plant'/exp OR 'aged plant' OR 'child*':ti OR 'infant*':ti OR 'female genital tract tumor'/exp OR 'urogenital tract tumor'/exp OR (Whipple NEAR/3 disease) OR 'bladder cancer*':kw,ti,ab OR 'Endometrial cancer*':kw,ti,ab OR 'Ovarian cancer*':kw,ti,ab OR Gynecologic:kw,ti,ab OR Urologic:kw,ti,ab OR Urothelial:kw,ti,ab OR Urethra:kw,ti,ab) | | 1 |
| Cochrane  KQ15  (24 Mar) | ([MH Aged, 80 and over] OR [MH Frail Elderly] OR elderly:kw,ti,ab OR (septuagenarian*):kw,ti,ab OR (octogenarian*'):kw,ti,ab OR (nonagenarian*):kw,ti,ab OR (centenarian*):kw,ti,ab OR (supercentenarian*):kw,ti,ab) AND ([MH Hepatectomy] OR [MH pancreatectomy] OR (pancreatect*):ti,ab OR (Hepatojejunost*):ti,ab OR (Renal surger*):ti,ab OR (biliary surger*):ti,ab OR (Hepato-pancreato-biliary):ti,ab OR (HPB):ti,ab OR hepatopancreaticobiliary:ti,ab OR (([MH liver] OR (liver):ti,ab OR [MH pancreas] OR (pancreas):ti,ab OR [MH gallbladder] OR (gallbladder):ti,ab OR [MH bile ducts] OR (bile Duct*):ti,ab OR [MH kidney] OR (kidney*):ti,ab OR [MH jejunum] OR (jejunum):ti,ab) AND ([MH surgical procedures, operative] OR (resection):ti,ab,kw OR (surger*):ti,ab,kw)) OR (Hepatect*):ti,ab) AND ([MH Alcohol Abstinence] OR ((Alcohol):ti,ab AND (abstinence):ti,ab) OR (((Alcohol):ti,ab AND drinking:ti,ab) OR (Alcohol use):ti,ab OR (alcohol consumption):ti,ab OR (Alcohol dependence):ti,ab OR (alcoholism):kw,ti,ab)) AND ((Cease):ti,ab OR (Cessation):ti,ab OR (stop*):ti,ab OR (giving up):ti,ab OR (quit*):ti,ab OR (reduc*):ti,ab OR (abstinen*):ti,ab OR (decreas*):ti,ab))) AND ((preop*):ti,ab OR (pre op):ti,ab OR (Prehabilit*):kw,ti,ab OR (Pre-habilit*):kw,ti,ab OR [MH Preoperative care]) NOT ([MH Endometrial Neoplasms] OR [MH Ovarian Neoplasms] OR [MH Urinary Bladder Neoplasms] OR [MH Urogenital Neoplasms"[mh] OR "Whipple disease] OR Gynecologic:kw,ti,ab OR Urologic:kw,ti,ab OR Urothelial:kw,ti,ab OR Urethra:kw,ti,ab) ; 1/1/2000-12/31/2021 | | 0 |
| ClinTrials  (31 Mar) | ("Hepato-pancreato-biliary" OR hepatopancreaticobiliary OR Hepatectomy OR pancreatectomy) AND ("Alcohol Cessation" OR "Alcohol Abstinence") \| Older Adult | | 0 |
| **KQ15**  **SEARCH RESULTS** | Total items identified by database searches | | 6 |
|  | Total trials identified in searches | | 0 |
|  | Total KQ15 duplicates removed during Topic 2 screening | | 6 |
|  | Total KQ15 records identified during Topic 2 screening | | 0 |
|  | Hand-searched items located after Topic 2 screening | | 0 |
|  | **Total items screened** | | **0** |

### KQ16

| **Q16: Should excess alcohol cessation (vs. no alcohol cessation) be applied in elderly patients undergoing hernia surgery?** | | | |
| --- | --- | --- | --- |
| **Search Concepts** | | **Elderly + Hernia + Alcohol Cessation + Limits** | |
| **Database** | **Final search strategies** | | **Results** |
| PubMed  KQ16  (22 Mar) | (("Aged, 80 and over"[mh] OR "Frail Elderly"[mh] OR elderly[tiab] OR "older adult" [tw] OR septuagenar*[tw] OR octogenar*[tw] OR nonagenar*[tw] OR centenarian*[tw] OR supercentenar*[tw]) AND (((Hernia[tiab] OR Hernia[mh]) AND ("surgical procedures, operative"[mh] OR "general surgery"[mh] OR surgery[sh] OR surg*[tiab])) OR Herniorrhaphy[mh] OR Herniorrhap*[tiab]) AND ((preop*[tiab] OR "pre-op"[tiab] OR Preoperative Care[mh] OR Prehabilit*[tw] OR Pre-habilit*[tw]) AND ("Alcohol Abstinence"[mh] OR (Alcohol[tiab] AND abstinence[tiab]) OR (("Alcohol Drinking"[mh] OR (Alcohol [tiab] AND drinking[tiab]) OR "Alcohol use"[tiab] OR "Alcohol consumption"[tiab] OR "Alcohol dependence"[tiab]) AND (Cease[tiab] OR Cessation[tiab] OR stop*[tiab] OR "giving up"[tiab] OR quit*[tiab] OR reduction[tiab] OR reduce[tiab] OR abstinen*[tiab] OR decreas*[tiab])))) AND ("Clinical Study"[pt] OR "Comparative Study"[pt] OR "Epidemiologic studies" [mh] OR "Evaluation Study"[pt] OR "Meta-Analysis"[pt] OR "Multicenter Study"[pt] OR "Systematic Review"[pt] OR "Validation Study" [pt] OR randomized[tiab] OR analysis[tiab] OR study[tiab] OR studies [tiab] OR "Practice Guideline"[pt] OR "practice guidelines as topic"[mh] OR guideline[title] OR guidelines[title])) NOT (("animals"[MH:noexp] NOT "humans"[MH]) OR rat[tiab] OR rats[tiab] OR mouse[tiab] OR mice[tiab] OR dog[tiab] OR dogs[tiab] OR porcine[tiab] OR infant[mh] OR (child[mh] NOT adult[mh]) OR children[title] OR child[title] OR infant[title] OR infants[title] OR "Endometrial Neoplasms"[mh] OR "Ovarian Neoplasms"[mh] OR "Urinary Bladder Neoplasms"[mh] OR "Urogenital Neoplasms"[mh] OR "Whipple disease"[mh] OR "Whipple's disease"[tw] OR "Whipple disease"[tw] OR "Bladder cancer*"[tw] OR "Endometrial cancer*"[tw] OR "Ovarian cancer*"[tw] OR "Renal cancer*"[tw] OR Gynecologic[tiab] OR Urologic[tiab] OR Urothelial[tiab] OR Urethra[tiab] OR ("1905/01/01"[pdat]:"1999/12/31"[pdat])) | | 1 |
| Embase  KQ16  (22 Mar) | (('very elderly' OR 'frail elderly' OR elderly:kw,ti,ab OR 'septuagenarian*':kw,ti,ab OR 'octogenarian*':kw,ti,ab OR 'nonagenarian*':kw,ti,ab OR 'centenarian*':kw,ti,ab OR 'supercentenarian*':kw,ti,ab) AND ('hernia surgery'/exp OR 'herniorrhaphy'/exp OR 'herniorrhap*':kw,ti,ab OR (('hernia'/exp OR 'hernia':kw,ti,ab) AND ('surgery'/exp OR surgery:kw,ti,ab OR surgical:kw,ti,ab)) OR 'hernia surgery') AND (('preop*':ti,ab OR 'pre op':ti,ab OR 'prehabilit*':kw,ti,ab OR 'pre-habilit*':kw,ti,ab OR 'preoperative care'/exp) AND ('alcohol abstinence'/exp OR 'alcohol abstinence' OR (Alcohol:ti,ab AND abstinence:ti,ab) OR (('Alcohol use':ti,ab OR (Alcohol:ti,ab AND drinking:ti,ab) OR 'alcohol consumption'/exp OR 'alcohol consumption' OR 'Alcohol dependence':ti,ab OR 'alcoholism'/exp OR alcoholism) AND (Cease:ti,ab OR Cessation:ti,ab OR stop*:ti,ab OR 'giving up':ti,ab OR quit*:ti,ab OR reduction:ti,ab OR reduce:ti,ab OR abstinen*:ti,ab OR decreas*:ti,ab)) OR 'alcohol abstinence')) AND ('practice guideline'/exp OR 'practice guideline' OR [cochrane review]/lim OR [systematic review]/lim OR [meta analysis]/lim OR [clinical study]/lim OR 'clinical study'/exp OR 'case report' OR 'case study') AND [2000-2021]/py) NOT ([conference abstract]/lim OR [animals]/lim OR 'aged plant'/exp OR 'aged plant' OR 'child*':ti OR 'infant*':ti OR 'female genital tract tumor'/exp OR 'urogenital tract tumor'/exp OR (Whipple NEAR/3 disease) OR 'bladder cancer*':kw,ti,ab OR 'Endometrial cancer*':kw,ti,ab OR 'Ovarian cancer*':kw,ti,ab OR Gynecologic:kw,ti,ab OR Urologic:kw,ti,ab OR Urothelial:kw,ti,ab OR Urethra:kw,ti,ab) | | 0 |
| Cochrane  KQ16  (24 Mar) | ([MH Aged, 80 and over] OR [MH Frail Elderly] OR elderly:kw,ti,ab OR (septuagenarian*):kw,ti,ab OR (octogenarian*'):kw,ti,ab OR (nonagenarian*):kw,ti,ab OR (centenarian*):kw,ti,ab OR (supercentenarian*):kw,ti,ab) AND ([MH Herniorrhaphy] OR (((Hernia):ti,ab,kw OR [MH Hernia]) AND ([MH surgical procedures, operative] OR surgery:kw,ti,ab OR surgical:kw,ti,ab)) OR (Herniorrhap*):ti,ab,kw) AND ([MH Alcohol Abstinence] OR ((Alcohol):ti,ab AND (abstinence):ti,ab) OR (((Alcohol):ti,ab AND drinking:ti,ab) OR (Alcohol use):ti,ab OR (alcohol consumption):ti,ab OR (Alcohol dependence):ti,ab OR (alcoholism):kw,ti,ab)) AND ((Cease):ti,ab OR (Cessation):ti,ab OR (stop*):ti,ab OR (giving up):ti,ab OR (quit*):ti,ab OR (reduc*):ti,ab OR (abstinen*):ti,ab OR (decreas*):ti,ab))) AND ((preop*):ti,ab OR (pre op):ti,ab OR (Prehabilit*):kw,ti,ab OR (Pre-habilit*):kw,ti,ab OR [MH Preoperative care]) NOT ([MH Endometrial Neoplasms] OR [MH Ovarian Neoplasms] OR [MH Urinary Bladder Neoplasms] OR [MH Urogenital Neoplasms"[mh] OR "Whipple disease] OR Gynecologic:kw,ti,ab OR Urologic:kw,ti,ab OR Urothelial:kw,ti,ab OR Urethra:kw,ti,ab) ; 1/1/2000-12/31/2021 | | 0 |
| ClinTrials  (31 Mar) | ("Herniorrhaphy" OR (Hernia AND Surgery)) AND ("Alcohol Cessation" OR "Alcohol Abstinence") \| Older Adult | | 0 |
| **KQ16**  **SEARCH RESULTS** | Total items identified by database searches | | 1 |
|  | Total trials identified in searches | | 0 |
|  | Total KQ16 duplicates removed during Topic 2 screening | | 1 |
|  | Total KQ16 records identified during Topic 2 screening | | 0 |
|  | Hand-searched items located after Topic 2 screening | | 0 |
|  | **Total items screened** | | **0** |

## Topic 3

| **Topic 3: Role of MIS in the elderly**  (KQ1-KQ12 combined in this first screening) | | |
| --- | --- | --- |
| **Topic 3**  **Search**  **Results** | Total items identified by database searches | 13399 |
|  | Additional articles located by handsearching | 138 |
|  | Total trials identified | 267 |
|  | Total duplicates | 5480 |
|  | Total items screened | 7790 |
|  | Records excluded | 6924 |
|  | Unique items to be split between KQ17-KQ20 | 866 |

### KQ17

| **Q17: Should laparoscopic (vs open) colorectal surgery be used in the elderly?** | | | |
| --- | --- | --- | --- |
| **Search Concepts** | | **Elderly + Colorectal + MIS + Limits** | |
| **Database** | **Final search strategies** | | **Results** |
| PubMed  KQ17  (22 Mar) | (("Aged, 80 and over"[mh] OR "Frail Elderly"[mh] OR elderly[tiab] OR "older adult" [tw] OR septuagenar*[tw] OR octogenar*[tw] OR nonagenar*[tw] OR centenarian*[tw] OR supercentenar*[tw]) AND ("Colorectal Surgery"[mh] OR ((abdomen[tiab] OR "anal canal"[tiab] OR "anal canal"[mh] OR anus[tiab] OR bowel[tiab] OR colon[tiab] OR colon [mh] OR rectum[tiab] OR rectum[mh] OR "Colon, Sigmoid"[mh] OR Sigmoid[tiab]) AND ("surgical procedures, operative"[mh] OR "general surgery"[mh] OR surgery[sh] OR resection[tiab] OR surg*[tiab])) OR ((rectal [tiab] OR Colorectal[tiab] OR abdominal[tiab]) AND Surger*[tiab])) AND (Laparoscopes[mh] OR Laparoscopy[mh] OR Laparoscop*[tiab] OR Laparoscop*[ot] OR "Heller myotomy"[mh] OR "Heller myotom*"[tiab] OR Cardiomyotom*[tw] OR "Robotic Surgical Procedures"[mh] OR ((Robot*[tiab] OR Robot*[ot]) AND (surgery[sh] OR surg*[tiab] OR "Surgical Procedures, Operative"[mh]))) AND ("Clinical Study"[pt] OR "Comparative Study"[pt] OR "Epidemiologic studies" [mh] OR "Evaluation Study"[pt] OR "Meta-Analysis"[pt] OR "Multicenter Study"[pt] OR "Systematic Review"[pt] OR "Validation Study" [pt] OR randomized[tiab] OR analysis[tiab] OR study[tiab] OR studies [tiab] OR "Practice Guideline"[pt] OR "practice guidelines as topic"[mh] OR guideline[title] OR guidelines[title])) NOT ("Case Reports"[pt] OR "case report*"[tiab] OR "case stud*"[tiab] OR ("animals"[MH:noexp] NOT "humans"[MH]) OR rat[tiab] OR rats[tiab] OR mouse[tiab] OR mice[tiab] OR dog[tiab] OR dogs[tiab] OR porcine[tiab] OR infant[mh] OR (child[mh] NOT adult[mh]) OR children[title] OR child[title] OR infant[title] OR infants[title] OR "Endometrial Neoplasms"[mh] OR "Ovarian Neoplasms"[mh] OR "Urinary Bladder Neoplasms"[mh] OR "Urogenital Neoplasms"[mh] OR "Whipple disease"[mh] OR "Whipple's disease"[tw] OR "Whipple disease"[tw] OR "Bladder cancer*"[tw] OR "Endometrial cancer*"[tw] OR "Ovarian cancer*"[tw] OR "Renal cancer*"[tw] OR Gynecologic[tiab] OR Urologic[tiab] OR Urothelial[tiab] OR Urethra[tiab] OR ("1905/01/01"[pdat]:"1999/12/31"[pdat])) | | 3,242 |
| Embase  KQ17  (22 Mar) | (('very elderly' OR 'frail elderly' OR elderly:kw,ti,ab OR 'septuagenarian*':kw,ti,ab OR 'octogenarian*':kw,ti,ab OR 'nonagenarian*':kw,ti,ab OR 'centenarian*':kw,ti,ab OR 'supercentenarian*':kw,ti,ab) AND ('colorectal surgery'/exp OR 'abdominal surgery'/exp OR 'abdominal surgery' OR 'rectum surgery'/exp OR 'rectum surgery' OR (('abdomen'/exp OR abdomen OR 'anal canal'/exp OR 'anal canal' OR 'anus'/exp OR anus OR 'colon'/exp OR colon OR 'rectum'/exp OR rectum OR 'intestine'/exp OR intestine:ti,ab OR 'sigmoid'/exp OR sigmoid) AND (resection:kw,ti,ab OR 'surgery'/exp OR surgery:kw,ti,ab OR surgical:kw,ti,ab)) OR 'colorectal surgery') AND ('laparoscope'/exp OR laparoscope OR 'laparoscopy'/exp OR laparoscop*:kw,ti,ab OR 'cardioesophagomyotomy'/exp OR cardioesophagomyotomy OR 'Heller myotomy' OR 'robot assisted surgery'/exp OR 'robot assisted surgery' OR (Robot*:kw,ti,ab AND ('surgery'/exp OR surgery:kw,ti,ab OR surgical:kw,ti,ab)) OR laparoscope) AND ('practice guideline'/exp OR 'practice guideline' OR [cochrane review]/lim OR [systematic review]/lim OR [meta analysis]/lim OR [clinical study]/lim OR 'clinical study'/exp) AND [2000-2021]/py) NOT ( 'case study'/exp OR 'case report' OR 'case study' OR [conference abstract]/lim OR [animals]/lim OR 'aged plant'/exp OR 'aged plant' OR 'child*':ti OR 'infant*':ti OR 'female genital tract tumor'/exp OR 'urogenital tract tumor'/exp OR (Whipple NEAR/3 disease) OR 'bladder cancer*':kw,ti,ab OR 'Endometrial cancer*':kw,ti,ab OR 'Ovarian cancer*':kw,ti,ab OR Gynecologic:kw,ti,ab OR Urologic:kw,ti,ab OR Urothelial:kw,ti,ab OR Urethra:kw,ti,ab) | | 2,824 |
| Cochrane  KQ17  (24 Mar) | ([MH Aged, 80 and over] OR [MH Frail Elderly] OR elderly:kw,ti,ab OR (septuagenarian*):kw,ti,ab OR (octogenarian*'):kw,ti,ab OR (nonagenarian*):kw,ti,ab OR (centenarian*):kw,ti,ab OR (supercentenarian*):kw,ti,ab) AND ([MH Colorectal Surgery] OR (abdominal surgery):kw,ti,ab OR (rectal surgery):kw,ti,ab OR (abdomen:ti,ab OR (anal canal):ti,ab OR (anus):ti,ab OR (colon):ti,ab OR (rectum):ti,ab OR (bowel):ti,ab OR (Sigmoid):ti,ab OR [MH anal canal] OR [MH colon] OR [MH rectum] ) AND ([MH surgical procedures, operative] OR resection:kw,ti,ab OR surgery:kw,ti,ab OR surgical:kw,ti,ab)) OR (colorectal surgery):kw,ti,ab) AND ([MH Laparoscopes] OR [MH Laparoscopy] OR [MH Heller myotomy] OR (Heller myotomy):ti,ab,kw OR [MH Robotic Surgical Procedures] OR ((Robot*):ti,ab,kw AND ([MH surgical procedures, operative] OR surgery:kw,ti,ab OR surgical:kw,ti,ab)) OR (Laparoscop*):ti,ab,kw ) NOT ([MH Endometrial Neoplasms] OR [MH Ovarian Neoplasms] OR [MH Urinary Bladder Neoplasms] OR [MH Urogenital Neoplasms"[mh] OR "Whipple disease] OR Gynecologic:kw,ti,ab OR Urologic:kw,ti,ab OR Urothelial:kw,ti,ab OR Urethra:kw,ti,ab) ; 1/1/2000-12/31/2021 | | 371 |
| ClinTrials  (31 Mar) | ("Colorectal Surgery" OR "Abdominal Surgery" OR "Rectal Surgery") AND (laparoscopic OR laparoscopy OR robot) \| Older Adult | | 38 |
| **KQ17**  **SEARCH RESULTS** | Total items identified by database searches | | 6,475 |
|  | Total trials identified in searches | | 98 |
|  | Total KQ17 duplicates removed during Topic 3 screening | | 6,030 |
|  | Total KQ17 records identified during Topic 3 screening | | 347 |
|  | Hand-searched items located after Topic 3 screening | | 2 |
|  | **Total items screened** | | **349** |

### KQ18

| **Q18: Should laparoscopic (vs open) Upper GI surgery be used in the elderly?** | | | |
| --- | --- | --- | --- |
| **Search Concepts** | | **Elderly + UGI + MIS + Limits** | |
| **Database** | **Final search strategies** | | **Results** |
| PubMed  KQ18  (1 Apr) | (("Aged, 80 and over"[mh] OR "Frail Elderly"[mh] OR elderly[tiab] OR "older adult" [tw] OR septuagenar*[tw] OR octogenar*[tw] OR nonagenar*[tw] OR centenarian*[tw] OR supercentenar*[tw]) AND ((("Upper Gastrointestinal Tract" [mh] OR esophagus[mh] OR esophagus[tiab] OR stomach[mh] OR stomach[tiab] OR duodenum[mh] OR duodenum[tiab] OR Spleen[mh] OR Spleen[tiab]) AND ("surgical procedures, operative"[mh] OR "general surgery"[mh] OR surgery[sh] OR resection[tiab] OR surg*[tiab])) OR "Bariatric Surgery"[mh] OR Fundoplication[mh] OR Fundoplication[tiab] OR Gastrectomy[mh] OR Gastrect*[tiab] OR Oesophagect*[tiab] OR Esophagectomy[mh] OR Esophagect*[tiab] OR Splenectomy[mh] OR Splenect*[tiab] OR ((UGI[tiab] OR "Upper GI*" OR "gastro-esophageal"[tiab] OR "Upper Gastrointest*"[tiab]) AND Surger*[tiab])) AND (Laparoscopes[mh] OR Laparoscopy[mh] OR Laparoscop*[tiab] OR Laparoscop*[ot] OR "Heller myotomy"[mh] OR "Heller myotom*"[tiab] OR Cardiomyotom*[tw] OR "Robotic Surgical Procedures"[mh] OR ((Robot*[tiab] OR Robot*[ot]) AND (surgery[sh] OR surg*[tiab] OR "Surgical Procedures, Operative"[mh]))) AND ("Clinical Study"[pt] OR "Comparative Study"[pt] OR "Epidemiologic studies" [mh] OR "Evaluation Study"[pt] OR "Meta-Analysis"[pt] OR "Multicenter Study"[pt] OR "Systematic Review"[pt] OR "Validation Study" [pt] OR randomized[tiab] OR analysis[tiab] OR study[tiab] OR studies [tiab] OR "Practice Guideline"[pt] OR "practice guidelines as topic"[mh] OR guideline[title] OR guidelines[title])) NOT ("Case Reports"[pt] OR "case report*"[tiab] OR "case stud*"[tiab] OR ("animals"[MH:noexp] NOT "humans"[MH]) OR rat[tiab] OR rats[tiab] OR mouse[tiab] OR mice[tiab] OR dog[tiab] OR dogs[tiab] OR porcine[tiab] OR infant[mh] OR (child[mh] NOT adult[mh]) OR children[title] OR child[title] OR infant[title] OR infants[title] OR "Endometrial Neoplasms"[mh] OR "Esophageal Neoplasms"[mh] OR "Ovarian Neoplasms"[mh] OR "Urinary Bladder Neoplasms"[mh] OR "Urogenital Neoplasms"[mh] OR "Whipple disease"[mh] OR "Whipple's disease"[tw] OR "Whipple disease"[tw] OR "Bladder cancer*"[tw] OR "Endometrial cancer*"[tw] OR "Ovarian cancer*"[tw] OR "Renal cancer*"[tw] OR Gynecologic[tiab] OR Urologic[tiab] OR Urothelial[tiab] OR Urethra[tiab] OR ("1905/01/01"[pdat]:"1999/12/31"[pdat])) | | 1,557 |
| Embase  KQ18  (22 Mar) | (('very elderly' OR 'frail elderly' OR elderly:kw,ti,ab OR 'septuagenarian*':kw,ti,ab OR 'octogenarian*':kw,ti,ab OR 'nonagenarian*':kw,ti,ab OR 'centenarian*':kw,ti,ab OR 'supercentenarian*':kw,ti,ab) AND ('Splenectomy'/exp OR (('upper gastrointestinal tract'/exp OR 'upper intestine*':kw,ti,ab OR 'esophagus'/exp OR esophagus:kw,ti,ab OR 'spleen'/exp OR spleen:kw,ti,ab OR 'stomach'/exp OR stomach:kw,ti,ab OR 'duodenum'/exp OR duodenum:kw,ti,ab OR 'upper gi*':kw,ti,ab OR ugi:ti,ab) AND (resection:kw,ti,ab OR 'surgery'/exp OR surgery:kw,ti,ab OR surgical:kw,ti,ab)) OR 'stomach fundoplication'/exp OR fundoplication:kw,ti,ab OR 'gastrectomy'/exp OR gastrectomy:kw,ti,ab OR 'gastrect*':kw,ti,ab OR oesophagectomy OR 'esophagus resection'/exp OR 'esophagus resection':kw,ti,ab OR esophagectomy:kw,ti,ab OR 'Splenect*':kw,ti,ab) AND ('laparoscope'/exp OR laparoscope OR 'laparoscopy'/exp OR laparoscop*:kw,ti,ab OR 'cardioesophagomyotomy'/exp OR cardioesophagomyotomy OR 'Heller myotomy' OR 'robot assisted surgery'/exp OR 'robot assisted surgery' OR (Robot*:kw,ti,ab AND ('surgery'/exp OR surgery:kw,ti,ab OR surgical:kw,ti,ab)) OR laparoscope) AND ('practice guideline'/exp OR 'practice guideline' OR [cochrane review]/lim OR [systematic review]/lim OR [meta analysis]/lim OR [clinical study]/lim OR 'clinical study'/exp) AND [2000-2021]/py) NOT ( 'case study'/exp OR 'case report' OR 'case study' OR [conference abstract]/lim OR [animals]/lim OR 'aged plant'/exp OR 'aged plant' OR 'child*':ti OR 'infant*':ti OR 'female genital tract tumor'/exp OR 'urogenital tract tumor'/exp OR 'esophagus tumor'/exp OR (Whipple NEAR/3 disease) OR 'bladder cancer*':kw,ti,ab OR 'Endometrial cancer*':kw,ti,ab OR 'Ovarian cancer*':kw,ti,ab OR Gynecologic:kw,ti,ab OR Urologic:kw,ti,ab OR Urothelial:kw,ti,ab OR Urethra:kw,ti,ab) | | 575 |
| Cochrane  KQ18  (24 Mar) | ([MH Aged, 80 and over] OR [MH Frail Elderly] OR elderly:kw,ti,ab OR (septuagenarian*):kw,ti,ab OR (octogenarian*'):kw,ti,ab OR (nonagenarian*):kw,ti,ab OR (centenarian*):kw,ti,ab OR (supercentenarian*):kw,ti,ab) AND ([MH Splenectomy] OR (([MH upper gastrointestinal tract] OR (upper intestine*):kw,ti,ab OR OR [MH esophagus] OR [MH stomach] OR [MH duodenum] OR [MH spleen] OR esophagus:ti,ab OR OR stomach:ti,ab OR duodenum:ti,ab OR spleen:ti,ab OR 'upper gi*':kw,ti,ab OR ugi:kw,ti,ab OR (gastro-esophageal):ti,ab) AND (resection:kw,ti,ab OR [MH surgical procedures, operative] OR surgery:kw,ti,ab OR surgical:kw,ti,ab)) OR [MH Fundoplication] OR fundoplication:kw,ti,ab OR [MH Gastrectomy] OR gastrectomy:kw,ti,ab OR (gastrect*):kw,ti,ab OR oesophagectomy OR (Splenect*):kw,ti,ab) AND ([MH Laparoscopes] OR [MH Laparoscopy] OR [MH Heller myotomy] OR (Heller myotomy):ti,ab,kw OR [MH Robotic Surgical Procedures] OR ((Robot*):ti,ab,kw AND ([MH surgical procedures, operative] OR surgery:kw,ti,ab OR surgical:kw,ti,ab)) OR (Laparoscop*):ti,ab,kw ) NOT ([MH Endometrial Neoplasms] OR [MH Ovarian Neoplasms] OR [MH Urinary Bladder Neoplasms] OR [MH Urogenital Neoplasms"[mh] OR "Whipple disease] OR Gynecologic:kw,ti,ab OR Urologic:kw,ti,ab OR Urothelial:kw,ti,ab OR Urethra:kw,ti,ab) ; 1/1/2000-12/31/2021 | | 114 |
| ClinTrials  (31 Mar) | ("Bariatric Surgery" OR Fundoplication OR Splenectomy OR pancreatectomy OR Gastrectomy OR (("Upper Gastrointestinal" OR "upper gI") AND surgery)) AND (laparoscopic OR laparoscopy OR robot) \| Older Adult | | 71 |
| **KQ18**  **SEARCH RESULTS** | Total items identified by database searches | | 2,317 |
|  | Total trials identified in searches | | 100 |
|  | Total KQ18 duplicates removed during Topic 3 screening | | 1,985 |
|  | Total KQ18 records identified during Topic 3 screening | | 232 |
|  | Hand-searched items located after Topic 3 screening | | 19 |
|  | **Total items screened** | | **251** |

### KQ19

| **Q19: Should laparoscopic (vs open) HPB surgery be used in the elderly?** | | | |
| --- | --- | --- | --- |
| **Search Concepts** | | **Elderly + HPB + MIS + Limits** | |
| **Database** | **Final search strategies** | | **Results** |
| PubMed  KQ19  (22 Mar) | (("Aged, 80 and over"[mh] OR "Frail Elderly"[mh] OR elderly[tiab] OR "older adult" [tw] OR septuagenar*[tw] OR octogenar*[tw] OR nonagenar*[tw] OR centenarian*[tw] OR supercentenar*[tw]) AND ("Hepato-pancreato-biliary"[tiab] OR HPB[tiab] OR hepatopancreaticobiliary[tiab] OR Whipple[tiab] OR Whipple [ot] OR ((liver[mh] OR liver[tiab] OR pancreas [mh] OR pancreas[tiab] OR gallbladder [mh] OR gallbladder [tiab] OR "bile ducts"[mh] OR "bile Duct*"[tiab] OR jejunum[mh] OR jejunum[tiab]) AND ("surgical procedures, operative"[mh] OR "general surgery"[mh] OR surgery[sh] OR surg*[tiab] OR resection[tiab])) OR Hepatectomy[mh] OR Hepatect*[tiab] OR pancreatectomy[mh] OR pancreatect*[tiab] OR Hepatojejunost*[tiab] OR ((Renal[tiab] OR biliary[tiab]) AND surger*[tiab])) AND (Laparoscopes[mh] OR Laparoscopy[mh] OR Laparoscop*[tiab] OR Laparoscop*[ot] OR "Heller myotomy"[mh] OR "Heller myotom*"[tiab] OR Cardiomyotom*[tw] OR "Robotic Surgical Procedures"[mh] OR ((Robot*[tiab] OR Robot*[ot]) AND (surgery[sh] OR surg*[tiab] OR "Surgical Procedures, Operative"[mh]))) AND ("Clinical Study"[pt] OR "Comparative Study"[pt] OR "Epidemiologic studies" [mh] OR "Evaluation Study"[pt] OR "Meta-Analysis"[pt] OR "Multicenter Study"[pt] OR "Systematic Review"[pt] OR "Validation Study" [pt] OR randomized[tiab] OR analysis[tiab] OR study[tiab] OR studies [tiab] OR "Practice Guideline"[pt] OR "practice guidelines as topic"[mh] OR guideline[title] OR guidelines[title])) NOT ("Case Reports"[pt] OR "case report*"[tiab] OR "case stud*"[tiab] OR ("animals"[MH:noexp] NOT "humans"[MH]) OR rat[tiab] OR rats[tiab] OR mouse[tiab] OR mice[tiab] OR dog[tiab] OR dogs[tiab] OR porcine[tiab] OR infant[mh] OR (child[mh] NOT adult[mh]) OR children[title] OR child[title] OR infant[title] OR infants[title] OR "Endometrial Neoplasms"[mh] OR "Ovarian Neoplasms"[mh] OR "Urinary Bladder Neoplasms"[mh] OR "Urogenital Neoplasms"[mh] OR "Whipple disease"[mh] OR "Whipple's disease"[tw] OR "Whipple disease"[tw] OR "Bladder cancer*"[tw] OR "Endometrial cancer*"[tw] OR "Ovarian cancer*"[tw] OR "Renal cancer*"[tw] OR Gynecologic[tiab] OR Urologic[tiab] OR Urothelial[tiab] OR Urethra[tiab] OR ("1905/01/01"[pdat]:"1999/12/31"[pdat])) | | 1,974 |
| Embase  KQ19  (22 Mar) | (('very elderly' OR 'frail elderly' OR elderly:kw,ti,ab OR 'septuagenarian*':kw,ti,ab OR 'octogenarian*':kw,ti,ab OR 'nonagenarian*':kw,ti,ab OR 'centenarian*':kw,ti,ab OR 'supercentenarian*':kw,ti,ab) AND ('biliary surgery' OR (('liver'/exp OR liver:kw,ti,ab OR 'pancreas'/exp OR pancreas:kw,ti,ab OR 'gallbladder'/exp OR gallbladder:kw,ti,ab OR 'bile duct'/exp OR 'bile duct' OR 'bile ducts' OR 'jejunum'/exp OR jejunum:kw,ti,ab) AND (resection:kw,ti,ab OR 'surgery'/exp OR surgery:kw,ti,ab OR surgical:kw,ti,ab)) OR 'liver surgery'/exp OR 'liver surgery' OR 'liver resection'/exp OR 'liver resection' OR 'pancreatectomy'/exp OR pancreatectomy OR 'hepatect*':kw,ti,ab OR 'nephrect*':kw,ti,ab OR 'hepatojejunostomy'/exp OR hepatojejunostomy:kw,ti,ab OR 'hepatopancreaticobiliary surgery' OR 'hepato pancreato biliary' OR hepatopancreaticobiliary OR HPB:kw,ti,ab OR 'biliary tract surgery'/exp OR 'biliary tract surgery' OR 'Renal surgery') AND ('laparoscope'/exp OR laparoscope OR 'laparoscopy'/exp OR laparoscop*:kw,ti,ab OR 'cardioesophagomyotomy'/exp OR cardioesophagomyotomy OR 'Heller myotomy' OR 'robot assisted surgery'/exp OR 'robot assisted surgery' OR (Robot*:kw,ti,ab AND ('surgery'/exp OR surgery:kw,ti,ab OR surgical:kw,ti,ab)) OR laparoscope) AND ('practice guideline'/exp OR 'practice guideline' OR [cochrane review]/lim OR [systematic review]/lim OR [meta analysis]/lim OR [clinical study]/lim OR 'clinical study'/exp) AND [2000-2021]/py) NOT ( 'case study'/exp OR 'case report' OR 'case study' OR [conference abstract]/lim OR [animals]/lim OR 'aged plant'/exp OR 'aged plant' OR 'child*':ti OR 'infant*':ti OR 'female genital tract tumor'/exp OR 'urogenital tract tumor'/exp OR (Whipple NEAR/3 disease) OR 'bladder cancer*':kw,ti,ab OR 'Endometrial cancer*':kw,ti,ab OR 'Ovarian cancer*':kw,ti,ab OR Gynecologic:kw,ti,ab OR Urologic:kw,ti,ab OR Urothelial:kw,ti,ab OR Urethra:kw,ti,ab) | | 936 |
| Cochrane  KQ19  (24 Mar) | ([MH Aged, 80 and over] OR [MH Frail Elderly] OR elderly:kw,ti,ab OR (septuagenarian*):kw,ti,ab OR (octogenarian*'):kw,ti,ab OR (nonagenarian*):kw,ti,ab OR (centenarian*):kw,ti,ab OR (supercentenarian*):kw,ti,ab) AND ([MH Hepatectomy] OR [MH pancreatectomy] OR (pancreatect*):ti,ab OR (Hepatojejunost*):ti,ab OR (Renal surger*):ti,ab OR (biliary surger*):ti,ab OR (Hepato-pancreato-biliary):ti,ab OR (HPB):ti,ab OR hepatopancreaticobiliary:ti,ab OR (([MH liver] OR (liver):ti,ab OR [MH pancreas] OR (pancreas):ti,ab OR [MH gallbladder] OR (gallbladder):ti,ab OR [MH bile ducts] OR (bile Duct*):ti,ab OR [MH kidney] OR (kidney*):ti,ab OR [MH jejunum] OR (jejunum):ti,ab) AND ([MH surgical procedures, operative] OR (resection):ti,ab,kw OR (surger*):ti,ab,kw)) OR (Hepatect*):ti,ab) AND ([MH Laparoscopes] OR [MH Laparoscopy] OR [MH Heller myotomy] OR (Heller myotomy):ti,ab,kw OR [MH Robotic Surgical Procedures] OR ((Robot*):ti,ab,kw AND ([MH surgical procedures, operative] OR surgery:kw,ti,ab OR surgical:kw,ti,ab)) OR (Laparoscop*):ti,ab,kw ) NOT ([MH Endometrial Neoplasms] OR [MH Ovarian Neoplasms] OR [MH Urinary Bladder Neoplasms] OR [MH Urogenital Neoplasms"[mh] OR "Whipple disease] OR Gynecologic:kw,ti,ab OR Urologic:kw,ti,ab OR Urothelial:kw,ti,ab OR Urethra:kw,ti,ab) ; 1/1/2000-12/31/2021 | | 112 |
| ClinTrials  (31 Mar) | ("Hepato-pancreato-biliary" OR hepatopancreaticobiliary OR Hepatectomy OR pancreatectomy OR Whipple) AND (laparoscopic OR laparoscopy OR robot) \| Older Adult | | 12 |
| **KQ19**  **SEARCH RESULTS** | Total items identified by database searches | | 3,034 |
|  | Total trials identified in searches | | 35 |
|  | Total KQ19 duplicates removed during Topic 3 screening | | 2785 |
|  | Total KQ19 records identified during Topic 3 screening | | 214 |
|  | Hand-searched items located after Topic 3 screening | | 9 |
|  | **Total items screened** (one duplicate removed) | | **233** |

### KQ20

| **Q20: Should laparoscopic (vs open) hernia surgery be used in the elderly?** | | | |
| --- | --- | --- | --- |
| **Search Concepts** | | **Elderly + Hernia + MIS + Limits** | |
| **Database** | **Final search strategies** | | **Results** |
| PubMed  KQ20  (22 Mar) | (("Aged, 80 and over"[mh] OR "Frail Elderly"[mh] OR elderly[tiab] OR "older adult" [tw] OR septuagenar*[tw] OR octogenar*[tw] OR nonagenar*[tw] OR centenarian*[tw] OR supercentenar*[tw]) AND (((Hernia[tiab] OR Hernia[mh]) AND ("surgical procedures, operative"[mh] OR "general surgery"[mh] OR surgery[sh] OR surg*[tiab])) OR Herniorrhaphy[mh] OR Herniorrhap*[tiab]) AND (Laparoscopes[mh] OR Laparoscopy[mh] OR Laparoscop*[tiab] OR Laparoscop*[ot] OR "Heller myotomy"[mh] OR "Heller myotom*"[tiab] OR Cardiomyotom*[tw] OR "Robotic Surgical Procedures"[mh] OR ((Robot*[tiab] OR Robot*[ot]) AND (surgery[sh] OR surg*[tiab] OR "Surgical Procedures, Operative"[mh]))) AND ("Clinical Study"[pt] OR "Comparative Study"[pt] OR "Epidemiologic studies" [mh] OR "Evaluation Study"[pt] OR "Meta-Analysis"[pt] OR "Multicenter Study"[pt] OR "Systematic Review"[pt] OR "Validation Study" [pt] OR randomized[tiab] OR analysis[tiab] OR study[tiab] OR studies [tiab] OR "Practice Guideline"[pt] OR "practice guidelines as topic"[mh] OR guideline[title] OR guidelines[title])) NOT ("Case Reports"[pt] OR "case report*"[tiab] OR "case stud*"[tiab] OR ("animals"[MH:noexp] NOT "humans"[MH]) OR rat[tiab] OR rats[tiab] OR mouse[tiab] OR mice[tiab] OR dog[tiab] OR dogs[tiab] OR porcine[tiab] OR infant[mh] OR (child[mh] NOT adult[mh]) OR children[title] OR child[title] OR infant[title] OR infants[title] OR "Endometrial Neoplasms"[mh] OR "Ovarian Neoplasms"[mh] OR "Urinary Bladder Neoplasms"[mh] OR "Urogenital Neoplasms"[mh] OR "Whipple disease"[mh] OR "Whipple's disease"[tw] OR "Whipple disease"[tw] OR "Bladder cancer*"[tw] OR "Endometrial cancer*"[tw] OR "Ovarian cancer*"[tw] OR "Renal cancer*"[tw] OR Gynecologic[tiab] OR Urologic[tiab] OR Urothelial[tiab] OR Urethra[tiab] OR ("1905/01/01"[pdat]:"1999/12/31"[pdat])) | | 1,084 |
| Embase  KQ20  (22 Mar) | (('very elderly' OR 'frail elderly' OR elderly:kw,ti,ab OR 'septuagenarian*':kw,ti,ab OR 'octogenarian*':kw,ti,ab OR 'nonagenarian*':kw,ti,ab OR 'centenarian*':kw,ti,ab OR 'supercentenarian*':kw,ti,ab) AND ('hernia surgery'/exp OR 'herniorrhaphy'/exp OR 'herniorrhap*':kw,ti,ab OR (('hernia'/exp OR 'hernia':kw,ti,ab) AND ('surgery'/exp OR surgery:kw,ti,ab OR surgical:kw,ti,ab)) OR 'hernia surgery') AND ('laparoscope'/exp OR laparoscope OR 'laparoscopy'/exp OR laparoscop*:kw,ti,ab OR 'cardioesophagomyotomy'/exp OR cardioesophagomyotomy OR 'Heller myotomy' OR 'robot assisted surgery'/exp OR 'robot assisted surgery' OR (Robot*:kw,ti,ab AND ('surgery'/exp OR surgery:kw,ti,ab OR surgical:kw,ti,ab)) OR laparoscope) AND ('practice guideline'/exp OR 'practice guideline' OR [cochrane review]/lim OR [systematic review]/lim OR [meta analysis]/lim OR [clinical study]/lim OR 'clinical study'/exp) AND [2000-2021]/py) NOT ( 'case study'/exp OR 'case report' OR 'case study' OR [conference abstract]/lim OR [animals]/lim OR 'aged plant'/exp OR 'aged plant' OR 'child*':ti OR 'infant*':ti OR 'female genital tract tumor'/exp OR 'urogenital tract tumor'/exp OR (Whipple NEAR/3 disease) OR 'bladder cancer*':kw,ti,ab OR 'Endometrial cancer*':kw,ti,ab OR 'Ovarian cancer*':kw,ti,ab OR Gynecologic:kw,ti,ab OR Urologic:kw,ti,ab OR Urothelial:kw,ti,ab OR Urethra:kw,ti,ab) | | 372 |
| Cochrane  KQ20  (24 Mar) | ([MH Aged, 80 and over] OR [MH Frail Elderly] OR elderly:kw,ti,ab OR (septuagenarian*):kw,ti,ab OR (octogenarian*'):kw,ti,ab OR (nonagenarian*):kw,ti,ab OR (centenarian*):kw,ti,ab OR (supercentenarian*):kw,ti,ab) AND ([MH Herniorrhaphy] OR (((Hernia):ti,ab,kw OR [MH Hernia]) AND ([MH surgical procedures, operative] OR surgery:kw,ti,ab OR surgical:kw,ti,ab)) OR (Herniorrhap*):ti,ab,kw) AND ([MH Laparoscopes] OR [MH Laparoscopy] OR [MH Heller myotomy] OR (Heller myotomy):ti,ab,kw OR [MH Robotic Surgical Procedures] OR ((Robot*):ti,ab,kw AND ([MH surgical procedures, operative] OR surgery:kw,ti,ab OR surgical:kw,ti,ab)) OR (Laparoscop*):ti,ab,kw ) NOT ([MH Endometrial Neoplasms] OR [MH Ovarian Neoplasms] OR [MH Urinary Bladder Neoplasms] OR [MH Urogenital Neoplasms"[mh] OR "Whipple disease] OR Gynecologic:kw,ti,ab OR Urologic:kw,ti,ab OR Urothelial:kw,ti,ab OR Urethra:kw,ti,ab) ; 1/1/2000-12/31/2021 | | 88 |
| ClinTrials  (31 Mar) | ("Herniorrhaphy" OR (Hernia AND Surgery)) AND (laparoscopic OR laparoscopy OR robot) \| Older Adult | | 29 |
| **KQ20**  **SEARCH RESULTS** | Total items identified by database searches | | 1,573 |
|  | Total trials identified in searches | | 32 |
|  | Total KQ20 duplicates removed during Topic 3 screening | | 1,477 |
|  | Total KQ20 records identified during Topic 3 screening | | 64 |
|  | Hand-searched items located after Topic 3 screening | | 1 |
|  | **Total items screened** | | **65** |

## Topic 4

| **Postoperative optimization and enhanced recovery after surgery (ERAS)**  (KQ21-KQ24 combined in this first screening) | | |
| --- | --- | --- |
| **Topic 4**  **Search**  **Results** | Total items identified by database searches | 2,321 |
|  | Additional articles located by handsearching | 90 |
|  | Total trials identified | 78 |
|  | Total duplicates | 830 |
|  | Total items screened | 1,503 |
|  | Records excluded | 1,399 |
|  | Unique items to be split between KQ21-KQ24 | 104 |

### KQ21

| **Q21: Should ERAS (vs conventional care) be used in elderly patients undergoing colorectal surgery?** | | | |
| --- | --- | --- | --- |
| **Search Concepts** | | **Elderly + Colorectal +ERAS + Limits** | |
| **Database** | **Final search strategies** | | **Results** |
| PubMed  KQ21  (22 Mar) | (("Aged, 80 and over"[mh] OR "Frail Elderly"[mh] OR elderly[tiab] OR "older adult" [tw] OR septuagenar*[tw] OR octogenar*[tw] OR nonagenar*[tw] OR centenarian*[tw] OR supercentenar*[tw]) AND ("Colorectal Surgery"[mh] OR ((abdomen[tiab] OR "anal canal"[tiab] OR "anal canal"[mh] OR anus[tiab] OR bowel[tiab] OR colon[tiab] OR colon [mh] OR rectum[tiab] OR rectum[mh] OR "Colon, Sigmoid"[mh] OR Sigmoid[tiab]) AND ("surgical procedures, operative"[mh] OR "general surgery"[mh] OR surgery[sh] OR resection[tiab] OR surg*[tiab])) OR ((rectal [tiab] OR Colorectal[tiab] OR abdominal[tiab]) AND Surger*[tiab])) AND ("Postoperative Care"[Mesh] OR "Enhanced Recovery After Surgery"[mh] OR "Enhanced Recovery"[tw] OR (ERAS[tiab] AND (Enhanced[tiab] OR Recovery[tiab] OR surgery[tiab] OR surgery[sh])) OR "Fast track"[tiab] OR "accelerated care pathway*"[tw] OR "clinical pathway*"[tw] OR " integrated care pathway*"[tw] OR "multimodal pathway*"[tiab] OR ("multimodal"[tiab] AND "pain"[tiab]) OR "Early Nutrition"[tw] OR "Early ambulation"[tw]) AND ("Clinical Study"[pt] OR "Comparative Study"[pt] OR "Epidemiologic studies" [mh] OR "Evaluation Study"[pt] OR "Meta-Analysis"[pt] OR "Multicenter Study"[pt] OR "Systematic Review"[pt] OR "Validation Study" [pt] OR randomized[tiab] OR analysis[tiab] OR study[tiab] OR studies [tiab] OR "Practice Guideline"[pt] OR "practice guidelines as topic"[mh] OR guideline[title] OR guidelines[title])) NOT ("Case Reports"[pt] OR "case report*"[tiab] OR "case stud*"[tiab] OR ("animals"[MH:noexp] NOT "humans"[MH]) OR rat[tiab] OR rats[tiab] OR mouse[tiab] OR mice[tiab] OR dog[tiab] OR dogs[tiab] OR porcine[tiab] OR infant[mh] OR (child[mh] NOT adult[mh]) OR children[title] OR child[title] OR infant[title] OR infants[title] OR "Endometrial Neoplasms"[mh] OR "Ovarian Neoplasms"[mh] OR "Urinary Bladder Neoplasms"[mh] OR "Urogenital Neoplasms"[mh] OR "Whipple disease"[mh] OR "Whipple's disease"[tw] OR "Whipple disease"[tw] OR "Bladder cancer*"[tw] OR "Endometrial cancer*"[tw] OR "Ovarian cancer*"[tw] OR "Renal cancer*"[tw] OR Gynecologic[tiab] OR Urologic[tiab] OR Urothelial[tiab] OR Urethra[tiab] OR ("1905/01/01"[pdat]:"1999/12/31"[pdat])) | | 644 |
| Embase  KQ21  (22 Mar) | (('very elderly' OR 'frail elderly' OR elderly:kw,ti,ab OR 'septuagenarian*':kw,ti,ab OR 'octogenarian*':kw,ti,ab OR 'nonagenarian*':kw,ti,ab OR 'centenarian*':kw,ti,ab OR 'supercentenarian*':kw,ti,ab) AND ('colorectal surgery'/exp OR 'abdominal surgery'/exp OR 'abdominal surgery' OR 'rectum surgery'/exp OR 'rectum surgery' OR (('abdomen'/exp OR abdomen OR 'anal canal'/exp OR 'anal canal' OR 'anus'/exp OR anus OR 'colon'/exp OR colon OR 'rectum'/exp OR rectum OR 'intestine'/exp OR intestine:ti,ab OR 'sigmoid'/exp OR sigmoid) AND (resection:kw,ti,ab OR 'surgery'/exp OR surgery:kw,ti,ab OR surgical:kw,ti,ab)) OR 'colorectal surgery') AND ('postoperative care'/exp OR 'postoperative care' OR 'enhanced recovery after surgery'/exp OR 'enhanced recovery' OR 'fast track' OR 'clinical pathway'/exp OR 'clinical pathway*' OR 'accelerated care pathway*' OR 'integrated care pathway*' OR 'multimodal pathway*' OR (multimodal NEAR/3 pain) OR 'early nutrition' OR 'early ambulation'/exp OR 'early ambulation' OR (eras:ti,ab AND (enhanced:ti,ab OR recovery:ti,ab OR surgery:ti,ab)) OR 'postoperative care') AND ('practice guideline'/exp OR 'practice guideline' OR [cochrane review]/lim OR [systematic review]/lim OR [meta analysis]/lim OR [clinical study]/lim OR 'clinical study'/exp) AND [2000-2021]/py) NOT ( 'case study'/exp OR 'case report' OR 'case study' OR [conference abstract]/lim OR [animals]/lim OR 'aged plant'/exp OR 'aged plant' OR 'child*':ti OR 'infant*':ti OR 'female genital tract tumor'/exp OR 'urogenital tract tumor'/exp OR (Whipple NEAR/3 disease) OR 'bladder cancer*':kw,ti,ab OR 'Endometrial cancer*':kw,ti,ab OR 'Ovarian cancer*':kw,ti,ab OR Gynecologic:kw,ti,ab OR Urologic:kw,ti,ab OR Urothelial:kw,ti,ab OR Urethra:kw,ti,ab) | | 546 |
| Cochrane  KQ21  (24 Mar) | ([MH Aged, 80 and over] OR [MH Frail Elderly] OR elderly:kw,ti,ab OR (septuagenarian*):kw,ti,ab OR (octogenarian*'):kw,ti,ab OR (nonagenarian*):kw,ti,ab OR (centenarian*):kw,ti,ab OR (supercentenarian*):kw,ti,ab) AND ([MH Colorectal Surgery] OR (abdominal surgery):kw,ti,ab OR (rectal surgery):kw,ti,ab OR (abdomen:ti,ab OR (anal canal):ti,ab OR (anus):ti,ab OR (colon):ti,ab OR (rectum):ti,ab OR (bowel):ti,ab OR (Sigmoid):ti,ab OR [MH anal canal] OR [MH colon] OR [MH rectum] ) AND ([MH surgical procedures, operative] OR resection:kw,ti,ab OR surgery:kw,ti,ab OR surgical:kw,ti,ab)) OR (colorectal surgery):kw,ti,ab) AND ([MH Postoperative Care] OR [MH Enhanced Recovery After Surgery] OR (Enhanced Recovery):ti,ab,kw OR ((ERAS):ti,ab AND ((Enhanced):ti,ab OR (Recovery):ti,ab OR (surgery):ti,ab,kw)) OR (Fast track):ti,ab,kw OR (accelerated care pathway*):ti,ab,kw OR (clinical pathway*):ti,ab,kw OR [MH critical pathways] OR (integrated care pathway*):ti,ab,kw OR (multimodal pathway*):ti,ab,kw OR ((multimodal):ti,ab AND (pain):ti,ab) OR (Early Nutrition):ti,ab,kw OR (Early ambulation):ti,ab,kw OR [MH (Early ambulation]) NOT ([MH Endometrial Neoplasms] OR [MH Ovarian Neoplasms] OR [MH Urinary Bladder Neoplasms] OR [MH Urogenital Neoplasms"[mh] OR "Whipple disease] OR Gynecologic:kw,ti,ab OR Urologic:kw,ti,ab OR Urothelial:kw,ti,ab OR Urethra:kw,ti,ab) ; 1/1/2000-12/31/2021 | | 210 |
| ClinTrials  (31 Mar) | ("Colorectal Surgery" OR "Abdominal Surgery" OR "Rectal Surgery") AND (ERAS OR "enhanced recovery") \| Older Adult | | 15 |
| **KQ21**  **SEARCH RESULTS** | Total items identified by database searches | | 1,415 |
|  | Total trials identified in searches | | 33 |
|  | Total KQ21 duplicates removed during Topic 4 screening | | 1,331 |
|  | Total KQ21 records identified during Topic 4 screening | | 51 |
|  | Hand-searched items located after Topic 4 screening | | 16 |
|  | **Total items screened** | | **67** |

### KQ22

| **Q22: Should ERAS (vs conventional care) be used in elderly patients undergoing UGI surgery?** | | | |
| --- | --- | --- | --- |
| **Search Concepts** | | **Elderly + UGI + ERAS + Limits** | |
| **Database** | **Final search strategies** | | **Results** |
| PubMed  KQ22  (1 Apr) | (("Aged, 80 and over"[mh] OR "Frail Elderly"[mh] OR elderly[tiab] OR "older adult" [tw] OR septuagenar*[tw] OR octogenar*[tw] OR nonagenar*[tw] OR centenarian*[tw] OR supercentenar*[tw]) AND ((("Upper Gastrointestinal Tract" [mh] OR esophagus[mh] OR esophagus[tiab] OR stomach[mh] OR stomach[tiab] OR duodenum[mh] OR duodenum[tiab] OR Spleen[mh] OR Spleen[tiab]) AND ("surgical procedures, operative"[mh] OR "general surgery"[mh] OR surgery[sh] OR resection[tiab] OR surg*[tiab])) OR "Bariatric Surgery"[mh] OR Fundoplication[mh] OR Fundoplication[tiab] OR Gastrectomy[mh] OR Gastrect*[tiab] OR Oesophagect*[tiab] OR Esophagectomy[mh] OR Esophagect*[tiab] OR Splenectomy[mh] OR Splenect*[tiab] OR ((UGI[tiab] OR "Upper GI*" OR "gastro-esophageal"[tiab] OR "Upper Gastrointest*"[tiab]) AND Surger*[tiab])) AND ("Postoperative Care"[Mesh] OR "Enhanced Recovery After Surgery"[mh] OR "Enhanced Recovery"[tw] OR (ERAS[tiab] AND (Enhanced[tiab] OR Recovery[tiab] OR surgery[tiab] OR surgery[sh])) OR "Fast track"[tiab] OR "accelerated care pathway*"[tw] OR "clinical pathway*"[tw] OR " integrated care pathway*"[tw] OR "multimodal pathway*"[tiab] OR ("multimodal"[tiab] AND "pain"[tiab]) OR "Early Nutrition"[tw] OR "Early ambulation"[tw]) AND ("Clinical Study"[pt] OR "Comparative Study"[pt] OR "Epidemiologic studies" [mh] OR "Evaluation Study"[pt] OR "Meta-Analysis"[pt] OR "Multicenter Study"[pt] OR "Systematic Review"[pt] OR "Validation Study" [pt] OR randomized[tiab] OR analysis[tiab] OR study[tiab] OR studies [tiab] OR "Practice Guideline"[pt] OR "practice guidelines as topic"[mh] OR guideline[title] OR guidelines[title])) NOT ("Case Reports"[pt] OR "case report*"[tiab] OR "case stud*"[tiab] OR ("animals"[MH:noexp] NOT "humans"[MH]) OR rat[tiab] OR rats[tiab] OR mouse[tiab] OR mice[tiab] OR dog[tiab] OR dogs[tiab] OR porcine[tiab] OR infant[mh] OR (child[mh] NOT adult[mh]) OR children[title] OR child[title] OR infant[title] OR infants[title] OR "Endometrial Neoplasms"[mh] OR "Esophageal Neoplasms"[mh] OR "Ovarian Neoplasms"[mh] OR "Urinary Bladder Neoplasms"[mh] OR "Urogenital Neoplasms"[mh] OR "Whipple disease"[mh] OR "Whipple's disease"[tw] OR "Whipple disease"[tw] OR "Bladder cancer*"[tw] OR "Endometrial cancer*"[tw] OR "Ovarian cancer*"[tw] OR "Renal cancer*"[tw] OR Gynecologic[tiab] OR Urologic[tiab] OR Urothelial[tiab] OR Urethra[tiab] OR ("1905/01/01"[pdat]:"1999/12/31"[pdat])) | | 154 |
| Embase  KQ22  (22 Mar) | (('very elderly' OR 'frail elderly' OR elderly:kw,ti,ab OR 'septuagenarian*':kw,ti,ab OR 'octogenarian*':kw,ti,ab OR 'nonagenarian*':kw,ti,ab OR 'centenarian*':kw,ti,ab OR 'supercentenarian*':kw,ti,ab) AND ('Splenectomy'/exp OR (('upper gastrointestinal tract'/exp OR 'upper intestine*':kw,ti,ab OR 'esophagus'/exp OR esophagus:kw,ti,ab OR 'spleen'/exp OR spleen:kw,ti,ab OR 'stomach'/exp OR stomach:kw,ti,ab OR 'duodenum'/exp OR duodenum:kw,ti,ab OR 'upper gi*':kw,ti,ab OR ugi:ti,ab) AND (resection:kw,ti,ab OR 'surgery'/exp OR surgery:kw,ti,ab OR surgical:kw,ti,ab)) OR 'stomach fundoplication'/exp OR fundoplication:kw,ti,ab OR 'gastrectomy'/exp OR gastrectomy:kw,ti,ab OR 'gastrect*':kw,ti,ab OR oesophagectomy OR 'esophagus resection'/exp OR 'esophagus resection':kw,ti,ab OR esophagectomy:kw,ti,ab OR 'Splenect*':kw,ti,ab) AND ('postoperative care'/exp OR 'postoperative care' OR 'enhanced recovery after surgery'/exp OR 'enhanced recovery' OR 'fast track' OR 'clinical pathway'/exp OR 'clinical pathway*' OR 'accelerated care pathway*' OR 'integrated care pathway*' OR 'multimodal pathway*' OR (multimodal NEAR/3 pain) OR 'early nutrition' OR 'early ambulation'/exp OR 'early ambulation' OR (eras:ti,ab AND (enhanced:ti,ab OR recovery:ti,ab OR surgery:ti,ab)) OR 'postoperative care') AND ('practice guideline'/exp OR 'practice guideline' OR [cochrane review]/lim OR [systematic review]/lim OR [meta analysis]/lim OR [clinical study]/lim OR 'clinical study'/exp) AND [2000-2021]/py) NOT ( 'case study'/exp OR 'case report' OR 'case study' OR [conference abstract]/lim OR [animals]/lim OR 'aged plant'/exp OR 'aged plant' OR 'child*':ti OR 'infant*':ti OR 'female genital tract tumor'/exp OR 'urogenital tract tumor'/exp OR 'esophagus tumor'/exp OR (Whipple NEAR/3 disease) OR 'bladder cancer*':kw,ti,ab OR 'Endometrial cancer*':kw,ti,ab OR 'Ovarian cancer*':kw,ti,ab OR Gynecologic:kw,ti,ab OR Urologic:kw,ti,ab OR Urothelial:kw,ti,ab OR Urethra:kw,ti,ab) | | 94 |
| Cochrane  KQ22  (24 Mar) | ([MH Aged, 80 and over] OR [MH Frail Elderly] OR elderly:kw,ti,ab OR (septuagenarian*):kw,ti,ab OR (octogenarian*'):kw,ti,ab OR (nonagenarian*):kw,ti,ab OR (centenarian*):kw,ti,ab OR (supercentenarian*):kw,ti,ab) AND ([MH Splenectomy] OR (([MH upper gastrointestinal tract] OR (upper intestine*):kw,ti,ab OR OR [MH esophagus] OR [MH stomach] OR [MH duodenum] OR [MH spleen] OR esophagus:ti,ab OR OR stomach:ti,ab OR duodenum:ti,ab OR spleen:ti,ab OR 'upper gi*':kw,ti,ab OR ugi:kw,ti,ab OR (gastro-esophageal):ti,ab) AND (resection:kw,ti,ab OR [MH surgical procedures, operative] OR surgery:kw,ti,ab OR surgical:kw,ti,ab)) OR [MH Fundoplication] OR fundoplication:kw,ti,ab OR [MH Gastrectomy] OR gastrectomy:kw,ti,ab OR (gastrect*):kw,ti,ab OR oesophagectomy OR (Splenect*):kw,ti,ab) AND ([MH Postoperative Care] OR [MH Enhanced Recovery After Surgery] OR (Enhanced Recovery):ti,ab,kw OR ((ERAS):ti,ab AND ((Enhanced):ti,ab OR (Recovery):ti,ab OR (surgery):ti,ab,kw)) OR (Fast track):ti,ab,kw OR (accelerated care pathway*):ti,ab,kw OR (clinical pathway*):ti,ab,kw OR [MH critical pathways] OR (integrated care pathway*):ti,ab,kw OR (multimodal pathway*):ti,ab,kw OR ((multimodal):ti,ab AND (pain):ti,ab) OR (Early Nutrition):ti,ab,kw OR (Early ambulation):ti,ab,kw OR [MH (Early ambulation]) NOT ([MH Endometrial Neoplasms] OR [MH Ovarian Neoplasms] OR [MH Urinary Bladder Neoplasms] OR [MH Urogenital Neoplasms"[mh] OR "Whipple disease] OR Gynecologic:kw,ti,ab OR Urologic:kw,ti,ab OR Urothelial:kw,ti,ab OR Urethra:kw,ti,ab) ; 1/1/2000-12/31/2021 | | 32 |
| ClinTrials  (31 Mar) | ("Bariatric Surgery" OR Fundoplication OR Splenectomy OR pancreatectomy OR Gastrectomy OR (("Upper Gastrointestinal" OR "upper gI") AND surgery)) AND (ERAS OR "enhanced recovery") \| Older Adult | | 4 |
| **KQ22**  **SEARCH RESULTS** | Total items identified by database searches | | 284 |
|  | Total trials identified in searches | | 6 |
|  | Total KQ22 duplicates removed during Topic 4 screening | | 269 |
|  | Total KQ22 records identified during Topic 4 screening | | 9 |
|  | Hand-searched items located after Topic 4 screening | | 3 |
|  | **Total items screened** | | **12** |

### KQ23

| **Q23: Should ERAS (vs conventional care) be used in elderly patients undergoing HPB surgery ?** | | | |
| --- | --- | --- | --- |
| **Search Concepts** | | **Elderly + HPB + ERAS + Limits** | |
| **Database** | **Final search strategies** | | **Results** |
| PubMed  KQ23  (22 Mar) | (("Aged, 80 and over"[mh] OR "Frail Elderly"[mh] OR elderly[tiab] OR "older adult" [tw] OR septuagenar*[tw] OR octogenar*[tw] OR nonagenar*[tw] OR centenarian*[tw] OR supercentenar*[tw]) AND ("Hepato-pancreato-biliary"[tiab] OR HPB[tiab] OR hepatopancreaticobiliary[tiab] OR Whipple[tiab] OR Whipple [ot] OR ((liver[mh] OR liver[tiab] OR pancreas [mh] OR pancreas[tiab] OR gallbladder [mh] OR gallbladder [tiab] OR "bile ducts"[mh] OR "bile Duct*"[tiab] OR jejunum[mh] OR jejunum[tiab]) AND ("surgical procedures, operative"[mh] OR "general surgery"[mh] OR surgery[sh] OR surg*[tiab] OR resection[tiab])) OR Hepatectomy[mh] OR Hepatect*[tiab] OR pancreatectomy[mh] OR pancreatect*[tiab] OR Hepatojejunost*[tiab] OR ((Renal[tiab] OR biliary[tiab]) AND surger*[tiab])) AND ("Postoperative Care"[Mesh] OR "Enhanced Recovery After Surgery"[mh] OR "Enhanced Recovery"[tw] OR (ERAS[tiab] AND (Enhanced[tiab] OR Recovery[tiab] OR surgery[tiab] OR surgery[sh])) OR "Fast track"[tiab] OR "accelerated care pathway*"[tw] OR "clinical pathway*"[tw] OR " integrated care pathway*"[tw] OR "multimodal pathway*"[tiab] OR ("multimodal"[tiab] AND "pain"[tiab]) OR "Early Nutrition"[tw] OR "Early ambulation"[tw]) AND ("Clinical Study"[pt] OR "Comparative Study"[pt] OR "Epidemiologic studies" [mh] OR "Evaluation Study"[pt] OR "Meta-Analysis"[pt] OR "Multicenter Study"[pt] OR "Systematic Review"[pt] OR "Validation Study" [pt] OR randomized[tiab] OR analysis[tiab] OR study[tiab] OR studies [tiab] OR "Practice Guideline"[pt] OR "practice guidelines as topic"[mh] OR guideline[title] OR guidelines[title])) NOT ("Case Reports"[pt] OR "case report*"[tiab] OR "case stud*"[tiab] OR ("animals"[MH:noexp] NOT "humans"[MH]) OR rat[tiab] OR rats[tiab] OR mouse[tiab] OR mice[tiab] OR dog[tiab] OR dogs[tiab] OR porcine[tiab] OR infant[mh] OR (child[mh] NOT adult[mh]) OR children[title] OR child[title] OR infant[title] OR infants[title] OR "Endometrial Neoplasms"[mh] OR "Ovarian Neoplasms"[mh] OR "Urinary Bladder Neoplasms"[mh] OR "Urogenital Neoplasms"[mh] OR "Whipple disease"[mh] OR "Whipple's disease"[tw] OR "Whipple disease"[tw] OR "Bladder cancer*"[tw] OR "Endometrial cancer*"[tw] OR "Ovarian cancer*"[tw] OR "Renal cancer*"[tw] OR Gynecologic[tiab] OR Urologic[tiab] OR Urothelial[tiab] OR Urethra[tiab] OR ("1905/01/01"[pdat]:"1999/12/31"[pdat])) | | 258 |
| Embase  KQ23  (22 Mar) | (('very elderly' OR 'frail elderly' OR elderly:kw,ti,ab OR 'septuagenarian*':kw,ti,ab OR 'octogenarian*':kw,ti,ab OR 'nonagenarian*':kw,ti,ab OR 'centenarian*':kw,ti,ab OR 'supercentenarian*':kw,ti,ab) AND ('biliary surgery' OR (('liver'/exp OR liver:kw,ti,ab OR 'pancreas'/exp OR pancreas:kw,ti,ab OR 'gallbladder'/exp OR gallbladder:kw,ti,ab OR 'bile duct'/exp OR 'bile duct' OR 'bile ducts' OR 'jejunum'/exp OR jejunum:kw,ti,ab) AND (resection:kw,ti,ab OR 'surgery'/exp OR surgery:kw,ti,ab OR surgical:kw,ti,ab)) OR 'liver surgery'/exp OR 'liver surgery' OR 'liver resection'/exp OR 'liver resection' OR 'pancreatectomy'/exp OR pancreatectomy OR 'hepatect*':kw,ti,ab OR 'nephrect*':kw,ti,ab OR 'hepatojejunostomy'/exp OR hepatojejunostomy:kw,ti,ab OR 'hepatopancreaticobiliary surgery' OR 'hepato pancreato biliary' OR hepatopancreaticobiliary OR HPB:kw,ti,ab OR 'biliary tract surgery'/exp OR 'biliary tract surgery' OR 'Renal surgery') AND ('postoperative care'/exp OR 'postoperative care' OR 'enhanced recovery after surgery'/exp OR 'enhanced recovery' OR 'fast track' OR 'clinical pathway'/exp OR 'clinical pathway*' OR 'accelerated care pathway*' OR 'integrated care pathway*' OR 'multimodal pathway*' OR (multimodal NEAR/3 pain) OR 'early nutrition' OR 'early ambulation'/exp OR 'early ambulation' OR (eras:ti,ab AND (enhanced:ti,ab OR recovery:ti,ab OR surgery:ti,ab)) OR 'postoperative care') AND ('practice guideline'/exp OR 'practice guideline' OR [cochrane review]/lim OR [systematic review]/lim OR [meta analysis]/lim OR [clinical study]/lim OR 'clinical study'/exp) AND [2000-2021]/py) NOT ( 'case study'/exp OR 'case report' OR 'case study' OR [conference abstract]/lim OR [animals]/lim OR 'aged plant'/exp OR 'aged plant' OR 'child*':ti OR 'infant*':ti OR 'female genital tract tumor'/exp OR 'urogenital tract tumor'/exp OR (Whipple NEAR/3 disease) OR 'bladder cancer*':kw,ti,ab OR 'Endometrial cancer*':kw,ti,ab OR 'Ovarian cancer*':kw,ti,ab OR Gynecologic:kw,ti,ab OR Urologic:kw,ti,ab OR Urothelial:kw,ti,ab OR Urethra:kw,ti,ab) | | 168 |
| Cochrane  KQ23  (24 Mar) | ([MH Aged, 80 and over] OR [MH Frail Elderly] OR elderly:kw,ti,ab OR (septuagenarian*):kw,ti,ab OR (octogenarian*'):kw,ti,ab OR (nonagenarian*):kw,ti,ab OR (centenarian*):kw,ti,ab OR (supercentenarian*):kw,ti,ab) AND ([MH Hepatectomy] OR [MH pancreatectomy] OR (pancreatect*):ti,ab OR (Hepatojejunost*):ti,ab OR (Renal surger*):ti,ab OR (biliary surger*):ti,ab OR (Hepato-pancreato-biliary):ti,ab OR (HPB):ti,ab OR hepatopancreaticobiliary:ti,ab OR (([MH liver] OR (liver):ti,ab OR [MH pancreas] OR (pancreas):ti,ab OR [MH gallbladder] OR (gallbladder):ti,ab OR [MH bile ducts] OR (bile Duct*):ti,ab OR [MH kidney] OR (kidney*):ti,ab OR [MH jejunum] OR (jejunum):ti,ab) AND ([MH surgical procedures, operative] OR (resection):ti,ab,kw OR (surger*):ti,ab,kw)) OR (Hepatect*):ti,ab) AND ([MH Postoperative Care] OR [MH Enhanced Recovery After Surgery] OR (Enhanced Recovery):ti,ab,kw OR ((ERAS):ti,ab AND ((Enhanced):ti,ab OR (Recovery):ti,ab OR (surgery):ti,ab,kw)) OR (Fast track):ti,ab,kw OR (accelerated care pathway*):ti,ab,kw OR (clinical pathway*):ti,ab,kw OR [MH critical pathways] OR (integrated care pathway*):ti,ab,kw OR (multimodal pathway*):ti,ab,kw OR ((multimodal):ti,ab AND (pain):ti,ab) OR (Early Nutrition):ti,ab,kw OR (Early ambulation):ti,ab,kw OR [MH (Early ambulation]) NOT ([MH Endometrial Neoplasms] OR [MH Ovarian Neoplasms] OR [MH Urinary Bladder Neoplasms] OR [MH Urogenital Neoplasms"[mh] OR "Whipple disease] OR Gynecologic:kw,ti,ab OR Urologic:kw,ti,ab OR Urothelial:kw,ti,ab OR Urethra:kw,ti,ab) ; 1/1/2000-12/31/2021 | | 58 |
| ClinTrials  (31 Mar) | ("Hepato-pancreato-biliary" OR hepatopancreaticobiliary OR Hepatectomy OR pancreatectomy OR Whipple) AND (ERAS OR "enhanced recovery") \| Older Adult | | 21 |
| **KQ23**  **SEARCH RESULTS** | Total items identified by database searches | | 505 |
|  | Total trials identified in searches | | 30 |
|  | Total KQ23 duplicates removed during Topic 4 screening | | 467 |
|  | Total KQ23 records identified during Topic 4 screening | | 8 |
|  | Hand-searched items located after Topic 4 screening | | 9 |
|  | **Total items screened** | | **17** |

### KQ24

| **Q24: Should ERAS (vs conventional care) be used in elderly patients undergoing hernia surgery ?** | | | |
| --- | --- | --- | --- |
| **Search Concepts** | | **Elderly + Hernia + ERAS + Limits** | |
| **Database** | **Final search strategies** | | **Results** |
| PubMed  KQ24  (22 Mar) | (("Aged, 80 and over"[mh] OR "Frail Elderly"[mh] OR elderly[tiab] OR "older adult" [tw] OR septuagenar*[tw] OR octogenar*[tw] OR nonagenar*[tw] OR centenarian*[tw] OR supercentenar*[tw]) AND (((Hernia[tiab] OR Hernia[mh]) AND ("surgical procedures, operative"[mh] OR "general surgery"[mh] OR surgery[sh] OR surg*[tiab])) OR Herniorrhaphy[mh] OR Herniorrhap*[tiab]) AND ("Postoperative Care"[Mesh] OR "Enhanced Recovery After Surgery"[mh] OR "Enhanced Recovery"[tw] OR (ERAS[tiab] AND (Enhanced[tiab] OR Recovery[tiab] OR surgery[tiab] OR surgery[sh])) OR "Fast track"[tiab] OR "accelerated care pathway*"[tw] OR "clinical pathway*"[tw] OR " integrated care pathway*"[tw] OR "multimodal pathway*"[tiab] OR ("multimodal"[tiab] AND "pain"[tiab]) OR "Early Nutrition"[tw] OR "Early ambulation"[tw]) AND ("Clinical Study"[pt] OR "Comparative Study"[pt] OR "Epidemiologic studies" [mh] OR "Evaluation Study"[pt] OR "Meta-Analysis"[pt] OR "Multicenter Study"[pt] OR "Systematic Review"[pt] OR "Validation Study" [pt] OR randomized[tiab] OR analysis[tiab] OR study[tiab] OR studies [tiab] OR "Practice Guideline"[pt] OR "practice guidelines as topic"[mh] OR guideline[title] OR guidelines[title])) NOT ("Case Reports"[pt] OR "case report*"[tiab] OR "case stud*"[tiab] OR ("animals"[MH:noexp] NOT "humans"[MH]) OR rat[tiab] OR rats[tiab] OR mouse[tiab] OR mice[tiab] OR dog[tiab] OR dogs[tiab] OR porcine[tiab] OR infant[mh] OR (child[mh] NOT adult[mh]) OR children[title] OR child[title] OR infant[title] OR infants[title] OR "Endometrial Neoplasms"[mh] OR "Ovarian Neoplasms"[mh] OR "Urinary Bladder Neoplasms"[mh] OR "Urogenital Neoplasms"[mh] OR "Whipple disease"[mh] OR "Whipple's disease"[tw] OR "Whipple disease"[tw] OR "Bladder cancer*"[tw] OR "Endometrial cancer*"[tw] OR "Ovarian cancer*"[tw] OR "Renal cancer*"[tw] OR Gynecologic[tiab] OR Urologic[tiab] OR Urothelial[tiab] OR Urethra[tiab] OR ("1905/01/01"[pdat]:"1999/12/31"[pdat])) | | 44 |
| Embase  KQ24  (22 Mar) | (('very elderly' OR 'frail elderly' OR elderly:kw,ti,ab OR 'septuagenarian*':kw,ti,ab OR 'octogenarian*':kw,ti,ab OR 'nonagenarian*':kw,ti,ab OR 'centenarian*':kw,ti,ab OR 'supercentenarian*':kw,ti,ab) AND ('hernia surgery'/exp OR 'herniorrhaphy'/exp OR 'herniorrhap*':kw,ti,ab OR (('hernia'/exp OR 'hernia':kw,ti,ab) AND ('surgery'/exp OR surgery:kw,ti,ab OR surgical:kw,ti,ab)) OR 'hernia surgery') AND ('postoperative care'/exp OR 'postoperative care' OR 'enhanced recovery after surgery'/exp OR 'enhanced recovery' OR 'fast track' OR 'clinical pathway'/exp OR 'clinical pathway*' OR 'accelerated care pathway*' OR 'integrated care pathway*' OR 'multimodal pathway*' OR (multimodal NEAR/3 pain) OR 'early nutrition' OR 'early ambulation'/exp OR 'early ambulation' OR (eras:ti,ab AND (enhanced:ti,ab OR recovery:ti,ab OR surgery:ti,ab)) OR 'postoperative care') AND ('practice guideline'/exp OR 'practice guideline' OR [cochrane review]/lim OR [systematic review]/lim OR [meta analysis]/lim OR [clinical study]/lim OR 'clinical study'/exp) AND [2000-2021]/py) NOT ( 'case study'/exp OR 'case report' OR 'case study' OR [conference abstract]/lim OR [animals]/lim OR 'aged plant'/exp OR 'aged plant' OR 'child*':ti OR 'infant*':ti OR 'female genital tract tumor'/exp OR 'urogenital tract tumor'/exp OR (Whipple NEAR/3 disease) OR 'bladder cancer*':kw,ti,ab OR 'Endometrial cancer*':kw,ti,ab OR 'Ovarian cancer*':kw,ti,ab OR Gynecologic:kw,ti,ab OR Urologic:kw,ti,ab OR Urothelial:kw,ti,ab OR Urethra:kw,ti,ab) | | 56 |
| Cochrane  KQ24  (24 Mar) | ([MH Aged, 80 and over] OR [MH Frail Elderly] OR elderly:kw,ti,ab OR (septuagenarian*):kw,ti,ab OR (octogenarian*'):kw,ti,ab OR (nonagenarian*):kw,ti,ab OR (centenarian*):kw,ti,ab OR (supercentenarian*):kw,ti,ab) AND ([MH Herniorrhaphy] OR (((Hernia):ti,ab,kw OR [MH Hernia]) AND ([MH surgical procedures, operative] OR surgery:kw,ti,ab OR surgical:kw,ti,ab)) OR (Herniorrhap*):ti,ab,kw) AND ([MH Postoperative Care] OR [MH Enhanced Recovery After Surgery] OR (Enhanced Recovery):ti,ab,kw OR ((ERAS):ti,ab AND ((Enhanced):ti,ab OR (Recovery):ti,ab OR (surgery):ti,ab,kw)) OR (Fast track):ti,ab,kw OR (accelerated care pathway*):ti,ab,kw OR (clinical pathway*):ti,ab,kw OR [MH critical pathways] OR (integrated care pathway*):ti,ab,kw OR (multimodal pathway*):ti,ab,kw OR ((multimodal):ti,ab AND (pain):ti,ab) OR (Early Nutrition):ti,ab,kw OR (Early ambulation):ti,ab,kw OR [MH (Early ambulation]) NOT ([MH Endometrial Neoplasms] OR [MH Ovarian Neoplasms] OR [MH Urinary Bladder Neoplasms] OR [MH Urogenital Neoplasms"[mh] OR "Whipple disease] OR Gynecologic:kw,ti,ab OR Urologic:kw,ti,ab OR Urothelial:kw,ti,ab OR Urethra:kw,ti,ab) ; 1/1/2000-12/31/2021 | | 8 |
| ClinTrials  (31 Mar) | ("Herniorrhaphy" OR (Hernia AND Surgery)) AND (ERAS OR "enhanced recovery") \| Older Adult | | 9 |
| **KQ24**  **SEARCH RESULTS** | Total items identified by database searches | | 117 |
|  | Total trials identified in searches | | 9 |
|  | Total KQ24 duplicates removed during Topic 4 screening | | 104 |
|  | Total KQ24 records identified during Topic 4 screening | | 4 |
|  | Hand-searched items located after Topic 4 screening | | 1 |
|  | **Total items screened** | | **5** |
