# Supplementary material for: EAES/SAGES evidence-based recommendations and expert consensus on optimization of perioperative care in older adults
Source: Surg Endosc. 2024 Jun 28;38(8):4104–26. doi: 10.1007/s00464-024-10977-7 (PMC11289045; doi:10.1007/s00464-024-10977-7)
Supplement: Supplementary file 2 — Supplement 2 PRISMA Flow Diagrams Supplementary file2 (DOCX 614 KB) [file 464_2024_10977_MOESM2_ESM.docx]

**Optimization of Perioperative Care in Older Adults**

# PRISMA Flow Diagram

## Topic 2 / KQ1-KQ16

(See individual charts for additional information)

Records identified through database searching

ClinicalTrials.gov/NLM (n = 321)

Cochrane Library/Cochrane (n = 1,377)

Embase/Elsevier (n = 6,616)

PubMed/NLM (n = 11,423)

(n = 19,737 including Topic 2 = 4,017)

Additional articles identified through handsearching trials and citation searching

ClinicalTrials.gov/NLM (n = 15)

PubMed/NLM (n = 60)

Citation searching (n = 293)

(n = 368 including Topic 2 = 65

and an additional KQ1-KQ16 = 14)

## Literature Search

Records removed

Duplicates identified during handsearching (n=169)

Duplicates identified by Endnotes (n = 7,829)

Duplicates identified manually in Endnotes (n = 11)

Duplicates identified by Covidence (n = 658)

Trials (n = 537)

(n = 9,204 including Topic 2 = 1,448)

Records available for screening

(n = 10,901 including Topic 2 = 2,634)

Records not identified for Topic 2 (n = 8,259)

## Screening

Records identified for Topic 2 and excluded
(n = 2,542)

Records available for second screening

(n = 92 including KQ1 - KQ16 = 155)

*(Note: includes duplicates)*

Full-text articles excluded

(n = 144)

*See individual KQ1-KQ16 PRISMA charts for details*

## Eligibility

KQ1-KQ16 Full-text articles assessed for eligibility
(n = 169 including 14 hand searched additions)

Included records available for analysis

(n = 25)

Studies excluded
(n = 9)

8 duplicate studies

1 duplicate reference

## Included

Studies combined and included

in appraisal/guideline/recommendations
(n = 16)

*(17 articles total, 2 reference the same study)*

See Literature Searches Summary document for search details.

**PRISMA Flow Diagram**

## KQ1

Additional articles identified through handsearching trials and citation searching

ClinicalTrials.gov/NLM (n = 15)

PubMed/NLM (n = 60)

Citation searching (n = 293)

(n = 368 including Topic 2 = 65

and KQ1 = 10)

Records identified through database searching

ClinicalTrials.gov/NLM (n = 321)

Cochrane Library/Cochrane (n = 1,377)

Embase/Elsevier (n = 6,616)

PubMed/NLM (n = 11,423)

(n = 19,737 including Topic 2 = 4,026

and KQ1 = 1,957)

## Literature Search

Records removed

Duplicates identified during handsearching (n=169)

Duplicates identified by Endnotes (n = 7,829)

Duplicates identified manually in Endnotes (n = 11)

Duplicates identified by Covidence (n = 658)

Trials (n = 537)

(n = 9,204 including Topic 2 = 1,448)

Records not identified for Topic 2 (n = 8,259)

Records available for screening

(n = 10,901 including Topic 2 = 2,634)

Records identified for Topic 2 and excluded
(n = 2,542 including KQ1 = 1,895)

## Screening

Records available for second screening

(n = 92 including KQ1 = 62)

Records not identified for KQ1
(n = 30)

Full-text articles excluded

(n = 57)

16 Wrong intervention

10 Wrong patient population

8 No intervention performed

7 SR – Citations searched

6 Wrong outcomes

3 Research protocol

3 Wrong KQ

1 Conference abstract

1 Narrative review/opinion

1 Wrong comparator

1 Wrong study design

KQ1 Full-text articles assessed for eligibility
(n = 70 with 8 hand searched additions)

## Eligibility

Included studies added to combined analysis

(n = 13)

## Included

See chart KQ1-KQ16 for details on the combined quantitative analysis (n=16)

See Literature Searches Summary document for search details.

**PRISMA Flow Diagram**

## KQ2

Additional articles identified through handsearching trials and citation searching

ClinicalTrials.gov/NLM (n = 15)

PubMed/NLM (n = 60)

Citation searching (n = 293)

(n = 368 including Topic 2 = 65

and KQ2 = 2)

Records identified through database searching

ClinicalTrials.gov/NLM (n = 321)

Cochrane Library/Cochrane (n = 1,377)

Embase/Elsevier (n = 6,616)

PubMed/NLM (n = 11,423)

(n = 19,737 including Topic 2 = 4,026

and KQ2 = 498)

## Literature Search

Records removed

Duplicates identified during handsearching (n=169)

Duplicates identified by Endnotes (n = 7,829)

Duplicates identified manually in Endnotes (n = 11)

Duplicates identified by Covidence (n = 658)

Trials (n = 537)

(n = 9,204 including Topic 2 = 1,448)

Records not identified for Topic 2 (n = 8,259)

Records available for screening

(n = 10,901 including Topic 2 = 2,634)

Records identified for Topic 2 and excluded
(n = 2,542 including and KQ2 = 476)

## Screening

Records not identified for KQ2
(n = 70)

Records available for second screening

(n = 92 including KQ2 = 22)

KQ2 Full-text articles assessed for eligibility
(n = 24 with 2 hand searched additions)

Full-text articles excluded

(n = 20)

9 Wrong patient population

5 Wrong intervention

3 Wrong study design

2 SR - Citations searched

1 Wrong outcomes

## Eligibility

Included studies added to combined analysis

(n = 4)

## Included

See chart KQ1-KQ16 for details on the combined quantitative analysis (n=16)

See Literature Searches Summary document for search details.

**PRISMA Flow Diagram**

## KQ3

Additional articles identified through handsearching trials and citation searching

ClinicalTrials.gov/NLM (n = 15)

PubMed/NLM (n = 60)

Citation searching (n = 293)

(n = 368 including Topic 2 = 65

and KQ3 = 3)

Records identified through database searching

ClinicalTrials.gov/NLM (n = 321)

Cochrane Library/Cochrane (n = 1,377)

Embase/Elsevier (n = 6,616)

PubMed/NLM (n = 11,423)

(n = 19,737 including Topic 2 = 4,026

and KQ3 = 983)

## Literature Search

Records removed

Duplicates identified during handsearching (n=169)

Duplicates identified by Endnotes (n = 7,829)

Duplicates identified manually in Endnotes (n = 11)

Duplicates identified by Covidence (n = 658)

Trials (n = 537)

(n = 9,204 including Topic 2 = 1,448)

Records not identified for Topic 2 (n = 8,259)

Records available for screening

(n = 10,901 including Topic 2 = 2,634)

Records identified for Topic 2 and excluded
(n = 2,542 including and KQ3 = 958)

## Screening

Records not identified for KQ3
(n = 67)

Records available for second screening

(n = 92 including KQ3 = 25)

## Eligibility

KQ3 Full-text articles assessed for eligibility
(n = 28 with 3 hand searched additions)

Full-text articles excluded

(n = 25)

12 Wrong patient population

6 Wrong intervention

4 Wrong study design

2 SR - Citations searched

1 Adult population

Included studies added to combined analysis

(n = 3)

## Included

See chart KQ1-KQ16 for details on the combined quantitative analysis (n=16)

See Literature Searches Summary document for search details.

**PRISMA Flow Diagram**

## KQ4

Additional articles identified through handsearching trials and citation searching

ClinicalTrials.gov/NLM (n = 15)

PubMed/NLM (n = 60)

Citation searching (n = 293)

(n = 368 including Topic 2 = 65

and KQ4 = 1)

Records identified through database searching

ClinicalTrials.gov/NLM (n = 321)

Cochrane Library/Cochrane (n = 1,377)

Embase/Elsevier (n = 6,616)

PubMed/NLM (n = 11,423)

(n = 19,737 including Topic 2 = 4,026

and KQ4 = 168)

## Literature Search

Records removed

Duplicates identified during handsearching (n=169)

Duplicates identified by Endnotes (n = 7,829)

Duplicates identified manually in Endnotes (n = 11)

Duplicates identified by Covidence (n = 658)

Trials (n = 537)

(n = 9,204 including Topic 2 = 1,448)

Records not identified for Topic 2 (n = 8,259)

Records available for screening

(n = 10,901 including Topic 2 = 2,634)

Records identified for Topic 2 and excluded
(n = 2,542 including and KQ4 = 154)

## Screening

Records not identified for KQ4
(n = 78)

Records available for second screening

(n = 92 including KQ4 = 14)

KQ4 Full-text articles assessed for eligibility
(n = 15 with 1 hand searched addition)

Full-text articles excluded

(n = 14)

6 Wrong patient population

3 Wrong intervention

3 Wrong study design

1 SR - Citations searched

1 Wrong outcomes

## Eligibility

Included studies added to combined analysis

(n = 1)

## Included

See chart KQ1-KQ16 for details on the combined quantitative analysis (n=16)

See Literature Searches Summary document for search details.

**PRISMA Flow Diagram**

## KQ5-KQ8

Additional articles identified through handsearching trials and citation searching

ClinicalTrials.gov/NLM (n = 15)

PubMed/NLM (n = 60)

Citation searching (n = 293)

(n = 368 including Topic 2 = 65 and

KQ5, KQ6, KQ7, KQ8 = 0)

Records identified through database searching

ClinicalTrials.gov/NLM (n = 321)

Cochrane Library/Cochrane (n = 1,377)

Embase/Elsevier (n = 6,616)

PubMed/NLM (n = 11,423)

(n = 19,737 including Topic 2 = 4,017 and

KQ5 = 169, KQ6 = 50, KQ7 = 78, KQ8 = 15)

## Literature Search

Records removed

Duplicates identified during handsearching (n=169)

Duplicates identified by Endnotes (n = 7,829)

Duplicates identified manually in Endnotes (n = 11)

Duplicates identified by Covidence (n = 658)

Trials (n = 537)

(n = 9,204 including Topic 2 = 1,448)

Records not identified for Topic 2 (n = 8,259)

Records available for screening

(n = 10,901 including Topic 2 = 2,634)

Records identified for Topic 2 and excluded
(n = 2,542 including

KQ5 = 155, KQ6 = 45, KQ7 = 74, KQ8 = 12)

## Screening

Records available for second screening

(n = 92 including

KQ5 = 14, KQ5 = 5, KQ7 = 4, KQ8 = 3)

)

Records not identified for

KQ5, KQ6, KQ7, or KQ8
(n = 66)

KQ5, KQ6, KQ7, KQ8 Full-text articles assessed for eligibility
(n = 26)

Full-text articles excluded

(n = 22)

KQ5: 8 Wrong patient population

KQ5: 1 Wrong intervention

KQ5: 1 Wrong study design

KQ6: 3 Wrong patient population

KQ6::1 Study protocol only

KQ6::1 Wrong intervention

KQ7: 2 Wrong patient population

KQ7::1 Study protocol only

KQ7::1 Wrong intervention

KQ8: 1 Wrong KQ

KQ8::1 Wrong patient population

KQ8::1 Wrong intervention

## Eligibility

Included studies added to combined analysis

(n = 4 including KQ5 = 4, KQ6 = 0, KQ7 = 0, KQ8 = 0)

## Included

See chart KQ1-KQ16 for details on the

combined quantitative analysis (n=16)

See Literature Searches Summary document for search details.

**PRISMA Flow Diagram**

## KQ9 – KQ12

Additional articles identified through handsearching trials and citation searching

ClinicalTrials.gov/NLM (n = 15)

PubMed/NLM (n=60)

Citation searching (n = 293)

(n = 368 including Topic 2 = 65 and

KQ9, KQ10, KQ11, KQ12 = 0)

Records identified through database searching

ClinicalTrials.gov/NLM (n = 321)

Cochrane Library/Cochrane (n = 1,377)

Embase/Elsevier (n = 6,616)

PubMed/NLM (n = 11,423)

(n = 19,737 including Topic 2 = 4,017 and

KQ9 = 34, KQ10 = 10, KQ11 = 18, KQ12 = 16)

## Literature Search

Records removed

Duplicates identified during handsearching (n=169)

Duplicates identified by Endnotes (n = 7,829)

Duplicates identified manually in Endnotes (n = 11)

Duplicates identified by Covidence (n = 657)

Trials (n = 537)

(n = 9,204 including Topic 2 = 1,448)

Records not identified for Topic 2 (n = 8,259)

Records available for screening

(n = 10,901 including Topic 2 = 2,634)

Records identified for Topic 2 and excluded
(n = 2,542 including

KQ9 = 32, KQ10 = 10, KQ11 = 18, KQ12 = 16)

## Screening

Records available for second screening

(n = 92 including

KQ9 = 2, KQ10 = 2, KQ11 = 1, KQ12 = 1)

)

Records not identified for

KQ9, KQ10, KQ11, or KQ12
(n = 86)

## Eligibility

Full-text articles excluded

(n = 6)

KQ9: 1 Wrong intervention

KQ9: 1 Wrong patient population

KQ10: 1 Wrong intervention

KQ11: 1 Wrong patient population

KQ11: 1 Wrong intervention

KQ12: 1 Wrong intervention

KQ9, KQ10, KQ11, KQ12 Full-text articles assessed for eligibility
(n = 6)

Included studies added to combined analysis

(n = 0)

## Included

See chart KQ1-KQ16 for details on the

combined quantitative analysis (n=16)

See Literature Searches Summary document for search details.

**PRISMA Flow Diagram**

## KQ13 – KQ16

Additional articles identified through handsearching trials and citation searching

ClinicalTrials.gov/NLM (n = 15)

PubMed/NLM (n = 60)

Citation searching (n = 293)

(n = 368 including Topic 2 = 65 and

KQ13, KQ14, KQ15, KQ16 = 0)

Records identified through database searching

ClinicalTrials.gov/NLM (n = 321)

Cochrane Library/Cochrane (n = 1,377)

Embase/Elsevier (n = 6,616)

PubMed/NLM (n = 11,423)

(n = 19,737 including Topic 2 = 4,017 and

KQ13 = 8, KQ14 = 15, KQ15 = 6, KQ16 = 1)

## Literature Search

Records removed

Duplicates identified during handsearching (n=169)

Duplicates identified by Endnotes (n = 7,829)

Duplicates identified manually in Endnotes (n = 11)

Duplicates identified by Covidence (n = 658)

Trials (n = 537)

(n = 9,204 including Topic 2 = 1,448)

Records not identified for Topic 2 (n = 8,259)

Records available for screening

(n = 10,901 including Topic 2 = 2,634)

Records identified for Topic 2 and excluded
(n = 2,542 including

KQ13 = 8, KQ14 = 15, KQ15 = 6, KQ16 = 1)

## Screening

Records available for second screening

(n = 92 including

KQ13, KQ14, KQ15, KQ16 = 0)

Records not identified for

KQ13, KQ14, KQ15, or KQ16
(n = 92)

Full-text articles excluded

(n = 0)

KQ13, KQ14, KQ15, and KQ16 Full-text articles assessed for eligibility
(n = 0)

## Eligibility

Included studies added to combined analysis

(n = 0)

## Included

See chart KQ1-KQ16 for details on the

combined quantitative analysis (n=16)

See Literature Searches Summary document for search details.

**PRISMA Flow Diagram**

## KQ17

Additional articles identified through handsearching trials and citation searching

ClinicalTrials.gov/NLM (n = 15)

PubMed/NLM (n = 60)

Citation searching (n = 293)

(n = 368 including Topic 3 = 138

and KQ17 = 2)

Records identified through database searching

ClinicalTrials.gov/NLM (n = 321)

Cochrane Library/Cochrane (n = 1,377)

Embase/Elsevier (n = 6,616)

PubMed/NLM (n = 11,423)

(n = 19,737 including Topic 3 = 13,399

and KQ17 = 6,475)

## Literature Search

Records removed

Duplicates identified during handsearching (n=169)

Duplicates identified by Endnotes (n = 7,829)

Duplicates identified manually in Endnotes (n = 11)

Duplicates identified by Covidence (n = 658)

Trials (n = 537)

(n = 9,204 including Topic 3 = 5,747)

Records not identified for Topic 3 (n = 3,103)

Records available for screening

(n = 10,901 including Topic 3 = 7,790)

Records identified for Topic 3 and excluded
(n = 6,924 including KQ17 = 6,128)

## Screening

Records not identified for KQ17
(n = 519)

Records available for second screening

(n = 866 including KQ17 = 347)

Full-text articles excluded

(n = 151)

79 Wrong patient population

33 Wrong intervention

12 Wrong study design

8 Wrong setting

7 Wrong comparator

7 Wrong outcomes

4 Less than 10 patients

1 Wrong indication

KQ17 Full-text articles assessed for eligibility
(n = 349 including 2 hand searched additions)

## Eligibility

Studies included in appraisal/ guideline/recommendations
(n = 198)

## Included

Studies included in quantitative synthesis
(n = 82)

See Literature Searches Summary document for search details.

**Optimization of Perioperative Care in the Elderly**

**PRISMA Flow Diagram**

## KQ18

Records identified through database searching

ClinicalTrials.gov/NLM (n = 321)

Cochrane Library/Cochrane (n = 1,377)

Embase/Elsevier (n = 6,616)

PubMed/NLM (n = 11,423)

(n = 19,737 including Topic 3 = 13,399

and KQ18 = 2,317)

Additional articles identified through handsearching trials and citation searching

ClinicalTrials.gov/NLM (n = 15)

PubMed/NLM (n = 60)

Citation searching (n = 293)

(n = 368 including Topic 3 = 138

and KQ18 = 19)

## Literature Search

Records removed

Duplicates identified during handsearching (n=169)

Duplicates identified by Endnotes (n = 7,829)

Duplicates identified manually in Endnotes (n = 11)

Duplicates identified by Covidence (n = 658)

Trials (n = 537)

(n = 9,204 including Topic 3 = 5,747)

Records not identified for Topic 3 (n = 3,103)

Records available for screening

(n = 10,901 including Topic 3 = 7,790)

Records identified for Topic 3 and excluded
(n = 6,924 including KQ18 = 2,085)

## Screening

Records available for second screening

(n = 866 including KQ18 = 232)

Records not identified for KQ18
(n = 634)

Full-text articles excluded

(n = 152)

64 Wrong patient population

49 Wrong comparator

22 Wrong outcomes

10 Wrong subject

2 Wrong setting

2 Wrong study design

1 Wrong indication

1 Wrong route of administration

1 SR - Citations searched

KQ18 Full-text articles assessed for eligibility
(n = 251 including 19 hand searched additions)

## Eligibility

Studies included in appraisal/ guideline/recommendations
(n = 99)

## Included

Studies included in quantitative synthesis
(n = 18)

See Literature Searches Summary document for search details.

**PRISMA Flow Diagram**

## KQ19

Additional articles identified through handsearching trials and citation searching

ClinicalTrials.gov/NLM (n = 15)

PubMed/NLM (n = 60)

Citation searching (n = 293)

(n = 368 including Topic 3 = 138

and KQ19 = 10)

Records identified through database searching

ClinicalTrials.gov/NLM (n = 321)

Cochrane Library/Cochrane (n = 1,377)

Embase/Elsevier (n = 6,616)

PubMed/NLM (n = 11,423)

(n = 19,737 including Topic 3 = 13,399

and KQ19 = 3,034)

## Literature Search

Records removed

Duplicates identified during handsearching (n=169)

Duplicates identified by Endnotes (n = 7,829)

Duplicates identified manually in Endnotes (n = 11)

Duplicates identified by Covidence (n = 658)

Trials (n = 537)

(n = 9,204 including Topic 3 = 5,747 and KQ19 = 1)

Records not identified for Topic 3 (n = 3,103)

Records available for screening

(n = 10,901 including Topic 3 = 7,790)

Records identified for Topic 3 and excluded
(n = 6,924 including KQ19 = 2,820)

## Screening

Records not identified for KQ19
(n = 652)

Records available for second screening

(n = 866 including KQ19 = 214)

Full-text articles excluded

(n = 137)

44 Wrong patient population

38 Wrong comparator

28 Wrong outcomes

14 Wrong study design

6 Wrong intervention

6 Wrong setting

1 Adult population

KQ19 Full-text articles assessed for eligibility
(n = 223 including 10 hand searched additions minus 1 duplicate)

## Eligibility

Studies included in appraisal/ guideline/recommendations
(n = 86)

## Included

Studies included in quantitative synthesis
(n = 37)

See Literature Searches Summary document for search details.

**PRISMA Flow Diagram**

## KQ20

Additional articles identified through handsearching trials and citation searching

ClinicalTrials.gov/NLM (n = 15)

PubMed/NLM (n = 60)

Citation searching (n = 293)

(n = 368 including Topic 3 = 138

and KQ20 = 1)

Records identified through database searching

ClinicalTrials.gov/NLM (n = 321)

Cochrane Library/Cochrane (n = 1,377)

Embase/Elsevier (n = 6,616)

PubMed/NLM (n = 11,423)

(n = 19,737 including Topic 3 = 13,399

and KQ20 = 1,573)

## Literature Search

Records removed

Duplicates identified during handsearching (n=169)

Duplicates identified by Endnotes (n = 7,829)

Duplicates identified manually in Endnotes (n = 11)

Duplicates identified by Covidence (n = 658)

Trials (n = 537)

(n = 9,204 including Topic 3 = 5,747)

Records not identified for Topic 3 (n = 3,103)

Records available for screening

(n = 10,901 including Topic 3 = 7,790)

Records identified for Topic 3 and excluded
(n = 6,924 including KQ20 = 1,509)

## Screening

Records not identified for KQ20
(n = 802)

Records available for second screening

(n = 866 including KQ20 = 64)

## Eligibility

Full-text articles excluded

(n = 47)

28 Wrong patient population

15 Wrong intervention

3 Wrong study design

1 Wrong outcomes

KQ20 Full-text articles assessed for eligibility
(n = 65 including 1 hand searched addition)

Studies included in appraisal/ guideline/recommendations
(n = 18)

## Included

Studies included in quantitative synthesis
(n = 5)

See Literature Searches Summary document for search details.

**PRISMA Flow Diagram**

## KQ21

Records identified through database searching

ClinicalTrials.gov/NLM (n = 321)

Cochrane Library/Cochrane (n = 1,377)

Embase/Elsevier (n = 6,616)

PubMed/NLM (n = 11,423)

(n = 19,737 including Topic 4 = 2,321

and KQ21 = 1,415)

Additional articles identified through handsearching trials and citation searching

ClinicalTrials.gov/NLM (n = 15)

PubMed/NLM (n = 60)

Citation searching (n = 293)

(n = 368 including Topic 4 = 90

and KQ21 = 16)

## Literature Search

Records removed

Duplicates identified during handsearching (n=169)

Duplicates identified by Endnotes (n = 7,829)

Duplicates identified manually in Endnotes (n = 11)

Duplicates identified by Covidence (n = 658)

Trials (n = 537)

(n = 9,204 including Topic 4 = 908)

Records not identified for Topic 4 (n = 9,390)

Records available for screening

(n = 10,901 including Topic 4 = 1,503)

Records identified for Topic 4 and excluded
(n = 1,399 including KQ21 = 1,364)

## Screening

Records not identified for KQ21
(n = 53)

Records available for second screening

(n = 104 including KQ21 = 51)

Full-text articles excluded

(n = 19)

7 Wrong study design

5 Wrong patient population

4 Wrong outcomes

3 Wrong intervention

KQ21 Full-text articles assessed for eligibility
(n = 67 including 16 hand-searched additions)

## Eligibility

Studies included in appraisal/ guideline/recommendations
(n = 48)

## Included

Studies included in quantitative synthesis

(n = 9)

See Literature Searches Summary document for search details.

**PRISMA Flow Diagram**

## KQ22

Additional articles identified through handsearching trials and citation searching

ClinicalTrials.gov/NLM (n = 15)

PubMed/NLM (n = 60)

Citation searching (n = 293)

(n = 368 including Topic 4 = 90

and KQ22 = 3)

Records identified through database searching

ClinicalTrials.gov/NLM (n = 321)

Cochrane Library/Cochrane (n = 1,377)

Embase/Elsevier (n = 6,616)

PubMed/NLM (n = 11,423)

(n = 19,737 including Topic 4 = 2,321

and KQ22 = 284)

## Literature Search

=

Records removed

Duplicates identified during handsearching (n=169)

Duplicates identified by Endnotes (n = 7,829)

Duplicates identified manually in Endnotes (n = 11)

Duplicates identified by Covidence (n = 658)

Trials (n = 537)

(n = 9,204 including Topic 4 = 908)

Records not identified for Topic 4 (n = 9,390)

Records available for screening

(n = 10,901 including Topic 4 = 1,503)

Records identified for Topic 4 and excluded
(n = 1,399 including KQ22 = 275)

## Screening

Records available for second screening

(n = 104 including KQ22 = 9)

Records not identified for KQ22
(n = 95)

KQ22 Full-text articles assessed for eligibility
(n = 12 including 3 hand-searched additions)

Full-text articles excluded

(n = 7)

5 Wrong patient population

2 Wrong study design

## Eligibility

Studies included in appraisal/ guideline/recommendations
(n = 5)

## Included

Studies included in quantitative synthesis
(n = 2)

See Literature Searches Summary document for search details.

**PRISMA Flow Diagram**

## KQ23

Additional articles identified through handsearching trials and citation searching

ClinicalTrials.gov/NLM (n = 15)

PubMed/NLM (n = 60)

Citation searching (n = 293)

(n = 368 including Topic 4 = 90

and KQ23 = 9)

Records identified through database searching

ClinicalTrials.gov/NLM (n = 321)

Cochrane Library/Cochrane (n = 1,377)

Embase/Elsevier (n = 6,616)

PubMed/NLM (n = 11,423)

(n = 19,737 including Topic 4 = 2,321

and KQ23 = 505)

## Literature Search

Records removed

Duplicates identified during handsearching (n=169)

Duplicates identified by Endnotes (n = 7,829)

Duplicates identified manually in Endnotes (n = 11)

Duplicates identified by Covidence (n = 658)

Trials (n = 537)

(n = 9,204 including Topic 4 = 908)

Records not identified for Topic 4 (n = 9,390)

Records available for screening

(n = 10,901 including Topic 4 = 1,503)

Records identified for Topic 4 and excluded
(n = 1,399 including KQ23 = 497)

## Screening

Records not identified for KQ23
(n = 96)

Records available for second screening

(n = 104 including KQ23 = 8)

## Eligibility

Full-text articles excluded

(n = 12)

4 Wrong patient population

4 Wrong study design

2 Wrong indication

2 Wrong outcomes

KQ23 Full-text articles assessed for eligibility
(n = 17 including 9 hand searched additions)

Studies included in appraisal/ guideline/recommendations
(n = 5)

## Included

Studies included in quantitative synthesis
(n = 2)

See Literature Searches Summary document for search details.

**PRISMA Flow Diagram**

## KQ24

Records identified through database searching

ClinicalTrials.gov/NLM (n = 321)

Cochrane Library/Cochrane (n = 1,377)

Embase/Elsevier (n = 6,616)

PubMed/NLM (n = 11,423)

(n = 19,737 including Topic 4 = 2,321

and KQ24 = 117)

Additional articles identified through handsearching trials and citation searching

ClinicalTrials.gov/NLM (n = 15)

PubMed/NLM (n = 60)

Citation searching (n = 293)

(n = 368 including Topic 4 = 90

and KQ24 = 1)

## Literature Search

Records removed

Duplicates identified during handsearching (n=169)

Duplicates identified by Endnotes (n = 7,829)

Duplicates identified manually in Endnotes (n = 11)

Duplicates identified by Covidence (n = 658)

Trials (n = 537)

(n = 9,204 including Topic 4 = 908)

Records not identified for Topic 4 (n = 9,390)

Records available for screening

(n = 10,901 including Topic 4 = 1,503)

Records identified for Topic 4 and excluded
(n = 1,399 including KQ24 = 113)

## Screening

Records not identified for KQ24
(n = 100)

Records available for second screening

(n = 104 including KQ24 = 4)

KQ24 Full-text articles assessed for eligibility
(n = 5 including 1 hand-searched addition)

Full-text articles excluded

(n = 5)

3 Wrong study design

2 Wrong indication

## Eligibility

Studies included in appraisal/ guideline/recommendations
(n = 0)

## Included

Studies included in quantitative synthesis
(n = 0)

See Literature Searches Summary document for search details.

# Literature Flow Diagram

Records removed

Duplicates identified during handsearching (n=169)

Duplicates identified by Endnotes (n = 7,829)

Duplicates identified manually in Endnotes (n = 11)

Duplicates identified by Covidence (n = 658)

Trials (n = 537)

(n = 9,204)

Additional articles identified through handsearching trials and citation searching

ClinicalTrials.gov/NLM (n = 15)

PubMed/NLM (n = 60)

Citation searching (n = 293)

(n = 368)

Records identified through database searching

ClinicalTrials.gov/NLM (n = 321)

Cochrane Library/Cochrane (n = 1,377)

Embase/Elsevier (n = 6,616)

PubMed/NLM (n = 11,423)

(n = 19,737)

Unique Records available for screening (n = 10,901)

Records identified for Topic 4 Records and screened

(n = 1,503)

Records identified for Topic 2 and screened

(n = 2,634)

Records identified for Topic 3 Records and screened

(n = 7,790)

Topic 4 Records excluded
(n = 1,399)

Topic 2 Records excluded
(n = 2,542)

Topic 3 Records excluded
(n = 6,924)

Records available for second screening

(n = 92)

Records available for second screening

(n = 104)

Records available for second screening

(n = 866)

KQ20

(n = 64)

(n = 1)

(n = 65)

(n = 47)

(n = 18)

(n = 5)

KQ19

(n = 214)

(n = 9)

(n = 223)

(n = 137)

(n = 86)

(n = 37)

KQ 17

(n = 347)

(n = 2)

(n = 349)

(n = 151)

(n = 198)

(n = 82)

KQ18

(n = 232)

(n = 19)

(n = 251)

(n = 152)

(n= 99)

(n = 18)

Topic 3 records for:

Records included:

Additions:

Records screened:

Records excluded:

Total studies included:

Studies in the analysis:

KQ5 KQ6 KQ7 KQ8

(n = 14) (n = 5) (n = 4) (n = 3)

(n = 0) (n = 0) (n = 0) (n = 0)

(n = 14) (n = 5) (n = 4) (n = 3)

(n = 10) (n = 5) (n = 4) (n = 3)

(n = 4) (n = 0) (n = 0) (n = 0)

(n = 0)

Topic 2 records for:

Records included:

Additions:

Records screened:

Records excluded:

Studies included:

KQ1 KQ2 KQ3 KQ4

(n = 62) (n = 22) (n = 25) (n = 14)

(n = 8) (n = 2) (n = 3) (n = 1)

(n = 70) (n = 24) (n = 28) (n = 15)

(n = 57) (n = 20) (n = 25) (n = 14)

(n = 13) (n = 4) (n = 3) (n = 1)

(n = 0)

Topic 2 records for:

Records included:

Additions:

Records screened:

Records excluded:

Studies included:

KQ13-KQ16

(n = 0)

(n = 0)

(n = 0)

(n = 0)

(n = 0)

KQ9-KQ10 KQ11-KQ12

(n = 2) (n = 1)

(n = 0) (n = 0)

(n = 2) (n = 1)

(n = 2) (n = 1)

(n = 0) (n = 0)

(n = 0)

KQ24

(n = 4)

(n = 1)

(n = 5)

(n = 5)

(n = 0)

(n = 0)

KQ23

(n = 8)

(n = 9)

(n = 17)

(n = 12)

(n = 5)

(n = 2)

KQ22

(n = 9)

(n = 3)

(n = 12)

(n = 7)

(n = 5)

(n = 2)

Topic 4 records for:

Records included:

Additions:

Records screened:

Records excluded:

Total studies included:

Studies in the analysis:

Topic 2 records for: KQ1-KQ16

Studies included: (n = 25)

Duplicates removed: (n = 9)

Total studies included: (n = 16)

Studies in the analysis: (n = 16)

KQ21

(n = 51)

(n = 16)

(n = 67)

(n = 19)

(n = 48)

(n = 9)
