# Supplementary material for: EAES/SAGES evidence-based recommendations and expert consensus on optimization of perioperative care in older adults
Source: Surg Endosc. 2024 Jun 28;38(8):4104–26. doi: 10.1007/s00464-024-10977-7 (PMC11289045; doi:10.1007/s00464-024-10977-7)
Supplement: Supplementary file 3 — Supplement 3 Quality Assessment for KQ1-KQ16 Supplementary file3 (DOCX 21 KB) [file 464_2024_10977_MOESM3_ESM.docx]

Supplement 3: Quality Assessment for KQ1-KQ16

| Table 1. Risk of bias for the observational studies included under KQ1-KQ16 as assessed by a modified Newcastle Ottawa Scale. | | | |
| --- | --- | --- | --- |
| Achilli 2020 | NOS | Moderate |  |
| Chia 2016 | NOS | Low |  |
| Indrakusuma 2015 | NOS | Low |  |
| Janssen 2019/2020 | NOS | Low |  |
| McDonald 2018 | NOS | Moderate |  |
| Souwer 2018 | NOS | Low |  |
| van der Vlies 2020 | NOS | Low |  |
| Watanabe 2020 | NOS | High |  |
| Yamamoto 2017 | NOS | High |  |
|  |  |  |  |
| Table 2. Risk of bias for the RCTs included under KQ1-KQ16 as assessed by a modified Cochrane Risk of Bias tool. | | | |
| Barberan-Garcia 2018 | Cochrane Rob2 | Low |  |
| Carli 2020 | Cochrane Rob2 | Low |  |
| Janssen 2021 | Cochrane Rob2 | High |  |
| Karlsson 2019 | Cochrane Rob2 | High |  |
| Keeler 2017 | Cochrane Rob2 | Low |  |
| Okazaki 2013 | Cochrane Rob2 | High |  |
| Ommundsen 2018 | Cochrane Rob2 | High |  |
