# Supplementary material for: EAES/SAGES evidence-based recommendations and expert consensus on optimization of perioperative care in older adults
Source: Surg Endosc. 2024 Jun 28;38(8):4104–26. doi: 10.1007/s00464-024-10977-7 (PMC11289045; doi:10.1007/s00464-024-10977-7)
Supplement: Supplementary file 4 — Supplement 4 Evidence to Decision (EtD) tables for KQ17-KQ24 Supplementary file4 (DOCX 103 KB) [file 464_2024_10977_MOESM4_ESM.docx]

EAES/SAGES Evidence-Based Guidelines on Optimization of Perioperative Care in Older Adults

# Supplement 4 - Evidence to Decision (EtD) Tables for KQ17-KQ24

# QUESTION #17

| **Should laparoscopic vs. open colorectal surgery be used for the elderly?** | |
| --- | --- |
| **POPULATION:** | the elderly |
| **INTERVENTION:** | laparoscopic |
| **COMPARISON:** | open colorectal surgery |
| **MAIN OUTCOMES:** | Complications; Length of stay; Readmission; |
| **SETTING:** | Inpatient (EU/USA) |
| **PERSPECTIVE:** |  |
| **BACKGROUND:** |  |
| **CONFLICT OF INTERESTS:** |  |

# ASSESSMENT

| Problem Is the problem a priority? | | |
| --- | --- | --- |
| JUDGEMENT | RESEARCH EVIDENCE | ADDITIONAL CONSIDERATIONS |
| ○ No ○ Probably no ○ Probably yes ● Yes ○ Varies ○ Don't know |  |  |

| Desirable Effects How substantial are the desirable anticipated effects? | | |
| --- | --- | --- |
| JUDGEMENT | RESEARCH EVIDENCE | ADDITIONAL CONSIDERATIONS |
| ○ Trivial ○ Small ● Moderate ○ Large ○ Varies ○ Don't know | \| **Outcomes** \| **№ of participants (studies) Follow-up** \| **Certainty of the evidence (GRADE)** \| **Relative effect (95% CI)** \| **Anticipated absolute effects^*^ (95% CI)** \| \| \| --- \| --- \| --- \| --- \| --- \| --- \| \| **Risk with open colorectal surgery** \| **Risk difference with laparoscopic** \| \| Complications \| 131241 (81 observational studies) \| ⨁◯◯◯ Very low^a^ \| **RR 0.70** (0.64 to 0.77) \| Study population \| \| \| 337 per 1,000 \| **101 fewer per 1,000** (121 fewer to 78 fewer) \| \| Length of stay \| 87465 (72 observational studies) \| ⨁◯◯◯ Very low^a^ \| - \| The mean length of stay was **0** days \| MD **2.48 days fewer** (2.9 fewer to 2.05 fewer) \| \| Readmission \| 16075 (7 observational studies) \| ⨁◯◯◯ Very low^a,b^ \| **RR 0.85** (0.54 to 1.34) \| Study population \| \| \| 100 per 1,000 \| **15 fewer per 1,000** (46 fewer to 34 more) \|  1. Contains unmatched data 2. Iˆ2=82% |  |
| Undesirable Effects How substantial are the undesirable anticipated effects? | | |
| JUDGEMENT | RESEARCH EVIDENCE | ADDITIONAL CONSIDERATIONS |
| ○ Large ○ Moderate ○  Small ●Trivial ○ Varies ○ Don't know | None of the outcomes showed undesirable effects with the intervention. |  |

| Certainty of evidence What is the overall certainty of the evidence of effects? | | |
| --- | --- | --- |
| JUDGEMENT | RESEARCH EVIDENCE | ADDITIONAL CONSIDERATIONS |
| ● Very low ○ Low ​​○Moderate ○ High ○ No included studies | \| **Outcomes** \| **Importance** \| **Certainty of the evidence (GRADE)** \| \| --- \| --- \| --- \| \| Complications \| CRITICAL \| ⨁◯◯◯ Very low^a^ \| \| Length of stay \| CRITICAL \| ⨁◯◯◯ Very low^a^ \| \| Readmission \| CRITICAL \| ⨁◯◯◯ Very low^a,b^ \|  1. Contains unmatched data 2. Iˆ2=82% |  |
| Values Is there important uncertainty about or variability in how much people value the main outcomes? | | |
| JUDGEMENT | RESEARCH EVIDENCE | ADDITIONAL CONSIDERATIONS |
| ○ Important uncertainty or variability ● Possibly important uncertainty or variability ○ Probably no important uncertainty or variability ○ No important uncertainty or variability |  |  |
| Balance of effects Does the balance between desirable and undesirable effects favor the intervention or the comparison? | | |
| JUDGEMENT | RESEARCH EVIDENCE | ADDITIONAL CONSIDERATIONS |
| ○ Favors the comparison ○ Probably favors the comparison ○ Does not favor either the intervention or the comparison ​​● Probably favors the intervention ○ Favors the intervention ○ Varies ○ Don't know |  |  |
| Acceptability Is the intervention acceptable to key stakeholders? | | |
| JUDGEMENT | RESEARCH EVIDENCE | ADDITIONAL CONSIDERATIONS |
| ○ No ○ Probably no ○ Probably yes ● Yes ○ Varies ○ Don't know |  |  |
| Feasibility Is the intervention feasible to implement? | | |
| JUDGEMENT | RESEARCH EVIDENCE | ADDITIONAL CONSIDERATIONS |
| ○ No ○ Probably no ○ Probably yes ● Yes ○ Varies ○ Don't know |  |  |

# SUMMARY OF JUDGEMENTS

|  | **JUDGEMENT** | | | | | | |
| --- | --- | --- | --- | --- | --- | --- | --- |
| **PROBLEM** | No | Probably no | Probably yes | **Yes** |  | Varies | Don't know |
| **DESIRABLE EFFECTS** | Trivial | Small | **Moderate** | Large |  | Varies | Don't know |
| **UNDESIRABLE EFFECTS** | Large | Moderate | Small | **Trivial** |  | Varies | Don't know |
| **CERTAINTY OF EVIDENCE** | **Very low** | Low | Moderate | High |  |  | No included studies |
| **VALUES** | Important uncertainty or variability | **Possibly important uncertainty or variability** | Probably no important uncertainty or variability | No important uncertainty or variability |  |  |  |
| **BALANCE OF EFFECTS** | Favors the comparison | Probably favors the comparison | Does not favor either the intervention or the comparison | **Probably favors the intervention** | Favors the intervention | Varies | Don't know |
| **ACCEPTABILITY** | No | Probably no | Probably yes | **Yes** |  | Varies | Don't know |
| **FEASIBILITY** | No | Probably no | Probably yes | **Yes** |  | Varies | Don't know |

# TYPE OF RECOMMENDATION

| Strong recommendation against the intervention | Conditional recommendation against the intervention | Conditional recommendation for either the intervention or the comparison | Conditional recommendation for the intervention | Strong recommendation for the intervention |
| --- | --- | --- | --- | --- |
| ○ | ○ | ○ | ● | ○ |

# CONCLUSIONS

| Recommendation |
| --- |
|  |
| Justification |

| Subgroup considerations |
| --- |
|  |
| Implementation considerations |
|  |

| Monitoring and evaluation |
| --- |
|  |
| Research priorities |

EAES/SAGES Evidence-Based Guidelines on Optimization of Perioperative Care in Older Adults

# QUESTION #18

| **Should laparoscopic vs. open upper GI surgery be used for the elderly?** | |
| --- | --- |
| **Population:** | the elderly |
| **Intervention:** | laparoscopic |
| **Comparison:** | open upper GI surgery |
| **Main outcomes:** | Complications; Length of stay; Readmission; |
| **Setting:** | Inpatient (EU/USA) |
| **Perspective:** |  |
| **Background:** |  |
| **Conflict of interests:** |  |

# Assessment

| Problem Is the problem a priority? | | |
| --- | --- | --- |
| Judgement | Research evidence | Additional considerations |
| ○ No ○ Probably no ○ Probably yes ○ Yes ○ Varies ○ Don't know |  |  |

| Desirable Effects How substantial are the desirable anticipated effects? | | |
| --- | --- | --- |
| Judgement | Research evidence | Additional considerations |
| ○ Trivial ○ Small ○ Moderate ○ Large ○ Varies ○ Don't know | \| **Outcomes** \| **№ of participants (studies) Follow-up** \| **Certainty of the evidence (GRADE)** \| **Relative effect (95% CI)** \| **Anticipated absolute effects^*^ (95% CI)** \| \| \| --- \| --- \| --- \| --- \| --- \| --- \| \| **Risk with open upper GI surgery** \| **Risk difference with laparoscopic** \| \| Complications \| 10431 (18 observational studies) \| ⨁◯◯◯ Very low^a^ \| **RR 0.68** (0.56 to 0.82) \| Study population \| \| \| 222 per 1,000 \| **71 fewer per 1,000** (98 fewer to 40 fewer) \| \| Length of stay \| 4134 (14 observational studies) \| ⨁◯◯◯ Very low^a^ \| - \| The mean length of stay was **0** days \| MD **2.84 days fewer** (4.24 fewer to 1.45 fewer) \| \| Readmission \| 328 (2 observational studies) \| ⨁◯◯◯ Very low^b^ \| **RR 0.40** (0.15 to 1.10) \| Study population \| \| \| 78 per 1,000 \| **47 fewer per 1,000** (66 fewer to 8 more) \|  1. Contains unmatched data 2. Interval estimates cross statistical and clinical significance thresholds |  |
| Undesirable Effects How substantial are the undesirable anticipated effects? | | |
| Judgement | Research evidence | Additional considerations |
| ○ Large ○ Moderate ○ Small ○ Trivial ○ Varies ○ Don't know | There were no outcomes with undesirable effects for the intervention. |  |

| Certainty of evidence What is the overall certainty of the evidence of effects? | | |
| --- | --- | --- |
| Judgement | Research evidence | Additional considerations |
| ○ Very low ○ Low ○ Moderate ○ High ○ No included studies | \| **Outcomes** \| **Importance** \| **Certainty of the evidence (GRADE)** \| \| --- \| --- \| --- \| \| Complications \| CRITICAL \| ⨁◯◯◯ Very low^a^ \| \| Length of stay \| CRITICAL \| ⨁◯◯◯ Very low^a^ \| \| Readmission \| CRITICAL \| ⨁◯◯◯ Very low^b^ \|  1. Contains unmatched data 2. Interval estimates cross statistical and clinical significance thresholds |  |
| Values Is there important uncertainty about or variability in how much people value the main outcomes? | | |
| Judgement | Research evidence | Additional considerations |
| ○ Important uncertainty or variability ○ Possibly important uncertainty or variability ○ Probably no important uncertainty or variability ○ No important uncertainty or variability |  |  |
| Balance of effects Does the balance between desirable and undesirable effects favor the intervention or the comparison? | | |
| Judgement | Research evidence | Additional considerations |
| ○ Favors the comparison ○ Probably favors the comparison ○ Does not favor either the intervention or the comparison ○ Probably favors the intervention ○ Favors the intervention ○ Varies ○ Don't know |  |  |

| Acceptability Is the intervention acceptable to key stakeholders? | | |
| --- | --- | --- |
| Judgement | Research evidence | Additional considerations |
| ○ No ○ Probably no ○ Probably yes ○ Yes ○ Varies ○ Don't know |  |  |
| Feasibility Is the intervention feasible to implement? | | |
| Judgement | Research evidence | Additional considerations |
| ○ No ○ Probably no ○ Probably yes ○ Yes ○ Varies ○ Don't know |  |  |

# Summary of judgements

|  | **Judgement** | | | | | | |
| --- | --- | --- | --- | --- | --- | --- | --- |
| **Problem** | No | Probably no | Probably yes | Yes |  | Varies | Don't know |
| **Desirable Effects** | Trivial | Small | Moderate | Large |  | Varies | Don't know |
| **Undesirable Effects** | Large | Moderate | Small | Trivial |  | Varies | Don't know |
| **Certainty of evidence** | Very low | Low | Moderate | High |  |  | No included studies |
| **Values** | Important uncertainty or variability | Possibly important uncertainty or variability | Probably no important uncertainty or variability | No important uncertainty or variability |  |  |  |
| **Balance of effects** | Favors the comparison | Probably favors the comparison | Does not favor either the intervention or the comparison | Probably favors the intervention | Favors the intervention | Varies | Don't know |
| **Acceptability** | No | Probably no | Probably yes | Yes |  | Varies | Don't know |
| **Feasibility** | No | Probably no | Probably yes | Yes |  | Varies | Don't know |

# Type of recommendation

| Strong recommendation against the intervention | Conditional recommendation against the intervention | Conditional recommendation for either the intervention or the comparison | Conditional recommendation for the intervention | Strong recommendation for the intervention |
| --- | --- | --- | --- | --- |
| ○ | ○ | ○ | ○ | ○ |

# Conclusions

| Recommendation |
| --- |
|  |
| Justification |

| Subgroup considerations |
| --- |
|  |
| Implementation considerations |
|  |
| Monitoring and evaluation |
|  |
| Research priorities |
|  |

EAES/SAGES Evidence-Based Guidelines on Optimization of Perioperative Care in Older Adults

# QUESTION #19

| **Should laparoscopic vs. open HPB surgery be used for the elderly?** | |
| --- | --- |
| **POPULATION:** | the elderly |
| **INTERVENTION:** | laparoscopic |
| **COMPARISON:** | open HPB surgery |
| **MAIN OUTCOMES:** | Complications; Length of stay; Readmission; |
| **SETTING:** | Inpatient (EU/USA) |
| **PERSPECTIVE:** |  |
| **BACKGROUND:** |  |
| **CONFLICT OF INTERESTS:** |  |

# ASSESSMENT

| Problem Is the problem a priority? | | |
| --- | --- | --- |
| JUDGEMENT | RESEARCH EVIDENCE | ADDITIONAL CONSIDERATIONS |
| ○ No ○ Probably no ○ Probably yes ○ Yes ○ Varies ○ Don't know |  |  |

| Desirable Effects How substantial are the desirable anticipated effects? | | |
| --- | --- | --- |
| JUDGEMENT | RESEARCH EVIDENCE | ADDITIONAL CONSIDERATIONS |
| ○ Trivial ○ Small ○ Moderate ○ Large ○ Varies ○ Don't know | \| **Outcomes** \| **№ of participants (studies) Follow-up** \| **Certainty of the evidence (GRADE)** \| **Relative effect (95% CI)** \| **Anticipated absolute effects^*^ (95% CI)** \| \| \| --- \| --- \| --- \| --- \| --- \| --- \| \| **Risk with open HPB surgery** \| **Risk difference with laparoscopic** \| \| Complications \| 6222 (36 observational studies) \| ⨁⨁◯◯ Low^a^ \| **RR 0.60** (0.50 to 0.71) \| Study population \| \| \| 305 per 1,000 \| **122 fewer per 1,000** (152 fewer to 88 fewer) \| \| Length of stay \| 5862 (25 observational studies) \| ⨁⨁◯◯ Low \| - \| The mean length of stay was **0** days \| MD **3.85 days lower** (5.12 lower to 2.59 lower) \| \| Readmission \| 2180 (5 observational studies) \| ⨁◯◯◯ Very low^b^ \| **RR 0.83** (0.59 to 1.17) \| Study population \| \| \| 123 per 1,000 \| **21 fewer per 1,000** (50 fewer to 21 more) \|  1. Complications: Iˆ2=68%, however interval estimates beyond decision thresholds 2. Interval estimates cross decision thresholds |  |
| Undesirable Effects How substantial are the undesirable anticipated effects? | | |
| JUDGEMENT | RESEARCH EVIDENCE | ADDITIONAL CONSIDERATIONS |
| ○ Large ○ Moderate ○ Small ○ Trivial ○ Varies ○ Don't know | There were no outcomes with undesirable effects for the intervention. |  |

| Certainty of evidence What is the overall certainty of the evidence of effects? | | |
| --- | --- | --- |
| JUDGEMENT | RESEARCH EVIDENCE | ADDITIONAL CONSIDERATIONS |
| ○ Very low ○ Low ○ Moderate ○ High ○ No included studies | \| **Outcomes** \| **Importance** \| **Certainty of the evidence (GRADE)** \| \| --- \| --- \| --- \| \| Complications \| CRITICAL \| ⨁⨁◯◯ Low^a^ \| \| Length of stay \| CRITICAL \| ⨁⨁◯◯ Low \| \| Readmission \| CRITICAL \| ⨁◯◯◯ Very low^b^ \|  1. Complications: Iˆ2=68%, however interval estimates beyond decision thresholds 2. Interval estimates cross decision thresholds |  |
| Values Is there important uncertainty about or variability in how much people value the main outcomes? | | |
| JUDGEMENT | RESEARCH EVIDENCE | ADDITIONAL CONSIDERATIONS |
| ○ Important uncertainty or variability ○ Possibly important uncertainty or variability ○ Probably no important uncertainty or variability ○ No important uncertainty or variability |  |  |
| Balance of effects Does the balance between desirable and undesirable effects favor the intervention or the comparison? | | |
| JUDGEMENT | RESEARCH EVIDENCE | ADDITIONAL CONSIDERATIONS |
| ○ Favors the comparison ○ Probably favors the comparison ○ Does not favor either the intervention or the comparison ○ Probably favors the intervention ○ Favors the intervention ○ Varies ○ Don't know |  |  |
| Acceptability Is the intervention acceptable to key stakeholders? | | |
| JUDGEMENT | RESEARCH EVIDENCE | ADDITIONAL CONSIDERATIONS |
| ○ No ○ Probably no ○ Probably yes ○ Yes ○ Varies ○ Don't know |  |  |
| Feasibility Is the intervention feasible to implement? | | |
| JUDGEMENT | RESEARCH EVIDENCE | ADDITIONAL CONSIDERATIONS |
| ○ No ○ Probably no ○ Probably yes ○ Yes ○ Varies ○ Don't know |  |  |

# SUMMARY OF JUDGEMENTS

|  | **JUDGEMENT** | | | | | | |
| --- | --- | --- | --- | --- | --- | --- | --- |
| **PROBLEM** | No | Probably no | Probably yes | Yes |  | Varies | Don't know |
| **DESIRABLE EFFECTS** | Trivial | Small | Moderate | Large |  | Varies | Don't know |
| **UNDESIRABLE EFFECTS** | Large | Moderate | Small | Trivial |  | Varies | Don't know |
| **CERTAINTY OF EVIDENCE** | Very low | Low | Moderate | High |  |  | No included studies |
| **VALUES** | Important uncertainty or variability | Possibly important uncertainty or variability | Probably no important uncertainty or variability | No important uncertainty or variability |  |  |  |
| **BALANCE OF EFFECTS** | Favors the comparison | Probably favors the comparison | Does not favor either the intervention or the comparison | Probably favors the intervention | Favors the intervention | Varies | Don't know |
| **ACCEPTABILITY** | No | Probably no | Probably yes | Yes |  | Varies | Don't know |
| **FEASIBILITY** | No | Probably no | Probably yes | Yes |  | Varies | Don't know |

# TYPE OF RECOMMENDATION

| Strong recommendation against the intervention | Conditional recommendation against the intervention | Conditional recommendation for either the intervention or the comparison | Conditional recommendation for the intervention | Strong recommendation for the intervention |
| --- | --- | --- | --- | --- |
| ○ | ○ | ○ | ○ | ○ |

# CONCLUSIONS

| Recommendation |
| --- |
|  |
| Justification |

| Subgroup considerations |
| --- |
|  |
| Implementation considerations |

| Monitoring and evaluation |
| --- |
|  |
| Research priorities |
|  |

EAES/SAGES Evidence-Based Guidelines on Optimization of Perioperative Care in Older Adults

# QUESTION #20

| **Should laparoscopic vs. open hernia surgery be used for the elderly?** | |
| --- | --- |
| **Population:** | the elderly |
| **Intervention:** | laparoscopic |
| **Comparison:** | open hernia surgery |
| **Main outcomes:** | Complications; Length of stay; |
| **Setting:** | Inpatient (EU/USA) |
| **Perspective:** |  |
| **Background:** |  |
| **Conflict of interests:** |  |

# Assessment

| Problem Is the problem a priority? | | |
| --- | --- | --- |
| Judgement | Research evidence | Additional considerations |
| ○ No ○ Probably no ○ Probably yes ○ Yes ○ Varies ○ Don't know |  |  |

| Desirable Effects How substantial are the desirable anticipated effects? | | |
| --- | --- | --- |
| Judgement | Research evidence | Additional considerations |
| ○ Trivial ○ Small ○ Moderate ○ Large ○ Varies ○ Don't know | \| **Outcomes** \| **№ of participants (studies) Follow-up** \| **Certainty of the evidence (GRADE)** \| **Relative effect (95% CI)** \| **Anticipated absolute effects^*^ (95% CI)** \| \| \| --- \| --- \| --- \| --- \| --- \| --- \| \| **Risk with open hernia surgery** \| **Risk difference with laparoscopic** \| \| Complications \| 29285 (5 observational studies) \| ⨁◯◯◯ Very low^a,b,c^ \| **RR 0.68** (0.38 to 1.22) \| Study population \| \| \| 93 per 1,000 \| **30 fewer per 1,000** (58 fewer to 20 more) \| \| Length of stay \| 29040 (5 observational studies) \| ⨁◯◯◯ Very low^d,e,f^ \| - \| The mean length of stay was **0** days \| MD **5.08 days fewer** (10.6 fewer to 0.44 more) \|  1. High risk of bias in 2 out of 3 studies 2. Iˆ2=67% 3. Interval estimates cross decision thresholds 4. High or unclear risk of bias in 4 out of 5 studies 5. Iˆ2=99% 6. Interval estimates cross decision thresholds |  |
| Undesirable Effects How substantial are the undesirable anticipated effects? | | |
| Judgement | Research evidence | Additional considerations |
| ○ Large ○ Moderate ○ Small ○ Trivial ○ Varies ○ Don't know | There were no outcomes with undesirable effects for the intervention, however, none of the included studies reported readmission rates, which was a critical outcome. |  |

| Certainty of evidence What is the overall certainty of the evidence of effects? | | |
| --- | --- | --- |
| Judgement | Research evidence | Additional considerations |
| ○ Very low ○ Low ○ Moderate ○ High ○ No included studies | \| **Outcomes** \| **Importance** \| **Certainty of the evidence (GRADE)** \| \| --- \| --- \| --- \| \| Complications \| CRITICAL \| ⨁◯◯◯ Very low^a,b,c^ \| \| Length of stay \| CRITICAL \| ⨁◯◯◯ Very low^d,e,f^ \|  1. High risk of bias in 2 out of 3 studies 2. Iˆ2=67% 3. Interval estimates cross decision thresholds 4. High or unclear risk of bias in 4 out of 5 studies 5. Iˆ2=99% 6. Interval estimates cross decision thresholds |  |
| Values Is there important uncertainty about or variability in how much people value the main outcomes? | | |
| Judgement | Research evidence | Additional considerations |
| ○ Important uncertainty or variability ○ Possibly important uncertainty or variability ○ Probably no important uncertainty or variability ○ No important uncertainty or variability |  |  |
| Balance of effects Does the balance between desirable and undesirable effects favor the intervention or the comparison? | | |
| Judgement | Research evidence | Additional considerations |
| ○ Favors the comparison ○ Probably favors the comparison ○ Does not favor either the intervention or the comparison ○ Probably favors the intervention ○ Favors the intervention ○ Varies ○ Don't know |  |  |

| Acceptability Is the intervention acceptable to key stakeholders? | | |
| --- | --- | --- |
| Judgement | Research evidence | Additional considerations |
| ○ No ○ Probably no ○ Probably yes ○ Yes ○ Varies ○ Don't know |  |  |
| Feasibility Is the intervention feasible to implement? | | |
| Judgement | Research evidence | Additional considerations |
| ○ No ○ Probably no ○ Probably yes ○ Yes ○ Varies ○ Don't know |  |  |

# Summary of judgements

|  | **Judgement** | | | | | | |
| --- | --- | --- | --- | --- | --- | --- | --- |
| **Problem** | No | Probably no | Probably yes | Yes |  | Varies | Don't know |
| **Desirable Effects** | Trivial | Small | Moderate | Large |  | Varies | Don't know |
| **Undesirable Effects** | Large | Moderate | Small | Trivial |  | Varies | Don't know |
| **Certainty of evidence** | Very low | Low | Moderate | High |  |  | No included studies |
| **Values** | Important uncertainty or variability | Possibly important uncertainty or variability | Probably no important uncertainty or variability | No important uncertainty or variability |  |  |  |
| **Balance of effects** | Favors the comparison | Probably favors the comparison | Does not favor either the intervention or the comparison | Probably favors the intervention | Favors the intervention | Varies | Don't know |
| **Acceptability** | No | Probably no | Probably yes | Yes |  | Varies | Don't know |
| **Feasibility** | No | Probably no | Probably yes | Yes |  | Varies | Don't know |

# Type of recommendation

| Strong recommendation against the intervention | Conditional recommendation against the intervention | Conditional recommendation for either the intervention or the comparison | Conditional recommendation for the intervention | Strong recommendation for the intervention |
| --- | --- | --- | --- | --- |
| ○ | ○ | ○ | ○ | ○ |

# Conclusions

| Recommendation |
| --- |
|  |
| Justification |
|  |
| Subgroup considerations |
|  |
| Implementation considerations |
|  |
| Monitoring and evaluation |
|  |
| Research priorities |
|  |

EAES/SAGES Evidence-Based Guidelines on Optimization of Perioperative Care in Older Adults

# QUESTION #21

| **Should ERAS vs. Conventional Care be used for Colorectal Surgery in Elderly Patients ?** | |
| --- | --- |
| **Population:** | Colorectal Surgery in Elderly Patients |
| **Intervention:** | ERAS |
| **Comparison:** | Conventional Care |
| **Main outcomes:** | Complications (30d); Hospital Length of Stay (LOS); Readmissions; |
| **Setting:** |  |
| **Perspective:** |  |
| **Background:** |  |
| **Conflict of interests:** |  |

# Assessment

| Problem Is the problem a priority? | | |
| --- | --- | --- |
| Judgement | Research evidence | Additional considerations |
| ○ No ○ Probably no ○ Probably yes ● Yes ○ Varies ○ Don't know |  |  |
| Desirable Effects How substantial are the desirable anticipated effects? | | |
| Judgement | Research evidence | Additional considerations |
| ○ Trivial ○ Small ○ Moderate ● Large ○ Varies ○ Don't know |  |  |

| Undesirable Effects How substantial are the undesirable anticipated effects? | | |
| --- | --- | --- |
| Judgement | Research evidence | Additional considerations |
| ○ Large ○ Moderate ● Small ○ Trivial ○ Varies ○ Don't know | \| **Outcomes** \| **№ of participants (studies) Follow-up** \| **Certainty of the evidence (GRADE)** \| **Relative effect (95% CI)** \| **Anticipated absolute effects^*^ (95% CI)** \| \| \| --- \| --- \| --- \| --- \| --- \| --- \| \| **Risk with Conventional Care** \| **Risk difference with ERAS** \| \| Readmissions \| 150 (1 RCT) \| ⨁⨁◯◯ Low^a^ \| **OR 1.22** (0.35 to 4.18) \| Study population \| \| \| 67 per 1,000 \| **13 more per 1,000** (42 fewer to 163 more) \|  1. Very small sample size and even smaller event rate in conjunction with a confidence interval that crosses multiple standards of clinical meaningfulness. The estimated effects ranges from moderate benefit to large harms. | Must consider patients that live far from operative institution and that may be a greater harm than one who lives close by  Any positive impact should be considered to patients, so should not be trivial  Readmission practices ranges in other countries and can be a huge burden to patients |
| Certainty of evidence What is the overall certainty of the evidence of effects? | | |
| Judgement | Research evidence | Additional considerations |
| ○ Very low ○ Low ● Moderate ○ High ○ No included studies | \| **Outcomes** \| **Importance** \| **Certainty of the evidence (GRADE)** \| \| --- \| --- \| --- \| \| Complications (30d) \| CRITICAL \| ⨁⨁⨁◯ Moderate^a^ \| \| Hospital Length of Stay (LOS) \| CRITICAL \| ⨁⨁⨁◯ Moderate^b^ \| \| Readmissions \| CRITICAL \| ⨁⨁◯◯ Low^c^ \|  1. The number of events is below the threshold of 300, hence the certainty was downgraded for imprecision. 2. Two trials had a high risk of boas due to lack of reporting of loss to follow up, missingness, and planned statistical analysis. 3. Very small sample size and even smaller event rate in conjunction with a confidence interval that crosses multiple standards of clinical meaningfulness. The estimated effects ranges from moderate benefit to large harms. |  |
| Values Is there important uncertainty about or variability in how much people value the main outcomes? | | |
| Judgement | Research evidence | Additional considerations |
| ○ Important uncertainty or variability ○ Possibly important uncertainty or variability ● Probably no important uncertainty or variability ○ No important uncertainty or variability |  | Because there was no patient representation, experts thought there may be patients that would value different outcomes. |
| Balance of effects Does the balance between desirable and undesirable effects favor the intervention or the comparison? | | |
| Judgement | Research evidence | Additional considerations |
| ○ Favors the comparison ○ Probably favors the comparison ○ Does not favor either the intervention or the comparison ○ Probably favors the intervention ● Favors the intervention ○ Varies ○ Don't know |  |  |
| Acceptability Is the intervention acceptable to key stakeholders? | | |
| Judgement | Research evidence | Additional considerations |
| ○ No ○ Probably no ○ Probably yes ● Yes ○ Varies ○ Don't know |  |  |
| Feasibility Is the intervention feasible to implement? | | |
| Judgement | Research evidence | Additional considerations |
| ○ No ○ Probably no ● Probably yes ○ Yes ○ Varies ○ Don't know |  | Patients that live far away may not be appropriate for ERAS  Literacy, language, transportation, lack of home support, |

# Summary of judgements

|  | **Judgement** | | | | | | |
| --- | --- | --- | --- | --- | --- | --- | --- |
| **Problem** | No | Probably no | Probably yes | **Yes** |  | Varies | Don't know |
| **Desirable Effects** | Trivial | Small | Moderate | **Large** |  | Varies | Don't know |
| **Undesirable Effects** | Large | Moderate | **Small** | Trivial |  | Varies | Don't know |
| **Certainty of evidence** | Very low | Low | **Moderate** | High |  |  | No included studies |
| **Values** | Important uncertainty or variability | Possibly important uncertainty or variability | **Probably no important uncertainty or variability** | No important uncertainty or variability |  |  |  |
| **Balance of effects** | Favors the comparison | Probably favors the comparison | Does not favor either the intervention or the comparison | Probably favors the intervention | **Favors the intervention** | Varies | Don't know |
| **Acceptability** | No | Probably no | Probably yes | **Yes** |  | Varies | Don't know |
| **Feasibility** | No | Probably no | **Probably yes** | Yes |  | Varies | Don't know |

# Type of recommendation

| Strong recommendation against the intervention | Conditional recommendation against the intervention | Conditional recommendation for either the intervention or the comparison | Conditional recommendation for the intervention | **Strong recommendation for the intervention** |
| --- | --- | --- | --- | --- |
| ○ | ○ | ○ | ○ | **●** |

# Conclusions

| Recommendation |
| --- |
| There is no discussion that ERAS is an advantage, the problem is implementing it in 100% patients for reasons mentioned above. Need institutional help to implement to everyone. |
|  |
| Justification |
|  |
| Subgroup considerations |
|  |
| Implementation considerations |
| More support of ERAS programs for patients outside of the hospital, social support |
| Monitoring and evaluation |
|  |
| Research priorities |
| More standardized data collection and definitions to collect better evidence  Multi-institutional studies with collaborative groups  Develop registries for large, international studies |

EAES/SAGES Evidence-Based Guidelines on Optimization of Perioperative Care in Older Adults

# QUESTION #22

| **Should ERAS vs. Conventional Care be used for Gastric Surgery in Elderly Patients ?** | |
| --- | --- |
| **Population:** | Gastric Surgery in Elderly Patients |
| **Intervention:** | ERAS |
| **Comparison:** | Conventional Care |
| **Main outcomes:** | 30d Complications; Hospital Length of Stay (LOS); Readmissions; |
| **Setting:** |  |
| **Perspective:** |  |
| **Background:** |  |
| **Conflict of interests:** |  |

# Assessment

| Problem Is the problem a priority? | | |
| --- | --- | --- |
| Judgement | Research evidence | Additional considerations |
| ○ No ○ Probably no ○ Probably yes ● Yes ○ Varies ○ Don't know |  |  |
| Desirable Effects How substantial are the desirable anticipated effects? | | |
| Judgement | Research evidence | Additional considerations |
| ○ Trivial ● Small ○ Moderate ○ Large ○ Varies ○ Don't know | \| **Outcomes** \| **№ of participants (studies) Follow-up** \| **Certainty of the evidence (GRADE)** \| **Relative effect (95% CI)** \| **Anticipated absolute effects^*^ (95% CI)** \| \| \| --- \| --- \| --- \| --- \| --- \| --- \| \| **Risk with Conventional Care** \| **Risk difference with ERAS** \| \| 30d Complications \| 299 (2 RCTs) \| ⨁◯◯◯ Very low^a,b,c^ \| **OR 1.01** (0.17 to 5.97) \| Study population \| \| \| 527 per 1,000 \| **2 more per 1,000** (368 fewer to 342 more) \| \| Hospital Length of Stay (LOS) \| 299 (2 RCTs) \| ⨁⨁◯◯ Low^a,d^ \| - \| The mean hospital Length of Stay (LOS) was **0** days \| MD **0.83 days lower** (1.65 lower to 0.01 lower) \|  1. Although one trial was well done, the other included study did not explain the randomization process well and the groups were not compared enough to know if the randomization was well done 2. The two included studies had opposite findings, with one demonstrating less complications with ERAS and the other less with conventional care. This may be explained by the lack of definition of complications and unknown comparability between cohorts in the high risk of bias trial. 3. In addition to the small sample size and relatively small event size, there is a wide confidence interval with the estimated effects ranging from large benefit to large harm with ERAS. 4. The small sample size increases the fragility of this outcome. | Trivial Complications, but easily changed with more data |
| Undesirable Effects How substantial are the undesirable anticipated effects? | | |
| Judgement | Research evidence | Additional considerations |
| ○ Large ○ Moderate ● Small ○ Trivial ○ Varies ○ Don't know | \| **Outcomes** \| **№ of participants (studies) Follow-up** \| **Certainty of the evidence (GRADE)** \| **Relative effect (95% CI)** \| **Anticipated absolute effects^*^ (95% CI)** \| \| \| --- \| --- \| --- \| --- \| --- \| --- \| \| **Risk with Conventional Care** \| **Risk difference with ERAS** \| \| Readmissions \| 299 (2 RCTs) \| ⨁⨁◯◯ Low^a,b^ \| **OR 3.88** (1.22 to 12.35) \| Study population \| \| \| 27 per 1,000 \| **69 more per 1,000** (6 more to 226 more) \|  1. Although one trial was well done, the other included study did not explain the randomization process well and the groups were not compared enough to know if the randomization was well done 2. There was a small number of events and a confidence interval that crosses minimally important differences. |  |
| Certainty of evidence What is the overall certainty of the evidence of effects? | | |
| Judgement | Research evidence | Additional considerations |
| ○ Very low ● Low ○ Moderate ○ High ○ No included studies | \| **Outcomes** \| **Importance** \| **Certainty of the evidence (GRADE)** \| \| --- \| --- \| --- \| \| 30d Complications \| CRITICAL \| ⨁◯◯◯ Very low^a,b,c^ \| \| Hospital Length of Stay (LOS) \| CRITICAL \| ⨁⨁◯◯ Low^a,d^ \| \| Readmissions \| CRITICAL \| ⨁⨁◯◯ Low^a,e^ \|  1. Although one trial was well done, the other included study did not explain the randomization process well and the groups were not compared enough to know if the randomization was well done 2. The two included studies had opposite findings, with one demonstrating less complications with ERAS and the other less with conventional care. This may be explained by the lack of definition of complications and unknown comparability between cohorts in the high risk of bias trial. 3. In addition to the small sample size and relatively small event size, there is a wide confidence interval with the estimated effects ranging from large benefit to large harm with ERAS. 4. The small sample size increases the fragility of this outcome. 5. There was a small number of events and a confidence interval that crosses minimally important differences. |  |
| Values Is there important uncertainty about or variability in how much people value the main outcomes? | | |
| Judgement | Research evidence | Additional considerations |
| ○ Important uncertainty or variability ● Possibly important uncertainty or variability ○ Probably no important uncertainty or variability ○ No important uncertainty or variability |  | Long distance traveled patients – readmissions and LOS may be more important than Cxs as they are very impactful |
| Balance of effects Does the balance between desirable and undesirable effects favor the intervention or the comparison? | | |
| Judgement | Research evidence | Additional considerations |
| ○ Favors the comparison ○ Probably favors the comparison ● Does not favor either the intervention or the comparison ○ Probably favors the intervention ○ Favors the intervention ○ Varies ○ Don't know |  | Readmissions more important to patients than LOS which may |
| Acceptability Is the intervention acceptable to key stakeholders? | | |
| Judgement | Research evidence | Additional considerations |
| ○ No ○ Probably no ○ Probably yes ● Yes ○ Varies ○ Don't know |  |  |
| Feasibility Is the intervention feasible to implement? | | |
| Judgement | Research evidence | Additional considerations |
| ○ No ○ Probably no ● Probably yes ○ Yes ○ Varies ○ Don't know |  | Global considerations – local culture, MIS technology, no follow up due to long distances traveled Long distance for patients, social support  Need close follow up after discharge  Need institutional support to implement |

# Summary of judgements

|  | **Judgement** | | | | | | |
| --- | --- | --- | --- | --- | --- | --- | --- |
| **Problem** | No | Probably no | Probably yes | **Yes** |  | Varies | Don't know |
| **Desirable Effects** | Trivial | **Small** | Moderate | Large |  | Varies | Don't know |
| **Undesirable Effects** | Large | Moderate | **Small** | Trivial |  | Varies | Don't know |
| **Certainty of evidence** | Very low | **Low** | Moderate | High |  |  | No included studies |
| **Values** | Important uncertainty or variability | **Possibly important uncertainty or variability** | Probably no important uncertainty or variability | No important uncertainty or variability |  |  |  |
| **Balance of effects** | Favors the comparison | Probably favors the comparison | **Does not favor either the intervention or the comparison** | Probably favors the intervention | Favors the intervention | Varies | Don't know |
| **Acceptability** | No | Probably no | Probably yes | **Yes** |  | Varies | Don't know |
| **Feasibility** | No | Probably no | **Probably yes** | Yes |  | Varies | Don't know |

# Type of recommendation

| Strong recommendation against the intervention | Conditional recommendation against the intervention | **Conditional recommendation for either the intervention or the comparison** | Conditional recommendation for the intervention | Strong recommendation for the intervention |
| --- | --- | --- | --- | --- |
| ○ | ○ | **●** | ○ | ○ |

# Conclusions

| Recommendation |
| --- |
|  |
| Justification |
|  |
| Subgroup considerations |
|  |
| Implementation considerations |
|  |
| Monitoring and evaluation |
|  |
| Research priorities |
| Need ERAS implementation studies in resource limited environments  Multi-institutional RCT looking at ERAS in gastric surgery in elderly patients  At least prospective observational studies due to feasibility issues of doing RCT |

EAES/SAGES Evidence-Based Guidelines on Optimization of Perioperative Care in Older Adults

# QUESTION #23

| **Should ERAS vs. Conventional Care be used for HPB Surgery in Elderly Patients ?** | |
| --- | --- |
| **Population:** | HPB Surgery in Elderly Patients |
| **Intervention:** | ERAS |
| **Comparison:** | Conventional Care |
| **Main outcomes:** | 30day Complications; Length of Stay; Readmission; |
| **Setting:** |  |
| **Perspective:** |  |
| **Background:** |  |
| **Conflict of interests:** |  |

# Assessment

| Problem Is the problem a priority? | | |
| --- | --- | --- |
| Judgement | Research evidence | Additional considerations |
| ○ No ○ Probably no ○ Probably yes ● Yes ○ Varies ○ Don't know |  |  |
| Desirable Effects How substantial are the desirable anticipated effects? | | |
| Judgement | Research evidence | Additional considerations |
| ○ Trivial ● Small ○ Moderate ○ Large ○ Varies ○ Don't know | \| **Outcomes** \| **№ of participants (studies) Follow-up** \| **Certainty of the evidence (GRADE)** \| **Relative effect (95% CI)** \| **Anticipated absolute effects^*^ (95% CI)** \| \| \| --- \| --- \| --- \| --- \| --- \| --- \| \| **Risk with Conventional Care** \| **Risk difference with ERAS** \| \| 30day Complications \| 265 (2 observational studies) \| ⨁◯◯◯ Very low^a,b,c^ \| **OR 0.69** (0.11 to 4.37) \| Study population \| \| \| 405 per 1,000 \| **85 fewer per 1,000** (335 fewer to 343 more) \| \| Length of Stay \| 265 (2 observational studies) \| ⨁◯◯◯ Very low^a,d^ \| - \| The mean length of Stay was **0** days \| MD **2.03 days lower** (5.01 lower to 0.95 higher) \| \| Readmission \| 265 (2 observational studies) \| ⨁◯◯◯ Very low^a,c^ \| **OR 0.64** (0.20 to 2.06) \| Study population \| \| \| 87 per 1,000 \| **29 fewer per 1,000** (68 fewer to 77 more) \|  1. Both studies were judged to be of high risk of bias based on the Newcastle-Ottawa Scale. This was due to unclear description of how patients were selected for either intervention and lack of reporting on follow up. 2. The two included studies had opposite findings which introduces heterogeneity into the analysis (I2 84%). 3. The is considerable imprecision due to small sample sizes and large confidence intervals. The estimated effects range from large benefit to large harms. 4. Small sample sizes increases the fragility and thus imprecision of this outcome. |  |
| Undesirable Effects How substantial are the undesirable anticipated effects? | | |
| Judgement | Research evidence | Additional considerations |
| ○ Large ○ Moderate ○ Small ○ Trivial ○ Varies ● Don't know | There were no undesirable effects with ERAS for any critical outcomes. |  |

| Certainty of evidence What is the overall certainty of the evidence of effects? | | |
| --- | --- | --- |
| Judgement | Research evidence | Additional considerations |
| ● Very low ○ Low ○ Moderate ○ High ○ No included studies | \| **Outcomes** \| **Importance** \| **Certainty of the evidence (GRADE)** \| \| --- \| --- \| --- \| \| 30day Complications \| CRITICAL \| ⨁◯◯◯ Very low^a,b,c^ \| \| Length of Stay \| CRITICAL \| ⨁◯◯◯ Very low^a,d^ \| \| Readmission \| CRITICAL \| ⨁◯◯◯ Very low^a,c^ \|  1. Both studies were judged to be of high risk of bias based on the Newcastle-Ottawa Scale. This was due to unclear description of how patients were selected for either intervention and lack of reporting on follow up. 2. The two included studies had opposite findings which introduces heterogeneity into the analysis (I2 84%). 3. The is considerable imprecision due to small sample sizes and large confidence intervals. The estimated effects range from large benefit to large harms. 4. Small sample sizes increases the fragility and thus imprecision of this outcome. |  |
| Values Is there important uncertainty about or variability in how much people value the main outcomes? | | |
| Judgement | Research evidence | Additional considerations |
| ○ Important uncertainty or variability ● Possibly important uncertainty or variability ○ Probably no important uncertainty or variability ○ No important uncertainty or variability |  |  |
| Balance of effects Does the balance between desirable and undesirable effects favor the intervention or the comparison? | | |
| Judgement | Research evidence | Additional considerations |
| ○ Favors the comparison ○ Probably favors the comparison ○ Does not favor either the intervention or the comparison ● Probably favors the intervention ○ Favors the intervention ○ Varies ○ Don't know |  |  |
| Acceptability Is the intervention acceptable to key stakeholders? | | |
| Judgement | Research evidence | Additional considerations |
| ○ No ○ Probably no ○ Probably yes ● Yes ○ Varies ○ Don't know |  |  |
| Feasibility Is the intervention feasible to implement? | | |
| Judgement | Research evidence | Additional considerations |
| ○ No ○ Probably no ● Probably yes ○ Yes ○ Varies ○ Don't know |  |  |

# Summary of judgements

|  | **Judgement** | | | | | | |
| --- | --- | --- | --- | --- | --- | --- | --- |
| **Problem** | No | Probably no | Probably yes | **Yes** |  | Varies | Don't know |
| **Desirable Effects** | Trivial | **Small** | Moderate | Large |  | Varies | Don't know |
| **Undesirable Effects** | Large | Moderate | Small | Trivial |  | Varies | **Don't know** |
| **Certainty of evidence** | **Very low** | Low | Moderate | High |  |  | No included studies |
| **Values** | Important uncertainty or variability | **Possibly important uncertainty or variability** | Probably no important uncertainty or variability | No important uncertainty or variability |  |  |  |
| **Balance of effects** | Favors the comparison | Probably favors the comparison | Does not favor either the intervention or the comparison | **Probably favors the intervention** | Favors the intervention | Varies | Don't know |
| **Acceptability** | No | Probably no | Probably yes | **Yes** |  | Varies | Don't know |
| **Feasibility** | No | Probably no | **Probably yes** | Yes |  | Varies | Don't know |

# Type of recommendation

| Strong recommendation against the intervention | Conditional recommendation against the intervention | Conditional recommendation for either the intervention or the comparison | **Conditional recommendation for the intervention** | Strong recommendation for the intervention |
| --- | --- | --- | --- | --- |
| ○ | ○ | ○ | **●** | ○ |

# Conclusions

| Recommendation |
| --- |
|  |
| Justification |
|  |
| Subgroup considerations |
|  |
| Implementation considerations |
|  |
| Monitoring and evaluation |
|  |
| Research priorities |
|  |

EAES/SAGES Evidence-Based Guidelines on Optimization of Perioperative Care in Older Adults

# QUESTION #24

| **Should ERAS vs. Conventional Care be used for Foregut Surgery in Elderly Patients ?** | |
| --- | --- |
| **Population:** | Foregut Surgery in Elderly Patients |
| **Intervention:** | ERAS |
| **Comparison:** | Conventional Care |
| **Main outcomes:** |  |
| **Setting:** |  |
| **Perspective:** |  |
| **Background:** |  |
| **Conflict of interests:** |  |

# Assessment

| Problem Is the problem a priority? | | |
| --- | --- | --- |
| Judgement | Research evidence | Additional considerations |
| ○ No ○ Probably no ○ Probably yes ● Yes ○ Varies ○ Don't know |  |  |
| Desirable Effects How substantial are the desirable anticipated effects? | | |
| Judgement | Research evidence | Additional considerations |
| ○ Trivial ○ Small ○ Moderate ○ Large ○ Varies ● Don't know | No included studies. |  |

| Undesirable Effects How substantial are the undesirable anticipated effects? | | |
| --- | --- | --- |
| Judgement | Research evidence | Additional considerations |
| ○ Large ○ Moderate ○ Small ○ Trivial ○ Varies ● Don't know | No included studies. |  |
| Certainty of evidence What is the overall certainty of the evidence of effects? | | |
| Judgement | Research evidence | Additional considerations |
| ○ Very low ○ Low ○ Moderate ○ High ● No included studies | No included studies. |  |
| Values Is there important uncertainty about or variability in how much people value the main outcomes? | | |
| Judgement | Research evidence | Additional considerations |
| ○ Important uncertainty or variability ● Possibly important uncertainty or variability ○ Probably no important uncertainty or variability ○ No important uncertainty or variability |  |  |
| Balance of effects Does the balance between desirable and undesirable effects favor the intervention or the comparison? | | |
| Judgement | Research evidence | Additional considerations |
| ○ Favors the comparison ○ Probably favors the comparison ○ Does not favor either the intervention or the comparison ○ Probably favors the intervention ○ Favors the intervention ○ Varies ● Don't know |  |  |
| Acceptability Is the intervention acceptable to key stakeholders? | | |
| Judgement | Research evidence | Additional considerations |
| ○ No ○ Probably no ○ Probably yes ○ Yes ○ Varies ● Don't know |  |  |
| Feasibility Is the intervention feasible to implement? | | |
| Judgement | Research evidence | Additional considerations |
| ○ No ○ Probably no ○ Probably yes ○ Yes ○ Varies ● Don't know |  |  |

# Summary of judgements

|  | **Judgement** | | | | | | |
| --- | --- | --- | --- | --- | --- | --- | --- |
| **Problem** | No | Probably no | Probably yes | **Yes** |  | Varies | Don't know |
| **Desirable Effects** | Trivial | Small | Moderate | Large |  | Varies | **Don't know** |
| **Undesirable Effects** | Large | Moderate | Small | Trivial |  | Varies | **Don't know** |
| **Certainty of evidence** | Very low | Low | Moderate | High |  |  | **No included studies** |
| **Values** | Important uncertainty or variability | **Possibly important uncertainty or variability** | Probably no important uncertainty or variability | No important uncertainty or variability |  |  |  |
| **Balance of effects** | Favors the comparison | Probably favors the comparison | Does not favor either the intervention or the comparison | Probably favors the intervention | Favors the intervention | Varies | **Don't know** |
| **Acceptability** | No | Probably no | Probably yes | Yes |  | Varies | **Don't know** |
| **Feasibility** | No | Probably no | Probably yes | Yes |  | Varies | **Don't know** |

# Type of recommendation

| Strong recommendation against the intervention | Conditional recommendation against the intervention | **Conditional recommendation for either the intervention or the comparison** | Conditional recommendation for the intervention | Strong recommendation for the intervention |
| --- | --- | --- | --- | --- |
| ○ | ○ | **●** | ○ | ○ |

# Conclusions

| Recommendation |
| --- |
| Nissen, esophagectomy, PEH  May not have a lot to gain from pushing for more ERAS – Nissen already leaves early hard to improve, Esophagectomy limited data in elderly patients, let alone ERAS |
|  |
| Justification |
|  |

| Subgroup considerations |
| --- |
|  |
| Implementation considerations |
|  |

| Monitoring and evaluation |
| --- |
|  |
| Research priorities |
|  |
