# Supplementary material for: EAES/SAGES evidence-based recommendations and expert consensus on optimization of perioperative care in older adults
Source: Surg Endosc. 2024 Jun 28;38(8):4104–26. doi: 10.1007/s00464-024-10977-7 (PMC11289045; doi:10.1007/s00464-024-10977-7)
Supplement: Supplementary file 5 — Supplement 5 Forest plots for KQ17-KQ24 Supplementary file5 (PDF 349 KB) [file 464_2024_10977_MOESM5_ESM.pdf]

Q17 Complications

Study	LAP Events	LAP Total	OPEN Events	OPEN Total	RR	95%−CI	Weight (common)	Weight (random)
Delgado 2000	6	59	21	67	0.32	[0.14; 0.75]	0.1%	0.8%
Tuech 2000	4	22	12	24	0.36	[0.14; 0.96]	0.1%	0.6%
Law 2002	19	65	37	89	0.70	[0.45; 1.10]	0.2%	1.4%
Sklow 2003	16	39	16	39	1.00	[0.59; 1.70]	0.1%	1.2%
Matsuoka 2004	5	14	15	60	1.43	[0.62; 3.27]	0.0%	0.8%
Vignali 2005	13	61	19	61	0.68	[0.37; 1.26]	0.1%	1.1%
Feng 2006	9	51	38	102	0.47	[0.25; 0.90]	0.2%	1.0%
Person 2006	16	106	32	128	0.60	[0.35; 1.04]	0.2%	1.2%
Wang 2006	3	24	2	29	1.81	[0.33; 9.98]	0.0%	0.3%
Hester 2007	25	101	5	101	5.00	[1.99; 12.54]	0.0%	0.7%
Frasson 2008	18	89	42	112	0.54	[0.33; 0.87]	0.2%	1.3%
Akiyoshi 2009	6	44	11	43	0.53	[0.22; 1.31]	0.1%	0.7%
Allerdyce 2010	64	127	77	160	1.05	[0.83; 1.33]	0.4%	1.9%
Kurian 2010	34	150	40	95	0.54	[0.37; 0.79]	0.3%	1.6%
Lian 2010	62	97	71	97	0.87	[0.72; 1.06]	0.4%	2.0%
Tei 2010	14	48	57	81	0.41	[0.26; 0.66]	0.3%	1.4%
Tomimaru 2010	7	36	4	15	0.73	[0.25; 2.13]	0.0%	0.5%
Baek 2011	2	30	15	47	0.21	[0.05; 0.85]	0.1%	0.4%
Issa 2011	13	47	22	46	0.58	[0.33; 1.00]	0.1%	1.2%
Kennedy 2011	340	2113	965	3801	0.63	[0.57; 0.71]	4.3%	2.1%
Pinto 2011	30	83	68	116	0.62	[0.45; 0.85]	0.4%	1.7%
Wu 2011	19	88	6	62	2.23	[0.95; 5.26]	0.0%	0.7%
Altuntas 2012	11	56	5	34	1.34	[0.51; 3.51]	0.0%	0.6%
Clark III 2012	2	90	16	130	0.18	[0.04; 0.77]	0.1%	0.3%
Tan 2012	46	225	105	502	0.98	[0.72; 1.33]	0.4%	1.7%
Hatakeyama 2013	18	48	15	34	0.85	[0.50; 1.44]	0.1%	1.2%
Moazzez 2013	104	1030	138	1030	0.75	[0.59; 0.96]	0.9%	1.9%
Scarpa 2013	15	33	19	24	0.57	[0.37; 0.88]	0.1%	1.5%
She 2013	39	189	70	245	0.72	[0.51; 1.02]	0.4%	1.6%
Fujii 2014	23	100	36	100	0.64	[0.41; 1.00]	0.2%	1.4%
Miyasaka 2014	3	28	29	79	0.29	[0.10; 0.88]	0.1%	0.5%
Mukai 2014	5	44	10	37	0.42	[0.16; 1.12]	0.1%	0.6%
Nakamura 2014	6	34	14	46	0.58	[0.25; 1.35]	0.1%	0.7%
VallriberaValls 2014	43	134	57	133	0.75	[0.55; 1.03]	0.4%	1.7%
Chen 2015	29	32	37	55	1.35	[1.09; 1.67]	0.2%	1.9%
Guida 2015	1	17	6	24	0.24	[0.03; 1.78]	0.0%	0.2%
Hinoi 2015	127	459	168	459	0.76	[0.62; 0.91]	1.0%	2.0%
Jia 2015	3	20	9	20	0.33	[0.11; 1.05]	0.1%	0.5%
Kannan 2015	1676	11008	2622	11008	0.64	[0.60; 0.68]	16.2%	2.1%
Kohn 2015	24	36	20	36	1.20	[0.83; 1.74]	0.1%	1.6%
Nitsu 2015	26	103	119	295	0.63	[0.44; 0.90]	0.4%	1.6%
Tominaga 2015	3	12	14	29	0.52	[0.18; 1.48]	0.1%	0.6%
Weng 2015	22	112	60	182	0.60	[0.39; 0.91]	0.3%	1.5%
Cocorullo 2016	4	31	7	36	0.66	[0.21; 2.06]	0.0%	0.5%
Landi 2016	26	52	26	53	1.02	[0.69; 1.50]	0.2%	1.5%
Li 2016	263	874	343	904	0.79	[0.70; 0.90]	2.1%	2.1%
Moon 2016	23	71	32	71	0.72	[0.47; 1.10]	0.2%	1.5%
Nishikawa 2016	19	62	37	88	0.73	[0.47; 1.14]	0.2%	1.4%
Shigeta 2016	4	52	19	55	0.22	[0.08; 0.61]	0.1%	0.6%
Wang 2016	12	71	14	71	0.86	[0.43; 1.72]	0.1%	0.9%
Yap 2016	4	26	9	20	0.34	[0.12; 0.95]	0.1%	0.6%
Devoto 2017	31	135	54	157	0.67	[0.46; 0.97]	0.3%	1.6%
Rinaldi 2017	30	68	40	55	0.61	[0.44; 0.83]	0.3%	1.7%
Wu 2017	21	56	59	62	0.39	[0.28; 0.56]	0.3%	1.6%
Yamamoto 2017	43	153	66	153	0.65	[0.48; 0.89]	0.4%	1.7%
Chen 2018	12	16	17	21	0.93	[0.65; 1.32]	0.1%	1.6%
Chern 2018	54	336	137	797	0.93	[0.70; 1.25]	0.5%	1.8%
De Angelis 2018	18	43	27	43	0.67	[0.44; 1.02]	0.2%	1.5%
Duan 2018	14	214	29	72	0.16	[0.09; 0.29]	0.3%	1.1%
Miguchi 2018	11	52	21	52	0.52	[0.28; 0.97]	0.1%	1.1%
Troian 2018	73	112	51	76	0.97	[0.79; 1.20]	0.4%	1.9%
Wei 2018	11	24	7	26	1.70	[0.79; 3.67]	0.0%	0.8%
Chang 2019	28	157	352	699	0.35	[0.25; 0.50]	0.8%	1.6%
Daniel 2019	7	201	21	250	0.41	[0.18; 0.96]	0.1%	0.8%
Inoue 2019	5	33	39	89	0.35	[0.15; 0.80]	0.1%	0.8%
Key 2019	68	107	14	33	1.50	[0.98; 2.29]	0.1%	1.5%
Nishikawa 2019	8	47	16	43	0.46	[0.22; 0.96]	0.1%	0.9%
Richards 2019	165	861	1150	5879	0.98	[0.85; 1.13]	1.8%	2.0%
Zhou 2019	10	93	25	93	0.40	[0.20; 0.79]	0.2%	1.0%
Chern 2020	47	305	118	662	0.86	[0.63; 1.18]	0.5%	1.7%
Hashida 2020	0	82	2	26	0.06	[0.00; 1.30]	0.0%	0.1%
Sotirova 2020	9	39	5	39	1.80	[0.66; 4.89]	0.0%	0.6%
Stocchi 2020	6	42	14	42	0.43	[0.18; 1.01]	0.1%	0.7%
Ueda 2020	12	85	8	25	0.44	[0.20; 0.96]	0.1%	0.8%
van Harten 2020	57	116	60	116	0.95	[0.74; 1.23]	0.4%	1.8%
Chung 2021	3589	10450	13597	31017	0.78	[0.76; 0.81]	42.4%	2.1%
Cummings 2021	91	424	7104	27012	0.82	[0.68; 0.98]	1.4%	2.0%
Huang 2021	7	73	8	73	0.88	[0.33; 2.29]	0.0%	0.6%
Keller 2021	1738	4252	2993	5125	0.70	[0.67; 0.73]	16.8%	2.1%
Miguchi 2021	18	48	22	48	0.82	[0.51; 1.32]	0.1%	1.3%
White 2021	23	60	35	54	0.59	[0.41; 0.86]	0.2%	1.6%
**Common effect model**		**37227**		**94014**	**0.73**	**[0.72; 0.75]**	**100.0%**	**−−**
**Random effects model**					**0.70**	**[0.64; 0.77]**	**−−**	**100.0%**

Heterogeneity: $I^2 = 74\%$, $\tau^2 = 0.0998$, $p < 0.01$

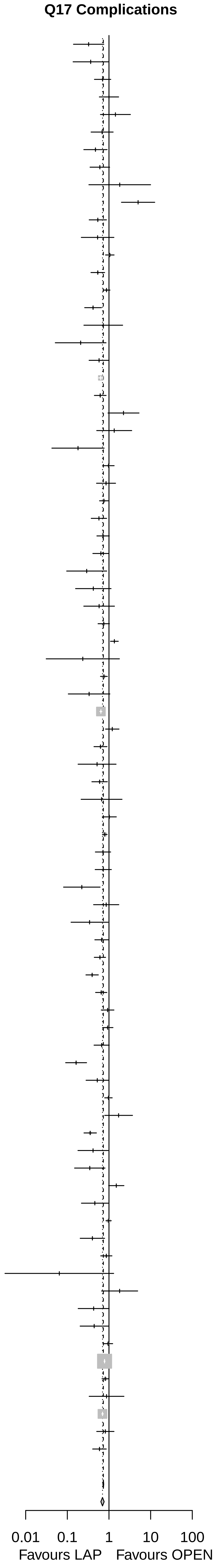

Q17 Complications - Subgroups

Study	LAP Events	LAP Total	OPEN Events	OPEN Total	RR	95%-CI	Weight (common)	Weight (random)
**group = colon**								
Delgado 2000	6	59	21	67	0.32	[0.14; 0.75]	0.2%	1.8%
Tuech 2000	4	22	12	24	0.36	[0.14; 0.96]	0.1%	1.5%
Sklow 2003	16	39	16	39	1.00	[0.59; 1.70]	0.2%	2.6%
Vignali 2005	13	61	19	61	0.68	[0.37; 1.26]	0.2%	2.3%
Person 2006	16	106	32	128	0.60	[0.35; 1.04]	0.3%	2.5%
Wang 2006	3	24	2	29	1.81	[0.33; 9.98]	0.0%	0.7%
Kurian 2010	34	150	40	95	0.54	[0.37; 0.79]	0.5%	3.0%
Lian 2010	62	97	71	97	0.87	[0.72; 1.06]	0.7%	3.5%
Tomimaru 2010	7	36	4	15	0.73	[0.25; 2.13]	0.1%	1.3%
Baek 2011	2	30	15	47	0.21	[0.05; 0.85]	0.1%	0.9%
Issa 2011	13	47	22	46	0.58	[0.33; 1.00]	0.2%	2.5%
Kennedy 2011	340	2113	965	3801	0.63	[0.57; 0.71]	6.6%	3.6%
Wu 2011	19	88	6	62	2.23	[0.95; 5.26]	0.1%	1.7%
Moazzez 2013	104	1030	138	1030	0.75	[0.59; 0.96]	1.3%	3.4%
She 2013	39	189	70	245	0.72	[0.51; 1.02]	0.6%	3.1%
Nakamura 2014	6	34	14	46	0.58	[0.25; 1.35]	0.1%	1.7%
VallriberaValls 2014	43	134	57	133	0.75	[0.55; 1.03]	0.5%	3.2%
Guida 2015	1	17	6	24	0.24	[0.03; 1.78]	0.0%	0.5%
Kannan 2015	1676	11008	2622	11008	0.64	[0.60; 0.68]	25.0%	3.7%
Tominaga 2015	3	12	14	29	0.52	[0.18; 1.48]	0.1%	1.4%
Hinoi 2015	145	402	100	402	1.45	[1.17; 1.80]	1.0%	3.5%
Cocorullo 2016	4	31	7	36	0.66	[0.21; 2.06]	0.1%	1.2%
Li 2016	263	874	343	904	0.79	[0.70; 0.90]	3.2%	3.6%
Wang 2016	12	71	14	71	0.86	[0.43; 1.72]	0.1%	2.1%
Rinaldi 2017	30	68	40	55	0.61	[0.44; 0.83]	0.4%	3.2%
Wu 2017	21	56	59	62	0.39	[0.28; 0.56]	0.5%	3.1%
Duan 2018	14	214	29	72	0.16	[0.09; 0.29]	0.4%	2.4%
Wei 2018	11	24	7	26	1.70	[0.79; 3.67]	0.1%	1.9%
Stocchi 2020	6	42	14	42	0.43	[0.18; 1.01]	0.1%	1.7%
Cummings 2021	91	424	7104	27012	0.82	[0.68; 0.98]	2.1%	3.5%
Chung 2021	13336	30519	3404	9932	1.27	[1.24; 1.31]	48.9%	3.7%
**Common effect model**		**48021**		**55640**	**0.99**	**[0.97; 1.01]**	**93.6%**	**--**
**Random effects model**					**0.69**	**[0.58; 0.82]**	**--**	**75.1%**
Heterogeneity: $I^2 = 96\%$, $\tau^2 = 0.1607$, $p < 0.01$								
**group = rectum**								
Akiyoshi 2009	6	44	11	43	0.53	[0.22; 1.31]	0.1%	1.6%
Clark III 2012	2	90	16	130	0.18	[0.04; 0.77]	0.1%	0.9%
Hinoi 2015	23	57	27	57	0.85	[0.56; 1.29]	0.3%	2.9%
Jia 2015	3	20	9	20	0.33	[0.11; 1.05]	0.1%	1.2%
Landi 2016	26	52	26	53	1.02	[0.69; 1.50]	0.2%	3.0%
Daniel 2019	7	201	21	250	0.41	[0.18; 0.96]	0.2%	1.8%
Richards 2019	165	861	1150	5879	0.98	[0.85; 1.13]	2.8%	3.6%
van Harten 2020	57	116	60	116	0.95	[0.74; 1.23]	0.6%	3.4%
Chung 2021	261	518	185	599	1.63	[1.41; 1.89]	1.6%	3.6%
White 2021	23	60	35	54	0.59	[0.41; 0.86]	0.4%	3.0%
**Common effect model**		**2019**		**7201**	**1.07**	**[0.98; 1.17]**	**6.4%**	**--**
**Random effects model**					**0.79**	**[0.58; 1.07]**	**--**	**24.9%**
Heterogeneity: $I^2 = 85\%$, $\tau^2 = 0.1659$, $p < 0.01$								
**Common effect model**		**50040**		**62841**	**0.99**	**[0.97; 1.02]**	**100.0%**	**--**
**Random effects model**					**0.71**	**[0.61; 0.83]**	**--**	**100.0%**

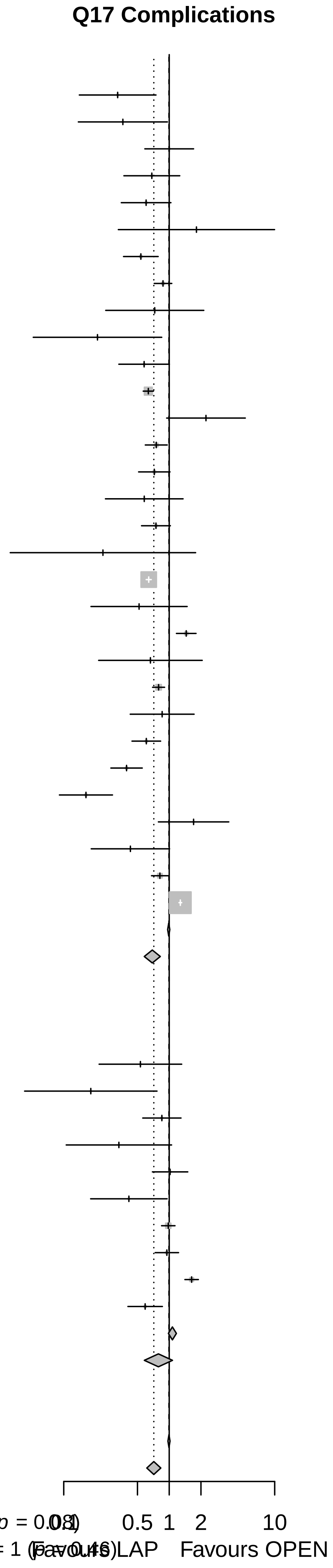

Heterogeneity: $I^2 = 95\%$, $\tau^2 = 0.1644$, $p < 0.01$
Test for subgroup differences (fixed effect): $\chi^2_1 = 2.99$, df = 1 ($p = 0.08$)
Test for subgroup differences (random effects): $\chi^2_1 = 0.55$, df = 1 ($p = 0.46$)

Q17 Length of Stay

Study	LAP Total	LAP Mean	LAP SD	OPEN Total	OPEN Mean	OPEN SD	MD	95%−CI	Weight (common)	Weight (random)
Delgado 2000	59	6.00	2.0000	67	7.00	3.0000	−1.00	[−1.88; −0.12]	0.7%	2.0%
Tuech 2000	22	13.10	5.2800	24	20.20	7.8100	−7.10	[−10.93; −3.27]	0.0%	0.8%
Law 2002	65	7.00	5.2500	89	9.00	6.8300	−2.00	[−3.91; −0.09]	0.1%	1.5%
Sklow 2003	39	3.90	0.2000	39	7.80	0.6000	−3.90	[−4.10; −3.70]	13.1%	2.2%
Matsuoka 2004	14	20.70	5.2800	46	24.70	7.8100	−4.00	[−7.57; −0.43]	0.0%	0.9%
Vignali 2005	61	9.80	5.3000	61	12.90	10.0000	−3.10	[−5.94; −0.26]	0.1%	1.1%
Feng 2006	51	21.20	11.8000	102	25.50	16.6000	−4.30	[−8.87; 0.27]	0.0%	0.6%
Person 2006	106	6.40	4.8000	128	8.70	3.9000	−2.30	[−3.44; −1.16]	0.4%	1.9%
Wang 2006	24	4.80	3.0000	29	5.00	3.1000	−0.20	[−1.85; 1.45]	0.2%	1.7%
Frasson 2008	89	9.50	3.8000	112	13.00	9.4000	−3.50	[−5.41; −1.59]	0.1%	1.5%
Akiyoshi 2009	44	19.00	29.0000	43	22.00	10.7500	−3.00	[−12.15; 6.15]	0.0%	0.2%
Allerdyce 2010	174	8.00	8.8300	152	10.00	9.3300	−2.00	[−3.98; −0.02]	0.1%	1.5%
Kurian 2010	95	7.11	7.7000	95	11.16	7.8200	−4.05	[−6.26; −1.84]	0.1%	1.4%
Lian 2010	97	6.00	11.0000	97	7.00	8.5000	−1.00	[−3.77; 1.77]	0.1%	1.1%
Tei 2010	86	9.00	9.5000	40	10.00	15.2500	−1.00	[−6.13; 4.13]	0.0%	0.5%
Tomimaru 2010	36	14.20	9.4000	15	18.00	8.3000	−3.80	[−9.00; 1.40]	0.0%	0.5%
Baek 2011	30	7.40	3.1000	47	9.00	5.8000	−1.60	[−3.60; 0.40]	0.1%	1.5%
Issa 2011	47	7.60	3.1000	46	8.80	3.6000	−1.20	[−2.57; 0.17]	0.3%	1.8%
Kennedy 2011	2113	6.70	5.2800	3801	8.70	7.8100	−2.00	[−2.34; −1.66]	4.6%	2.2%
Pinto 2011	83	6.00	8.5000	116	8.00	12.0000	−2.00	[−4.85; 0.85]	0.1%	1.1%
Wu 2011	88	8.30	8.5000	62	5.40	3.6000	2.90	[0.91; 4.89]	0.1%	1.5%
Altuntas 2012	56	8.50	7.5000	34	10.20	7.8000	−1.70	[−4.98; 1.58]	0.0%	1.0%
Clark III 2012	90	3.77	2.2000	130	6.23	4.7600	−2.46	[−3.40; −1.52]	0.6%	2.0%
Tan 2012	225	6.00	17.6700	502	7.00	18.8300	−1.00	[−3.84; 1.84]	0.1%	1.1%
Hatakeyama 2013	48	14.50	40.7500	34	21.00	12.7500	−6.50	[−18.80; 5.80]	0.0%	0.1%
Moazzez 2013	1030	3.60	4.2000	1030	4.70	5.1000	−1.10	[−1.50; −0.70]	3.2%	2.1%
She 2013	189	5.00	10.1700	245	7.00	17.6700	−2.00	[−4.65; 0.65]	0.1%	1.2%
Fujii 2014	100	11.70	9.2000	100	14.40	12.7000	−2.70	[−5.77; 0.37]	0.1%	1.0%
Miyasaka 2014	28	16.00	10.7500	79	28.00	18.0000	−12.00	[−17.62; −6.38]	0.0%	0.5%
Mukai 2014	44	14.70	10.2500	37	21.70	15.7500	−7.00	[−12.91; −1.09]	0.0%	0.4%
Nakamura 2014	74	10.00	6.5000	74	9.00	3.6700	1.00	[−0.70; 2.70]	0.2%	1.6%
VallriberaValls 2014	89	10.00	5.2800	88	14.30	7.8100	−4.30	[−6.27; −2.33]	0.1%	1.5%
Chen 2015	89	13.00	12.6700	89	16.00	31.3300	−3.00	[−10.02; 4.02]	0.0%	0.3%
Guida 2015	17	7.00	1.0000	24	8.50	2.0000	−1.50	[−2.43; −0.57]	0.6%	2.0%
Hinoi 2015	402	12.00	1.0000	402	13.00	1.5000	−1.00	[−1.18; −0.82]	16.6%	2.2%
Jia 2015	71	6.30	1.1000	71	9.90	1.7000	−3.60	[−4.07; −3.13]	2.3%	2.1%
Kannan 2015	11008	6.60	6.7000	11008	9.60	8.3000	−3.00	[−3.20; −2.80]	13.0%	2.2%
Kohn 2015	36	7.00	5.0000	36	8.00	19.2500	−1.00	[−7.50; 5.50]	0.0%	0.4%
Nitsu 2015	103	12.00	1.6700	295	15.00	1.8300	−3.00	[−3.38; −2.62]	3.5%	2.1%
Weng 2015	112	8.00	2.3300	182	11.00	9.8300	−3.00	[−4.49; −1.51]	0.2%	1.7%
Landi 2016	53	9.50	2.1300	53	9.00	2.0000	0.50	[−0.29; 1.29]	0.8%	2.0%
Moon 2016	71	9.00	0.6700	71	10.00	0.8300	−1.00	[−1.25; −0.75]	8.4%	2.2%
Nishikawa 2016	62	17.20	6.8000	88	22.00	14.0000	−4.80	[−8.18; −1.42]	0.0%	0.9%
Shigeta 2016	52	9.00	1.0000	55	13.00	3.2500	−4.00	[−4.90; −3.10]	0.6%	2.0%
Wang 2016	71	8.00	2.1700	71	10.00	2.6700	−2.00	[−2.80; −1.20]	0.8%	2.0%
Devoto 2017	135	13.10	5.2800	157	18.90	7.8100	−5.80	[−7.31; −4.29]	0.2%	1.7%
Rinaldi 2017	68	8.00	15.5000	55	12.00	30.2500	−4.00	[−12.80; 4.80]	0.0%	0.2%
Wu 2017	56	6.10	2.5000	59	9.60	3.5000	−3.50	[−4.61; −2.39]	0.4%	1.9%
Yamamoto 2017	153	12.00	0.8300	153	14.00	2.0000	−2.00	[−2.34; −1.66]	4.4%	2.2%
Chen 2018	27	24.80	33.0000	37	24.80	24.8000	0.00	[−14.79; 14.79]	0.0%	0.1%
Chern 2018	336	10.40	8.7000	797	13.80	13.5000	−3.40	[−4.72; −2.08]	0.3%	1.8%
De Angelis 2018	43	11.67	8.1710	43	14.78	9.3600	−3.11	[−6.82; 0.60]	0.0%	0.8%
Duan 2018	214	4.52	2.8900	72	6.17	3.6700	−1.65	[−2.58; −0.72]	0.6%	2.0%
Miguchi 2018	52	11.00	7.2500	52	14.00	14.0000	−3.00	[−7.29; 1.29]	0.0%	0.7%
Troian 2018	112	12.70	11.8000	113	10.90	5.5000	1.80	[−0.61; 4.21]	0.1%	1.3%
Wei 2018	24	16.00	14.2500	26	16.00	8.5000	0.00	[−6.57; 6.57]	0.0%	0.4%
Chang 2019	157	4.00	4.4000	699	8.00	7.0000	−4.00	[−4.86; −3.14]	0.7%	2.0%
Daniel 2019	201	4.60	3.3000	250	6.20	4.8000	−1.60	[−2.35; −0.85]	0.9%	2.0%
Inoue 2019	33	12.30	2.4000	89	18.40	1.5000	−6.10	[−6.98; −5.22]	0.7%	2.0%
Key 2019	107	9.39	4.6100	33	14.21	8.6000	−4.82	[−7.88; −1.76]	0.1%	1.0%
Nishikawa 2019	47	14.00	14.0000	43	19.00	25.0000	−5.00	[−13.48; 3.48]	0.0%	0.2%
Richards 2019	861	5.80	4.8000	5879	6.70	0.8300	−0.90	[−1.22; −0.58]	5.0%	2.2%
Zhou 2019	93	9.60	3.3000	93	12.20	5.5000	−2.60	[−3.90; −1.30]	0.3%	1.8%
Chern 2020	305	10.30	8.5000	662	13.50	9.5000	−3.20	[−4.40; −2.00]	0.4%	1.9%
Sotirova 2020	39	6.00	1.1300	39	8.00	1.2500	−2.00	[−2.53; −1.47]	1.8%	2.1%
Stocchi 2020	42	6.50	4.0000	42	10.20	4.4000	−3.70	[−5.50; −1.90]	0.2%	1.6%
Ueda 2020	85	23.00	39.0000	25	35.00	37.0000	−12.00	[−28.71; 4.71]	0.0%	0.1%
van Harten 2020	116	9.50	11.8300	116	12.00	11.5000	−2.50	[−5.50; 0.50]	0.1%	1.1%
Cummings 2021	424	8.30	6.2000	27012	10.60	7.6000	−2.30	[−2.90; −1.70]	1.5%	2.1%
Huang 2021	73	10.40	5.2000	73	14.70	8.9100	−4.30	[−6.67; −1.93]	0.1%	1.3%
Keller 2021	4252	5.70	4.5000	5125	8.20	6.5000	−2.50	[−2.72; −2.28]	10.3%	2.2%
White 2021	60	13.00	5.2800	54	17.00	7.8100	−4.00	[−6.47; −1.53]	0.1%	1.3%
**Common effect model**	**25658**			**61807**			**−2.23**	**[−2.30; −2.15]**	**100.0%**	**--**
**Random effects model**							**−2.48**	**[−2.90; −2.05]**	**--**	**100.0%**

Heterogeneity: $I^2 = 93\%$, $\tau^2 = 2.1762$, $p < 0.01$

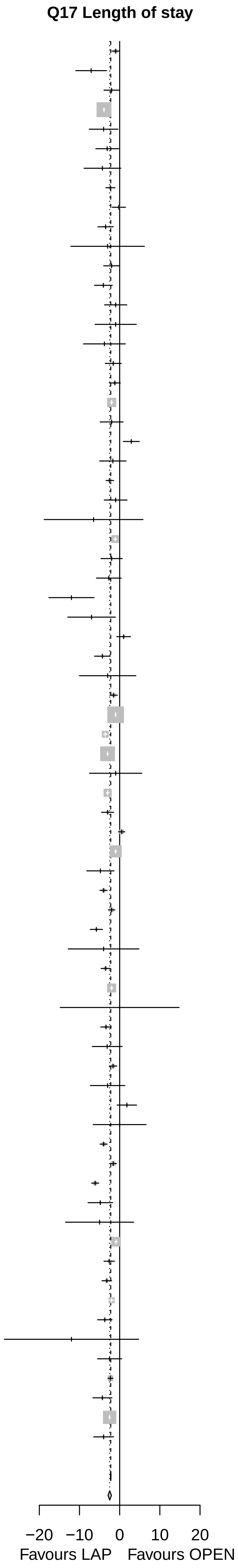

Q17 Length of Stay - Subgroups

Study	LAP Total	LAP Mean	LAP SD	OPEN Total	OPEN Mean	OPEN SD	MD	95%−CI	Weight (common)	Weight (random)
**group = colon**										
Delgado 2000	59	6.00	2.0000	67	7.00	3.0000	−1.00	[−1.88; −0.12]	1.2%	3.7%
Tuech 2000	22	13.10	4.5000	24	20.20	4.5800	−7.10	[−9.73; −4.47]	0.1%	2.2%
Sklow 2003	39	3.90	0.2000	39	7.80	0.6000	−3.90	[−4.10; −3.70]	23.9%	4.0%
Vignali 2005	61	9.80	5.3000	61	12.90	10.0000	−3.10	[−5.94; −0.26]	0.1%	2.1%
Person 2006	106	6.40	4.8000	128	8.70	3.9000	−2.30	[−3.44; −1.16]	0.7%	3.5%
Wang 2006	24	4.80	3.0000	29	5.00	3.1000	−0.20	[−1.85; 1.45]	0.3%	3.0%
Kurian 2010	95	7.11	7.7000	95	11.16	7.8200	−4.05	[−6.26; −1.84]	0.2%	2.6%
Lian 2010	97	6.00	11.0000	97	7.00	8.5000	−1.00	[−3.77; 1.77]	0.1%	2.1%
Tomimaru 2010	36	14.20	9.4000	15	18.00	8.3000	−3.80	[−9.00; 1.40]	0.0%	1.0%
Baek 2011	30	7.40	3.1000	47	9.00	5.8000	−1.60	[−3.60; 0.40]	0.2%	2.7%
Issa 2011	47	7.60	3.1000	46	8.80	3.6000	−1.20	[−2.57; 0.17]	0.5%	3.3%
Kennedy 2011	2113	6.70	4.5000	3801	8.70	4.5800	−2.00	[−2.24; −1.76]	16.2%	4.0%
Wu 2011	88	8.30	8.5000	62	5.40	3.6000	2.90	[0.91; 4.89]	0.2%	2.7%
Moazzez 2013	1030	3.60	4.2000	1030	4.70	5.1000	−1.10	[−1.50; −0.70]	5.8%	3.9%
She 2013	189	5.00	10.1700	245	7.00	17.6700	−2.00	[−4.65; 0.65]	0.1%	2.2%
Nakamura 2014	74	10.00	6.5000	74	9.00	3.6700	1.00	[−0.70; 2.70]	0.3%	3.0%
VallriberaValls 2014	89	10.00	4.5000	88	14.30	4.5800	−4.30	[−5.64; −2.96]	0.5%	3.3%
Guida 2015	17	7.00	1.0000	24	8.50	2.0000	−1.50	[−2.43; −0.57]	1.1%	3.6%
Kannan 2015	11008	6.60	6.7000	11008	9.60	8.3000	−3.00	[−3.20; −2.80]	23.7%	4.0%
Wang 2016	71	8.00	2.1700	71	10.00	2.6700	−2.00	[−2.80; −1.20]	1.5%	3.7%
Rinaldi 2017	68	8.00	15.5000	55	12.00	30.2500	−4.00	[−12.80; 4.80]	0.0%	0.4%
Wu 2017	56	6.10	2.5000	59	9.60	3.5000	−3.50	[−4.61; −2.39]	0.8%	3.5%
Duan 2018	214	4.52	2.8900	72	6.17	3.6700	−1.65	[−2.58; −0.72]	1.1%	3.6%
Wei 2018	24	16.00	14.2500	26	16.00	8.5000	0.00	[−6.57; 6.57]	0.0%	0.7%
Stocchi 2020	42	6.50	4.0000	42	10.20	4.4000	−3.70	[−5.50; −1.90]	0.3%	2.9%
Cummings 2021	424	8.30	6.2000	27012	10.60	7.6000	−2.30	[−2.90; −1.70]	2.6%	3.8%
**Common effect model**	**16123**			**44317**			**−2.78**	**[−2.88; −2.67]**	**81.9%**	**−−**
**Random effects model**							**−2.10**	**[−2.80; −1.40]**	**−−**	**75.7%**
Heterogeneity: $I^2 = 93\%$, $\tau^2 = 2.4346$, $p < 0.01$										
**group = rectum**										
Akiyoshi 2009	44	19.00	29.0000	43	22.00	10.7500	−3.00	[−12.15; 6.15]	0.0%	0.4%
Clark III 2012	90	3.77	2.2000	130	6.23	4.7600	−2.46	[−3.40; −1.52]	1.1%	3.6%
Jia 2015	71	6.30	1.1000	71	9.90	1.7000	−3.60	[−4.07; −3.13]	4.2%	3.9%
Landi 2016	53	9.50	2.1300	53	9.00	2.0000	0.50	[−0.29; 1.29]	1.5%	3.7%
Daniel 2019	201	4.60	3.3000	250	6.20	4.8000	−1.60	[−2.35; −0.85]	1.7%	3.8%
Richards 2019	861	5.80	4.8000	5879	6.70	0.8300	−0.90	[−1.22; −0.58]	9.1%	3.9%
van Harten 2020	116	9.50	11.8300	116	12.00	11.5000	−2.50	[−5.50; 0.50]	0.1%	2.0%
White 2021	60	13.00	4.5000	54	17.00	4.5800	−4.00	[−5.67; −2.33]	0.3%	3.0%
**Common effect model**	**1496**			**6596**			**−1.64**	**[−1.87; −1.41]**	**18.1%**	**−−**
**Random effects model**							**−2.01**	**[−3.20; −0.81]**	**−−**	**24.3%**
Heterogeneity: $I^2 = 94\%$, $\tau^2 = 2.2535$, $p < 0.01$										
**Common effect model**	**17619**			**50913**			**−2.57**	**[−2.67; −2.47]**	**100.0%**	**−−**
**Random effects model**							**−2.08**	**[−2.67; −1.49]**	**−−**	**100.0%**

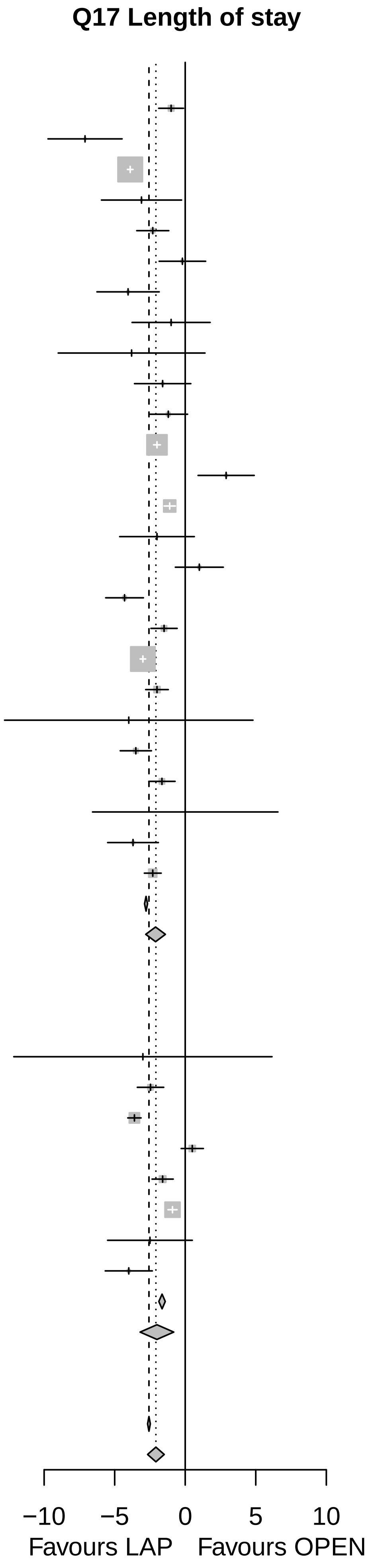

Heterogeneity: $I^2 = 94\%$, $\tau^2 = 2.2701$, $p < 0.01$
Test for subgroup differences (fixed effect): $\chi^2_1 = 77.77$, df = 1 ($p < 0.01$)
Test for subgroup differences (random effects): $\chi^2_1 = 0.02$, df = 1 ($p = 0.89$)

Q17 Readmissions

Study	LAP Events	LAP Total	OPEN Events	OPEN Total	RR	95%−CI	Weight (common)	Weight (random)
Vignali 2005	2	61	5	61	0.40	[0.08; 1.98]	0.7%	6.4%
Wu 2017	2	56	10	59	0.21	[0.05; 0.92]	1.3%	7.3%
Miguchi 2018	2	52	1	52	2.00	[0.19; 21.38]	0.1%	3.3%
Daniel 2019	3	201	2	250	1.87	[0.31; 11.06]	0.2%	5.4%
van Harten 2020	19	116	13	116	1.46	[0.76; 2.82]	1.8%	18.8%
Horsey 2021	264	3782	119	1892	1.11	[0.90; 1.37]	21.5%	28.9%
Keller 2021	303	4252	605	5125	0.60	[0.53; 0.69]	74.4%	30.0%
**Common effect model**		**8520**		**7555**	**0.73**	**[0.65; 0.81]**	**100.0%**	**--**
**Random effects model**					**0.85**	**[0.54; 1.34]**	**--**	**100.0%**

Heterogeneity: $I^2 = 82\%$, $\tau^2 = 0.1760$, $p < 0.01$

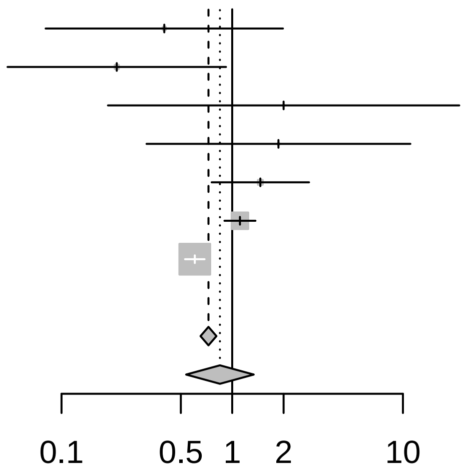

Q18 Complications

Study	LAP Events	LAP Total	OPEN Events	OPEN Total	RR	95%−CI	Weight (common)	Weight (random)
Mochiki 2005	4	30	4	16	0.53	[0.15; 1.85]	0.6%	1.9%
Li 2014	8	54	16	54	0.50	[0.23; 1.07]	1.9%	4.2%
Molena 2014	363	4764	247	1191	0.37	[0.32; 0.43]	45.9%	12.0%
Qiu 2014	7	30	16	34	0.50	[0.24; 1.04]	1.7%	4.3%
Lu 2015	57	252	78	252	0.73	[0.54; 0.98]	9.1%	9.9%
Wu 2016	13	64	16	64	0.81	[0.43; 1.55]	1.9%	5.1%
Zheng 2016	5	23	7	27	0.84	[0.31; 2.29]	0.7%	2.8%
Inokuchi 2017	13	45	12	25	0.60	[0.33; 1.11]	1.8%	5.4%
Konishi 2017	2	12	38	122	0.54	[0.15; 1.95]	0.8%	1.8%
Liu 2017	6	27	6	53	1.96	[0.70; 5.51]	0.5%	2.6%
Pan 2017	149	1180	167	1180	0.89	[0.73; 1.10]	19.4%	11.3%
Kim 2018	13	59	55	183	0.73	[0.43; 1.24]	3.1%	6.4%
Tsuchiya 2018	9	39	7	39	1.29	[0.53; 3.11]	0.8%	3.4%
Abbassi−Ghadi 2019	5	35	10	44	0.63	[0.24; 1.67]	1.0%	2.9%
Costa 2020	7	25	38	81	0.60	[0.31; 1.17]	2.1%	4.9%
Ushimaru 2020	11	56	14	46	0.65	[0.32; 1.28]	1.8%	4.8%
Chen 2021	28	129	37	129	0.76	[0.49; 1.16]	4.3%	7.8%
Fransvea 2021	18	33	24	34	0.77	[0.53; 1.13]	2.7%	8.5%
**Common effect model**		**6857**		**3574**	**0.59**	**[0.54; 0.65]**	**100.0%**	**−−**
**Random effects model**					**0.68**	**[0.56; 0.82]**	**−−**	**100.0%**

Heterogeneity: $I^2 = 76\%$, $\tau^2 = 0.0706$, $p < 0.01$

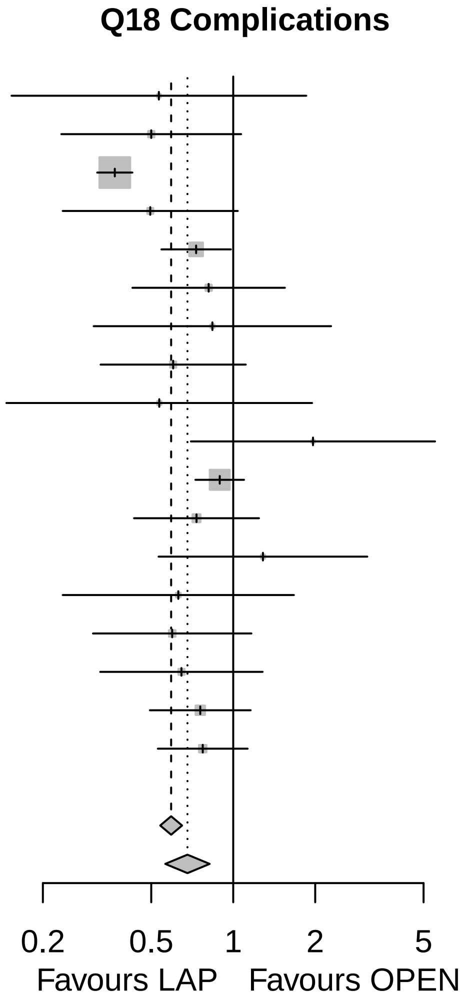

Q18 Complications - Subgroups

Study	LAP Events	LAP Total	OPEN Events	OPEN Total	RR	95%−CI	Weight (common)	Weight (random)
**group = gastric**								
Mochiki 2005	4	30	4	16	0.53	[0.15; 1.85]	0.6%	1.9%
Li 2014	8	54	16	54	0.50	[0.23; 1.07]	1.9%	4.2%
Qiu 2014	7	30	16	34	0.50	[0.24; 1.04]	1.7%	4.3%
Lu 2015	57	252	78	252	0.73	[0.54; 0.98]	9.1%	9.9%
Wu 2016	13	64	16	64	0.81	[0.43; 1.55]	1.9%	5.1%
Zheng 2016	5	23	7	27	0.84	[0.31; 2.29]	0.7%	2.8%
Inokuchi 2017	13	45	12	25	0.60	[0.33; 1.11]	1.8%	5.4%
Konishi 2017	2	12	38	122	0.54	[0.15; 1.95]	0.8%	1.8%
Liu 2017	6	27	6	53	1.96	[0.70; 5.51]	0.5%	2.6%
Pan 2017	149	1180	167	1180	0.89	[0.73; 1.10]	19.4%	11.3%
Kim 2018	13	59	55	183	0.73	[0.43; 1.24]	3.1%	6.4%
Tsuchiya 2018	9	39	7	39	1.29	[0.53; 3.11]	0.8%	3.4%
Abbassi−Ghadi 2019	5	35	10	44	0.63	[0.24; 1.67]	1.0%	2.9%
Costa 2020	7	25	38	81	0.60	[0.31; 1.17]	2.1%	4.9%
Ushimaru 2020	11	56	14	46	0.65	[0.32; 1.28]	1.8%	4.8%
Chen 2021	28	129	37	129	0.76	[0.49; 1.16]	4.3%	7.8%
Fransvea 2021	18	33	24	34	0.77	[0.53; 1.13]	2.7%	8.5%
**Common effect model**		**2093**		**2383**	**0.78**	**[0.69; 0.88]**	**54.1%**	**−−**
**Random effects model**					**0.78**	**[0.69; 0.88]**	**−−**	**88.0%**
Heterogeneity: $I^2 = 0\%$, $\tau^2 = 0$, $p = 0.78$								
**group = gastr/esoph**								
Molena 2014	363	4764	247	1191	0.37	[0.32; 0.43]	45.9%	12.0%
**Common effect model**		**4764**		**1191**	**0.37**	**[0.32; 0.43]**	**45.9%**	**−−**
**Random effects model**					**0.37**	**[0.32; 0.43]**	**−−**	**12.0%**
Heterogeneity: not applicable								
**Common effect model**		**6857**		**3574**	**0.59**	**[0.54; 0.65]**	**100.0%**	**−−**
**Random effects model**					**0.68**	**[0.56; 0.82]**	**−−**	**100.0%**

Heterogeneity: $I^2 = 76\%$, $\tau^2 = 0.0706$, $p < 0.01$
Test for subgroup differences (fixed effect): $\chi^2_1 = 59.51$, df = 1 ($p < 0.01$)
Test for subgroup differences (random effects): $\chi^2_1 = 59.00$, df = 1 ($p < 0.01$)

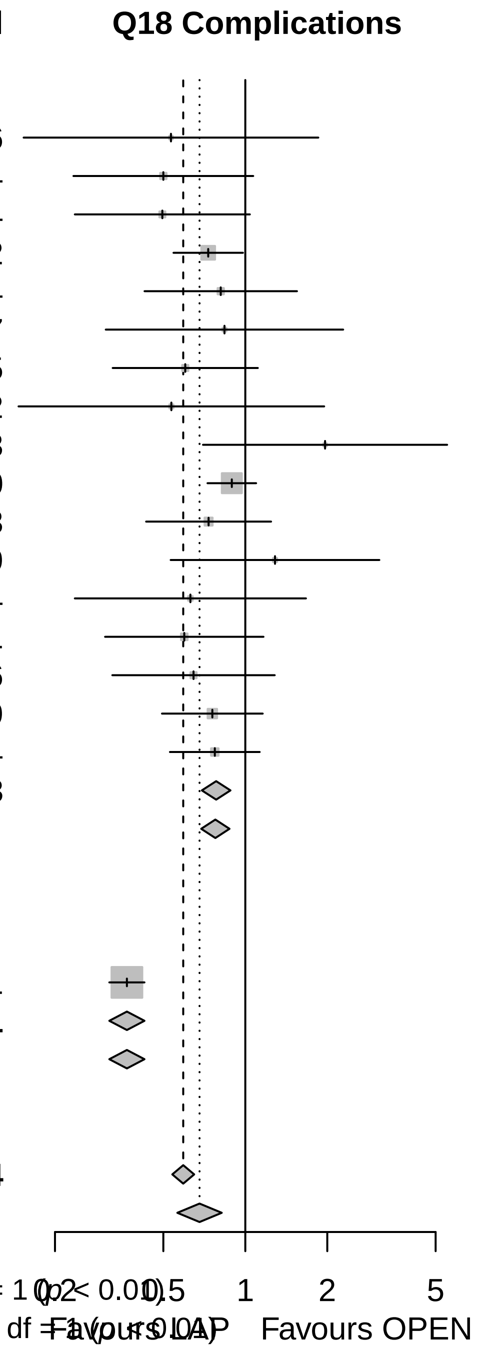

Q18 Length of stay

Study	LAP Total	LAP Mean	LAP SD	OPEN Total	OPEN Mean	OPEN SD	MD	95%−CI	Weight (common)	Weight (random)
Mochiki 2005	30	19.20	1.9000	16	28.40	3.5000	−9.20	[−11.04; −7.36]	1.4%	8.2%
Li 2014	54	7.80	1.3000	54	9.40	1.5000	−1.60	[−2.13; −1.07]	16.4%	9.4%
Qiu 2014	30	13.00	4.2000	34	16.90	4.1000	−3.90	[−5.94; −1.86]	1.1%	7.9%
Lu 2015	252	14.40	6.3000	252	16.60	8.9000	−2.20	[−3.55; −0.85]	2.5%	8.8%
Wu 2016	64	10.00	2.7500	64	12.00	4.0000	−2.00	[−3.19; −0.81]	3.3%	8.9%
Zheng 2016	23	12.50	2.5000	27	16.00	3.0000	−3.50	[−5.02; −1.98]	2.0%	8.6%
Inokuchi 2017	45	8.00	59.0000	25	14.50	7.0000	−6.50	[−23.96; 10.96]	0.0%	0.6%
Liu 2017	27	10.70	8.6000	53	15.40	9.7000	−4.70	[−8.86; −0.54]	0.3%	5.2%
Pan 2017	1180	2.00	4.2000	1180	6.00	4.1000	−4.00	[−4.33; −3.67]	41.0%	9.5%
Kim 2018	59	7.80	1.8000	183	8.40	0.9000	−0.60	[−1.08; −0.12]	20.2%	9.4%
Tsuchiya 2018	39	13.00	26.0000	39	15.40	11.2500	−2.40	[−11.29; 6.49]	0.1%	2.0%
Abbassi−Ghadi 2019	35	8.00	1.7500	44	8.00	1.2500	0.00	[−0.69; 0.69]	9.7%	9.3%
Chen 2021	129	13.00	5.5000	129	14.00	7.0000	−1.00	[−2.54; 0.54]	1.9%	8.6%
Fransvea 2021	33	11.00	11.0000	34	14.00	13.0000	−3.00	[−8.76; 2.76]	0.1%	3.6%
**Common effect model**	**2000**			**2134**			**−2.42**	**[−2.64; −2.21]**	**100.0%**	**−−**
**Random effects model**							**−2.84**	**[−4.24; −1.45]**	**−−**	**100.0%**

Heterogeneity: $I^2 = 95\%$, $\tau^2 = 5.3178$, $p < 0.01$

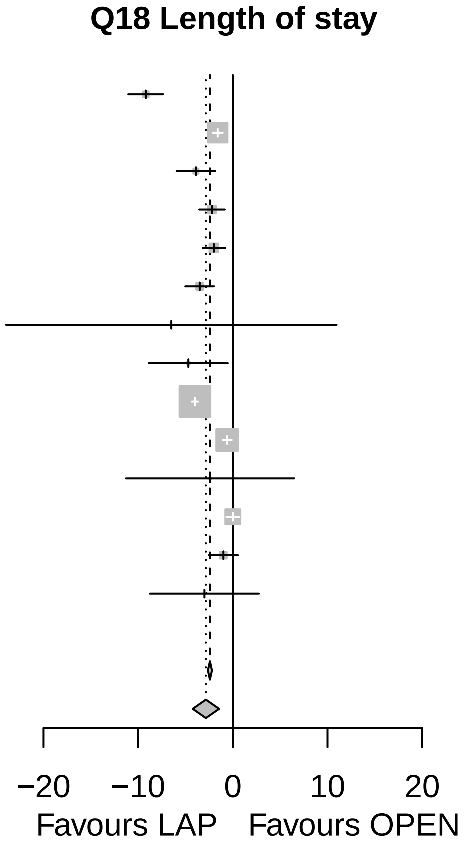

Q18 Length of stay - Subgroups

Study	LAP Total	LAP Mean	LAP SD	OPEN Total	OPEN Mean	OPEN SD	MD	95%−CI	Weight (common)	Weight (random)
**group = gastric**										
Mochiki 2005	30	19.20	1.9000	16	28.40	3.5000	−9.20	[−11.04; −7.36]	1.4%	8.2%
Li 2014	54	7.80	1.3000	54	9.40	1.5000	−1.60	[−2.13; −1.07]	16.4%	9.4%
Qiu 2014	30	13.00	4.2000	34	16.90	4.1000	−3.90	[−5.94; −1.86]	1.1%	7.9%
Lu 2015	252	14.40	6.3000	252	16.60	8.9000	−2.20	[−3.55; −0.85]	2.5%	8.8%
Wu 2016	64	10.00	2.7500	64	12.00	4.0000	−2.00	[−3.19; −0.81]	3.3%	8.9%
Zheng 2016	23	12.50	2.5000	27	16.00	3.0000	−3.50	[−5.02; −1.98]	2.0%	8.6%
Inokuchi 2017	45	8.00	59.0000	25	14.50	7.0000	−6.50	[−23.96; 10.96]	0.0%	0.6%
Liu 2017	27	10.70	8.6000	53	15.40	9.7000	−4.70	[−8.86; −0.54]	0.3%	5.2%
Pan 2017	1180	2.00	4.2000	1180	6.00	4.1000	−4.00	[−4.33; −3.67]	41.0%	9.5%
Kim 2018	59	7.80	1.8000	183	8.40	0.9000	−0.60	[−1.08; −0.12]	20.2%	9.4%
Tsuchiya 2018	39	13.00	26.0000	39	15.40	11.2500	−2.40	[−11.29; 6.49]	0.1%	2.0%
Abbassi−Ghadi 2019	35	8.00	1.7500	44	8.00	1.2500	0.00	[−0.69; 0.69]	9.7%	9.3%
Chen 2021	129	13.00	5.5000	129	14.00	7.0000	−1.00	[−2.54; 0.54]	1.9%	8.6%
Fransvea 2021	33	11.00	11.0000	34	14.00	13.0000	−3.00	[−8.76; 2.76]	0.1%	3.6%
**Common effect model**	**2000**			**2134**			**−2.42**	**[−2.64; −2.21]**	**100.0%**	**−−**
**Random effects model**							**−2.84**	**[−4.24; −1.45]**	**−−**	**100.0%**
Heterogeneity: $I^2 = 95\%$, $\tau^2 = 5.3178$, $p < 0.01$										
**Common effect model**	**2000**			**2134**			**−2.42**	**[−2.64; −2.21]**	**100.0%**	**−−**
**Random effects model**							**−2.84**	**[−4.24; −1.45]**	**−−**	**100.0%**

Heterogeneity: $I^2 = 95\%$, $\tau^2 = 5.3178$, $p < 0.01$

Test for subgroup differences (fixed effect): $\chi^2_0 = 0.00$, df = 0 (p = NA)

Test for subgroup differences (random effects): $\chi^2_0 = 0.00$, df = 0 (p = NA)

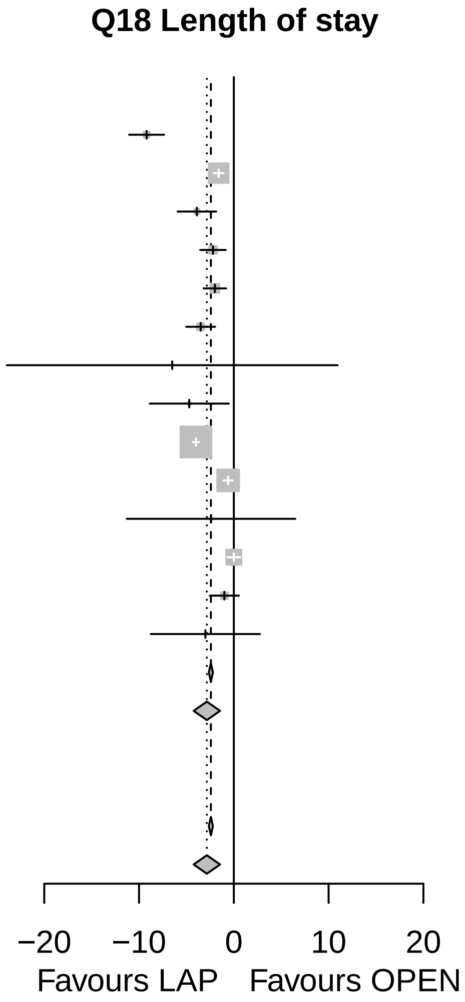

Q18 Readmission

Study	LAP Events	LAP Total	OPEN Events	OPEN Total	RR	95%−CI	Weight (common)	Weight (random)
Inokuchi 2017	1	45	0	25	1.68	[0.07; 39.78]	5.1%	10.9%
Chen 2021	4	129	12	129	0.33	[0.11; 1.01]	94.9%	89.1%
**Common effect model**		**174**		**154**	**0.40**	**[0.15; 1.10]**	**100.0%**	**−−**
**Random effects model**					**0.40**	**[0.14; 1.13]**	**−−**	**100.0%**

Heterogeneity: $I^2 = 0\%$, $\tau^2 = 0$, $p = 0.34$

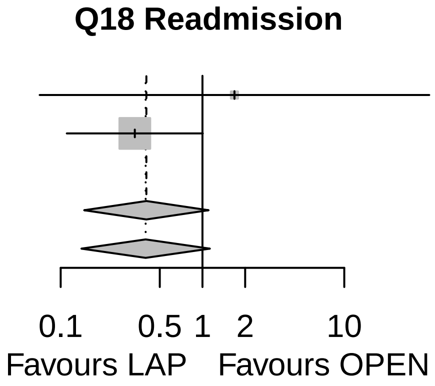

Q18 Readmission - Subgroups

Study	LAP Events	LAP Total	OPEN Events	OPEN Total	RR	95%−CI	Weight (common)	Weight (random)
**group = gastric**								
Inokuchi 2017	1	45	0	25	1.68	[0.07; 39.78]	5.1%	10.9%
Chen 2021	4	129	12	129	0.33	[0.11; 1.01]	94.9%	89.1%
**Common effect model**		**174**		**154**	**0.40**	**[0.15; 1.10]**	**100.0%**	**−−**
**Random effects model**					**0.40**	**[0.14; 1.13]**	**−−**	**100.0%**
Heterogeneity: $I^2 = 0\%$, $\tau^2 = 0$, $p = 0.34$								
**Common effect model**		**174**		**154**	**0.40**	**[0.15; 1.10]**	**100.0%**	**−−**
**Random effects model**					**0.40**	**[0.14; 1.13]**	**−−**	**100.0%**

Heterogeneity: $I^2 = 0\%$, $\tau^2 = 0$, $p = 0.34$

Test for subgroup differences (fixed effect): $\chi^2_0 = 0.00$, df = 0 (p = NA)

Test for subgroup differences (random effects): $\chi^2_0 = 0.00$, df = 0 (p = NA)

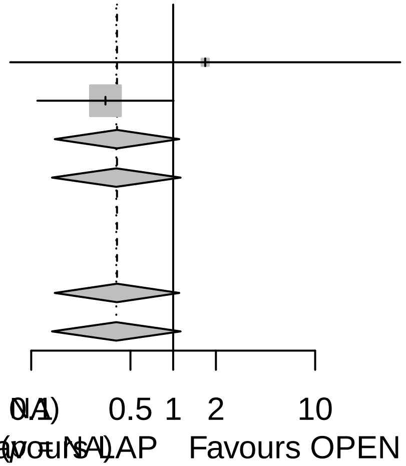

Q19 Complications

Study	LAP Events	LAP Total	OPEN Events	OPEN Total	RR	95%−CI	Weight (common)	Weight (random)
Pessaux 2001	9	50	19	89	0.84	[0.41; 1.72]	1.7%	2.9%
Chau 2002	4	31	17	42	0.32	[0.12; 0.85]	1.8%	2.0%
Fisichella 2002	3	24	6	11	0.23	[0.07; 0.75]	1.0%	1.6%
Moyson 2008	19	73	14	15	0.28	[0.19; 0.42]	2.9%	4.2%
Leardi 2009	12	258	14	83	0.28	[0.13; 0.57]	2.6%	2.8%
Vanounou 2010	5	19	11	15	0.36	[0.16; 0.81]	1.5%	2.5%
Gurgenidze 2013	36	134	22	159	1.94	[1.20; 3.13]	2.5%	3.9%
Chan 2014	4	17	6	34	1.33	[0.43; 4.10]	0.5%	1.7%
Tanaka 2015	0	20	9	20	0.05	[0.00; 0.85]	1.2%	0.4%
Wang 2015	3	30	10	60	0.60	[0.18; 2.02]	0.8%	1.5%
Cauchi 2016	13	26	20	26	0.65	[0.42; 1.01]	2.5%	4.0%
Cocorullo 2016	0	17	3	19	0.16	[0.01; 2.87]	0.4%	0.3%
Harada 2016	6	20	23	48	0.63	[0.30; 1.30]	1.7%	2.8%
Zeng 2016	14	79	19	79	0.74	[0.40; 1.36]	2.4%	3.2%
Amato 2017	0	11	6	18	0.12	[0.01; 2.00]	0.6%	0.4%
Aprea 2017	2	7	5	15	0.86	[0.22; 3.38]	0.4%	1.3%
Irojah 2017	103	791	69	216	0.41	[0.31; 0.53]	13.4%	4.8%
Lin 2017	1	21	26	97	0.18	[0.03; 1.24]	1.1%	0.7%
Martinez 2017	49	225	87	225	0.56	[0.42; 0.76]	10.8%	4.7%
Goh 2018	8	32	11	32	0.73	[0.34; 1.57]	1.4%	2.7%
Le Roux 2018	17	38	17	24	0.63	[0.41; 0.98]	2.6%	4.0%
Souche 2018	8	53	7	56	1.21	[0.47; 3.10]	0.8%	2.1%
Badawy 2019	6	40	15	40	0.40	[0.17; 0.93]	1.9%	2.4%
Chen 2019	7	70	7	48	0.69	[0.26; 1.83]	1.0%	2.0%
Shin 2019	3	56	6	270	2.41	[0.62; 9.35]	0.3%	1.3%
Untereiner 2019	13	33	22	33	0.59	[0.36; 0.96]	2.7%	3.8%
Chen 2020	10	64	11	64	0.91	[0.42; 1.99]	1.4%	2.6%
Costa 2020	80	358	40	94	0.53	[0.39; 0.71]	7.8%	4.6%
Delvecchio 2020	7	38	39	84	0.40	[0.20; 0.80]	3.0%	2.9%
Dumronggittigule 2020	32	127	20	49	0.62	[0.39; 0.97]	3.6%	4.0%
Ke 2020	37	75	84	225	1.32	[0.99; 1.76]	5.2%	4.7%
Kim 2020	10	96	27	160	0.62	[0.31; 1.22]	2.5%	3.0%
Liang 2020	11	27	8	19	0.97	[0.48; 1.94]	1.2%	2.9%
Nomi 2020	27	155	57	155	0.47	[0.32; 0.71]	7.1%	4.2%
Tee 2020	11	113	34	225	0.64	[0.34; 1.22]	2.8%	3.1%
Yin 2021	11	26	114	119	0.44	[0.28; 0.69]	5.1%	4.0%
**Common effect model**		**3254**		**2968**	**0.60**	**[0.55; 0.66]**	**100.0%**	**−−**
**Random effects model**					**0.60**	**[0.50; 0.71]**	**−−**	**100.0%**

Heterogeneity: $I^2 = 68\%$, $\tau^2 = 0.1507$, $p < 0.01$

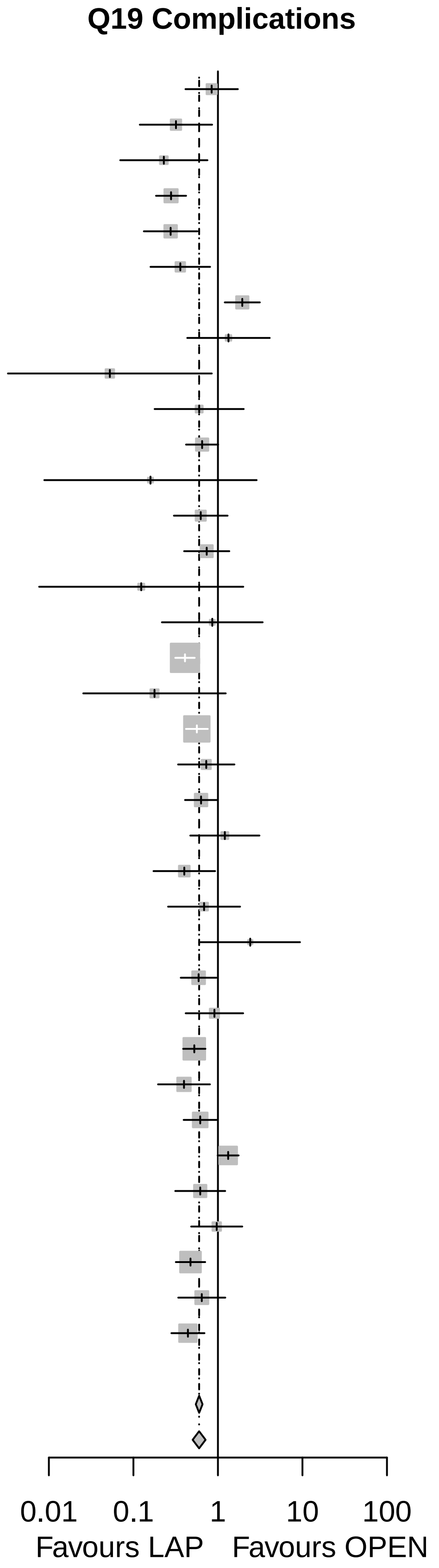

Q19 Complications - Subgroups

Study	LAP Events	LAP Total	OPEN Events	OPEN Total	RR	95%−CI	Weight (common)	Weight (random)
**group = cholecyst**								
Pessaux 2001	9	50	19	89	0.84	[0.41; 1.72]	1.7%	2.9%
Chau 2002	4	31	17	42	0.32	[0.12; 0.85]	1.8%	2.0%
Fisichella 2002	3	24	6	11	0.23	[0.07; 0.75]	1.0%	1.6%
Moyson 2008	19	73	14	15	0.28	[0.19; 0.42]	2.9%	4.2%
Leardi 2009	12	258	14	83	0.28	[0.13; 0.57]	2.6%	2.8%
Gurgenidze 2013	36	134	22	159	1.94	[1.20; 3.13]	2.5%	3.9%
Cocorullo 2016	0	17	3	19	0.16	[0.01; 2.87]	0.4%	0.3%
Irojah 2017	103	791	69	216	0.41	[0.31; 0.53]	13.4%	4.8%
Lin 2017	1	21	26	97	0.18	[0.03; 1.24]	1.1%	0.7%
Costa 2020	80	358	40	94	0.53	[0.39; 0.71]	7.8%	4.6%
**Common effect model**		**1757**		**825**	**0.52**	**[0.45; 0.61]**	**35.3%**	**−−**
**Random effects model**					**0.45**	**[0.28; 0.73]**	**−−**	**27.7%**
Heterogeneity: $I^2 = 82\%$, $\tau^2 = 0.4142$, $p < 0.01$								
**group = liver**								
Vanounou 2010	5	19	11	15	0.36	[0.16; 0.81]	1.5%	2.5%
Chan 2014	4	17	6	34	1.33	[0.43; 4.10]	0.5%	1.7%
Tanaka 2015	0	20	9	20	0.05	[0.00; 0.85]	1.2%	0.4%
Wang 2015	3	30	10	60	0.60	[0.18; 2.02]	0.8%	1.5%
Cauchi 2016	13	26	20	26	0.65	[0.42; 1.01]	2.5%	4.0%
Harada 2016	6	20	23	48	0.63	[0.30; 1.30]	1.7%	2.8%
Zeng 2016	14	79	19	79	0.74	[0.40; 1.36]	2.4%	3.2%
Amato 2017	0	11	6	18	0.12	[0.01; 2.00]	0.6%	0.4%
Martinez 2017	49	225	87	225	0.56	[0.42; 0.76]	10.8%	4.7%
Goh 2018	8	32	11	32	0.73	[0.34; 1.57]	1.4%	2.7%
Le Roux 2018	17	38	17	24	0.63	[0.41; 0.98]	2.6%	4.0%
Badawy 2019	6	40	15	40	0.40	[0.17; 0.93]	1.9%	2.4%
Untereiner 2019	13	33	22	33	0.59	[0.36; 0.96]	2.7%	3.8%
Chen 2020	10	64	11	64	0.91	[0.42; 1.99]	1.4%	2.6%
Delvecchio 2020	7	38	39	84	0.40	[0.20; 0.80]	3.0%	2.9%
Dumronggittigule 2020	32	127	20	49	0.62	[0.39; 0.97]	3.6%	4.0%
Kim 2020	10	96	27	160	0.62	[0.31; 1.22]	2.5%	3.0%
Nomi 2020	27	155	57	155	0.47	[0.32; 0.71]	7.1%	4.2%
Tee 2020	11	113	34	225	0.64	[0.34; 1.22]	2.8%	3.1%
**Common effect model**		**1183**		**1391**	**0.56**	**[0.49; 0.65]**	**50.8%**	**−−**
**Random effects model**					**0.58**	**[0.51; 0.67]**	**−−**	**54.0%**
Heterogeneity: $I^2 = 0\%$, $\tau^2 = < 0.0001$, $p = 0.78$								
**group = pancreas**								
Aprea 2017	2	7	5	15	0.86	[0.22; 3.38]	0.4%	1.3%
Souche 2018	8	53	7	56	1.21	[0.47; 3.10]	0.8%	2.1%
Chen 2019	7	70	7	48	0.69	[0.26; 1.83]	1.0%	2.0%
Shin 2019	3	56	6	270	2.41	[0.62; 9.35]	0.3%	1.3%
Ke 2020	37	75	84	225	1.32	[0.99; 1.76]	5.2%	4.7%
Liang 2020	11	27	8	19	0.97	[0.48; 1.94]	1.2%	2.9%
Yin 2021	11	26	114	119	0.44	[0.28; 0.69]	5.1%	4.0%
**Common effect model**		**314**		**752**	**0.93**	**[0.75; 1.14]**	**13.9%**	**−−**
**Random effects model**					**0.93**	**[0.60; 1.42]**	**−−**	**18.3%**
Heterogeneity: $I^2 = 68\%$, $\tau^2 = 0.1727$, $p < 0.01$								
**Common effect model**		**3254**		**2968**	**0.60**	**[0.55; 0.66]**	**100.0%**	**−−**
**Random effects model**					**0.60**	**[0.50; 0.71]**	**−−**	**100.0%**

Heterogeneity: $I^2 = 68\%$, $\tau^2 = 0.1507$, $p < 0.01$

Test for subgroup differences (fixed effect): $\chi^2_2 = 20.36$, df = 2 ($p < 0.01$)

Test for subgroup differences (random effects): $\chi^2_2 = 5.46$, df = 2 ($p = 0.07$)

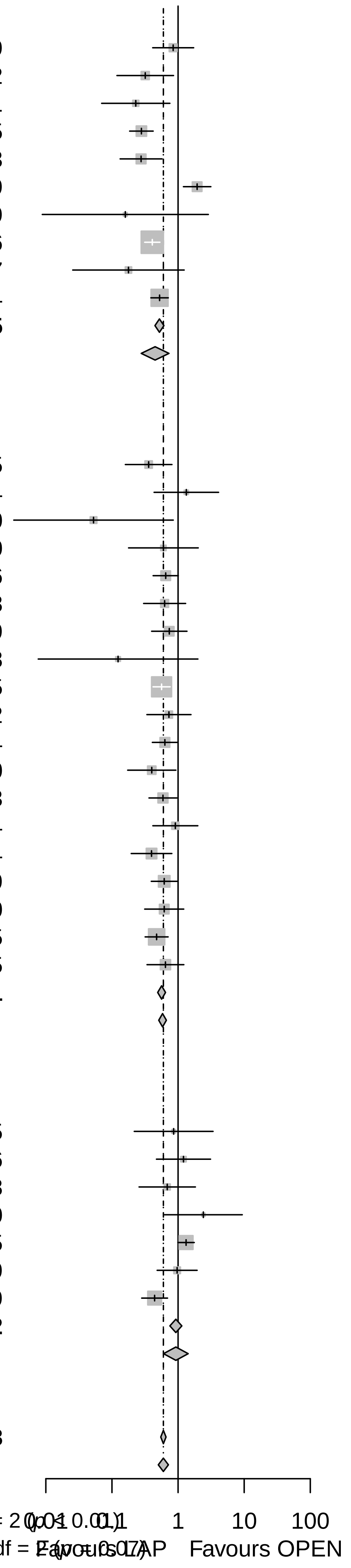

Q19 Length of stay

Study	LAP Total	LAP Mean	LAP SD	OPEN Total	OPEN Mean	OPEN SD	MD	95%−CI	Weight (common)	Weight (random)
Pessaux 2001	50	7.70	3.1000	89	12.70	5.8000	−5.00	[−6.48; −3.52]	0.9%	4.4%
Chau 2002	31	7.20	3.2000	42	10.60	6.1000	−3.40	[−5.56; −1.24]	0.4%	4.1%
Fisichella 2002	24	3.50	2.1000	11	7.60	3.1000	−4.10	[−6.12; −2.08]	0.5%	4.2%
Chan 2014	17	7.40	4.0000	34	8.00	25.0000	−0.60	[−9.22; 8.02]	0.0%	1.5%
Wang 2015	30	5.00	3.2500	60	10.00	3.7500	−5.00	[−6.50; −3.50]	0.9%	4.4%
Cauchi 2016	26	10.00	11.2500	26	14.00	20.0000	−4.00	[−12.82; 4.82]	0.0%	1.4%
Zeng 2016	79	10.00	4.3000	79	13.00	5.5000	−3.00	[−4.54; −1.46]	0.8%	4.4%
Amato 2017	11	3.20	0.4000	18	5.70	0.9000	−2.50	[−2.98; −2.02]	8.6%	4.7%
Aprea 2017	7	7.20	1.2000	15	11.30	4.0000	−4.10	[−6.31; −1.89]	0.4%	4.1%
Irojah 2017	791	5.00	3.1000	216	9.00	5.8000	−4.00	[−4.80; −3.20]	3.1%	4.6%
Lin 2017	21	5.70	2.3000	97	11.20	9.0000	−5.50	[−7.54; −3.46]	0.5%	4.2%
Martinez 2017	225	5.00	5.0000	225	8.00	12.0000	−3.00	[−4.70; −1.30]	0.7%	4.3%
Chapman 2018	248	10.00	1.3300	1520	10.00	1.4200	0.00	[−0.18; 0.18]	60.8%	4.7%
Goh 2018	32	2.00	0.1000	32	5.00	6.2500	−3.00	[−5.17; −0.83]	0.4%	4.1%
Souche 2018	53	14.00	10.0000	56	16.00	11.0000	−2.00	[−5.94; 1.94]	0.1%	3.2%
Badawy 2019	40	10.00	1.5000	40	23.00	2.5000	−13.00	[−13.90; −12.10]	2.4%	4.6%
Chen 2019	70	11.40	5.8000	48	15.10	6.7000	−3.70	[−6.03; −1.37]	0.4%	4.1%
Shin 2019	56	13.50	11.3000	270	16.50	11.3000	−3.00	[−6.25; 0.25]	0.2%	3.6%
Chen 2020	64	10.00	3.5000	64	12.00	4.7500	−2.00	[−3.45; −0.55]	0.9%	4.4%
Delvecchio 2020	38	6.00	2.2500	84	8.00	1.5000	−2.00	[−2.78; −1.22]	3.2%	4.6%
Dumronggittigule 2020	127	7.00	0.8300	49	11.00	1.8800	−4.00	[−4.55; −3.45]	6.6%	4.7%
Ke 2020	75	16.00	2.3300	225	14.00	1.3300	2.00	[1.44; 2.56]	6.4%	4.7%
Kim 2020	96	7.00	6.1700	160	10.00	28.6700	−3.00	[−7.61; 1.61]	0.1%	2.9%
Liang 2020	27	12.00	2.7500	19	23.25	7.2500	−11.25	[−14.67; −7.83]	0.2%	3.5%
Yin 2021	26	18.00	3.0000	119	24.00	2.3300	−6.00	[−7.23; −4.77]	1.3%	4.5%
**Common effect model**	**2264**			**3598**			**−1.22**	**[−1.36; −1.08]**	**100.0%**	**--**
**Random effects model**							**−3.85**	**[−5.12; −2.59]**	**--**	**100.0%**

Heterogeneity: $I^2 = 98\%$, $\tau^2 = 8.8845$, $p < 0.01$

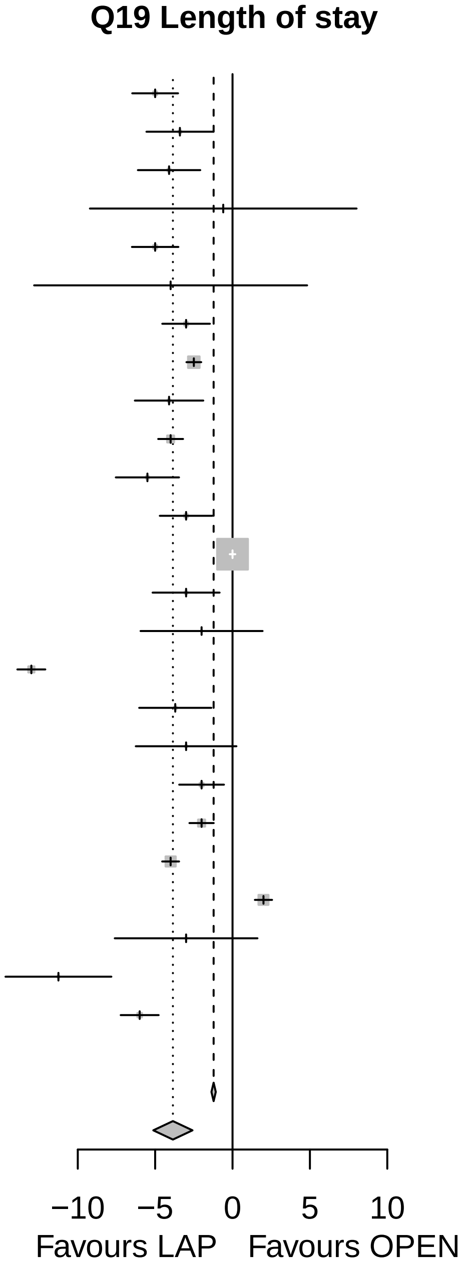

Q19 Length of stay - Subgroups

Study	LAP Total	LAP Mean	LAP SD	OPEN Total	OPEN Mean	OPEN SD	MD	95%−CI	Weight (common)	Weight (random)
**group = cholecyst**										
Pessaux 2001	50	7.70	3.1000	89	12.70	5.8000	−5.00	[−6.48; −3.52]	0.9%	4.4%
Chau 2002	31	7.20	3.2000	42	10.60	6.1000	−3.40	[−5.56; −1.24]	0.4%	4.1%
Fisichella 2002	24	3.50	2.1000	11	7.60	3.1000	−4.10	[−6.12; −2.08]	0.5%	4.2%
Irojah 2017	791	5.00	3.1000	216	9.00	5.8000	−4.00	[−4.80; −3.20]	3.1%	4.6%
Lin 2017	21	5.70	2.3000	97	11.20	9.0000	−5.50	[−7.54; −3.46]	0.5%	4.2%
**Common effect model**	**917**			**455**			**−4.26**	**[−4.87; −3.66]**	**5.4%**	**−−**
**Random effects model**							**−4.26**	**[−4.87; −3.66]**	**−−**	**21.6%**
Heterogeneity: $I^2 = 0\%$, $\tau^2 = 0$, $p = 0.49$										
**group = liver**										
Chan 2014	17	7.40	4.0000	34	8.00	25.0000	−0.60	[−9.22; 8.02]	0.0%	1.5%
Wang 2015	30	5.00	3.2500	60	10.00	3.7500	−5.00	[−6.50; −3.50]	0.9%	4.4%
Cauchi 2016	26	10.00	11.2500	26	14.00	20.0000	−4.00	[−12.82; 4.82]	0.0%	1.4%
Zeng 2016	79	10.00	4.3000	79	13.00	5.5000	−3.00	[−4.54; −1.46]	0.8%	4.4%
Amato 2017	11	3.20	0.4000	18	5.70	0.9000	−2.50	[−2.98; −2.02]	8.6%	4.7%
Martinez 2017	225	5.00	5.0000	225	8.00	12.0000	−3.00	[−4.70; −1.30]	0.7%	4.3%
Goh 2018	32	2.00	0.1000	32	5.00	6.2500	−3.00	[−5.17; −0.83]	0.4%	4.1%
Badawy 2019	40	10.00	1.5000	40	23.00	2.5000	−13.00	[−13.90; −12.10]	2.4%	4.6%
Chen 2020	64	10.00	3.5000	64	12.00	4.7500	−2.00	[−3.45; −0.55]	0.9%	4.4%
Delvecchio 2020	38	6.00	2.2500	84	8.00	1.5000	−2.00	[−2.78; −1.22]	3.2%	4.6%
Dumronggittigule 2020	127	7.00	0.8300	49	11.00	1.8800	−4.00	[−4.55; −3.45]	6.6%	4.7%
Kim 2020	96	7.00	6.1700	160	10.00	28.6700	−3.00	[−7.61; 1.61]	0.1%	2.9%
**Common effect model**	**785**			**871**			**−3.97**	**[−4.25; −3.69]**	**24.8%**	**−−**
**Random effects model**							**−3.98**	**[−5.97; −1.99]**	**−−**	**46.1%**
Heterogeneity: $I^2 = 98\%$, $\tau^2 = 10.2475$, $p < 0.01$										
**group = pancreas**										
Aprea 2017	7	7.20	1.2000	15	11.30	4.0000	−4.10	[−6.31; −1.89]	0.4%	4.1%
Chapman 2018	248	10.00	1.3300	1520	10.00	1.4200	0.00	[−0.18; 0.18]	60.8%	4.7%
Souche 2018	53	14.00	10.0000	56	16.00	11.0000	−2.00	[−5.94; 1.94]	0.1%	3.2%
Chen 2019	70	11.40	5.8000	48	15.10	6.7000	−3.70	[−6.03; −1.37]	0.4%	4.1%
Shin 2019	56	13.50	11.3000	270	16.50	11.3000	−3.00	[−6.25; 0.25]	0.2%	3.6%
Ke 2020	75	16.00	2.3300	225	14.00	1.3300	2.00	[1.44; 2.56]	6.4%	4.7%
Liang 2020	27	12.00	2.7500	19	23.25	7.2500	−11.25	[−14.67; −7.83]	0.2%	3.5%
Yin 2021	26	18.00	3.0000	119	24.00	2.3300	−6.00	[−7.23; −4.77]	1.3%	4.5%
**Common effect model**	**562**			**2272**			**−0.01**	**[−0.18; 0.16]**	**69.8%**	**−−**
**Random effects model**							**−3.36**	**[−6.12; −0.61]**	**−−**	**32.4%**
Heterogeneity: $I^2 = 97\%$, $\tau^2 = 14.2834$, $p < 0.01$										
**Common effect model**	**2264**			**3598**			**−1.22**	**[−1.36; −1.08]**	**100.0%**	**−−**
**Random effects model**							**−3.85**	**[−5.12; −2.59]**	**−−**	**100.0%**

Heterogeneity: $I^2 = 98\%$, $\tau^2 = 8.8845$, $p < 0.01$

Test for subgroup differences (fixed effect): $\chi^2_2 = 659.42$, df = 2 ($p < 0.01$)

Test for subgroup differences (random effects): $\chi^2_2 = 0.44$, df = 2 ($p = 0.80$)

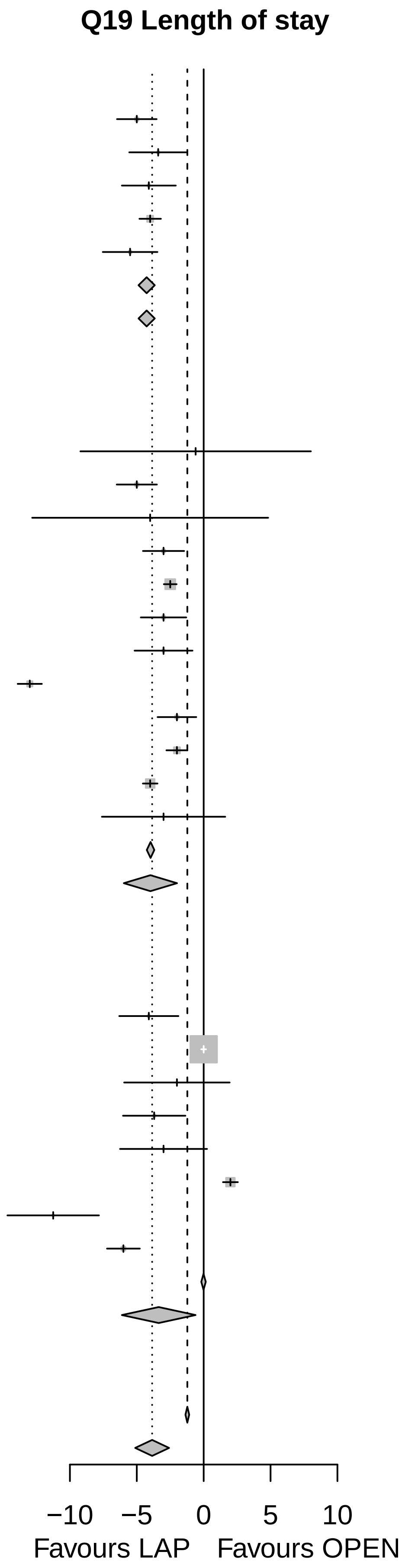

Q19 Readmission

Study	LAP Events	LAP Total	OPEN Events	OPEN Total	RR	95%−CI	Weight (common)	Weight (random)
Chapman 2018	24	248	199	1520	0.74	[0.49; 1.10]	80.2%	66.0%
Souche 2018	4	53	4	56	1.06	[0.28; 4.01]	5.6%	9.5%
Shin 2019	9	56	5	56	1.80	[0.64; 5.03]	7.2%	15.4%
Liang 2020	2	27	2	19	0.70	[0.11; 4.57]	3.4%	5.0%
Yin 2021	1	26	7	119	0.65	[0.08; 5.09]	3.6%	4.2%
**Common effect model**		**410**		**1770**	**0.83**	**[0.59; 1.17]**	**100.0%**	**−−**
**Random effects model**					**0.87**	**[0.57; 1.33]**	**−−**	**100.0%**

Heterogeneity: $I^2 = 0\%$, $\tau^2 = 0.0288$, $p = 0.61$

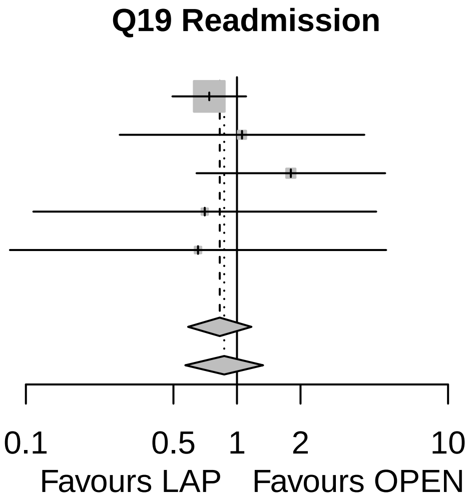

Q19 Readmission - Subgroups

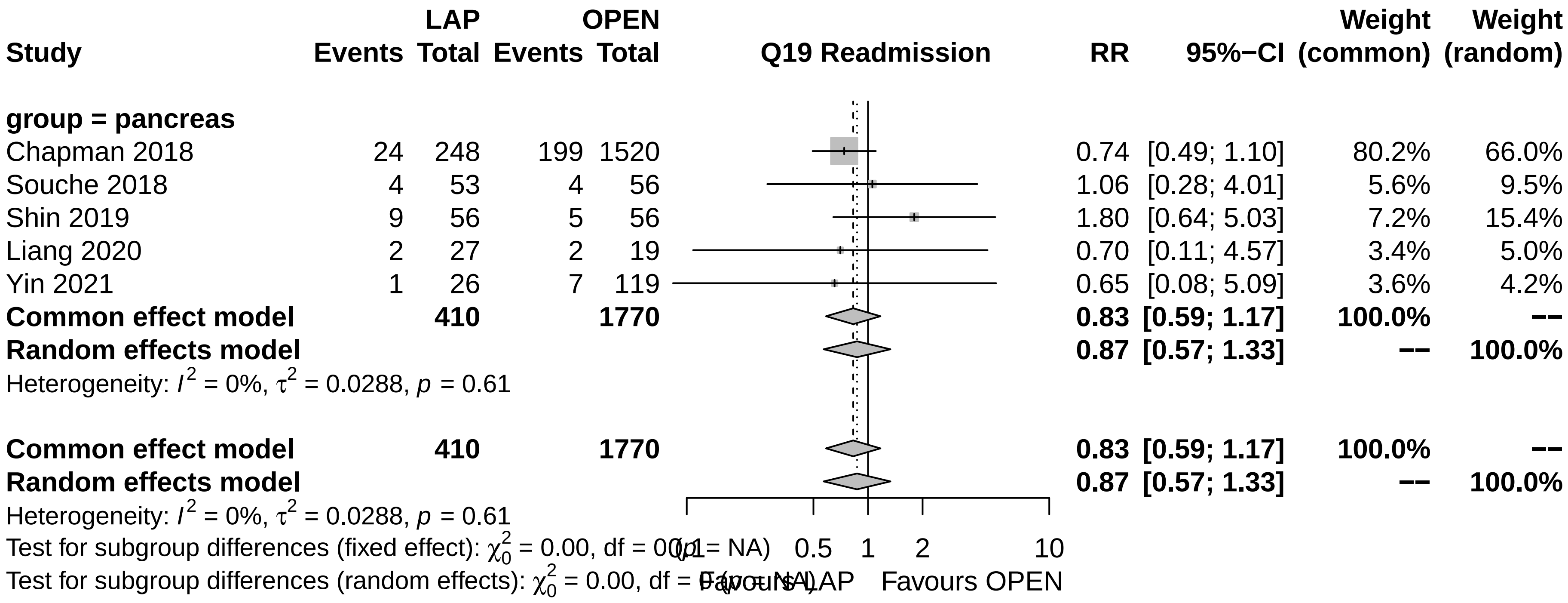

Q20 Complications

Study	LAP Events	LAP Total	OPEN Events	OPEN Total	RR	95%−CI	Weight (common)	Weight (random)
Hernandez Meno Rosa 2011	8	31	7	73	2.69	[1.07; 6.78]	0.3%	16.4%
Dallas 2013	2	31	11	84	0.49	[0.12; 2.10]	0.4%	10.2%
Neupane 2017	13	84	60	179	0.46	[0.27; 0.79]	2.8%	22.7%
Neupane 2017	13	84	60	179	0.46	[0.27; 0.79]	2.8%	22.7%
Aly 2020	842	14270	1297	14270	0.65	[0.60; 0.71]	93.7%	28.1%
**Common effect model**		**14500**		**14785**	**0.64**	**[0.59; 0.70]**	**100.0%**	**−−**
**Random effects model**					**0.68**	**[0.38; 1.22]**	**−−**	**100.0%**

Heterogeneity: I^2 = 67%, τ^2 = 0.3061, p = 0.02

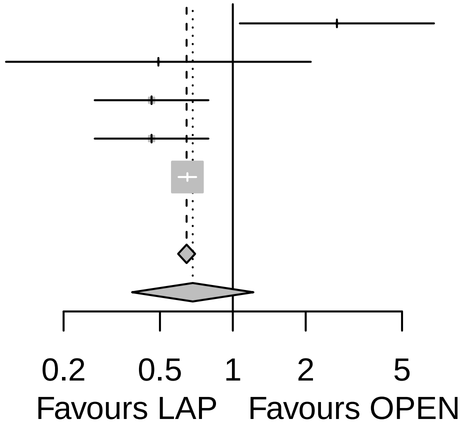

Q20 Complications - Subgroups

Study	LAP Events	LAP Total	OPEN Events	OPEN Total	RR	95%−CI	Weight (common)	Weight (random)
**group = inguinal**								
Hernandez Meno Rosa 2011	8	31	7	73	2.69	[1.07; 6.78]	0.3%	16.4%
Dallas 2013	2	31	11	84	0.49	[0.12; 2.10]	0.4%	10.2%
Neupane 2017	13	84	60	179	0.46	[0.27; 0.79]	2.8%	22.7%
**Common effect model**		**146**		**336**	**0.66**	**[0.43; 1.00]**	**3.5%**	**−−**
**Random effects model**					**0.86**	**[0.27; 2.77]**	**−−**	**49.2%**
Heterogeneity: $I^2 = 81\%$, $\tau^2 = 0.8209$, $p < 0.01$								
**group = ventral**								
Neupane 2017	13	84	60	179	0.46	[0.27; 0.79]	2.8%	22.7%
Aly 2020	842	14270	1297	14270	0.65	[0.60; 0.71]	93.7%	28.1%
**Common effect model**		**14354**		**14449**	**0.64**	**[0.59; 0.70]**	**96.5%**	**−−**
**Random effects model**					**0.61**	**[0.47; 0.79]**	**−−**	**50.8%**
Heterogeneity: $I^2 = 33\%$, $\tau^2 = 0.0191$, $p = 0.22$								
**Common effect model**		**14500**		**14785**	**0.64**	**[0.59; 0.70]**	**100.0%**	**−−**
**Random effects model**					**0.68**	**[0.38; 1.22]**	**−−**	**100.0%**

Heterogeneity: $I^2 = 67\%$, $\tau^2 = 0.3061$, $p = 0.02$

Test for subgroup differences (fixed effect): $\chi^2_1 = 0.01$, df = 1 ($p = 0.92$)

Test for subgroup differences (random effects): $\chi^2_1 = 0.31$, df = 1 ($p = 0.58$)

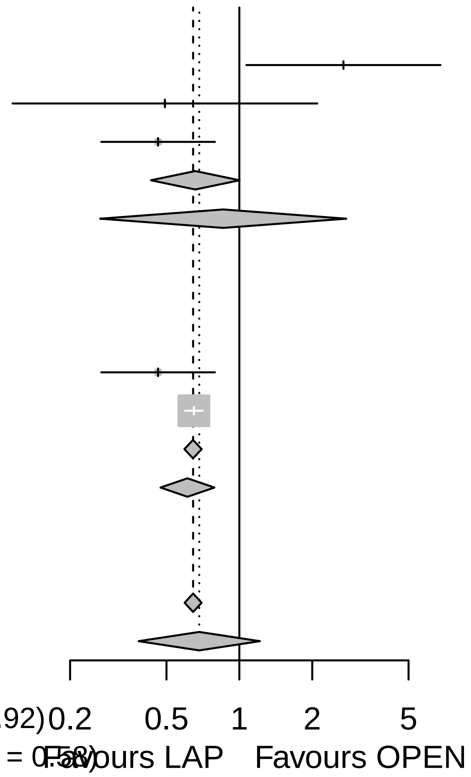

Q20 Length of stay

Study	LAP Total	LAP Mean	LAP SD	OPEN Total	OPEN Mean	OPEN SD	MD	95%−CI	Weight (common)	Weight (random)
Hernandez Meno Rosa 2011	31	0.60	1.4200	73	0.50	3.9200	0.10	[−0.93; 1.13]	0.0%	20.2%
Dallas 2013	31	7.50	3.0000	84	23.10	12.3000	−15.60	[−18.43; −12.77]	0.0%	19.3%
Neupane 2017	84	3.30	1.8300	179	7.70	4.8300	−4.40	[−5.21; −3.59]	0.0%	20.3%
Payziwula 2019	7	6.00	1.0000	11	12.00	3.0000	−6.00	[−7.92; −4.08]	0.0%	19.9%
Aly 2020	14270	1.00	0.3300	14270	1.00	0.5000	0.00	[−0.01; 0.01]	100.0%	20.4%
**Common effect model**	**14423**			**14617**			**−0.00**	**[−0.01; 0.01]**	**100.0%**	**−−**
**Random effects model**							**−5.08**	**[−10.60; 0.44]**	**−−**	**100.0%**

Heterogeneity: $I^2 = 99\%$, $\tau^2 = 38.9793$, $p < 0.01$

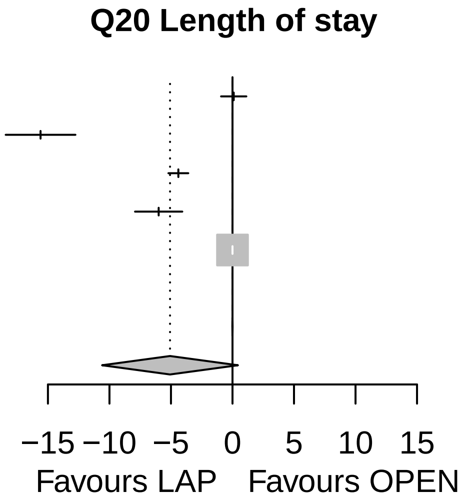

Q20 Length of stay - Subgroups

Study	LAP Total	LAP Mean	LAP SD	OPEN Total	OPEN Mean	OPEN SD	MD	95%−CI	Weight (common)	Weight (random)
**group = inguinal**										
Hernandez Meno Rosa 2011	31	0.60	1.4200	73	0.50	3.9200	0.10	[−0.93; 1.13]	0.0%	20.2%
Dallas 2013	31	7.50	3.0000	84	23.10	12.3000	−15.60	[−18.43; −12.77]	0.0%	19.3%
Payziwula 2019	7	6.00	1.0000	11	12.00	3.0000	−6.00	[−7.92; −4.08]	0.0%	19.9%
**Common effect model**	**69**			**168**			**−2.59**	**[−3.46; −1.73]**	**0.0%**	**--**
**Random effects model**							**−7.09**	**[−16.02; 1.84]**	**--**	**59.4%**
Heterogeneity: $I^2 = 98\%$, $\tau^2 = 61.1315$, $p < 0.01$										
**group = ventral**										
Neupane 2017	84	3.30	1.8300	179	7.70	4.8300	−4.40	[−5.21; −3.59]	0.0%	20.3%
Aly 2020	14270	1.00	0.3300	14270	1.00	0.5000	0.00	[−0.01; 0.01]	100.0%	20.4%
**Common effect model**	**14354**			**14449**			**−0.00**	**[−0.01; 0.01]**	**100.0%**	**--**
**Random effects model**							**−2.18**	**[−6.49; 2.13]**	**--**	**40.6%**
Heterogeneity: $I^2 = 99\%$, $\tau^2 = 9.5949$, $p < 0.01$										
**Common effect model**	**14423**			**14617**			**−0.00**	**[−0.01; 0.01]**	**100.0%**	**--**
**Random effects model**							**−5.08**	**[−10.60; 0.44]**	**--**	**100.0%**

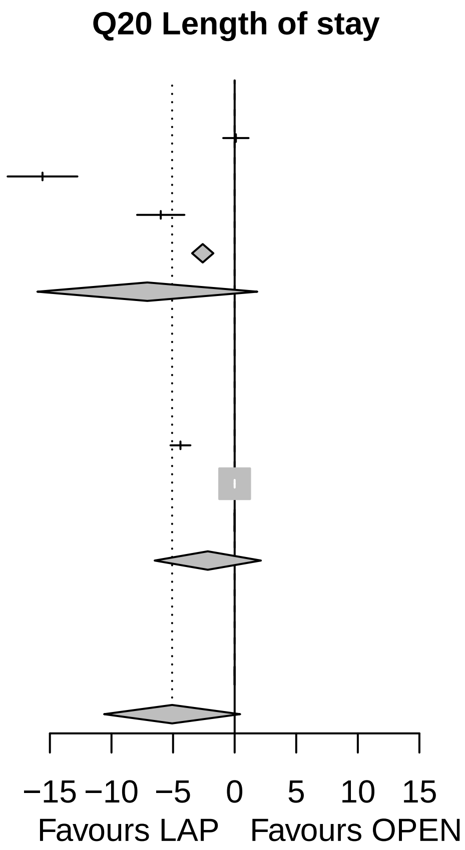

Heterogeneity: $I^2 = 99\%$, $\tau^2 = 38.9793$, $p < 0.01$

Test for subgroup differences (fixed effect): $\chi^2_1 = 34.54$, df = 1 ($p < 0.01$)

Test for subgroup differences (random effects): $\chi^2_1 = 0.94$, df = 1 ($p = 0.33$)

Q21 – ERAS versus Conventional Care for Colorectal Surgery in Elderly Patients

Q21 30-Day Complications

Study or Subgroup	ERAS Events	ERAS Total	Conventional Care Events	Conventional Care Total	Weight	Odds Ratio M-H, Random, 95% CI
Boon 2020	41	66	121	153	14.4%	0.43 [0.23, 0.82]
Kocian 2019	40	74	34	61	12.8%	0.93 [0.47, 1.85]
Lirosi 2019	19	61	20	53	10.3%	0.75 [0.34, 1.62]
Meillat 2020	27	131	48	135	17.7%	0.47 [0.27, 0.82]
Ostermann 2019	26	75	49	75	13.1%	0.28 [0.14, 0.55]
Tejedor 2018	16	156	34	156	14.1%	0.41 [0.22, 0.78]
Zeng 2018	25	94	70	157	17.5%	0.45 [0.26, 0.78]
Total (95% CI)		657		790	100.0%	0.48 [0.37, 0.64]
Total events	194		376			

Heterogeneity: Tau² = 0.03; Chi² = 7.71, df = 6 (P = 0.26); I² = 22%
Test for overall effect: Z = 5.21 (P < 0.00001)

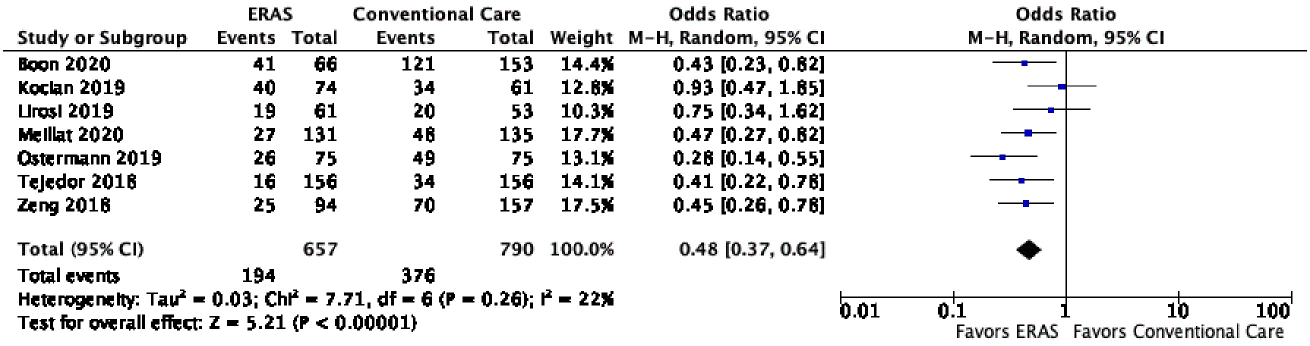

Q21 Length of Stay

Study or Subgroup	ERAS Mean	ERAS SD	ERAS Total	Conventional Care Mean	Conventional Care SD	Conventional Care Total	Weight	Mean Difference IV, Random, 95% CI
Boon 2020	9	6.3	66	10	6.3	153	7.9%	−1.00 [−2.82, 0.82]
Kocian 2019	10.3	7.1	74	11.9	4.8	61	6.9%	−1.60 [−3.62, 0.42]
Lirosi 2019	5.4	2.7	61	6.3	2.4	53	14.7%	−0.90 [−1.84, 0.04]
Mari 2016	4.7	1.2	43	6	1.6	40	17.8%	−1.30 [−1.91, −0.69]
Meillat 2020	5	9	131	7	18	135	3.1%	−2.00 [−5.41, 1.41]
Ostermann 2019	7	4.6	75	12	6.1	75	8.4%	−5.00 [−6.73, −3.27]
Tejedor 2018	6	3.7	156	8.5	4	156	15.5%	−2.50 [−3.36, −1.64]
Wang 2011	5.5	0.7	40	7	1.5	38	18.6%	−1.50 [−2.02, −0.98]
Zeng 2018	6	5.7	94	8	10	157	7.2%	−2.00 [−3.94, −0.06]
Total (95% CI)			740			868	100.0%	−1.84 [−2.49, −1.20]

Heterogeneity: Tau² = 0.51; Chi² = 22.90, df = 8 (P = 0.003); I² = 65%
Test for overall effect: Z = 5.62 (P < 0.00001)

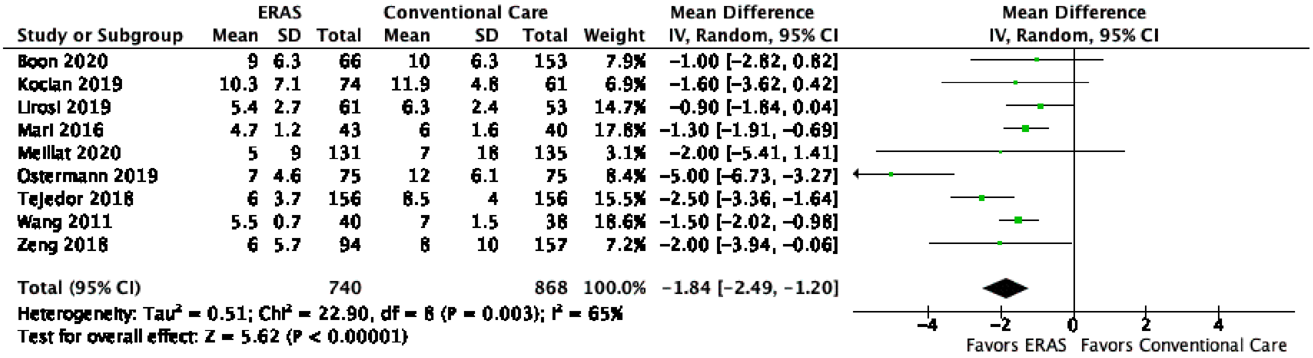

Q21 Readmissions

Study or Subgroup	ERAS Events	ERAS Total	Conventional Care Events	Conventional Care Total	Weight	Odds Ratio M-H, Random, 95% CI
Boon 2020	6	66	9	153	22.8%	1.60 [0.55, 4.69]
Kocian 2019	2	74	1	61	4.5%	1.67 [0.15, 18.83]
Lirosi 2019	3	61	2	53	7.9%	1.32 [0.21, 8.21]
Meillat 2020	6	131	6	135	19.7%	1.03 [0.32, 3.29]
Ostermann 2019	6	75	5	75	17.4%	1.22 [0.35, 4.18]
Tejedor 2018	7	156	11	156	27.8%	0.62 [0.23, 1.64]
Total (95% CI)		563		633	100.0%	1.06 [0.63, 1.77]
Total events	30		34			

Heterogeneity: Tau² = 0.00; Chi² = 1.97, df = 5 (P = 0.85); I² = 0%
Test for overall effect: Z = 0.23 (P = 0.82)

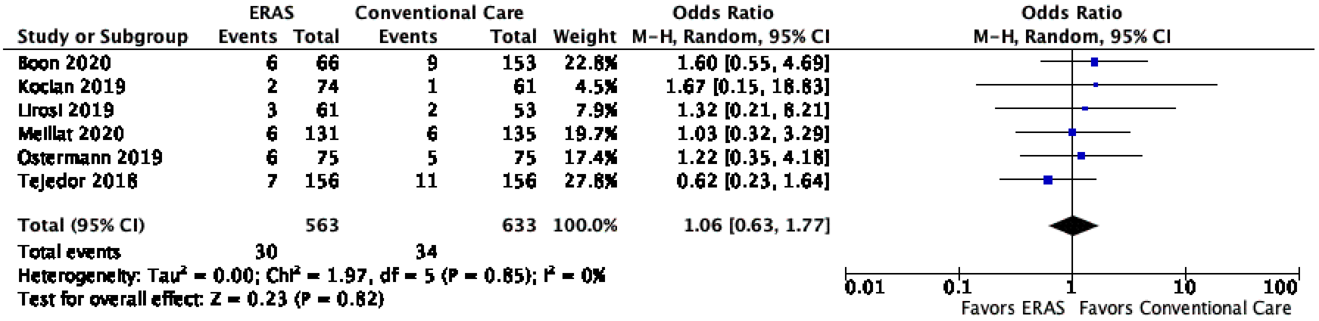

Q22 – ERAS versus Conventional Care for Gastric Surgery in Elderly Patients

Q22 30-day Complications

	ERAS		Conventional Care			Odds Ratio
Study or Subgroup	Events	Total	Events	Total	Weight	M-H, Random, 95% CI
Bu 2015	45	64	31	64	49.4%	2.52 [1.22, 5.21]
Cao 2016	29	85	48	86	50.6%	0.41 [0.22, 0.76]
Total (95% CI)		149		150	100.0%	1.01 [0.17, 5.97]
Total events	74		79			

Heterogeneity: $Tau^2 = 1.53$; $Chi^2 = 13.93$, df = 1 ($P = 0.0002$); $I^2 = 93\%$
Test for overall effect: $Z = 0.01$ ($P = 0.99$)

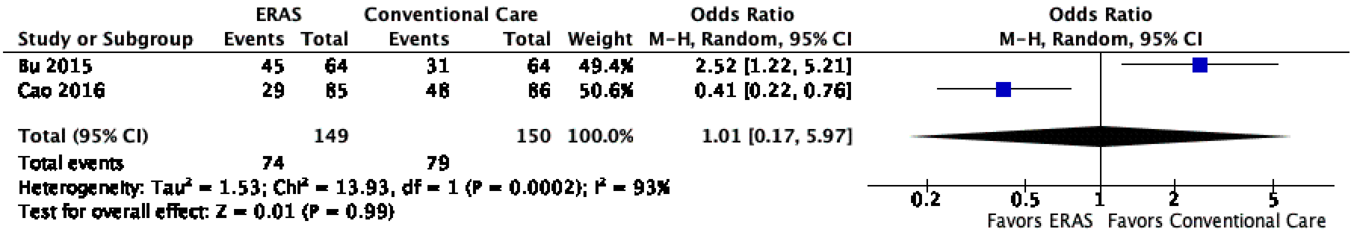

Q22 Length of Stay

	ERAS			Conventional Care				Mean Difference
Study or Subgroup	Mean	SD	Total	Mean	SD	Total	Weight	IV, Random, 95% CI
Bu 2015	10	2.3	64	10.8	2.5	64	97.5%	−0.80 [−1.63, 0.03]
Cao 2016	11	7.97	85	13	23.5	86	2.5%	−2.00 [−7.25, 3.25]
Total (95% CI)			149			150	100.0%	−0.83 [−1.65, −0.01]

Heterogeneity: $Tau^2 = 0.00$; $Chi^2 = 0.20$, df = 1 ($P = 0.66$); $I^2 = 0\%$
Test for overall effect: $Z = 1.98$ ($P = 0.05$)

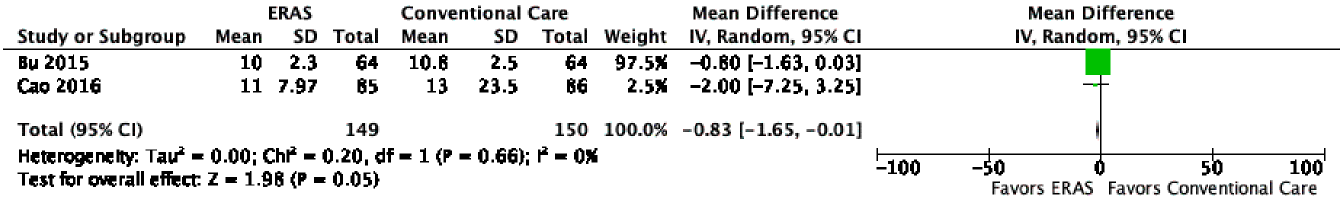

Q22 Readmissions

	ERAS		Conventional Care			Odds Ratio
Study or Subgroup	Events	Total	Events	Total	Weight	M-H, Random, 95% CI
Bu 2015	12	64	3	64	77.1%	4.69 [1.26, 17.53]
Cao 2016	2	85	1	86	22.9%	2.05 [0.18, 23.02]
Total (95% CI)		149		150	100.0%	3.88 [1.22, 12.35]
Total events	14		4			

Heterogeneity: $Tau^2 = 0.00$; $Chi^2 = 0.35$, df = 1 ($P = 0.56$); $I^2 = 0\%$
Test for overall effect: $Z = 2.30$ ($P = 0.02$)

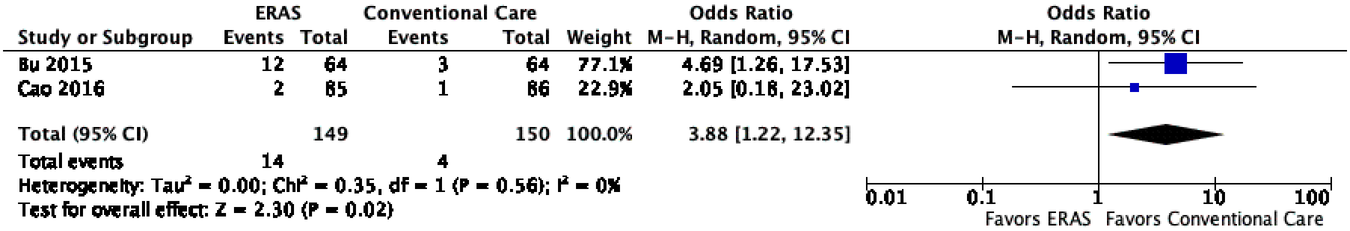

Q23 – ERAS versus Conventional Care for HPB Surgery in Elderly Patients

Q23 30-day Complications

Study or Subgroup	ERAS Events	ERAS Total	Conventional Care Events	Conventional Care Total	Weight	Odds Ratio M-H, Random, 95% CI
Jiang 2020	6	70	27	107	51.3%	0.28 [0.11, 0.71]
Partelli 2016	17	22	43	66	48.7%	1.82 [0.59, 5.56]
Total (95% CI)		92		173	100.0%	0.69 [0.11, 4.37]
Total events	23		70			

Heterogeneity: $Tau^2 = 1.49$; $Chi^2 = 6.34$, df = 1 (P = 0.01); $I^2 = 84\%$
Test for overall effect: Z = 0.39 (P = 0.70)

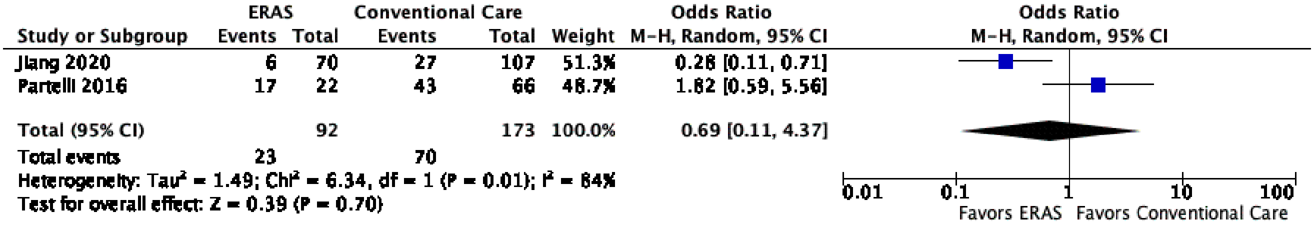

Q23 Length of Stay

Study or Subgroup	ERAS Mean	ERAS SD	ERAS Total	Conventional Care Mean	Conventional Care SD	Conventional Care Total	Weight	Mean Difference IV, Fixed, 95% CI
Jiang 2020	6	7.94	70	9	13.86	107	58.2%	-3.00 [-6.22, 0.22]
Partelli 2016	8	5.9	22	8	6.9	22	41.8%	0.00 [-3.79, 3.79]
Total (95% CI)			92			129	100.0%	-1.74 [-4.20, 0.71]

Heterogeneity: $Chi^2 = 1.40$, df = 1 (P = 0.24); $I^2 = 28\%$
Test for overall effect: Z = 1.39 (P = 0.16)

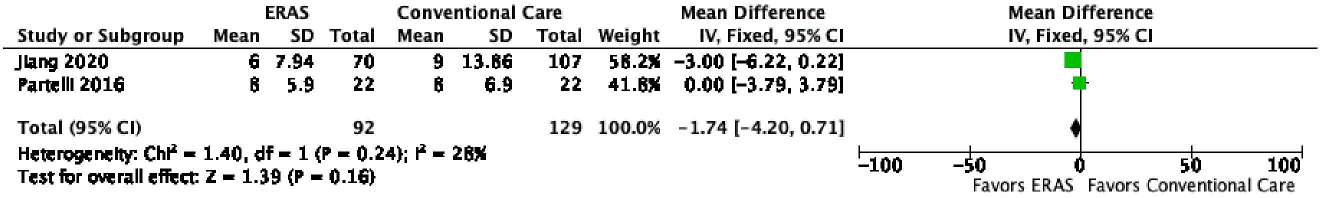

Q23 Readmissions

Study or Subgroup	ERAS Events	ERAS Total	Conventional Care Events	Conventional Care Total	Weight	Odds Ratio M-H, Random, 95% CI
Jiang 2020	1	70	4	107	28.0%	0.37 [0.04, 3.41]
Partelli 2016	11	66	3	22	72.0%	1.27 [0.32, 5.03]
Total (95% CI)		136		129	100.0%	0.90 [0.28, 2.90]
Total events	12		7			

Heterogeneity: $Tau^2 = 0.00$; $Chi^2 = 0.85$, df = 1 (P = 0.36); $I^2 = 0\%$
Test for overall effect: Z = 0.18 (P = 0.86)

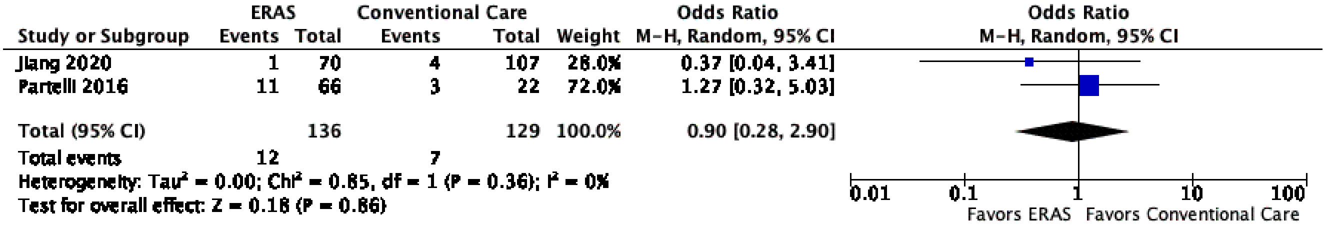

Q24 – ERAS versus Conventional Care for Foregut Surgery in Elderly Patients

No studies found
