# Supplementary material for: EAES/SAGES evidence-based recommendations and expert consensus on optimization of perioperative care in older adults
Source: Surg Endosc. 2024 Jun 28;38(8):4104–26. doi: 10.1007/s00464-024-10977-7 (PMC11289045; doi:10.1007/s00464-024-10977-7)
Supplement: Supplementary file 6 — Supplement 6 Quality assessment for KQ17-KQ24 Supplementary file6 (DOCX 39 KB) [file 464_2024_10977_MOESM6_ESM.docx]

Supplement 6: Quality assessment for KQ17-KQ24

| Table 1. Risk of bias for the studies included under KQ17. | | |  |
| --- | --- | --- | --- |
| **Author, Year** | **Type of Study** | **Cochrane RoB Tool used** | **Final Risk of Bias** |
| Akiyoshi 2009 | Observational | ROBINS-I | Serious |
| Allerdyce 2010 | Observational | ROBINS-I | Low |
| Altuntas 2012 | Observational | ROBINS-I | Serious |
| Baek 2011 | Observational | ROBINS-I | Serious |
| Chang 2019 | Observational | ROBINS-I | Low |
| Chen 2015 | Observational | ROBINS-I | Serious |
| Chen 2018 | Observational | ROBINS-I | Serious |
| Chern 2018 | Observational | ROBINS-I | Serious |
| Chern 2020 | Observational | ROBINS-I | Moderate |
| Chung 2021 | Observational | ROBINS-I | Low |
| Clark III 2012 | Observational | ROBINS-I | Low |
| Cocorullo 2016 | Observational | ROBINS-I | Critical |
| Cummings 2021 (2012) | Observational | ROBINS-I | Low |
| Daniel 2019 | Observational | ROBINS-I | Low |
| De Angelis 2018 | Observational | ROBINS-I | Low |
| Delgado 2000 | RCT | RoB2 | Moderate |
| Devoto 2017 | Observational | ROBINS-I | Serious |
| Duan 2018 | Observational | ROBINS-I | Low |
| Feng 2006 | Observational | ROBINS-I | Moderate |
| Frasson 2008 | RCT | RoB2 | Low |
| Fujii 2014 | RCT | RoB 2 | High |
| Guida 2015 | Observational | ROBINS-I | Low |
| Hashida 2020 | Observational | ROBINS-I | Moderate |
| Hatakeyama 2013 | Observational | ROBINS-I | Low |
| Hester 2007 (Cheung) | Observational | ROBINS-I | Moderate |
| Hinoi 2015 | Observational | ROBINS-I | Low |
| Horsey 2021 (2022) | Observational | ROBINS-I | Low |
| Huang 2021 | Observational | ROBINS-I | Low |
| Inoue 2019 | Observational | ROBINS-I | Low |
| Issa 2011 | Observational | ROBINS-I | Moderate |
| Jia 2015 | Observational | ROBINS-I | Low |
| Kannan 2015 | Observational | ROBINS-I | Low |
| Keller 2021 | Observational | ROBINS-I | Low |
| Kennedy 2011 | Observational | ROBINS-I | Serious |
| Key 2019 (Kye) | Observational | ROBINS-I | Moderate |
| Kohn 2015 | Observational | ROBINS-I | Moderate |
| Kurian 2010 | Observational | ROBINS-I | Moderate |
| Landi 2016 | Observational | ROBINS-I | Moderate |
| Law 2002 | Observational | ROBINS-I | Moderate |
| Li 2016 | Observational | ROBINS-I | Serious |
| Lian 2010 | Observational | ROBINS-I | Low |
| Matsuoka 2004 | Observational | ROBINS-I | Moderate |
| Miguchi 2018 | Observational | ROBINS-I | Low |
| Miguchi 2021 | Observational | ROBINS-I | Moderate |
| Miyasaka 2014 | Observational | ROBINS-I | Serious |
| Moazzez 2013 | Observational | ROBINS-I | Low |
| Moon 2016 | Observational | ROBINS-I | Moderate |
| Mukai 2014 | Observational | ROBINS-I | Serious |
| Nakamura 2014 | Observational | ROBINS-I | Serious |
| Nishikawa 2016 | Observational | ROBINS-I | Moderate |
| Nishikawa 2019 | Observational | ROBINS-I | Low |
| Niitsu 2015 (2016) | Observational | ROBINS-I | Moderate |
| Person 2006 (2008) | Observational | ROBINS-I | Moderate |
| Pinto 2011 | Observational | ROBINS-I | Moderate |
| Richards 2019 | Observational | ROBINS-I | Low |
| Rinaldi 2017 | Observational | ROBINS-I | Low |
| Scarpa 2013 | Observational | ROBINS-I | Low |
| She 2013 | Observational | ROBINS-I | Low |
| Shigeta 2016 | Observational | ROBINS-I | Moderate |
| Sklow 2003 | Observational | ROBINS-I | Moderate |
| Sotirova 2020 | Observational | ROBINS-I | Serious |
| Stocchi 2020 (2000) | Observational | ROBINS-I | Moderate |
| Tan 2012 | Observational | ROBINS-I | Serious |
| Tei 2010 | Observational | ROBINS-I | Serious |
| Tomimaru 2010 (2011) | Observational | ROBINS-I | Moderate |
| Tominaga 2015 | Observational | ROBINS-I | Moderate |
| Troian 2018 | Observational | ROBINS-I | Low |
| Tuech 2000 | Observational | ROBINS-I | Moderate |
| Ueda 2020 | Observational | ROBINS-I | Low |
| VallriberaValls 2014 | Observational | ROBINS-I | Low |
| van Harten 2020 | Observational | ROBINS-I | Low |
| Vignali 2005 | Observational | ROBINS-I | Moderate |
| Wang 2006 | Observational | ROBINS-I | Serious |
| Wang 2016 | Observational | ROBINS-I | Low |
| Wei 2018 | Observational | ROBINS-I | Serious |
| Weng 2015 (Zeng) | Observational | ROBINS-I | Moderate |
| White 2021 (2012) | Observational | ROBINS-I | Moderate |
| Wu 2011 | Observational | ROBINS-I | Serious |
| Wu 2017 | Observational | ROBINS-I | Critical |
| Yamamoto 2017 | Observational | ROBINS-I | Low |
| Yap 2016 | Observational | ROBINS-I | Moderate |
| Zhou 2019 | Observational | ROBINS-I | Serious |
|  |  |  |  |
| Table 2. Risk of bias for the studies included under KQ18. | | |  |
| **Author, Year** | **Type of Study** | **Cochrane RoB Tool used** | **Final Risk of Bias** |
| Abbassi-Ghadi 2020 | Observational | Newcastle-Ottawa | Low |
| Chen 2021 | Observational | Newcastle-Ottawa | Low |
| Costa 2020 | Observational | Newcastle-Ottawa | Low |
| Fransvea 2020 | Observational | Newcastle-Ottawa | Moderate |
| Inokuchi 2018 | Observational | Newcastle-Ottawa | Moderate |
| Kim 2018 | Observational | Newcastle-Ottawa | Moderate |
| Konishi 2017 | Observational | Newcastle-Ottawa | Moderate |
| Li 2014 | Observational | Newcastle-Ottawa | Low |
| Liu 2017 | Observational | Newcastle-Ottawa | Moderate |
| Lu 2015 | Observational | Newcastle-Ottawa | Low |
| Mochiki 2005 | Observational | Newcastle-Ottawa | Low |
| Molena 2014 | Observational | Newcastle-Ottawa | Low |
| Pan 2018 | Observational | Newcastle-Ottawa | Low |
| Qiu 2014 | Observational | Newcastle-Ottawa | Low |
| Tsuchiya 2018 | Observational | Newcastle-Ottawa | Low |
| Ushimaru 2020 | Observational | Newcastle-Ottawa | Low |
| Wu 2016 | Observational | Newcastle-Ottawa | Low |
| Zheng 2016 | Observational | Newcastle-Ottawa | Low |
|  |  |  |  |
| Table 3. Risk of bias for the studies included under KQ19. | | |  |
| **Author, Year** | **Type of Study** | **Cochrane RoB Tool used** | **Final Risk of Bias** |
| Amato 2017 | Observational | Newcastle-Ottawa | High |
| Aprea 2017 | Observational | Newcastle-Ottawa | Moderate |
| Badawy 2019 | Observational | Newcastle-Ottawa | Moderate |
| Cauchy 2016 | Observational | Newcastle-Ottawa | Moderate |
| Chan 2014 | Observational | Newcastle-Ottawa | Moderate |
| Chapman 2018 | Observational | Newcastle-Ottawa | Low |
| Chau 2002 | Observational | Newcastle-Ottawa | Moderate |
| Chen 2019 | Observational | Newcastle-Ottawa | Moderate |
| Chen 2020 | Observational | Newcastle-Ottawa | Low |
| Cocorullo 2016 | Observational | Newcastle-Ottawa | High |
| Costa 2020 | Observational | Newcastle-Ottawa | Low |
| Delvecchio 2020 (2021) | Observational | Newcastle-Ottawa | Low |
| Dumronggittigule 2020 | Observational | Newcastle-Ottawa | Low |
| Fisichella 2002 | Observational | Newcastle-Ottawa | Moderate |
| Goh 2018 | Observational | Newcastle-Ottawa | Moderate |
| Gurgenidze 2013 | Observational | Newcastle-Ottawa | Moderate |
| Harada 2016 | Observational | n/a | n/a |
| Irojah 2017 | Observational | n/a | n/a |
| Ke 2020 | Observational | Newcastle-Ottawa | Moderate |
| Kim 2020 | Observational | Newcastle-Ottawa | Low |
| Le Roux 2018 | Observational | n/a | n/a |
| Leardi 2009 | Observational | n/a | n/a |
| Liang 2020 | Observational | Newcastle-Ottawa | Low |
| Lin 2017 | Observational | Newcastle-Ottawa | Moderate |
| Martínez-Cecilia 2017 | Observational | Newcastle-Ottawa | Low |
| Moyson 2008 | Observational | n/a | n/a |
| Nomi 2020 | Observational | Newcastle-Ottawa | Low |
| Pessaux 2001 | Observational | Newcastle-Ottawa | Moderate |
| Shin 2019 | Observational | Newcastle-Ottawa | Low |
| Souche 2018 | Observational | Newcastle-Ottawa | Moderate |
| Tanaka 2015 | Observational | Newcastle-Ottawa | Low |
| Tee 2020 | Observational | Newcastle-Ottawa | Low |
| Untereiner 2019 | Observational | n/a | n/a |
| Vanounou 2010 | Observational | n/a | n/a |
| Wang 2015 | Observational | Newcastle-Ottawa | Moderate |
| Yin 2021 | Observational | Newcastle-Ottawa | Moderate |
| Zeng 2016 | Observational | Newcastle-Ottawa | Low |
|  |  |  |  |
| Table 4. Risk of bias for the studies included under KQ20. | | |  |
| **Author, Year** | **Type of Study** | **Cochrane RoB Tool used** | **Final Risk of Bias** |
| Aly 2020 (2021) | Observational | Newcastle-Ottawa | Low |
| Dallas 2013 | Observational | Newcastle-Ottawa | High |
| Hernandez-Rosa 2011 | Observational | Newcastle-Ottawa | High |
| Neupane 2017 | Observational | Newcastle-Ottawa | High |
| Payiziwula 2019 | Observational | Newcastle-Ottawa | Unclear |
|  |  |  |  |
| Table 5. Risk of bias for the studies included under KQ21. | | |  |
| **Author, Year** | **Type of Study** | **Cochrane RoB Tool used** | **Final Risk of Bias** |
| Boon 2020 (2021) | Observational | Newcastle-Ottawa | High |
| Kocian 2019 (2020) | Observational | Newcastle-Ottawa | Low |
| Lirosi 2019 | Observational | Newcastle-Ottawa | Low |
| Mari 2016 | RCT | RoB2 | High |
| Meillat 2020 (2021) | Observational | Newcastle-Ottawa | Low |
| Ostermann 2019 | RCT | RoB2 | High |
| Tejedor 2018 | Observational | Newcastle-Ottawa | High |
| Wang 2011 (2012) | Observational | Newcastle-Ottawa | High |
| Zeng 2018 | Observational | Newcastle-Ottawa | High |
|  |  |  |  |
| Table 6. Risk of bias for the studies included under KQ22. | | |  |
| **Author, Year** | **Type of Study** | **Cochrane RoB Tool used** | **Final Risk of Bias** |
| Bu 2015 | RCT | RoB2 | Low |
| Cao 2016 (2021) | RCT | RoB2 | High |
|  |  |  |  |
| Table 7. Risk of bias for the studies included under KQ23. | | |  |
| **Author, Year** | **Type of Study** | **Cochrane RoB Tool used** | **Final Risk of Bias** |
| Jiang 2020 | Observational | Newcastle-Ottawa | High |
| Partelli 2016 | Observational | Newcastle-Ottawa | High |
|  |  |  |  |
| Table 8. Risk of bias for the studies included under KQ24. | | |  |
| None |  |  |  |

**References**

Newcastle-Ottawa

Wells G, Shea B, O'Connell D, Peterson J, Welch V, Losos M, Tugwell P (2000) The Newcastle-Otawa Scale (NOS) for assessing the quality of nonrandomised studies in meta-analyses. Otawa, Ontario: Otawa Hospital Research Institute.

RoB2

Higgins JP, Altman DG, Gøtzsche PC, Jüni P, Moher D, Oxman AD, Savovic J, Schulz KF, Weeks L, Sterne JA; Cochrane Bias Methods Group; Cochrane Sta􀆟s􀆟cal Methods Group (2011) The Cochrane Collabora􀆟on’s tool for assessing risk of bias in randomised trials. BMJ 343:d5928.

ROBINS-1

Sterne JAC, Hernán MA, Reeves BC, Savović J, Berkman ND, Viswanathan M, Henry D, Altman DG, Ansari MT, Boutron I, Carpenter JR, Chan AW, Churchill R, Deeks JJ, Hróbjartsson A, Kirkham J, Jüni P, Loke YK, Pigot TD, Ramsay CR, Regidor D, Rothstein HR, Sandhu L, Santaguida PL, Schünemann HJ, Shea B, Shrier I, Tugwell P, Turner L, Valen􀆟ne JC, Waddington H, Waters E, Wells GA, Whi􀆟ng PF, Higgins JPT. ROBINS-I: a tool for assessing risk of bias in non-randomized studies of interven􀆟ons. BMJ 2016; 355; i4919.
